# Supplementary material for: Emotional tones of voice affect the acoustics and perception of Mandarin tones
Source: PLoS One. 2023 Apr 5;18(4):e0283635. doi: 10.1371/journal.pone.0283635 (PMC10075469; doi:10.1371/journal.pone.0283635)
Supplement: S1 Table — (DOCX) [file pone.0283635.s001.docx]

1. Mean F0

| **Main effects** |  | Chisq | Df | Pr(>Chisq) |  |  |  |
| --- | --- | --- | --- | --- | --- | --- | --- |
|  | Tone | 973.9485873 | 3 | 0.00000 |  |  |  |
|  | Emotion | 1749.663312 | 4 | 0.00000 |  |  |  |
|  | Tone:Emotion | 70.18038279 | 12 | 0.00000 |  |  |  |
| **Interaction** |  |  |  |  |  |  |  |
| **tone*emotion** | contrast | Tone | estimate | SE | df | t.ratio | p.value |
|  | anger - fear | 1 | 85.055125 | 9.554426671 | 930 | 8.902169427 | 0 |
|  | anger - happiness | 1 | 93.60514583 | 9.554426671 | 930 | 9.797044769 | 0 |
|  | anger - neutral | 1 | 195.3258958 | 9.554426671 | 930 | 20.4434973 | 0 |
|  | anger - sadness | 1 | 94.9766875 | 9.554426671 | 930 | 9.940595158 | 0 |
|  | fear - happiness | 1 | 8.550020833 | 9.554426671 | 930 | 0.894875342 | 0.898831322 |
|  | fear - neutral | 1 | 110.2707708 | 9.554426671 | 930 | 11.54132787 | 0 |
|  | fear - sadness | 1 | 9.9215625 | 9.554426671 | 930 | 1.038425731 | 0.837457698 |
|  | happiness - neutral | 1 | 101.72075 | 9.554426671 | 930 | 10.64645253 | 0 |
|  | happiness - sadness | 1 | 1.371541667 | 9.554426671 | 930 | 0.143550389 | 0.999904513 |
|  | neutral - sadness | 1 | -100.3492083 | 9.554426671 | 930 | -10.50290214 | 0 |
|  | anger - fear | 2 | 69.05035417 | 9.554426671 | 930 | 7.227053652 | 1.00E-11 |
|  | anger - happiness | 2 | 139.5265 | 9.554426671 | 930 | 14.60333569 | 0 |
|  | anger - neutral | 2 | 213.6071458 | 9.554426671 | 930 | 22.35687741 | 0 |
|  | anger - sadness | 2 | 79.70895833 | 9.554426671 | 930 | 8.342620764 | 0 |
|  | fear - happiness | 2 | 70.47614583 | 9.554426671 | 930 | 7.376282038 | 3.36E-12 |
|  | fear - neutral | 2 | 144.5567917 | 9.554426671 | 930 | 15.12982376 | 0 |
|  | fear - sadness | 2 | 10.65860417 | 9.554426671 | 930 | 1.115567112 | 0.798371782 |
|  | happiness - neutral | 2 | 74.08064583 | 9.554426671 | 930 | 7.753541723 | 2.22E-15 |
|  | happiness - sadness | 2 | -59.81754167 | 9.554426671 | 930 | -6.260714926 | 5.84E-09 |
|  | neutral - sadness | 2 | -133.8981875 | 9.554426671 | 930 | -14.01425665 | 0 |
|  | anger - fear | 3 | 45.36052083 | 9.554426671 | 930 | 4.747592126 | 2.35E-05 |
|  | anger - happiness | 3 | 92.96045833 | 9.554426671 | 930 | 9.729569501 | 0 |
|  | anger - neutral | 3 | 176.7904583 | 9.554426671 | 930 | 18.50351302 | 0 |
|  | anger - sadness | 3 | 33.42329167 | 9.554426671 | 930 | 3.498199611 | 0.004460161 |
|  | fear - happiness | 3 | 47.5999375 | 9.554426671 | 930 | 4.981977374 | 7.43E-06 |
|  | fear - neutral | 3 | 131.4299375 | 9.554426671 | 930 | 13.7559209 | 0 |
|  | fear - sadness | 3 | -11.93722917 | 9.554426671 | 930 | -1.249392515 | 0.722245079 |
|  | happiness - neutral | 3 | 83.83 | 9.554426671 | 930 | 8.773943523 | 0 |
|  | happiness - sadness | 3 | -59.53716667 | 9.554426671 | 930 | -6.231369889 | 6.99E-09 |
|  | neutral - sadness | 3 | -143.3671667 | 9.554426671 | 930 | -15.00531341 | 0 |
|  | anger - fear | 4 | 74.30045833 | 9.554426671 | 930 | 7.776548075 | 0 |
|  | anger - happiness | 4 | 76.9705625 | 9.554426671 | 930 | 8.056010596 | 0 |
|  | anger - neutral | 4 | 193.8001875 | 9.554426671 | 930 | 20.28381128 | 0 |
|  | anger - sadness | 4 | 79.99091667 | 9.554426671 | 930 | 8.372131518 | 0 |
|  | fear - happiness | 4 | 2.670104167 | 9.554426671 | 930 | 0.279462521 | 0.998664751 |
|  | fear - neutral | 4 | 119.4997292 | 9.554426671 | 930 | 12.50726321 | 0 |
|  | fear - sadness | 4 | 5.690458333 | 9.554426671 | 930 | 0.595583443 | 0.975753691 |
|  | happiness - neutral | 4 | 116.829625 | 9.554426671 | 930 | 12.22780069 | 0 |
|  | happiness - sadness | 4 | 3.020354167 | 9.554426671 | 930 | 0.316120922 | 0.997835953 |
|  | neutral - sadness | 4 | -113.8092708 | 9.554426671 | 930 | -11.91167976 | 0 |

1. F0 Range

| **Main effects** |  | Chisq | Df | Pr(>Chisq) |  |  |  |
| --- | --- | --- | --- | --- | --- | --- | --- |
|  | Tone | 779.2258864 | 3 | 0.00000 |  |  |  |
|  | Emotion | 123.0198853 | 4 | 0.00000 |  |  |  |
|  | Tone:Emotion | 114.6370162 | 12 | 0.00000 |  |  |  |
| **Interaction** |  |  |  |  |  |  |  |
|  | contrast | Tone | estimate | SE | df | t.ratio | p.value |
|  | anger - fear | 1 | 15.93520833 | 7.932800997 | 930 | 2.008774497 | 0.262486503 |
|  | anger - happiness | 1 | -8.366020833 | 7.932800997 | 930 | -1.054611207 | 0.829587517 |
|  | anger - neutral | 1 | 25.60189583 | 7.932800997 | 930 | 3.227346286 | 0.011294893 |
|  | anger - sadness | 1 | 17.22775 | 7.932800997 | 930 | 2.171710851 | 0.19137989 |
|  | fear - happiness | 1 | -24.30122917 | 7.932800997 | 930 | -3.063385704 | 0.019072907 |
|  | fear - neutral | 1 | 9.6666875 | 7.932800997 | 930 | 1.218571789 | 0.740580998 |
|  | fear - sadness | 1 | 1.292541667 | 7.932800997 | 930 | 0.162936353 | 0.999841951 |
|  | happiness - neutral | 1 | 33.96791667 | 7.932800997 | 930 | 4.281957493 | 0.000198207 |
|  | happiness - sadness | 1 | 25.59377083 | 7.932800997 | 930 | 3.226322057 | 0.011332955 |
|  | neutral - sadness | 1 | -8.374145833 | 7.932800997 | 930 | -1.055635435 | 0.829083392 |
|  | anger - fear | 2 | 34.517875 | 7.932800997 | 930 | 4.351284624 | 0.000146161 |
|  | anger - happiness | 2 | 11.20122917 | 7.932800997 | 930 | 1.412014391 | 0.620045961 |
|  | anger - neutral | 2 | 45.40227083 | 7.932800997 | 930 | 5.72335936 | 1.40E-07 |
|  | anger - sadness | 2 | 27.35025 | 7.932800997 | 930 | 3.447741852 | 0.005334477 |
|  | fear - happiness | 2 | -23.31664583 | 7.932800997 | 930 | -2.939270233 | 0.027799569 |
|  | fear - neutral | 2 | 10.88439583 | 7.932800997 | 930 | 1.372074736 | 0.645780222 |
|  | fear - sadness | 2 | -7.167625 | 7.932800997 | 930 | -0.903542772 | 0.895573809 |
|  | happiness - neutral | 2 | 34.20104167 | 7.932800997 | 930 | 4.311344969 | 0.000174296 |
|  | happiness - sadness | 2 | 16.14902083 | 7.932800997 | 930 | 2.035727461 | 0.249709305 |
|  | neutral - sadness | 2 | -18.05202083 | 7.932800997 | 930 | -2.275617508 | 0.153716477 |
|  | anger - fear | 3 | 23.97283333 | 7.932800997 | 930 | 3.021988494 | 0.021668279 |
|  | anger - happiness | 3 | 7.2891875 | 7.932800997 | 930 | 0.918866804 | 0.889670238 |
|  | anger - neutral | 3 | 67.259625 | 7.932800997 | 930 | 8.478672921 | 0 |
|  | anger - sadness | 3 | 5.099479167 | 7.932800997 | 930 | 0.642834627 | 0.967959197 |
|  | fear - happiness | 3 | -16.68364583 | 7.932800997 | 930 | -2.103121689 | 0.219513378 |
|  | fear - neutral | 3 | 43.28679167 | 7.932800997 | 930 | 5.456684428 | 6.19E-07 |
|  | fear - sadness | 3 | -18.87335417 | 7.932800997 | 930 | -2.379153867 | 0.121910162 |
|  | happiness - neutral | 3 | 59.9704375 | 7.932800997 | 930 | 7.559806117 | 7.26E-13 |
|  | happiness - sadness | 3 | -2.189708333 | 7.932800997 | 930 | -0.276032178 | 0.998727984 |
|  | neutral - sadness | 3 | -62.16014583 | 7.932800997 | 930 | -7.835838295 | 0 |
|  | anger - fear | 4 | 57.82535417 | 7.932800997 | 930 | 7.289399317 | 6.41E-12 |
|  | anger - happiness | 4 | 24.79854167 | 7.932800997 | 930 | 3.12607636 | 0.015665173 |
|  | anger - neutral | 4 | 6.301770833 | 7.932800997 | 930 | 0.794394166 | 0.932260329 |
|  | anger - sadness | 4 | 42.80597917 | 7.932800997 | 930 | 5.396073743 | 8.60E-07 |
|  | fear - happiness | 4 | -33.0268125 | 7.932800997 | 930 | -4.163322957 | 0.000330227 |
|  | fear - neutral | 4 | -51.52358333 | 7.932800997 | 930 | -6.495005151 | 1.35E-09 |
|  | fear - sadness | 4 | -15.019375 | 7.932800997 | 930 | -1.893325574 | 0.321579078 |
|  | happiness - neutral | 4 | -18.49677083 | 7.932800997 | 930 | -2.331682194 | 0.135806549 |
|  | happiness - sadness | 4 | 18.0074375 | 7.932800997 | 930 | 2.269997383 | 0.155603514 |
|  | neutral - sadness | 4 | 36.50420833 | 7.932800997 | 930 | 4.601679577 | 4.68E-05 |

1. Mean Amplitude

| **Main effects** |  | Chisq | Df | Pr(>Chisq) |  |  |  |
| --- | --- | --- | --- | --- | --- | --- | --- |
|  | Tone | 121.1045977 | 3 | 0.00000 |  |  |  |
|  | Emotion | 1801.441001 | 4 | 0.00000 |  |  |  |
|  | Tone:Emotion | 3.920457435 | 12 | 0.98483 |  |  |  |
| **Interaction** |  |  |  |  |  |  |  |
|  | contrast | Tone | estimate | SE | df | t.ratio | p.value |
|  | anger - fear | 1 | 12.51227083 | 0.969663287 | 930 | 12.90372752 | 0 |
|  | anger - happiness | 1 | 11.94595833 | 0.969663287 | 930 | 12.31969746 | 0 |
|  | anger - neutral | 1 | 20.0934375 | 0.969663287 | 930 | 20.72207722 | 0 |
|  | anger - sadness | 1 | 9.913458333 | 0.969663287 | 930 | 10.22360903 | 0 |
|  | fear - happiness | 1 | -0.5663125 | 0.969663287 | 930 | -0.584030052 | 0.977441106 |
|  | fear - neutral | 1 | 7.581166667 | 0.969663287 | 930 | 7.818349701 | 0 |
|  | fear - sadness | 1 | -2.5988125 | 0.969663287 | 930 | -2.680118486 | 0.057687117 |
|  | happiness - neutral | 1 | 8.147479167 | 0.969663287 | 930 | 8.402379753 | 0 |
|  | happiness - sadness | 1 | -2.0325 | 0.969663287 | 930 | -2.096088434 | 0.222546652 |
|  | neutral - sadness | 1 | -10.17997917 | 0.969663287 | 930 | -10.49846819 | 0 |
|  | anger - fear | 2 | 12.876375 | 0.969663287 | 930 | 13.27922298 | 0 |
|  | anger - happiness | 2 | 11.85304167 | 0.969663287 | 930 | 12.22387382 | 0 |
|  | anger - neutral | 2 | 20.27504167 | 0.969663287 | 930 | 20.90936302 | 0 |
|  | anger - sadness | 2 | 9.760958333 | 0.969663287 | 930 | 10.06633794 | 0 |
|  | fear - happiness | 2 | -1.023333333 | 0.969663287 | 930 | -1.055349158 | 0.82922437 |
|  | fear - neutral | 2 | 7.398666667 | 0.969663287 | 930 | 7.630140038 | 3.41E-13 |
|  | fear - sadness | 2 | -3.115416667 | 0.969663287 | 930 | -3.212885039 | 0.011843088 |
|  | happiness - neutral | 2 | 8.422 | 0.969663287 | 930 | 8.685489197 | 0 |
|  | happiness - sadness | 2 | -2.092083333 | 0.969663287 | 930 | -2.157535881 | 0.196979026 |
|  | neutral - sadness | 2 | -10.51408333 | 0.969663287 | 930 | -10.84302508 | 0 |
|  | anger - fear | 3 | 12.99185417 | 0.969663287 | 930 | 13.39831501 | 0 |
|  | anger - happiness | 3 | 10.72275 | 0.969663287 | 930 | 11.05822005 | 0 |
|  | anger - neutral | 3 | 20.73122917 | 0.969663287 | 930 | 21.37982272 | 0 |
|  | anger - sadness | 3 | 8.884583333 | 0.969663287 | 930 | 9.16254483 | 0 |
|  | fear - happiness | 3 | -2.269104167 | 0.969663287 | 930 | -2.340094957 | 0.133260394 |
|  | fear - neutral | 3 | 7.739375 | 0.969663287 | 930 | 7.981507712 | 0 |
|  | fear - sadness | 3 | -4.107270833 | 0.969663287 | 930 | -4.235770179 | 0.000242177 |
|  | happiness - neutral | 3 | 10.00847917 | 0.969663287 | 930 | 10.32160267 | 0 |
|  | happiness - sadness | 3 | -1.838166667 | 0.969663287 | 930 | -1.895675223 | 0.32030862 |
|  | neutral - sadness | 3 | -11.84664583 | 0.969663287 | 930 | -12.21727789 | 0 |
|  | anger - fear | 4 | 12.61885417 | 0.969663287 | 930 | 13.0136454 | 0 |
|  | anger - happiness | 4 | 10.8819375 | 0.969663287 | 930 | 11.22238786 | 0 |
|  | anger - neutral | 4 | 19.87195833 | 0.969663287 | 930 | 20.49366889 | 0 |
|  | anger - sadness | 4 | 9.245791667 | 0.969663287 | 930 | 9.535053863 | 0 |
|  | fear - happiness | 4 | -1.736916667 | 0.969663287 | 930 | -1.791257533 | 0.379243495 |
|  | fear - neutral | 4 | 7.253104167 | 0.969663287 | 930 | 7.480023496 | 1.48E-12 |
|  | fear - sadness | 4 | -3.3730625 | 0.969663287 | 930 | -3.478591534 | 0.004782987 |
|  | happiness - neutral | 4 | 8.990020833 | 0.969663287 | 930 | 9.271281029 | 0 |
|  | happiness - sadness | 4 | -1.636145833 | 0.969663287 | 930 | -1.687334001 | 0.442276931 |
|  | neutral - sadness | 4 | -10.62616667 | 0.969663287 | 930 | -10.95861503 | 0 |

1. Duration

| **Main effects** |  | Chisq | Df | Pr(>Chisq) |  |  |  |
| --- | --- | --- | --- | --- | --- | --- | --- |
|  | Tone | 32.50337477 | 3 | 0.00000 |  |  |  |
|  | Emotion | 639.7428496 | 4 | 0.00000 |  |  |  |
|  | Tone:Emotion | 14.20415941 | 12 | 0.28786 |  |  |  |
| **Interaction** |  |  |  |  |  |  |  |
|  | contrast | Tone | estimate | SE | df | t.ratio | p.value |
|  | anger - fear | 1 | -0.005895833 | 0.008136049 | 930 | -0.724655573 | 0.950812242 |
|  | anger - happiness | 1 | -0.024291667 | 0.008136049 | 930 | -2.985683387 | 0.024195314 |
|  | anger - neutral | 1 | -0.017895833 | 0.008136049 | 930 | -2.199572924 | 0.180699431 |
|  | anger - sadness | 1 | -0.077395833 | 0.008136049 | 930 | -9.512704787 | 0 |
|  | fear - happiness | 1 | -0.018395833 | 0.008136049 | 930 | -2.261027813 | 0.15865026 |
|  | fear - neutral | 1 | -0.012 | 0.008136049 | 930 | -1.474917351 | 0.579103528 |
|  | fear - sadness | 1 | -0.0715 | 0.008136049 | 930 | -8.788049214 | 0 |
|  | happiness - neutral | 1 | 0.006395833 | 0.008136049 | 930 | 0.786110463 | 0.934660802 |
|  | happiness - sadness | 1 | -0.053104167 | 0.008136049 | 930 | -6.5270214 | 1.10E-09 |
|  | neutral - sadness | 1 | -0.0595 | 0.008136049 | 930 | -7.313131863 | 5.39E-12 |
|  | anger - fear | 2 | -0.014708333 | 0.008136049 | 930 | -1.807798003 | 0.369586362 |
|  | anger - happiness | 2 | -0.028770833 | 0.008136049 | 930 | -3.536216773 | 0.003890547 |
|  | anger - neutral | 2 | -0.026541667 | 0.008136049 | 930 | -3.26223039 | 0.010065272 |
|  | anger - sadness | 2 | -0.099479167 | 0.008136049 | 930 | -12.22696241 | 0 |
|  | fear - happiness | 2 | -0.0140625 | 0.008136049 | 930 | -1.72841877 | 0.416907957 |
|  | fear - neutral | 2 | -0.011833333 | 0.008136049 | 930 | -1.454432387 | 0.5924714 |
|  | fear - sadness | 2 | -0.084770833 | 0.008136049 | 930 | -10.41916441 | 0 |
|  | happiness - neutral | 2 | 0.002229167 | 0.008136049 | 930 | 0.273986383 | 0.998764629 |
|  | happiness - sadness | 2 | -0.070708333 | 0.008136049 | 930 | -8.690745638 | 0 |
|  | neutral - sadness | 2 | -0.0729375 | 0.008136049 | 930 | -8.964732021 | 0 |
|  | anger - fear | 3 | -0.0155625 | 0.008136049 | 930 | -1.912783439 | 0.31114026 |
|  | anger - happiness | 3 | -0.023354167 | 0.008136049 | 930 | -2.870455469 | 0.03400164 |
|  | anger - neutral | 3 | -0.010604167 | 0.008136049 | 930 | -1.303355784 | 0.689207691 |
|  | anger - sadness | 3 | -0.096791667 | 0.008136049 | 930 | -11.89664238 | 0 |
|  | fear - happiness | 3 | -0.007791667 | 0.008136049 | 930 | -0.95767203 | 0.873903974 |
|  | fear - neutral | 3 | 0.004958333 | 0.008136049 | 930 | 0.609427655 | 0.973620876 |
|  | fear - sadness | 3 | -0.081229167 | 0.008136049 | 930 | -9.983858941 | 0 |
|  | happiness - neutral | 3 | 0.01275 | 0.008136049 | 930 | 1.567099685 | 0.518991788 |
|  | happiness - sadness | 3 | -0.0734375 | 0.008136049 | 930 | -9.026186911 | 0 |
|  | neutral - sadness | 3 | -0.0861875 | 0.008136049 | 930 | -10.5932866 | 0 |
|  | anger - fear | 4 | -0.005479167 | 0.008136049 | 930 | -0.673443165 | 0.962105592 |
|  | anger - happiness | 4 | -0.0395 | 0.008136049 | 930 | -4.854936279 | 1.39E-05 |
|  | anger - neutral | 4 | -0.018375 | 0.008136049 | 930 | -2.258467193 | 0.15952798 |
|  | anger - sadness | 4 | -0.095270833 | 0.008136049 | 930 | -11.70971709 | 0 |
|  | fear - happiness | 4 | -0.034020833 | 0.008136049 | 930 | -4.181493114 | 0.000305663 |
|  | fear - neutral | 4 | -0.012895833 | 0.008136049 | 930 | -1.585024028 | 0.507382304 |
|  | fear - sadness | 4 | -0.089791667 | 0.008136049 | 930 | -11.03627393 | 0 |
|  | happiness - neutral | 4 | 0.021125 | 0.008136049 | 930 | 2.596469086 | 0.071802915 |
|  | happiness - sadness | 4 | -0.055770833 | 0.008136049 | 930 | -6.854780811 | 1.30E-10 |
|  | neutral - sadness | 4 | -0.076895833 | 0.008136049 | 930 | -9.451249897 | 0 |
