# Supplementary material for: Emotional tones of voice affect the acoustics and perception of Mandarin tones
Source: PLoS One. 2023 Apr 5;18(4):e0283635. doi: 10.1371/journal.pone.0283635 (PMC10075469; doi:10.1371/journal.pone.0283635)
Supplement: S2 Table — (DOCX) [file pone.0283635.s002.docx]

| **Main effects** |  | Chisq | Df | Pr(>Chisq) |  |  |
| --- | --- | --- | --- | --- | --- | --- |
|  | Tone | 83.117382 | 3 | 0.00000 |  |  |
|  | Emotion | 486.3770095 | 4 | 0.00000 |  |  |
|  | Context | 1534.706301 | 1 | 0.00000 |  |  |
|  | Tone:Emotion | 492.8922021 | 12 | 0.00000 |  |  |
| **Interaction** |  |  |  |  |  |  |
| **tone*emotion** | contrast | Emotion | estimate | SE | z.ratio | p.value |
|  | Tone1 - Tone2 | 1 | 0.84349404 | 0.133124709 | 6.336119326 | 0.00000 |
|  | Tone1 - Tone3 | 1 | -0.26857053 | 0.120379636 | -2.231029591 | 0.11489 |
|  | Tone1 - Tone4 | 1 | 1.68134013 | 0.156297644 | 10.75729672 | 0.00000 |
|  | Tone2 - Tone3 | 1 | -1.11206457 | 0.131674767 | -8.445540433 | 0.00000 |
|  | Tone2 - Tone4 | 1 | 0.837846091 | 0.164710672 | 5.086774785 | 0.00000 |
|  | Tone3 - Tone4 | 1 | 1.949910661 | 0.155138703 | 12.56882143 | 0.00000 |
|  | Tone1 - Tone2 | 2 | -1.390457515 | 0.15274502 | -9.103128309 | 0.00000 |
|  | Tone1 - Tone3 | 2 | -1.910852463 | 0.150087619 | -12.73157957 | 0.00000 |
|  | Tone1 - Tone4 | 2 | -1.032268154 | 0.156020821 | -6.616220511 | 0.00000 |
|  | Tone2 - Tone3 | 2 | -0.520394948 | 0.121477857 | -4.28386672 | 0.00011 |
|  | Tone2 - Tone4 | 2 | 0.358189361 | 0.129215341 | 2.772034318 | 0.02850 |
|  | Tone3 - Tone4 | 2 | 0.878584309 | 0.125846481 | 6.981397488 | 0.00000 |
|  | Tone1 - Tone2 | 3 | 1.480726373 | 0.145484556 | 10.17789385 | 0.00000 |
|  | Tone1 - Tone3 | 3 | 0.671108208 | 0.127845677 | 5.249361746 | 0.00000 |
|  | Tone1 - Tone4 | 3 | 0.030886122 | 0.120888803 | 0.255492001 | 0.99415 |
|  | Tone2 - Tone3 | 3 | -0.809618165 | 0.151148911 | -5.356427392 | 0.00000 |
|  | Tone2 - Tone4 | 3 | -1.449840251 | 0.145669207 | -9.952963144 | 0.00000 |
|  | Tone3 - Tone4 | 3 | -0.640222086 | 0.12806689 | -4.999122625 | 0.00000 |
|  | Tone1 - Tone2 | 4 | -0.867787303 | 0.316652597 | -2.740502722 | 0.03120 |
|  | Tone1 - Tone3 | 4 | -0.801940209 | 0.319502818 | -2.509962866 | 0.05837 |
|  | Tone1 - Tone4 | 4 | 0.418101796 | 0.412366616 | 1.013907962 | 0.74129 |
|  | Tone2 - Tone3 | 4 | 0.065847094 | 0.255887022 | 0.257328776 | 0.99403 |
|  | Tone2 - Tone4 | 4 | 1.285889099 | 0.365551365 | 3.517670081 | 0.00246 |
|  | Tone3 - Tone4 | 4 | 1.220042005 | 0.36802619 | 3.315095607 | 0.00507 |
|  | Tone1 - Tone2 | 5 | -0.871397742 | 0.147212982 | -5.919299587 | 0.00000 |
|  | Tone1 - Tone3 | 5 | -1.005342064 | 0.145902743 | -6.890494587 | 0.00000 |
|  | Tone1 - Tone4 | 5 | -1.117745792 | 0.144949495 | -7.711277566 | 0.00000 |
|  | Tone2 - Tone3 | 5 | -0.133944322 | 0.129202561 | -1.036700209 | 0.72785 |
|  | Tone2 - Tone4 | 5 | -0.24634805 | 0.128089 | -1.923256883 | 0.21819 |
|  | Tone3 - Tone4 | 5 | -0.112403728 | 0.126521089 | -0.888418913 | 0.81090 |
