# Supplementary material for: Emotional tones of voice affect the acoustics and perception of Mandarin tones
Source: PLoS One. 2023 Apr 5;18(4):e0283635. doi: 10.1371/journal.pone.0283635 (PMC10075469; doi:10.1371/journal.pone.0283635)
Supplement: S3 Table — (DOCX) [file pone.0283635.s003.docx]

| **Main effects** |  | Chisq | Df | Pr(>Chisq) |  |  |
| --- | --- | --- | --- | --- | --- | --- |
|  | Tone | 92.9186782 | 3 | 0.00000 |  |  |
|  | Emotion | 775.0807423 | 3 | 0.00000 |  |  |
|  | Context | 1843.41991 | 1 | 0.00000 |  |  |
|  | Tone:Emotion | 327.9450391 | 9 | 0.00000 |  |  |
| **Interaction** |  |  |  |  |  |  |
| **tone*emotion** | contrast | Tone | estimate | SE | z.ratio | p.value |
|  | anger - fear | 1 | -2.235020417 | 0.13626027 | -16.40258315 | 0.00000 |
|  | anger - happiness | 1 | 0.247366567 | 0.157452528 | 1.571054914 | 0.39525 |
|  | anger - sadness | 1 | -1.661795262 | 0.136188467 | -12.20217321 | 0.00000 |
|  | fear - happiness | 1 | 2.482386984 | 0.142788343 | 17.38508152 | 0.00000 |
|  | fear - sadness | 1 | 0.573225155 | 0.116588339 | 4.916659403 | 0.00001 |
|  | happiness - sadness | 1 | -1.909161829 | 0.14268399 | -13.38035073 | 0.00000 |
|  | anger - fear | 2 | -1.73710438 | 0.135270662 | -12.84169353 | 0.00000 |
|  | anger - happiness | 2 | -0.01128649 | 0.150038333 | -0.075224041 | 0.99985 |
|  | anger - sadness | 2 | -0.426745283 | 0.143213484 | -2.979784248 | 0.01529 |
|  | fear - happiness | 2 | 1.72581789 | 0.135011796 | 12.78271927 | 0.00000 |
|  | fear - sadness | 2 | 1.310359097 | 0.127146703 | 10.30588343 | 0.00000 |
|  | happiness - sadness | 2 | -0.415458793 | 0.142972961 | -2.905855696 | 0.01919 |
|  | anger - fear | 3 | -0.916046115 | 0.121096207 | -7.564614434 | 0.00000 |
|  | anger - happiness | 3 | -0.160877439 | 0.123752832 | -1.299989961 | 0.56290 |
|  | anger - sadness | 3 | 0.632158249 | 0.132772102 | 4.761227979 | 0.00001 |
|  | fear - happiness | 3 | 0.755168676 | 0.119735442 | 6.306976978 | 0.00000 |
|  | fear - sadness | 3 | 1.548204364 | 0.129447125 | 11.96012939 | 0.00000 |
|  | happiness - sadness | 3 | 0.793035688 | 0.1316845 | 6.022240168 | 0.00000 |
|  | anger - fear | 4 | -3.091700241 | 0.191190336 | -16.17079763 | 0.00000 |
|  | anger - happiness | 4 | -1.693960384 | 0.19761772 | -8.5719053 | 0.00000 |
|  | anger - sadness | 4 | -2.166361615 | 0.19380647 | -11.17796336 | 0.00000 |
|  | fear - happiness | 4 | 1.397739857 | 0.128681222 | 10.86203436 | 0.00000 |
|  | fear - sadness | 4 | 0.925338625 | 0.122298728 | 7.566216273 | 0.00000 |
|  | happiness - sadness | 4 | -0.472401231 | 0.132892113 | -3.554772515 | 0.00214 |
