# Supplementary material for: Emotional tones of voice affect the acoustics and perception of Mandarin tones
Source: PLoS One. 2023 Apr 5;18(4):e0283635. doi: 10.1371/journal.pone.0283635 (PMC10075469; doi:10.1371/journal.pone.0283635)
Supplement: S4 Table — (RTF) [file pone.0283635.s004.rtf]

Experiment 1
No. of Talker	Talker Gender	Emotion	Tone	Syllable	Repetition	mean F0	F0 range	intensity	duration	
1	Female	Anger	Tone 1	/ci/	1	502.25	26.53	66.826	0.186	
1	Female	Anger	Tone 1	/ci/	2	513.265	37.164	69.449	0.184	
1	Female	Anger	Tone 2	/ci/	1	427.585	63.315	65.51	0.191	
1	Female	Anger	Tone 2	/ci/	2	415.663	69.341	66.179	0.181	
1	Female	Anger	Tone 3	/ci/	1	352.333	178.949	64.194	0.211	
1	Female	Anger	Tone 3	/ci/	2	359.442	153.692	61.818	0.199	
1	Female	Anger	Tone 4	/ci/	1	489.951	179.314	68.295	0.193	
1	Female	Anger	Tone 4	/ci/	2	514.123	187.273	70.105	0.216	
1	Female	Anger	Tone 1	/fa/	1	479.108	30.017	77.441	0.211	
1	Female	Anger	Tone 1	/fa/	2	484.746	56.215	77.486	0.198	
1	Female	Anger	Tone 2	/fa/	1	361.137	96.559	75.321	0.212	
1	Female	Anger	Tone 2	/fa/	2	409.442	74.131	76.663	0.216	
1	Female	Anger	Tone 3	/fa/	1	382.998	162.744	75.558	0.229	
1	Female	Anger	Tone 3	/fa/	2	339.178	168.502	74.242	0.218	
1	Female	Anger	Tone 4	/fa/	1	486.668	132.038	76.752	0.204	
1	Female	Anger	Tone 4	/fa/	2	479.783	128.556	74.128	0.258	
1	Female	Anger	Tone 1	/pu/	1	522.386	98.387	70.086	0.22	
1	Female	Anger	Tone 1	/pu/	2	519.069	79.83	69.929	0.237	
1	Female	Anger	Tone 2	/pu/	1	441.319	76.457	66.596	0.245	
1	Female	Anger	Tone 2	/pu/	2	447.099	76.804	67.581	0.232	
1	Female	Anger	Tone 3	/pu/	1	397.774	198.011	66.66	0.213	
1	Female	Anger	Tone 3	/pu/	2	400.05	216.419	67.069	0.237	
1	Female	Anger	Tone 4	/pu/	1	485.021	201.999	72.004	0.205	
1	Female	Anger	Tone 4	/pu/	2	498.036	199.198	71.118	0.222	
1	Female	Fear	Tone 1	/ci/	1	399.984	40.588	60.461	0.192	
1	Female	Fear	Tone 1	/ci/	2	435.442	48.861	63.634	0.219	
1	Female	Fear	Tone 2	/ci/	1	418.471	78.449	62.067	0.201	
1	Female	Fear	Tone 2	/ci/	2	417.896	74.964	61.02	0.184	
1	Female	Fear	Tone 3	/ci/	1	355.685	113.488	60.194	0.187	
1	Female	Fear	Tone 3	/ci/	2	377.342	132.24	61.74	0.182	
1	Female	Fear	Tone 4	/ci/	1	445.084	87.889	62.247	0.191	
1	Female	Fear	Tone 4	/ci/	2	447.796	109.195	61.946	0.171	
1	Female	Fear	Tone 1	/fa/	1	446.596	57.695	66.231	0.208	
1	Female	Fear	Tone 1	/fa/	2	452.283	58.039	70.713	0.222	
1	Female	Fear	Tone 2	/fa/	1	406.697	90.41	68.351	0.203	
1	Female	Fear	Tone 2	/fa/	2	422.093	88.29	71.508	0.223	
1	Female	Fear	Tone 3	/fa/	1	368.093	200.47	66.792	0.236	
1	Female	Fear	Tone 3	/fa/	2	368.841	162.56	65.554	0.222	
1	Female	Fear	Tone 4	/fa/	1	459.918	125.551	72.897	0.21	
1	Female	Fear	Tone 4	/fa/	2	469.074	142.199	73.729	0.24	
1	Female	Fear	Tone 1	/pu/	1	459.077	57.357	62.646	0.221	
1	Female	Fear	Tone 1	/pu/	2	444.916	70.568	58.477	0.179	
1	Female	Fear	Tone 2	/pu/	1	405.155	136.857	63.571	0.185	
1	Female	Fear	Tone 2	/pu/	2	400.315	120.692	61.716	0.175	
1	Female	Fear	Tone 3	/pu/	1	367.162	197.339	61.103	0.215	

1	Female	Fear	Tone 3	/pu/	2	323.142	132.393	58.519	0.155	
1	Female	Fear	Tone 4	/pu/	1	421.465	113.438	60.608	0.175	
1	Female	Fear	Tone 4	/pu/	2	423.99	131.383	60.709	0.185	
1	Female	appines	Tone 1	/ci/	1	426.135	35.333	62.266	0.19	
1	Female	appines	Tone 1	/ci/	2	441.332	63.247	61.971	0.188	
1	Female	appines	Tone 2	/ci/	1	273.206	87.437	53.357	0.24	
1	Female	appines	Tone 2	/ci/	2	279.143	71.515	55.251	0.2	
1	Female	appines	Tone 3	/ci/	1	244.489	132.996	53.66	0.231	
1	Female	appines	Tone 3	/ci/	2	230.325	104.929	51.758	0.22	
1	Female	appines	Tone 4	/ci/	1	429.257	223.48	60.95	0.209	
1	Female	appines	Tone 4	/ci/	2	434.897	192.129	62.13	0.218	
1	Female	appines	Tone 1	/fa/	1	434.383	81.598	62.531	0.244	
1	Female	appines	Tone 1	/fa/	2	437.678	83.7	64.442	0.241	
1	Female	appines	Tone 2	/fa/	1	274.059	108.247	68.238	0.25	
1	Female	appines	Tone 2	/fa/	2	267.007	119.93	65.71	0.25	
1	Female	appines	Tone 3	/fa/	1	226.386	219.333	64.466	0.23	
1	Female	appines	Tone 3	/fa/	2	239.759	164.106	63.781	0.226	
1	Female	appines	Tone 4	/fa/	1	386.15	229.861	62.604	0.271	
1	Female	appines	Tone 4	/fa/	2	371.976	248.707	64.18	0.266	
1	Female	appines	Tone 1	/pu/	1	421.246	88.395	59.027	0.253	
1	Female	appines	Tone 1	/pu/	2	422.67	42.983	59.27	0.183	
1	Female	appines	Tone 2	/pu/	1	274.352	143.407	58.289	0.216	
1	Female	appines	Tone 2	/pu/	2	272.708	124.969	60.532	0.215	
1	Female	appines	Tone 3	/pu/	1	250.988	180.099	55.421	0.246	
1	Female	appines	Tone 3	/pu/	2	253.406	194.346	56.908	0.23	
1	Female	appines	Tone 4	/pu/	1	423.483	141.173	59.991	0.236	
1	Female	appines	Tone 4	/pu/	2	426.554	162.957	61.09	0.216	
1	Female	Neutral	Tone 1	/ci/	1	319.792	29.158	50.076	0.201	
1	Female	Neutral	Tone 1	/ci/	2	311.001	25.081	49.735	0.178	
1	Female	Neutral	Tone 2	/ci/	1	239.549	27.631	50.301	0.194	
1	Female	Neutral	Tone 2	/ci/	2	230.741	31.894	48.662	0.189	
1	Female	Neutral	Tone 3	/ci/	1	177.563	59.099	48.604	0.167	
1	Female	Neutral	Tone 3	/ci/	2	176.547	57.188	45.696	0.193	
1	Female	Neutral	Tone 4	/ci/	1	280.604	212.479	49.148	0.209	
1	Female	Neutral	Tone 4	/ci/	2	299.027	189.56	47.967	0.176	
1	Female	Neutral	Tone 1	/fa/	1	299.856	43.969	55.189	0.228	
1	Female	Neutral	Tone 1	/fa/	2	274.461	39.362	54.511	0.22	
1	Female	Neutral	Tone 2	/fa/	1	226.696	62.199	52.964	0.25	
1	Female	Neutral	Tone 2	/fa/	2	216.893	62.877	51.764	0.225	
1	Female	Neutral	Tone 3	/fa/	1	174.713	91.24	48.589	0.197	
1	Female	Neutral	Tone 3	/fa/	2	172.98	90.594	47.745	0.21	
1	Female	Neutral	Tone 4	/fa/	1	279.479	205.453	52.299	0.23	
1	Female	Neutral	Tone 4	/fa/	2	277.603	158.413	53.194	0.206	
1	Female	Neutral	Tone 1	/pu/	1	316.684	55.947	50.135	0.165	
1	Female	Neutral	Tone 1	/pu/	2	316.102	49.541	50.502	0.176	
1	Female	Neutral	Tone 2	/pu/	1	231.851	49.783	49.296	0.2	
1	Female	Neutral	Tone 2	/pu/	2	222.754	42.083	49.693	0.19	

1	Female	Neutral	Tone 3	/pu/	1	180.722	59.611	47.105	0.19	
1	Female	Neutral	Tone 3	/pu/	2	175.983	78.726	45.244	0.21	
1	Female	Neutral	Tone 4	/pu/	1	299.683	205.85	49.868	0.154	
1	Female	Neutral	Tone 4	/pu/	2	239.177	140.655	47.178	0.186	
1	Female	Sadness	Tone 1	/ci/	1	315.876	17.006	49.542	0.185	
1	Female	Sadness	Tone 1	/ci/	2	318.129	36.507	48.66	0.193	
1	Female	Sadness	Tone 2	/ci/	1	264.451	62.965	46.338	0.23	
1	Female	Sadness	Tone 2	/ci/	2	252.73	47.225	44.712	0.23	
1	Female	Sadness	Tone 3	/ci/	1	244.588	117.813	46.478	0.176	
1	Female	Sadness	Tone 3	/ci/	2	246.758	105.141	45.974	0.18	
1	Female	Sadness	Tone 4	/ci/	1	315.18	117.839	50.085	0.188	
1	Female	Sadness	Tone 4	/ci/	2	337.162	118.711	50.658	0.193	
1	Female	Sadness	Tone 1	/fa/	1	296.697	44.954	51.659	0.225	
1	Female	Sadness	Tone 1	/fa/	2	297.932	43.505	48.953	0.253	
1	Female	Sadness	Tone 2	/fa/	1	269.479	101.271	53.792	0.28	
1	Female	Sadness	Tone 2	/fa/	2	265.62	64.884	54.907	0.27	
1	Female	Sadness	Tone 3	/fa/	1	235.313	131.072	48.139	0.239	
1	Female	Sadness	Tone 3	/fa/	2	236.456	132.107	51.008	0.254	
1	Female	Sadness	Tone 4	/fa/	1	282.723	109.383	55.616	0.257	
1	Female	Sadness	Tone 4	/fa/	2	295.344	117.155	55.427	0.28	
1	Female	Sadness	Tone 1	/pu/	1	313.496	64.728	47.636	0.177	
1	Female	Sadness	Tone 1	/pu/	2	309.121	71.134	44.966	0.201	
1	Female	Sadness	Tone 2	/pu/	1	259.292	58.435	47.309	0.282	
1	Female	Sadness	Tone 2	/pu/	2	277.3	71.911	46.671	0.24	
1	Female	Sadness	Tone 3	/pu/	1	242.067	120.911	44.53	0.243	
1	Female	Sadness	Tone 3	/pu/	2	242.613	124.31	43.804	0.211	
1	Female	Sadness	Tone 4	/pu/	1	291.705	130.857	49.621	0.275	
1	Female	Sadness	Tone 4	/pu/	2	297.25	135.838	49.391	0.254	
2	Female	Anger	Tone 1	/ci/	1	504.102	57.828	65.637	0.162	
2	Female	Anger	Tone 1	/ci/	2	510.891	58.064	64.497	0.153	
2	Female	Anger	Tone 2	/ci/	1	406.668	81.566	61.747	0.198	
2	Female	Anger	Tone 2	/ci/	2	429.03	66.163	61.848	0.213	
2	Female	Anger	Tone 3	/ci/	1	366.877	140.043	62.606	0.24	
2	Female	Anger	Tone 3	/ci/	2	385.11	147.247	63.033	0.229	
2	Female	Anger	Tone 4	/ci/	1	463.074	217.64	64.714	0.21	
2	Female	Anger	Tone 4	/ci/	2	496.002	205.11	67.271	0.202	
2	Female	Anger	Tone 1	/fa/	1	466.799	34.653	69.436	0.18	
2	Female	Anger	Tone 1	/fa/	2	435.836	59.119	66.919	0.19	
2	Female	Anger	Tone 2	/fa/	1	356.083	95.529	67.112	0.189	
2	Female	Anger	Tone 2	/fa/	2	400.121	100.182	69.557	0.177	
2	Female	Anger	Tone 3	/fa/	1	356.962	179.886	68.241	0.184	
2	Female	Anger	Tone 3	/fa/	2	351.429	176.258	70.147	0.188	
2	Female	Anger	Tone 4	/fa/	1	456.998	127.465	71.63	0.163	
2	Female	Anger	Tone 4	/fa/	2	462.541	137.573	72.755	0.182	
2	Female	Anger	Tone 1	/pu/	1	497.667	20.538	70.931	0.197	
2	Female	Anger	Tone 1	/pu/	2	512.76	28.111	67.553	0.22	
2	Female	Anger	Tone 2	/pu/	1	426.361	91.563	61.875	0.227	

2	Female	Anger	Tone 2	/pu/	2	436.697	80.419	63.602	0.231	
2	Female	Anger	Tone 3	/pu/	1	332.031	212.769	65.872	0.25	
2	Female	Anger	Tone 3	/pu/	2	412.633	188.19	66.214	0.189	
2	Female	Anger	Tone 4	/pu/	1	490.281	217.543	69.678	0.217	
2	Female	Anger	Tone 4	/pu/	2	484.047	156.709	68.238	0.196	
2	Female	Fear	Tone 1	/ci/	1	376.865	43.258	54.308	0.259	
2	Female	Fear	Tone 1	/ci/	2	359.105	32.964	53.946	0.206	
2	Female	Fear	Tone 2	/ci/	1	299.416	60.42	51.918	0.3	
2	Female	Fear	Tone 2	/ci/	2	317.8	62.234	53.806	0.264	
2	Female	Fear	Tone 3	/ci/	1	283.22	143.228	52.049	0.302	
2	Female	Fear	Tone 3	/ci/	2	289.24	192.803	54.295	0.287	
2	Female	Fear	Tone 4	/ci/	1	355.356	149.681	56.298	0.251	
2	Female	Fear	Tone 4	/ci/	2	358.661	111.691	53.518	0.237	
2	Female	Fear	Tone 1	/fa/	1	359.302	35.804	47.208	0.246	
2	Female	Fear	Tone 1	/fa/	2	364.809	28.574	47.694	0.236	
2	Female	Fear	Tone 2	/fa/	1	313.343	93.595	45.159	0.266	
2	Female	Fear	Tone 2	/fa/	2	300.56	51.968	48.306	0.275	
2	Female	Fear	Tone 3	/fa/	1	279.223	182.05	48.608	0.429	
2	Female	Fear	Tone 3	/fa/	2	288.281	165.97	49.794	0.382	
2	Female	Fear	Tone 4	/fa/	1	373.381	135.869	46.876	0.275	
2	Female	Fear	Tone 4	/fa/	2	380.607	113.729	52.808	0.336	
2	Female	Fear	Tone 1	/pu/	1	376.67	60.158	54.157	0.225	
2	Female	Fear	Tone 1	/pu/	2	361.138	47.599	54.053	0.276	
2	Female	Fear	Tone 2	/pu/	1	307.615	65.501	49.886	0.28	
2	Female	Fear	Tone 2	/pu/	2	311.348	66.074	48.884	0.306	
2	Female	Fear	Tone 3	/pu/	1	270.922	129.828	47.38	0.294	
2	Female	Fear	Tone 3	/pu/	2	269.54	148.96	49.434	0.256	
2	Female	Fear	Tone 4	/pu/	1	336.592	162.821	51.187	0.286	
2	Female	Fear	Tone 4	/pu/	2	355.63	154.979	49.546	0.205	
2	Female	appines	Tone 1	/ci/	1	430.987	37.823	57.073	0.17	
2	Female	appines	Tone 1	/ci/	2	426.553	74.103	57.376	0.179	
2	Female	appines	Tone 2	/ci/	1	254.832	58.177	52.242	0.185	
2	Female	appines	Tone 2	/ci/	2	263.705	57.192	53.559	0.2	
2	Female	appines	Tone 3	/ci/	1	258.139	105.068	55.365	0.161	
2	Female	appines	Tone 3	/ci/	2	193.182	98.135	50.912	0.221	
2	Female	appines	Tone 4	/ci/	1	447.363	187.948	62.613	0.245	
2	Female	appines	Tone 4	/ci/	2	420.265	134.598	62.051	0.211	
2	Female	appines	Tone 1	/fa/	1	371.769	41.172	57.981	0.194	
2	Female	appines	Tone 1	/fa/	2	376.26	60.791	58.659	0.216	
2	Female	appines	Tone 2	/fa/	1	193.841	70.003	58.086	0.15	
2	Female	appines	Tone 2	/fa/	2	219.196	52.451	60.207	0.133	
2	Female	appines	Tone 3	/fa/	1	273.082	180.564	62.12	0.182	
2	Female	appines	Tone 3	/fa/	2	231.106	189.859	60.168	0.236	
2	Female	appines	Tone 4	/fa/	1	403.319	147.439	60.101	0.225	
2	Female	appines	Tone 4	/fa/	2	410.973	107.22	63.493	0.236	
2	Female	appines	Tone 1	/pu/	1	463.848	83.678	62.515	0.245	
2	Female	appines	Tone 1	/pu/	2	442.827	88.208	61.135	0.251	

2	Female	appines	Tone 2	/pu/	1	312.716	81.855	55.357	0.18	
2	Female	appines	Tone 2	/pu/	2	326.571	68.86	55.956	0.196	
2	Female	appines	Tone 3	/pu/	1	300.384	146.086	58.52	0.165	
2	Female	appines	Tone 3	/pu/	2	271.821	181.472	60.363	0.226	
2	Female	appines	Tone 4	/pu/	1	490.69	181.273	64.678	0.214	
2	Female	appines	Tone 4	/pu/	2	472.127	197.134	64.572	0.215	
2	Female	Neutral	Tone 1	/ci/	1	319.988	31.766	51.722	0.271	
2	Female	Neutral	Tone 1	/ci/	2	317.282	32.216	51.99	0.257	
2	Female	Neutral	Tone 2	/ci/	1	196.682	66.97	47.15	0.304	
2	Female	Neutral	Tone 2	/ci/	2	200.249	52.515	47.31	0.29	
2	Female	Neutral	Tone 3	/ci/	1	169.375	71.288	48.739	0.317	
2	Female	Neutral	Tone 3	/ci/	2	170.581	89.555	49.235	0.321	
2	Female	Neutral	Tone 4	/ci/	1	269.29	213.956	51.13	0.274	
2	Female	Neutral	Tone 4	/ci/	2	270.686	203.295	52.456	0.284	
2	Female	Neutral	Tone 1	/fa/	1	327.099	33.792	54.007	0.265	
2	Female	Neutral	Tone 1	/fa/	2	320.872	45.755	55.602	0.281	
2	Female	Neutral	Tone 2	/fa/	1	197.894	45.259	51.215	0.31	
2	Female	Neutral	Tone 2	/fa/	2	194.194	51.769	50.595	0.286	
2	Female	Neutral	Tone 3	/fa/	1	173.318	138.428	50.568	0.36	
2	Female	Neutral	Tone 3	/fa/	2	173.396	137.704	51.2	0.36	
2	Female	Neutral	Tone 4	/fa/	1	275.668	223.908	53.042	0.286	
2	Female	Neutral	Tone 4	/fa/	2	272.413	233.323	53.46	0.317	
2	Female	Neutral	Tone 1	/pu/	1	339.953	38.332	48.819	0.25	
2	Female	Neutral	Tone 1	/pu/	2	338.072	41.044	49.578	0.254	
2	Female	Neutral	Tone 2	/pu/	1	208.909	86.02	47.746	0.311	
2	Female	Neutral	Tone 2	/pu/	2	201.985	62.328	49.76	0.321	
2	Female	Neutral	Tone 3	/pu/	1	164.717	147.511	49.107	0.35	
2	Female	Neutral	Tone 3	/pu/	2	180.628	139.91	49.784	0.388	
2	Female	Neutral	Tone 4	/pu/	1	295.807	254.33	50.498	0.257	
2	Female	Neutral	Tone 4	/pu/	2	270.162	236.815	51.127	0.328	
2	Female	Sadness	Tone 1	/ci/	1	411.032	12.411	57.368	0.262	
2	Female	Sadness	Tone 1	/ci/	2	422.665	19.643	58.526	0.294	
2	Female	Sadness	Tone 2	/ci/	1	322.211	120.37	54.369	0.342	
2	Female	Sadness	Tone 2	/ci/	2	330.527	112.021	55.154	0.285	
2	Female	Sadness	Tone 3	/ci/	1	271.536	147.75	53.348	0.4	
2	Female	Sadness	Tone 3	/ci/	2	291.942	214.299	54.486	0.352	
2	Female	Sadness	Tone 4	/ci/	1	414.206	189.323	59.771	0.297	
2	Female	Sadness	Tone 4	/ci/	2	378.936	162.632	57.342	0.355	
2	Female	Sadness	Tone 1	/fa/	1	439.625	29.779	63.758	0.278	
2	Female	Sadness	Tone 1	/fa/	2	402.759	20.688	63.348	0.276	
2	Female	Sadness	Tone 2	/fa/	1	309.959	124.801	58.032	0.356	
2	Female	Sadness	Tone 2	/fa/	2	295.603	152.277	58.692	0.334	
2	Female	Sadness	Tone 3	/fa/	1	247.156	178.131	58.76	0.368	
2	Female	Sadness	Tone 3	/fa/	2	267.469	183.802	58.59	0.361	
2	Female	Sadness	Tone 4	/fa/	1	390.919	148.415	60.524	0.303	
2	Female	Sadness	Tone 4	/fa/	2	369.935	151.152	59.543	0.33	
2	Female	Sadness	Tone 1	/pu/	1	427.888	41.259	58.237	0.225	

2	Female	Sadness	Tone 1	/pu/	2	418.946	41.321	58.236	0.296	
2	Female	Sadness	Tone 2	/pu/	1	343.021	181.876	53.772	0.315	
2	Female	Sadness	Tone 2	/pu/	2	345.162	170.673	55.1	0.335	
2	Female	Sadness	Tone 3	/pu/	1	289.18	223.932	53.285	0.355	
2	Female	Sadness	Tone 3	/pu/	2	308.765	207.38	53.458	0.315	
2	Female	Sadness	Tone 4	/pu/	1	412.511	187.879	60.787	0.335	
2	Female	Sadness	Tone 4	/pu/	2	387.505	215.462	58.904	0.33	
3	Male	Anger	Tone 1	/ci/	1	375.026	28.057	76.773	0.132	
3	Male	Anger	Tone 1	/ci/	2	362.567	43.884	77.126	0.136	
3	Male	Anger	Tone 2	/ci/	1	304.364	48.263	75.735	0.16	
3	Male	Anger	Tone 2	/ci/	2	303.394	56.121	75.633	0.127	
3	Male	Anger	Tone 3	/ci/	1	233.259	125.25	71.654	0.18	
3	Male	Anger	Tone 3	/ci/	2	232.034	108.305	69.256	0.14	
3	Male	Anger	Tone 4	/ci/	1	379.815	103.636	73.522	0.125	
3	Male	Anger	Tone 4	/ci/	2	373.928	140.018	75.376	0.135	
3	Male	Anger	Tone 1	/fa/	1	321.019	54.267	74.967	0.163	
3	Male	Anger	Tone 1	/fa/	2	323.653	41.784	79.453	0.171	
3	Male	Anger	Tone 2	/fa/	1	367.099	52.821	76.604	0.17	
3	Male	Anger	Tone 2	/fa/	2	302.836	69.809	77.814	0.18	
3	Male	Anger	Tone 3	/fa/	1	267.291	68.933	76.675	0.17	
3	Male	Anger	Tone 3	/fa/	2	238.118	101.372	74.251	0.191	
3	Male	Anger	Tone 4	/fa/	1	337.509	97.907	78.178	0.143	
3	Male	Anger	Tone 4	/fa/	2	325.49	135.827	74.1	0.15	
3	Male	Anger	Tone 1	/pu/	1	378.728	46.502	78.582	0.135	
3	Male	Anger	Tone 1	/pu/	2	386.098	49.977	79.97	0.125	
3	Male	Anger	Tone 2	/pu/	1	351.969	59.342	76.378	0.135	
3	Male	Anger	Tone 2	/pu/	2	334.248	77.289	74.252	0.135	
3	Male	Anger	Tone 3	/pu/	1	270.159	124.612	75.252	0.155	
3	Male	Anger	Tone 3	/pu/	2	287.867	53.42	75.858	0.13	
3	Male	Anger	Tone 4	/pu/	1	368.608	118.933	76.528	0.166	
3	Male	Anger	Tone 4	/pu/	2	376.443	131.825	76.957	0.145	
3	Male	Fear	Tone 1	/ci/	1	332.018	34.168	63.023	0.14	
3	Male	Fear	Tone 1	/ci/	2	316.307	26.847	62.754	0.151	
3	Male	Fear	Tone 2	/ci/	1	200.096	81.347	58.681	0.142	
3	Male	Fear	Tone 2	/ci/	2	218.159	79.058	53.975	0.145	
3	Male	Fear	Tone 3	/ci/	1	141.678	26.083	54.036	0.139	
3	Male	Fear	Tone 3	/ci/	2	160.895	75.459	57.346	0.149	
3	Male	Fear	Tone 4	/ci/	1	271.994	179.69	62.743	0.166	
3	Male	Fear	Tone 4	/ci/	2	280.362	180.996	62.809	0.167	
3	Male	Fear	Tone 1	/fa/	1	268.035	47.235	55.146	0.183	
3	Male	Fear	Tone 1	/fa/	2	278.344	47.516	55.992	0.166	
3	Male	Fear	Tone 2	/fa/	1	207.477	115.838	53.004	0.202	
3	Male	Fear	Tone 2	/fa/	2	242.167	103.019	55.071	0.214	
3	Male	Fear	Tone 3	/fa/	1	161.796	93.141	53.368	0.188	
3	Male	Fear	Tone 3	/fa/	2	175.656	86.615	50.723	0.197	
3	Male	Fear	Tone 4	/fa/	1	333.07	94.307	56.9	0.14	
3	Male	Fear	Tone 4	/fa/	2	322.836	132.046	56.993	0.191	

3	Male	Fear	Tone 1	/pu/	1	298.319	27.081	60.115	0.16	
3	Male	Fear	Tone 1	/pu/	2	304.572	24.503	61.065	0.17	
3	Male	Fear	Tone 2	/pu/	1	219.823	90.288	61.509	0.19	
3	Male	Fear	Tone 2	/pu/	2	234.34	78.956	60.933	0.2	
3	Male	Fear	Tone 3	/pu/	1	141.163	67.308	58.991	0.187	
3	Male	Fear	Tone 3	/pu/	2	157.855	80.899	55.691	0.16	
3	Male	Fear	Tone 4	/pu/	1	290.208	99.678	60.799	0.13	
3	Male	Fear	Tone 4	/pu/	2	316.937	120.241	63.147	0.175	
3	Male	appines	Tone 1	/ci/	1	310.958	41.812	67.701	0.154	
3	Male	appines	Tone 1	/ci/	2	314.754	44.073	68.132	0.155	
3	Male	appines	Tone 2	/ci/	1	234.99	89.727	66.029	0.195	
3	Male	appines	Tone 2	/ci/	2	228.464	107.295	66.126	0.175	
3	Male	appines	Tone 3	/ci/	1	181.489	76.222	65.221	0.153	
3	Male	appines	Tone 3	/ci/	2	200.367	78.468	66.713	0.152	
3	Male	appines	Tone 4	/ci/	1	304.801	199.235	69.191	0.172	
3	Male	appines	Tone 4	/ci/	2	312.535	175.615	69.945	0.165	
3	Male	appines	Tone 1	/fa/	1	279.218	38.498	64.262	0.172	
3	Male	appines	Tone 1	/fa/	2	276.104	30.253	68.128	0.193	
3	Male	appines	Tone 2	/fa/	1	203.98	120.248	70.406	0.184	
3	Male	appines	Tone 2	/fa/	2	209.933	112.023	63.909	0.243	
3	Male	appines	Tone 3	/fa/	1	158.692	108.552	68.261	0.217	
3	Male	appines	Tone 3	/fa/	2	167.005	97.951	70.042	0.2	
3	Male	appines	Tone 4	/fa/	1	298.532	177.905	71.325	0.207	
3	Male	appines	Tone 4	/fa/	2	264.944	187.975	69.694	0.229	
3	Male	appines	Tone 1	/pu/	1	315.216	78.951	68.836	0.175	
3	Male	appines	Tone 1	/pu/	2	343.533	62.653	71.806	0.175	
3	Male	appines	Tone 2	/pu/	1	248.282	113.97	69.174	0.211	
3	Male	appines	Tone 2	/pu/	2	254.441	101.844	68.133	0.195	
3	Male	appines	Tone 3	/pu/	1	175.788	85.675	69.432	0.228	
3	Male	appines	Tone 3	/pu/	2	184.42	74.629	67.728	0.198	
3	Male	appines	Tone 4	/pu/	1	292.18	187.837	67.388	0.2	
3	Male	appines	Tone 4	/pu/	2	266.145	196.694	67.818	0.245	
3	Male	Neutral	Tone 1	/ci/	1	202.976	26.926	57.956	0.125	
3	Male	Neutral	Tone 1	/ci/	2	201.432	27.298	58.469	0.135	
3	Male	Neutral	Tone 2	/ci/	1	125.901	60.417	52.108	0.21	
3	Male	Neutral	Tone 2	/ci/	2	130.15	52.718	56.457	0.201	
3	Male	Neutral	Tone 3	/ci/	1	91.269	36.479	51.166	0.165	
3	Male	Neutral	Tone 3	/ci/	2	92.16	37.678	48.331	0.167	
3	Male	Neutral	Tone 4	/ci/	1	192.691	134.48	58.712	0.165	
3	Male	Neutral	Tone 4	/ci/	2	183.431	136.469	57.141	0.148	
3	Male	Neutral	Tone 1	/fa/	1	199.083	23.885	63.539	0.196	
3	Male	Neutral	Tone 1	/fa/	2	188.306	16.429	60.749	0.191	
3	Male	Neutral	Tone 2	/fa/	1	110.417	41.868	60.233	0.167	
3	Male	Neutral	Tone 2	/fa/	2	109.95	58.45	60.522	0.178	
3	Male	Neutral	Tone 3	/fa/	1	87.059	6.641	58.01	0.124	
3	Male	Neutral	Tone 3	/fa/	2	90.774	20.777	56.982	0.193	
3	Male	Neutral	Tone 4	/fa/	1	201.25	118.715	65.62	0.169	

3	Male	Neutral	Tone 4	/fa/	2	198.774	116.997	66.022	0.17	
3	Male	Neutral	Tone 1	/pu/	1	212.566	12.741	58.558	0.165	
3	Male	Neutral	Tone 1	/pu/	2	219.977	19.619	59.342	0.175	
3	Male	Neutral	Tone 2	/pu/	1	138.227	60.994	57.235	0.244	
3	Male	Neutral	Tone 2	/pu/	2	140.543	65.15	58.233	0.25	
3	Male	Neutral	Tone 3	/pu/	1	107.745	71.744	59.093	0.216	
3	Male	Neutral	Tone 3	/pu/	2	102.885	44.047	55.243	0.209	
3	Male	Neutral	Tone 4	/pu/	1	202.955	132.22	59.702	0.159	
3	Male	Neutral	Tone 4	/pu/	2	210.167	140.964	58.206	0.152	
3	Male	Sadness	Tone 1	/ci/	1	375.026	28.057	76.773	0.132	
3	Male	Sadness	Tone 1	/ci/	2	368.154	27.491	79.393	0.136	
3	Male	Sadness	Tone 2	/ci/	1	304.925	36.794	78.06	0.151	
3	Male	Sadness	Tone 2	/ci/	2	303.432	44.945	78.374	0.12	
3	Male	Sadness	Tone 3	/ci/	1	238.943	116.659	73.141	0.165	
3	Male	Sadness	Tone 3	/ci/	2	231.731	115.152	69.256	0.142	
3	Male	Sadness	Tone 4	/ci/	1	381.484	95.394	75.502	0.12	
3	Male	Sadness	Tone 4	/ci/	2	364.95	142.042	75.483	0.151	
3	Male	Sadness	Tone 1	/fa/	1	321.017	54.267	74.967	0.157	
3	Male	Sadness	Tone 1	/fa/	2	323.653	41.784	79.453	0.171	
3	Male	Sadness	Tone 2	/fa/	1	286.459	79.461	77.048	0.18	
3	Male	Sadness	Tone 2	/fa/	2	303.131	70.444	77.814	0.181	
3	Male	Sadness	Tone 3	/fa/	1	268.367	71.639	75.29	0.126	
3	Male	Sadness	Tone 3	/fa/	2	238.133	101.372	72.903	0.187	
3	Male	Sadness	Tone 4	/fa/	1	337.509	97.907	78.178	0.143	
3	Male	Sadness	Tone 4	/fa/	2	279.958	184.74	74.1	0.199	
3	Male	Sadness	Tone 1	/pu/	1	379.183	48.252	78.667	0.143	
3	Male	Sadness	Tone 1	/pu/	2	384.33	61.657	79.755	0.128	
3	Male	Sadness	Tone 2	/pu/	1	334.619	124.082	76.378	0.163	
3	Male	Sadness	Tone 2	/pu/	2	323.473	145.563	74.252	0.155	
3	Male	Sadness	Tone 3	/pu/	1	275.819	83.324	75.644	0.18	
3	Male	Sadness	Tone 3	/pu/	2	288.128	89.089	75.597	0.128	
3	Male	Sadness	Tone 4	/pu/	1	375.538	102.369	76.51	0.173	
3	Male	Sadness	Tone 4	/pu/	2	385.539	115.444	76.699	0.158	
4	Female	Anger	Tone 1	/ci/	1	497.613	68.854	62.541	0.252	
4	Female	Anger	Tone 1	/ci/	2	486.333	56.338	62.189	0.183	
4	Female	Anger	Tone 2	/ci/	1	333.971	223.783	56.206	0.21	
4	Female	Anger	Tone 2	/ci/	2	361.046	194.79	57.73	0.209	
4	Female	Anger	Tone 3	/ci/	1	263.101	121.367	53.641	0.219	
4	Female	Anger	Tone 3	/ci/	2	281.542	117.715	54.109	0.232	
4	Female	Anger	Tone 4	/ci/	1	469.675	372.656	62.774	0.229	
4	Female	Anger	Tone 4	/ci/	2	422.241	365.522	61.74	0.236	
4	Female	Anger	Tone 1	/fa/	1	528.523	57.871	72.397	0.263	
4	Female	Anger	Tone 1	/fa/	2	487.983	89.953	72.455	0.267	
4	Female	Anger	Tone 2	/fa/	1	338.302	175.466	69.617	0.275	
4	Female	Anger	Tone 2	/fa/	2	362.536	180.337	70.668	0.246	
4	Female	Anger	Tone 3	/fa/	1	292.756	284.823	68.363	0.26	
4	Female	Anger	Tone 3	/fa/	2	285.339	271.9	69.471	0.264	

4	Female	Anger	Tone 4	/fa/	1	473.475	314.661	72.582	0.243	
4	Female	Anger	Tone 4	/fa/	2	414.462	339.972	71.425	0.251	
4	Female	Anger	Tone 1	/pu/	1	538.585	91.281	67.591	0.235	
4	Female	Anger	Tone 1	/pu/	2	522.825	64.825	66.087	0.238	
4	Female	Anger	Tone 2	/pu/	1	388.896	224.656	61.609	0.25	
4	Female	Anger	Tone 2	/pu/	2	372.174	199.341	60.748	0.26	
4	Female	Anger	Tone 3	/pu/	1	313.55	209.017	62.404	0.281	
4	Female	Anger	Tone 3	/pu/	2	302.356	170.94	61.239	0.235	
4	Female	Anger	Tone 4	/pu/	1	384.484	352.918	65.818	0.252	
4	Female	Anger	Tone 4	/pu/	2	397.871	359.169	67.303	0.234	
4	Female	Fear	Tone 1	/ci/	1	340.914	47.496	49.982	0.232	
4	Female	Fear	Tone 1	/ci/	2	367.874	46.323	53.971	0.261	
4	Female	Fear	Tone 2	/ci/	1	289.518	95.283	48.106	0.256	
4	Female	Fear	Tone 2	/ci/	2	266.831	80.358	46.935	0.243	
4	Female	Fear	Tone 3	/ci/	1	256.512	86.233	47.736	0.214	
4	Female	Fear	Tone 3	/ci/	2	244.647	95.582	45.136	0.245	
4	Female	Fear	Tone 4	/ci/	1	314.926	137.582	50.666	0.248	
4	Female	Fear	Tone 4	/ci/	2	313.691	127.169	47.789	0.208	
4	Female	Fear	Tone 1	/fa/	1	340.882	38.438	49.439	0.295	
4	Female	Fear	Tone 1	/fa/	2	342.928	44.445	47.54	0.26	
4	Female	Fear	Tone 2	/fa/	1	276	100.513	42.576	0.255	
4	Female	Fear	Tone 2	/fa/	2	282.002	91.757	42.851	0.276	
4	Female	Fear	Tone 3	/fa/	1	246.105	93.425	42.631	0.28	
4	Female	Fear	Tone 3	/fa/	2	244.998	127.394	44.983	0.275	
4	Female	Fear	Tone 4	/fa/	1	330.315	101.862	45.771	0.264	
4	Female	Fear	Tone 4	/fa/	2	325.195	102.261	46.613	0.251	
4	Female	Fear	Tone 1	/pu/	1	349.039	68.502	50.438	0.275	
4	Female	Fear	Tone 1	/pu/	2	353.648	68.594	52.896	0.243	
4	Female	Fear	Tone 2	/pu/	1	278.506	84.629	45.46	0.235	
4	Female	Fear	Tone 2	/pu/	2	283.493	73.218	45.637	0.23	
4	Female	Fear	Tone 3	/pu/	1	239.995	138.22	45.072	0.246	
4	Female	Fear	Tone 3	/pu/	2	228.062	136.275	45.101	0.255	
4	Female	Fear	Tone 4	/pu/	1	320.649	141.597	49.505	0.201	
4	Female	Fear	Tone 4	/pu/	2	306.765	127.337	49.238	0.245	
4	Female	appines	Tone 1	/ci/	1	348.971	51.14	48.975	0.324	
4	Female	appines	Tone 1	/ci/	2	354.103	47.245	50.025	0.325	
4	Female	appines	Tone 2	/ci/	1	234.575	73.985	46.183	0.324	
4	Female	appines	Tone 2	/ci/	2	253.743	109.766	43.993	0.331	
4	Female	appines	Tone 3	/ci/	1	211.723	115.201	45.265	0.221	
4	Female	appines	Tone 3	/ci/	2	209.483	152.037	46.44	0.281	
4	Female	appines	Tone 4	/ci/	1	333.805	200.382	49.024	0.365	
4	Female	appines	Tone 4	/ci/	2	372.885	249.897	50.185	0.312	
4	Female	appines	Tone 1	/fa/	1	337.76	67.537	55.323	0.353	
4	Female	appines	Tone 1	/fa/	2	341.35	60.75	55.413	0.345	
4	Female	appines	Tone 2	/fa/	1	211.816	118.31	55.323	0.413	
4	Female	appines	Tone 2	/fa/	2	210.771	111.307	54.259	0.414	
4	Female	appines	Tone 3	/fa/	1	220.825	177.66	51.601	0.252	

4	Female	appines	Tone 3	/fa/	2	221.901	178.671	48.527	0.269	
4	Female	appines	Tone 4	/fa/	1	330.512	273.205	50.772	0.389	
4	Female	appines	Tone 4	/fa/	2	323.058	214.964	54.007	0.39	
4	Female	appines	Tone 1	/pu/	1	427.119	58.012	52.888	0.295	
4	Female	appines	Tone 1	/pu/	2	405.082	62.472	48.905	0.304	
4	Female	appines	Tone 2	/pu/	1	234.781	95.864	51.06	0.299	
4	Female	appines	Tone 2	/pu/	2	235.857	99.841	47.986	0.316	
4	Female	appines	Tone 3	/pu/	1	214.275	147.047	47.486	0.309	
4	Female	appines	Tone 3	/pu/	2	214.398	181.84	46.897	0.346	
4	Female	appines	Tone 4	/pu/	1	317.101	231.114	47.685	0.369	
4	Female	appines	Tone 4	/pu/	2	337.23	252.778	51.582	0.417	
4	Female	Neutral	Tone 1	/ci/	1	281.73	29.098	43.422	0.189	
4	Female	Neutral	Tone 1	/ci/	2	280.666	19.986	42.944	0.191	
4	Female	Neutral	Tone 2	/ci/	1	209.172	40.877	41.662	0.21	
4	Female	Neutral	Tone 2	/ci/	2	208.762	43.771	42.037	0.217	
4	Female	Neutral	Tone 3	/ci/	1	173.024	48.806	41.697	0.189	
4	Female	Neutral	Tone 3	/ci/	2	157.939	48.222	41.845	0.25	
4	Female	Neutral	Tone 4	/ci/	1	259.144	133.061	42.567	0.209	
4	Female	Neutral	Tone 4	/ci/	2	262.872	104.088	44.056	0.175	
4	Female	Neutral	Tone 1	/fa/	1	290.869	44.925	47.684	0.24	
4	Female	Neutral	Tone 1	/fa/	2	282.896	48.09	49.762	0.245	
4	Female	Neutral	Tone 2	/fa/	1	191.739	54.509	44.41	0.23	
4	Female	Neutral	Tone 2	/fa/	2	192.519	74.796	42.856	0.26	
4	Female	Neutral	Tone 3	/fa/	1	158.017	16.483	38.829	0.221	
4	Female	Neutral	Tone 3	/fa/	2	160.591	12.826	38.832	0.241	
4	Female	Neutral	Tone 4	/fa/	1	264.624	179.865	44.218	0.26	
4	Female	Neutral	Tone 4	/fa/	2	268.758	154.591	46.611	0.24	
4	Female	Neutral	Tone 1	/pu/	1	297.85	46.545	46.355	0.242	
4	Female	Neutral	Tone 1	/pu/	2	281.858	41.514	43.868	0.26	
4	Female	Neutral	Tone 2	/pu/	1	206.768	58.378	44.074	0.235	
4	Female	Neutral	Tone 2	/pu/	2	209.342	54.121	42.039	0.254	
4	Female	Neutral	Tone 3	/pu/	1	241.278	73.815	42.148	0.251	
4	Female	Neutral	Tone 3	/pu/	2	188.178	123.245	41.139	0.23	
4	Female	Neutral	Tone 4	/pu/	1	287.35	174.618	45.955	0.284	
4	Female	Neutral	Tone 4	/pu/	2	285.066	196.955	45.643	0.28	
4	Female	Sadness	Tone 1	/ci/	1	376.059	18.869	51.45	0.32	
4	Female	Sadness	Tone 1	/ci/	2	385.19	32.197	51.344	0.291	
4	Female	Sadness	Tone 2	/ci/	1	314.05	74.779	48.745	0.385	
4	Female	Sadness	Tone 2	/ci/	2	289.255	54.263	47.086	0.369	
4	Female	Sadness	Tone 3	/ci/	1	292.42	127.061	47.097	0.327	
4	Female	Sadness	Tone 3	/ci/	2	276.711	122.349	46.712	0.335	
4	Female	Sadness	Tone 4	/ci/	1	349.787	199.916	48.634	0.347	
4	Female	Sadness	Tone 4	/ci/	2	366.102	190.183	50.737	0.348	
4	Female	Sadness	Tone 1	/fa/	1	344.621	63.779	60.645	0.38	
4	Female	Sadness	Tone 1	/fa/	2	351.222	53.665	60.511	0.375	
4	Female	Sadness	Tone 2	/fa/	1	285.713	95.793	58.027	0.387	
4	Female	Sadness	Tone 2	/fa/	2	288.931	120.178	57.913	0.391	

4	Female	Sadness	Tone 3	/fa/	1	265.275	132.256	56.733	0.374	
4	Female	Sadness	Tone 3	/fa/	2	265.88	128.626	58.503	0.388	
4	Female	Sadness	Tone 4	/fa/	1	344.421	197.109	59.805	0.394	
4	Female	Sadness	Tone 4	/fa/	2	342.374	190.338	57.879	0.414	
4	Female	Sadness	Tone 1	/pu/	1	383.879	48.498	54.518	0.335	
4	Female	Sadness	Tone 1	/pu/	2	376.083	53.072	53.557	0.31	
4	Female	Sadness	Tone 2	/pu/	1	316.757	98.773	49.739	0.378	
4	Female	Sadness	Tone 2	/pu/	2	304.016	92.035	49.166	0.389	
4	Female	Sadness	Tone 3	/pu/	1	278.787	151.243	49.063	0.41	
4	Female	Sadness	Tone 3	/pu/	2	270.503	145.83	51.449	0.396	
4	Female	Sadness	Tone 4	/pu/	1	362.667	200.215	53.493	0.368	
4	Female	Sadness	Tone 4	/pu/	2	368.044	202.648	54.092	0.375	
5	Female	Anger	Tone 1	/ci/	1	681.127	114.736	77.135	0.14	
5	Female	Anger	Tone 1	/ci/	2	662.804	118.602	76.566	0.17	
5	Female	Anger	Tone 2	/ci/	1	600.047	155.909	74.781	0.16	
5	Female	Anger	Tone 2	/ci/	2	630.788	142.456	74.932	0.19	
5	Female	Anger	Tone 3	/ci/	1	505.464	106.566	69.427	0.16	
5	Female	Anger	Tone 3	/ci/	2	464.547	107.043	68.862	0.149	
5	Female	Anger	Tone 4	/ci/	1	713.087	124.072	78.382	0.17	
5	Female	Anger	Tone 4	/ci/	2	658.803	106.777	76.529	0.16	
5	Female	Anger	Tone 1	/fa/	1	632.818	127.419	82.405	0.19	
5	Female	Anger	Tone 1	/fa/	2	643.327	98.088	84.23	0.19	
5	Female	Anger	Tone 2	/fa/	1	555.034	231.471	83.077	0.21	
5	Female	Anger	Tone 2	/fa/	2	619.188	177.476	83.437	0.19	
5	Female	Anger	Tone 3	/fa/	1	445.924	217.872	83.652	0.186	
5	Female	Anger	Tone 3	/fa/	2	519.031	258.904	84.883	0.181	
5	Female	Anger	Tone 4	/fa/	1	721.038	313.535	83.264	0.17	
5	Female	Anger	Tone 4	/fa/	2	708.771	315.351	85.893	0.19	
5	Female	Anger	Tone 1	/pu/	1	747.419	182.341	80.238	0.2	
5	Female	Anger	Tone 1	/pu/	2	759.826	130.409	79.271	0.22	
5	Female	Anger	Tone 2	/pu/	1	520.987	258.723	75.402	0.24	
5	Female	Anger	Tone 2	/pu/	2	559.27	189.132	76.424	0.24	
5	Female	Anger	Tone 3	/pu/	1	447.266	97.562	75.563	0.185	
5	Female	Anger	Tone 3	/pu/	2	471.46	198.1	78.051	0.185	
5	Female	Anger	Tone 4	/pu/	1	662.662	311.622	84.313	0.22	
5	Female	Anger	Tone 4	/pu/	2	683.545	271.499	81.86	0.22	
5	Female	Fear	Tone 1	/ci/	1	457.406	48.894	60.347	0.165	
5	Female	Fear	Tone 1	/ci/	2	478.987	58.161	61.703	0.155	
5	Female	Fear	Tone 2	/ci/	1	408.394	33.896	60.285	0.169	
5	Female	Fear	Tone 2	/ci/	2	420.964	29.086	59.227	0.162	
5	Female	Fear	Tone 3	/ci/	1	314.265	117.097	50.493	0.189	
5	Female	Fear	Tone 3	/ci/	2	320.017	128.939	52.647	0.16	
5	Female	Fear	Tone 4	/ci/	1	502.06	117.085	63.105	0.137	
5	Female	Fear	Tone 4	/ci/	2	493.256	164.497	61.542	0.155	
5	Female	Fear	Tone 1	/fa/	1	491.178	42.269	68.085	0.185	
5	Female	Fear	Tone 1	/fa/	2	468.547	19.369	68.809	0.183	
5	Female	Fear	Tone 2	/fa/	1	311.472	99.071	60.866	0.225	

5	Female	Fear	Tone 2	/fa/	2	329.111	85.046	62.96	0.264	
5	Female	Fear	Tone 3	/fa/	1	361.567	102.291	63.675	0.19	
5	Female	Fear	Tone 3	/fa/	2	385.387	118.2	68.308	0.199	
5	Female	Fear	Tone 4	/fa/	1	423.598	97.398	65.87	0.232	
5	Female	Fear	Tone 4	/fa/	2	445.243	79.936	66.819	0.225	
5	Female	Fear	Tone 1	/pu/	1	406.476	57.717	56.596	0.189	
5	Female	Fear	Tone 1	/pu/	2	402.479	65.026	57.76	0.16	
5	Female	Fear	Tone 2	/pu/	1	296.656	44.706	50.582	0.225	
5	Female	Fear	Tone 2	/pu/	2	298.244	53.938	49.884	0.205	
5	Female	Fear	Tone 3	/pu/	1	234.119	82.162	48.152	0.145	
5	Female	Fear	Tone 3	/pu/	2	265.451	155	49.025	0.253	
5	Female	Fear	Tone 4	/pu/	1	445.445	115.108	62.058	0.16	
5	Female	Fear	Tone 4	/pu/	2	454.805	152.528	61.758	0.17	
5	Female	appines	Tone 1	/ci/	1	439.695	61.55	59.1	0.122	
5	Female	appines	Tone 1	/ci/	2	430.648	104.407	57.981	0.148	
5	Female	appines	Tone 2	/ci/	1	259.669	111.631	52.892	0.173	
5	Female	appines	Tone 2	/ci/	2	245.145	123.991	52.137	0.201	
5	Female	appines	Tone 3	/ci/	1	263.744	88.945	51.273	0.152	
5	Female	appines	Tone 3	/ci/	2	279.814	57.906	52.031	0.137	
5	Female	appines	Tone 4	/ci/	1	525.428	76.709	67.293	0.151	
5	Female	appines	Tone 4	/ci/	2	487.522	44.885	63.703	0.135	
5	Female	appines	Tone 1	/fa/	1	481.83	149.085	72.943	0.183	
5	Female	appines	Tone 1	/fa/	2	475.787	197.619	74.323	0.181	
5	Female	appines	Tone 2	/fa/	1	237.442	162.591	69.868	0.206	
5	Female	appines	Tone 2	/fa/	2	239.632	170.324	66.547	0.191	
5	Female	appines	Tone 3	/fa/	1	282.055	177.696	69.482	0.2	
5	Female	appines	Tone 3	/fa/	2	288.955	119.473	69.626	0.134	
5	Female	appines	Tone 4	/fa/	1	476.784	96.508	72.369	0.205	
5	Female	appines	Tone 4	/fa/	2	483.442	82.362	73.577	0.18	
5	Female	appines	Tone 1	/pu/	1	534.211	176.878	65.309	0.14	
5	Female	appines	Tone 1	/pu/	2	489.082	171.302	64.027	0.17	
5	Female	appines	Tone 2	/pu/	1	277.295	97.757	54.51	0.208	
5	Female	appines	Tone 2	/pu/	2	216.458	118.066	56.221	0.215	
5	Female	appines	Tone 3	/pu/	1	313.767	221.755	58.549	0.171	
5	Female	appines	Tone 3	/pu/	2	299.958	245.162	57.928	0.212	
5	Female	appines	Tone 4	/pu/	1	508.04	86.048	70.473	0.188	
5	Female	appines	Tone 4	/pu/	2	509.94	97.033	65.943	0.183	
5	Female	Neutral	Tone 1	/ci/	1	379.321	32.971	51.464	0.152	
5	Female	Neutral	Tone 1	/ci/	2	392	28.965	52.192	0.155	
5	Female	Neutral	Tone 2	/ci/	1	231.317	103.984	48.66	0.18	
5	Female	Neutral	Tone 2	/ci/	2	209.885	91.622	48.125	0.214	
5	Female	Neutral	Tone 3	/ci/	1	177.219	79.179	46.099	0.099	
5	Female	Neutral	Tone 3	/ci/	2	201.647	74.739	48.359	0.112	
5	Female	Neutral	Tone 4	/ci/	1	369.062	210.055	54.514	0.213	
5	Female	Neutral	Tone 4	/ci/	2	366.844	225.884	52.865	0.182	
5	Female	Neutral	Tone 1	/fa/	1	357.434	36.73	57.345	0.172	
5	Female	Neutral	Tone 1	/fa/	2	376.214	33.531	62.028	0.168	

5	Female	Neutral	Tone 2	/fa/	1	222.124	91.497	60.701	0.199	
5	Female	Neutral	Tone 2	/fa/	2	173.281	90.27	57.242	0.149	
5	Female	Neutral	Tone 3	/fa/	1	228.902	89.343	61.855	0.165	
5	Female	Neutral	Tone 3	/fa/	2	242.23	131.371	58.188	0.163	
5	Female	Neutral	Tone 4	/fa/	1	362.508	195.205	61.107	0.204	
5	Female	Neutral	Tone 4	/fa/	2	369.784	195.34	62.471	0.203	
5	Female	Neutral	Tone 1	/pu/	1	379.822	41.127	50.013	0.14	
5	Female	Neutral	Tone 1	/pu/	2	370.633	35.726	49.274	0.15	
5	Female	Neutral	Tone 2	/pu/	1	216.255	94.139	47.735	0.2	
5	Female	Neutral	Tone 2	/pu/	2	203.487	68.372	46.799	0.18	
5	Female	Neutral	Tone 3	/pu/	1	192.671	123.031	47.117	0.136	
5	Female	Neutral	Tone 3	/pu/	2	190.916	98.786	46.747	0.125	
5	Female	Neutral	Tone 4	/pu/	1	359.445	186.087	51.924	0.145	
5	Female	Neutral	Tone 4	/pu/	2	346.311	236.723	52.7	0.175	
5	Female	Sadness	Tone 1	/ci/	1	359.224	48.235	52.91	0.295	
5	Female	Sadness	Tone 1	/ci/	2	407.717	41.328	56.221	0.266	
5	Female	Sadness	Tone 2	/ci/	1	328.144	42.004	52.599	0.306	
5	Female	Sadness	Tone 2	/ci/	2	308.049	39.719	49.593	0.281	
5	Female	Sadness	Tone 3	/ci/	1	397.69	88.281	63.321	0.223	
5	Female	Sadness	Tone 3	/ci/	2	440.778	67.863	64.11	0.262	
5	Female	Sadness	Tone 4	/ci/	1	449.083	110.719	64.444	0.356	
5	Female	Sadness	Tone 4	/ci/	2	520.419	130.781	72.757	0.365	
5	Female	Sadness	Tone 1	/fa/	1	416.221	79.666	66.112	0.225	
5	Female	Sadness	Tone 1	/fa/	2	558.008	52.733	75.779	0.26	
5	Female	Sadness	Tone 2	/fa/	1	338.472	93.394	74.021	0.33	
5	Female	Sadness	Tone 2	/fa/	2	340.596	87.091	74.147	0.434	
5	Female	Sadness	Tone 3	/fa/	1	387.408	179.322	77.603	0.32	
5	Female	Sadness	Tone 3	/fa/	2	375.007	151.201	76.219	0.364	
5	Female	Sadness	Tone 4	/fa/	1	559.857	72.382	82.853	0.261	
5	Female	Sadness	Tone 4	/fa/	2	614.032	61.282	84.921	0.29	
5	Female	Sadness	Tone 1	/pu/	1	530.228	42.675	70.988	0.29	
5	Female	Sadness	Tone 1	/pu/	2	375.996	23.625	66.534	0.336	
5	Female	Sadness	Tone 2	/pu/	1	355.132	58.012	59.707	0.244	
5	Female	Sadness	Tone 2	/pu/	2	325.115	37.956	62.139	0.275	
5	Female	Sadness	Tone 3	/pu/	1	387.76	188.047	63.428	0.391	
5	Female	Sadness	Tone 3	/pu/	2	431.459	174.626	65.116	0.386	
5	Female	Sadness	Tone 4	/pu/	1	519.448	138.279	70.497	0.296	
5	Female	Sadness	Tone 4	/pu/	2	396.307	132.871	60.945	0.301	
6	Male	Anger	Tone 1	/ci/	1	375.124	21.695	80.859	0.248	
6	Male	Anger	Tone 1	/ci/	2	375.124	21.695	80.859	0.248	
6	Male	Anger	Tone 2	/ci/	1	325.299	116.931	77.747	0.263	
6	Male	Anger	Tone 2	/ci/	2	318.512	117.585	77.267	0.247	
6	Male	Anger	Tone 3	/ci/	1	208.244	162.13	74.143	0.236	
6	Male	Anger	Tone 3	/ci/	2	163.82	186.187	71.817	0.234	
6	Male	Anger	Tone 4	/ci/	1	357.287	92.372	80.192	0.286	
6	Male	Anger	Tone 4	/ci/	2	357.287	92.372	80.192	0.286	
6	Male	Anger	Tone 1	/fa/	1	368.849	12.157	85.791	0.256	

6	Male	Anger	Tone 1	/fa/	2	368.849	12.157	85.791	0.256	
6	Male	Anger	Tone 2	/fa/	1	257.357	84.529	82.153	0.248	
6	Male	Anger	Tone 2	/fa/	2	299.983	102.784	80.711	0.263	
6	Male	Anger	Tone 3	/fa/	1	228.865	118.874	73.987	0.196	
6	Male	Anger	Tone 3	/fa/	2	213.805	112.081	73.241	0.264	
6	Male	Anger	Tone 4	/fa/	1	343.895	138.575	85.302	0.3	
6	Male	Anger	Tone 4	/fa/	2	322.681	144.418	83.043	0.263	
6	Male	Anger	Tone 1	/pu/	1	377.922	25.713	81.509	0.241	
6	Male	Anger	Tone 1	/pu/	2	374.313	44.177	82.072	0.226	
6	Male	Anger	Tone 2	/pu/	1	363.39	96.912	80.588	0.278	
6	Male	Anger	Tone 2	/pu/	2	355.179	99.279	77.825	0.277	
6	Male	Anger	Tone 3	/pu/	1	257.786	69.406	73.508	0.308	
6	Male	Anger	Tone 3	/pu/	2	284.12	51.621	76.764	0.315	
6	Male	Anger	Tone 4	/pu/	1	365.258	71.112	79.89	0.308	
6	Male	Anger	Tone 4	/pu/	2	357.351	122.83	80.404	0.308	
6	Male	Fear	Tone 1	/ci/	1	348.453	30.935	74.737	0.218	
6	Male	Fear	Tone 1	/ci/	2	345.511	26.704	73.896	0.225	
6	Male	Fear	Tone 2	/ci/	1	303.284	57.157	71.959	0.265	
6	Male	Fear	Tone 2	/ci/	2	303.308	55.417	71.621	0.25	
6	Male	Fear	Tone 3	/ci/	1	274.65	104.456	72.506	0.247	
6	Male	Fear	Tone 3	/ci/	2	268.631	114.061	70.26	0.231	
6	Male	Fear	Tone 4	/ci/	1	356.732	68.817	77.535	0.24	
6	Male	Fear	Tone 4	/ci/	2	333.265	83.232	75.593	0.256	
6	Male	Fear	Tone 1	/fa/	1	326.193	30.595	77.066	0.263	
6	Male	Fear	Tone 1	/fa/	2	324.343	26.806	76.38	0.27	
6	Male	Fear	Tone 2	/fa/	1	285.632	74.49	73.365	0.307	
6	Male	Fear	Tone 2	/fa/	2	276.205	96.052	71.309	0.3	
6	Male	Fear	Tone 3	/fa/	1	227.414	122.676	70.093	0.275	
6	Male	Fear	Tone 3	/fa/	2	234.739	118.445	70.573	0.296	
6	Male	Fear	Tone 4	/fa/	1	298.73	126.133	75.467	0.36	
6	Male	Fear	Tone 4	/fa/	2	311.758	121.273	75.112	0.33	
6	Male	Fear	Tone 1	/pu/	1	359.137	49.958	75.07	0.241	
6	Male	Fear	Tone 1	/pu/	2	332.107	23.922	72.58	0.24	
6	Male	Fear	Tone 2	/pu/	1	307.886	80.506	72.66	0.336	
6	Male	Fear	Tone 2	/pu/	2	304.053	91.516	74.041	0.355	
6	Male	Fear	Tone 3	/pu/	1	299.428	113.739	72.207	0.315	
6	Male	Fear	Tone 3	/pu/	2	284.8	96.55	70.941	0.306	
6	Male	Fear	Tone 4	/pu/	1	345.655	75.468	76.486	0.36	
6	Male	Fear	Tone 4	/pu/	2	338.799	66.637	75.253	0.345	
6	Male	appines	Tone 1	/ci/	1	310.95	72.192	70.136	0.21	
6	Male	appines	Tone 1	/ci/	2	319.21	58.413	69.772	0.256	
6	Male	appines	Tone 2	/ci/	1	232.501	84.23	66.434	0.29	
6	Male	appines	Tone 2	/ci/	2	228.584	104.202	65.908	0.282	
6	Male	appines	Tone 3	/ci/	1	190.857	88.735	65.585	0.268	
6	Male	appines	Tone 3	/ci/	2	209.858	47.847	64.766	0.26	
6	Male	appines	Tone 4	/ci/	1	317.425	56.128	70.85	0.24	
6	Male	appines	Tone 4	/ci/	2	312.217	73.053	70.18	0.24	

6	Male	appines	Tone 1	/fa/	1	310.567	37.43	75.759	0.225	
6	Male	appines	Tone 1	/fa/	2	299.794	34.639	77.822	0.225	
6	Male	appines	Tone 2	/fa/	1	229.452	117.7	74.163	0.279	
6	Male	appines	Tone 2	/fa/	2	233.995	101.08	73.77	0.287	
6	Male	appines	Tone 3	/fa/	1	181.847	113.897	71.509	0.305	
6	Male	appines	Tone 3	/fa/	2	185.538	113.641	74.678	0.323	
6	Male	appines	Tone 4	/fa/	1	295.819	72.622	77.884	0.363	
6	Male	appines	Tone 4	/fa/	2	282.448	94.369	77.438	0.331	
6	Male	appines	Tone 1	/pu/	1	292.618	72.699	70.184	0.255	
6	Male	appines	Tone 1	/pu/	2	317.103	82.87	69.384	0.3	
6	Male	appines	Tone 2	/pu/	1	230.637	118.369	67.255	0.302	
6	Male	appines	Tone 2	/pu/	2	239.021	119.723	66.915	0.338	
6	Male	appines	Tone 3	/pu/	1	203.283	88.583	69.997	0.284	
6	Male	appines	Tone 3	/pu/	2	209.875	88.16	70.2	0.347	
6	Male	appines	Tone 4	/pu/	1	308.551	76.839	70.228	0.33	
6	Male	appines	Tone 4	/pu/	2	326.492	56.79	71.413	0.27	
6	Male	Neutral	Tone 1	/ci/	1	249.006	38.17	64.18	0.278	
6	Male	Neutral	Tone 1	/ci/	2	237.502	29.98	68.7	0.338	
6	Male	Neutral	Tone 2	/ci/	1	174.138	85.381	60.436	0.334	
6	Male	Neutral	Tone 2	/ci/	2	164.374	71.63	58.455	0.333	
6	Male	Neutral	Tone 3	/ci/	1	122.943	68.741	57.484	0.251	
6	Male	Neutral	Tone 3	/ci/	2	123.33	65.99	56.937	0.266	
6	Male	Neutral	Tone 4	/ci/	1	223.791	156.38	63.908	0.304	
6	Male	Neutral	Tone 4	/ci/	2	215.172	156.156	63.651	0.333	
6	Male	Neutral	Tone 1	/fa/	1	247.773	23.134	71.912	0.3	
6	Male	Neutral	Tone 1	/fa/	2	224.872	32.173	68.704	0.338	
6	Male	Neutral	Tone 2	/fa/	1	168.459	119.517	64.378	0.372	
6	Male	Neutral	Tone 2	/fa/	2	174.36	104.844	65.212	0.358	
6	Male	Neutral	Tone 3	/fa/	1	115.493	84.193	59.731	0.317	
6	Male	Neutral	Tone 3	/fa/	2	118.643	85.661	59.008	0.328	
6	Male	Neutral	Tone 4	/fa/	1	205.97	158.618	68.907	0.341	
6	Male	Neutral	Tone 4	/fa/	2	208.584	142.692	69.451	0.332	
6	Male	Neutral	Tone 1	/pu/	1	275.619	40.927	65.539	0.24	
6	Male	Neutral	Tone 1	/pu/	2	246.472	26.943	62.456	0.24	
6	Male	Neutral	Tone 2	/pu/	1	173.74	122.801	61.342	0.33	
6	Male	Neutral	Tone 2	/pu/	2	171.562	113.273	60.303	0.305	
6	Male	Neutral	Tone 3	/pu/	1	121.612	66.063	58.564	0.315	
6	Male	Neutral	Tone 3	/pu/	2	127.739	64.617	58.322	0.29	
6	Male	Neutral	Tone 4	/pu/	1	217.076	170.92	63.527	0.353	
6	Male	Neutral	Tone 4	/pu/	2	217.732	162.127	61.849	0.328	
6	Male	Sadness	Tone 1	/ci/	1	297.597	25.572	72.969	0.349	
6	Male	Sadness	Tone 1	/ci/	2	315.442	37.835	71.888	0.368	
6	Male	Sadness	Tone 2	/ci/	1	270.072	36.807	69.626	0.401	
6	Male	Sadness	Tone 2	/ci/	2	281.603	39.33	69.334	0.4	
6	Male	Sadness	Tone 3	/ci/	1	252.792	77.492	68.398	0.371	
6	Male	Sadness	Tone 3	/ci/	2	249.383	85.715	68.606	0.372	
6	Male	Sadness	Tone 4	/ci/	1	294.178	64.569	72.438	0.418	

6	Male	Sadness	Tone 4	/ci/	2	311.452	75.229	72.367	0.401	
6	Male	Sadness	Tone 1	/fa/	1	286.742	36.261	78.685	0.382	
6	Male	Sadness	Tone 1	/fa/	2	297.847	16.691	75.847	0.368	
6	Male	Sadness	Tone 2	/fa/	1	255.58	79.564	76.697	0.392	
6	Male	Sadness	Tone 2	/fa/	2	268.954	60.889	77.342	0.379	
6	Male	Sadness	Tone 3	/fa/	1	241.86	86.761	76.466	0.378	
6	Male	Sadness	Tone 3	/fa/	2	258.499	74.215	77.478	0.386	
6	Male	Sadness	Tone 4	/fa/	1	300.793	64.155	77.599	0.412	
6	Male	Sadness	Tone 4	/fa/	2	290.59	74.647	75.85	0.41	
6	Male	Sadness	Tone 1	/pu/	1	307.921	37.158	76.029	0.477	
6	Male	Sadness	Tone 1	/pu/	2	293.629	29.281	75.804	0.45	
6	Male	Sadness	Tone 2	/pu/	1	264.871	37.974	71.877	0.421	
6	Male	Sadness	Tone 2	/pu/	2	282.054	49.786	73.436	0.477	
6	Male	Sadness	Tone 3	/pu/	1	243.238	47.616	69.57	0.385	
6	Male	Sadness	Tone 3	/pu/	2	246.182	54.89	70.362	0.456	
6	Male	Sadness	Tone 4	/pu/	1	291.511	41.49	75.941	0.503	
6	Male	Sadness	Tone 4	/pu/	2	283.929	95.962	74.486	0.503	
7	Male	Anger	Tone 1	/ci/	1	486.894	23.258	76.586	0.065	
7	Male	Anger	Tone 1	/ci/	2	463.205	28.036	78.822	0.093	
7	Male	Anger	Tone 2	/ci/	1	450.059	40.005	76.993	0.093	
7	Male	Anger	Tone 2	/ci/	2	451.271	33.531	77.032	0.085	
7	Male	Anger	Tone 3	/ci/	1	436.784	39.489	77.943	0.093	
7	Male	Anger	Tone 3	/ci/	2	400.47	42.019	73.749	0.091	
7	Male	Anger	Tone 4	/ci/	1	479.128	33.474	77.701	0.089	
7	Male	Anger	Tone 4	/ci/	2	472.175	34.019	78.162	0.088	
7	Male	Anger	Tone 1	/fa/	1	474.946	49.113	81.59	0.146	
7	Male	Anger	Tone 1	/fa/	2	469.013	59.095	78.316	0.145	
7	Male	Anger	Tone 2	/fa/	1	426.367	64.495	78.092	0.165	
7	Male	Anger	Tone 2	/fa/	2	428.995	50.097	76.47	0.136	
7	Male	Anger	Tone 3	/fa/	1	409.535	83.323	78.706	0.135	
7	Male	Anger	Tone 3	/fa/	2	422.431	70.658	80.408	0.145	
7	Male	Anger	Tone 4	/fa/	1	451.683	38.828	80.068	0.145	
7	Male	Anger	Tone 4	/fa/	2	443.09	41.69	77.494	0.125	
7	Male	Anger	Tone 1	/pu/	1	448.167	86.438	79.777	0.135	
7	Male	Anger	Tone 1	/pu/	2	455.199	113.049	78.518	0.135	
7	Male	Anger	Tone 2	/pu/	1	395.502	93.607	74.223	0.145	
7	Male	Anger	Tone 2	/pu/	2	422.754	87.543	74.708	0.115	
7	Male	Anger	Tone 3	/pu/	1	361.778	83.524	72.494	0.115	
7	Male	Anger	Tone 3	/pu/	2	346.613	116.887	76.598	0.125	
7	Male	Anger	Tone 4	/pu/	1	456.81	50.548	77.388	0.125	
7	Male	Anger	Tone 4	/pu/	2	456.267	79.406	77.948	0.135	
7	Male	Fear	Tone 1	/ci/	1	400.434	3.089	70.457	0.093	
7	Male	Fear	Tone 1	/ci/	2	403.531	19.98	69.253	0.119	
7	Male	Fear	Tone 2	/ci/	1	364.631	25.219	68.838	0.098	
7	Male	Fear	Tone 2	/ci/	2	387.92	12.567	67.139	0.106	
7	Male	Fear	Tone 3	/ci/	1	367.051	51.126	70.398	0.095	
7	Male	Fear	Tone 3	/ci/	2	351.179	82.493	67.314	0.109	

7	Male	Fear	Tone 4	/ci/	1	433.807	47.293	73.441	0.075	
7	Male	Fear	Tone 4	/ci/	2	419.662	74.443	72.967	0.116	
7	Male	Fear	Tone 1	/fa/	1	437.45	20.426	75.571	0.115	
7	Male	Fear	Tone 1	/fa/	2	426.807	22.733	75.271	0.115	
7	Male	Fear	Tone 2	/fa/	1	383.495	48.723	73.926	0.135	
7	Male	Fear	Tone 2	/fa/	2	388.995	52.439	76.645	0.125	
7	Male	Fear	Tone 3	/fa/	1	366.48	123.709	76.973	0.125	
7	Male	Fear	Tone 3	/fa/	2	386.116	84.763	75.298	0.122	
7	Male	Fear	Tone 4	/fa/	1	405.118	66.177	72.798	0.136	
7	Male	Fear	Tone 4	/fa/	2	416.242	72.482	76.943	0.115	
7	Male	Fear	Tone 1	/pu/	1	435.511	37.436	74.097	0.104	
7	Male	Fear	Tone 1	/pu/	2	448.508	53.51	72.705	0.105	
7	Male	Fear	Tone 2	/pu/	1	399.592	28.068	71.383	0.105	
7	Male	Fear	Tone 2	/pu/	2	403.436	24.064	73.065	0.115	
7	Male	Fear	Tone 3	/pu/	1	371.702	86.615	71.97	0.156	
7	Male	Fear	Tone 3	/pu/	2	375.945	47.826	73.541	0.135	
7	Male	Fear	Tone 4	/pu/	1	445.576	79.245	76.55	0.135	
7	Male	Fear	Tone 4	/pu/	2	434.683	80.924	75.816	0.135	
7	Male	appines	Tone 1	/ci/	1	349.784	16.27	68.877	0.133	
7	Male	appines	Tone 1	/ci/	2	356.493	23.069	66.625	0.157	
7	Male	appines	Tone 2	/ci/	1	290.645	37.925	62.649	0.13	
7	Male	appines	Tone 2	/ci/	2	300.829	43.679	62.088	0.145	
7	Male	appines	Tone 3	/ci/	1	284.505	117.968	60.291	0.165	
7	Male	appines	Tone 3	/ci/	2	306.227	134.421	63.156	0.15	
7	Male	appines	Tone 4	/ci/	1	350.184	93.657	68.402	0.164	
7	Male	appines	Tone 4	/ci/	2	330.261	148.436	65.2	0.174	
7	Male	appines	Tone 1	/fa/	1	344.238	30.364	72.165	0.181	
7	Male	appines	Tone 1	/fa/	2	354.959	28.494	75.3	0.165	
7	Male	appines	Tone 2	/fa/	1	285.047	54.991	73.492	0.12	
7	Male	appines	Tone 2	/fa/	2	279.154	71.504	72.399	0.12	
7	Male	appines	Tone 3	/fa/	1	302.615	134.253	74.242	0.18	
7	Male	appines	Tone 3	/fa/	2	298.67	121.731	72.895	0.183	
7	Male	appines	Tone 4	/fa/	1	338.936	139.065	72.739	0.232	
7	Male	appines	Tone 4	/fa/	2	349.963	104.863	71.714	0.224	
7	Male	appines	Tone 1	/pu/	1	374.346	55.034	70.796	0.159	
7	Male	appines	Tone 1	/pu/	2	359.679	69.122	67.485	0.14	
7	Male	appines	Tone 2	/pu/	1	278.944	94.186	65.527	0.143	
7	Male	appines	Tone 2	/pu/	2	291.219	66.648	66.629	0.164	
7	Male	appines	Tone 3	/pu/	1	317.638	95.857	72.235	0.135	
7	Male	appines	Tone 3	/pu/	2	302.201	91.999	70.243	0.157	
7	Male	appines	Tone 4	/pu/	1	371.126	49.25	69.777	0.152	
7	Male	appines	Tone 4	/pu/	2	381.19	118.102	68.679	0.171	
7	Male	Neutral	Tone 1	/ci/	1	181.256	8.17	53.307	0.115	
7	Male	Neutral	Tone 1	/ci/	2	171.08	24.198	53.09	0.139	
7	Male	Neutral	Tone 2	/ci/	1	119.488	30.664	51.604	0.141	
7	Male	Neutral	Tone 2	/ci/	2	111.597	23.818	50.494	0.119	
7	Male	Neutral	Tone 3	/ci/	1	116.541	40.466	50.9	0.115	

7	Male	Neutral	Tone 3	/ci/	2	124.924	44.697	51.94	0.101	
7	Male	Neutral	Tone 4	/ci/	1	190.363	63.649	53.713	0.111	
7	Male	Neutral	Tone 4	/ci/	2	180.457	63.259	52.896	0.121	
7	Male	Neutral	Tone 1	/fa/	1	189.032	23.683	62.028	0.185	
7	Male	Neutral	Tone 1	/fa/	2	168.91	33.666	59.593	0.194	
7	Male	Neutral	Tone 2	/fa/	1	125.626	18.103	55.299	0.104	
7	Male	Neutral	Tone 2	/fa/	2	117.171	13.181	55.99	0.088	
7	Male	Neutral	Tone 3	/fa/	1	116.541	40.466	50.9	0.115	
7	Male	Neutral	Tone 3	/fa/	2	124.924	44.697	51.94	0.101	
7	Male	Neutral	Tone 4	/fa/	1	186.255	66.091	62.331	0.153	
7	Male	Neutral	Tone 4	/fa/	2	190.16	95.755	63.485	0.163	
7	Male	Neutral	Tone 1	/pu/	1	195.223	19.934	55.749	0.13	
7	Male	Neutral	Tone 1	/pu/	2	199.171	14.901	56.792	0.14	
7	Male	Neutral	Tone 2	/pu/	1	128.049	13.311	53.915	0.103	
7	Male	Neutral	Tone 2	/pu/	2	132.468	34.567	52.43	0.141	
7	Male	Neutral	Tone 3	/pu/	1	126.309	52.445	56.648	0.133	
7	Male	Neutral	Tone 3	/pu/	2	126.952	41.248	57.394	0.111	
7	Male	Neutral	Tone 4	/pu/	1	190.576	74.301	61.579	0.14	
7	Male	Neutral	Tone 4	/pu/	2	178.566	86.391	62.949	0.145	
7	Male	Sadness	Tone 1	/ci/	1	425.336	52.403	71.869	0.197	
7	Male	Sadness	Tone 1	/ci/	2	423.893	37.019	72.225	0.191	
7	Male	Sadness	Tone 2	/ci/	1	375.128	43.053	70.836	0.183	
7	Male	Sadness	Tone 2	/ci/	2	369.623	42.755	68.219	0.192	
7	Male	Sadness	Tone 3	/ci/	1	366.226	115.284	69.396	0.194	
7	Male	Sadness	Tone 3	/ci/	2	409.295	121.918	70.909	0.196	
7	Male	Sadness	Tone 4	/ci/	1	414.812	72.149	73.236	0.194	
7	Male	Sadness	Tone 4	/ci/	2	400.588	67.554	73.705	0.197	
7	Male	Sadness	Tone 1	/fa/	1	412.025	41.596	80.487	0.2	
7	Male	Sadness	Tone 1	/fa/	2	417.507	53.477	83.361	0.212	
7	Male	Sadness	Tone 2	/fa/	1	399.451	68.418	82.366	0.263	
7	Male	Sadness	Tone 2	/fa/	2	393.227	77.262	81.458	0.244	
7	Male	Sadness	Tone 3	/fa/	1	385.372	104.536	81.131	0.259	
7	Male	Sadness	Tone 3	/fa/	2	379.171	156.422	80.804	0.263	
7	Male	Sadness	Tone 4	/fa/	1	400.595	109.793	81.795	0.235	
7	Male	Sadness	Tone 4	/fa/	2	374.814	151.028	81.165	0.26	
7	Male	Sadness	Tone 1	/pu/	1	433.601	14.592	78.284	0.216	
7	Male	Sadness	Tone 1	/pu/	2	413.335	18.858	75.105	0.205	
7	Male	Sadness	Tone 2	/pu/	1	387.411	34.477	74.922	0.225	
7	Male	Sadness	Tone 2	/pu/	2	403.197	29.878	75.699	0.195	
7	Male	Sadness	Tone 3	/pu/	1	439.46	233.758	75.641	0.2	
7	Male	Sadness	Tone 3	/pu/	2	327.307	168.91	71.708	0.225	
7	Male	Sadness	Tone 4	/pu/	1	428.146	124.095	76.559	0.179	
7	Male	Sadness	Tone 4	/pu/	2	392.221	104.729	76.599	0.226	
8	Male	Anger	Tone 1	/ci/	1	269.847	8.266	73.24	0.142	
8	Male	Anger	Tone 1	/ci/	2	276.034	26.163	72.621	0.145	
8	Male	Anger	Tone 2	/ci/	1	237.05	46.293	72.944	0.143	
8	Male	Anger	Tone 2	/ci/	2	229.367	46.351	71.05	0.155	

8	Male	Anger	Tone 3	/ci/	1	189.666	59.845	71.158	0.153	
8	Male	Anger	Tone 3	/ci/	2	175.423	55.915	68.126	0.149	
8	Male	Anger	Tone 4	/ci/	1	263.301	92.192	74.217	0.172	
8	Male	Anger	Tone 4	/ci/	2	261.063	111.13	73.881	0.18	
8	Male	Anger	Tone 1	/fa/	1	267.994	25.214	78.938	0.18	
8	Male	Anger	Tone 1	/fa/	2	263.452	27.615	77.139	0.175	
8	Male	Anger	Tone 2	/fa/	1	204.292	50.518	75.186	0.172	
8	Male	Anger	Tone 2	/fa/	2	227.299	46.302	80.26	0.187	
8	Male	Anger	Tone 3	/fa/	1	212.07	80.929	82.31	0.143	
8	Male	Anger	Tone 3	/fa/	2	212.07	80.929	82.31	0.143	
8	Male	Anger	Tone 4	/fa/	1	277.256	114.745	82.159	0.163	
8	Male	Anger	Tone 4	/fa/	2	281.583	60.329	83.829	0.19	
8	Male	Anger	Tone 1	/pu/	1	289.059	25.097	77.66	0.158	
8	Male	Anger	Tone 1	/pu/	2	302.962	37.217	77.701	0.165	
8	Male	Anger	Tone 2	/pu/	1	267.414	61.317	77.926	0.147	
8	Male	Anger	Tone 2	/pu/	2	265.537	62.57	78.148	0.155	
8	Male	Anger	Tone 3	/pu/	1	202.391	97.459	77.597	0.205	
8	Male	Anger	Tone 3	/pu/	2	210.278	82.942	78.361	0.204	
8	Male	Anger	Tone 4	/pu/	1	265.41	145.221	80.22	0.188	
8	Male	Anger	Tone 4	/pu/	2	294.619	110.049	82.583	0.158	
8	Male	Fear	Tone 1	/ci/	1	306.652	17.392	63.344	0.109	
8	Male	Fear	Tone 1	/ci/	2	323.162	24.83	68.79	0.144	
8	Male	Fear	Tone 2	/ci/	1	288.979	53.151	59.055	0.129	
8	Male	Fear	Tone 2	/ci/	2	288.946	26.973	58.696	0.13	
8	Male	Fear	Tone 3	/ci/	1	194.337	62.368	52.599	0.112	
8	Male	Fear	Tone 3	/ci/	2	227.954	82.754	53.288	0.102	
8	Male	Fear	Tone 4	/ci/	1	300.945	77.282	63.888	0.118	
8	Male	Fear	Tone 4	/ci/	2	284.525	51.763	62.041	0.095	
8	Male	Fear	Tone 1	/fa/	1	331.189	27.904	70.351	0.153	
8	Male	Fear	Tone 1	/fa/	2	342.024	72.805	68.824	0.139	
8	Male	Fear	Tone 2	/fa/	1	263.172	60.742	62.695	0.178	
8	Male	Fear	Tone 2	/fa/	2	289.912	60.232	63.539	0.189	
8	Male	Fear	Tone 3	/fa/	1	204.348	70.641	55.881	0.151	
8	Male	Fear	Tone 3	/fa/	2	219.511	95.806	56.142	0.165	
8	Male	Fear	Tone 4	/fa/	1	271.627	73.819	60.38	0.18	
8	Male	Fear	Tone 4	/fa/	2	293.831	104.969	59.382	0.179	
8	Male	Fear	Tone 1	/pu/	1	295.518	33.665	62.35	0.13	
8	Male	Fear	Tone 1	/pu/	2	288.79	16.17	63.435	0.145	
8	Male	Fear	Tone 2	/pu/	1	262.129	28.063	58.5	0.136	
8	Male	Fear	Tone 2	/pu/	2	289.027	48.165	62.725	0.12	
8	Male	Fear	Tone 3	/pu/	1	282.375	66.574	66.226	0.152	
8	Male	Fear	Tone 3	/pu/	2	259.206	73.679	63.09	0.179	
8	Male	Fear	Tone 4	/pu/	1	302.17	49.581	67.59	0.115	
8	Male	Fear	Tone 4	/pu/	2	306.12	34.73	68.464	0.162	
8	Male	appines	Tone 1	/ci/	1	241.807	35.463	54.822	0.182	
8	Male	appines	Tone 1	/ci/	2	239.974	40.2	53.704	0.191	
8	Male	appines	Tone 2	/ci/	1	199.073	53.496	52.719	0.204	

8	Male	appines	Tone 2	/ci/	2	202.691	50.358	52.62	0.213	
8	Male	appines	Tone 3	/ci/	1	151.327	130.918	55.712	0.232	
8	Male	appines	Tone 3	/ci/	2	157.688	103.168	55.471	0.215	
8	Male	appines	Tone 4	/ci/	1	259.707	70.231	55.857	0.204	
8	Male	appines	Tone 4	/ci/	2	246.832	80.626	55.87	0.175	
8	Male	appines	Tone 1	/fa/	1	245.989	36.919	60.403	0.23	
8	Male	appines	Tone 1	/fa/	2	242.507	43.968	60.915	0.221	
8	Male	appines	Tone 2	/fa/	1	199.397	46.491	62.563	0.208	
8	Male	appines	Tone 2	/fa/	2	199.858	34.668	62.085	0.185	
8	Male	appines	Tone 3	/fa/	1	146.178	25.901	60.265	0.217	
8	Male	appines	Tone 3	/fa/	2	140.905	19.839	60.681	0.218	
8	Male	appines	Tone 4	/fa/	1	294.122	56.343	66.203	0.201	
8	Male	appines	Tone 4	/fa/	2	277.299	56.741	67.924	0.201	
8	Male	appines	Tone 1	/pu/	1	275.456	36.067	59.716	0.202	
8	Male	appines	Tone 1	/pu/	2	262.476	40.887	58.361	0.173	
8	Male	appines	Tone 2	/pu/	1	210.68	86.238	63.931	0.24	
8	Male	appines	Tone 2	/pu/	2	211.372	84.133	62.328	0.195	
8	Male	appines	Tone 3	/pu/	1	197.778	169.977	58.447	0.21	
8	Male	appines	Tone 3	/pu/	2	183.212	141.97	60.476	0.21	
8	Male	appines	Tone 4	/pu/	1	303.232	118.303	62.776	0.201	
8	Male	appines	Tone 4	/pu/	2	282.247	98.815	62.011	0.175	
8	Male	Neutral	Tone 1	/ci/	1	176.985	26.189	49.697	0.156	
8	Male	Neutral	Tone 1	/ci/	2	195.27	19.5	50.819	0.166	
8	Male	Neutral	Tone 2	/ci/	1	109.719	36.104	52.334	0.175	
8	Male	Neutral	Tone 2	/ci/	2	111.522	26.002	51.555	0.181	
8	Male	Neutral	Tone 3	/ci/	1	98.132	35.624	50.11	0.167	
8	Male	Neutral	Tone 3	/ci/	2	98.206	40.79	49.889	0.179	
8	Male	Neutral	Tone 4	/ci/	1	184.519	133.369	55.075	0.153	
8	Male	Neutral	Tone 4	/ci/	2	183.695	140.769	55.051	0.179	
8	Male	Neutral	Tone 1	/fa/	1	142.132	8.84	61.799	0.2	
8	Male	Neutral	Tone 1	/fa/	2	151.501	12.005	59.947	0.231	
8	Male	Neutral	Tone 2	/fa/	1	110.132	37.349	59.036	0.165	
8	Male	Neutral	Tone 2	/fa/	2	105.113	40.717	55.427	0.18	
8	Male	Neutral	Tone 3	/fa/	1	80.225	9.826	56.347	0.1	
8	Male	Neutral	Tone 3	/fa/	2	80.351	22.168	55.746	0.104	
8	Male	Neutral	Tone 4	/fa/	1	190.372	102.402	59.873	0.16	
8	Male	Neutral	Tone 4	/fa/	2	198.201	110.347	58.214	0.181	
8	Male	Neutral	Tone 1	/pu/	1	185.195	30.963	54.74	0.169	
8	Male	Neutral	Tone 1	/pu/	2	182.669	23.463	53.588	0.17	
8	Male	Neutral	Tone 2	/pu/	1	118.983	34.288	52.828	0.191	
8	Male	Neutral	Tone 2	/pu/	2	121.101	37.743	52.137	0.179	
8	Male	Neutral	Tone 3	/pu/	1	103.692	64.589	49.864	0.18	
8	Male	Neutral	Tone 3	/pu/	2	106.504	61.82	51.366	0.161	
8	Male	Neutral	Tone 4	/pu/	1	210.687	160.686	57.499	0.18	
8	Male	Neutral	Tone 4	/pu/	2	189.356	142.877	58.692	0.181	
8	Male	Sadness	Tone 1	/ci/	1	276.832	52.666	60.385	0.257	
8	Male	Sadness	Tone 1	/ci/	2	276.432	12.576	59.465	0.224	

8	Male	Sadness	Tone 2	/ci/	1	244.1	43.688	55.851	0.298	
8	Male	Sadness	Tone 2	/ci/	2	246.388	39.891	55.081	0.265	
8	Male	Sadness	Tone 3	/ci/	1	225.073	92.899	55.767	0.271	
8	Male	Sadness	Tone 3	/ci/	2	222.897	78.221	54.94	0.267	
8	Male	Sadness	Tone 4	/ci/	1	271.873	87.868	60.784	0.257	
8	Male	Sadness	Tone 4	/ci/	2	273.671	75.903	61.28	0.262	
8	Male	Sadness	Tone 1	/fa/	1	279.469	32.794	69.024	0.28	
8	Male	Sadness	Tone 1	/fa/	2	280.768	34.526	69.293	0.274	
8	Male	Sadness	Tone 2	/fa/	1	246.705	69.774	64.588	0.305	
8	Male	Sadness	Tone 2	/fa/	2	229.915	64.208	63.087	0.305	
8	Male	Sadness	Tone 3	/fa/	1	200.198	132.741	64.863	0.319	
8	Male	Sadness	Tone 3	/fa/	2	219.827	110.308	65.264	0.279	
8	Male	Sadness	Tone 4	/fa/	1	269.238	105.324	67.955	0.255	
8	Male	Sadness	Tone 4	/fa/	2	267.451	84.763	68.398	0.242	
8	Male	Sadness	Tone 1	/pu/	1	302.61	42.141	63.423	0.275	
8	Male	Sadness	Tone 1	/pu/	2	302.262	32.636	63.5	0.282	
8	Male	Sadness	Tone 2	/pu/	1	269.426	94.749	60.314	0.305	
8	Male	Sadness	Tone 2	/pu/	2	289.622	100.553	61.046	0.28	
8	Male	Sadness	Tone 3	/pu/	1	241.587	180.742	58.98	0.305	
8	Male	Sadness	Tone 3	/pu/	2	246.703	172.838	61.697	0.306	
8	Male	Sadness	Tone 4	/pu/	1	292.331	69.318	64.361	0.305	
8	Male	Sadness	Tone 4	/pu/	2	295.924	67.1	64.391	0.274	

Experiment 2: tone identificaito
No. of Participant	No. of Talker	Talker gender	Syllable	Repetition	Emotion	Tone	Context	Accuracy	
1	2	Female	/ci/	1	Anger	Tone 1	In isolation	Incorrect	
1	2	Female	/ci/	2	Anger	Tone 1	In isolation	Correct	
1	1	Male	/ci/	1	Anger	Tone 1	In isolation	Correct	
1	1	Male	/ci/	2	Anger	Tone 1	In isolation	Correct	
1	2	Female	/fa/	1	Anger	Tone 1	In isolation	Incorrect	
1	2	Female	/fa/	2	Anger	Tone 1	In isolation	Correct	
1	1	Male	/fa/	1	Anger	Tone 1	In isolation	Correct	
1	1	Male	/fa/	2	Anger	Tone 1	In isolation	Incorrect	
1	2	Female	/pu/	1	Anger	Tone 1	In isolation	Incorrect	
1	2	Female	/pu/	2	Anger	Tone 1	In isolation	Correct	
1	1	Male	/pu/	1	Anger	Tone 1	In isolation	Correct	
1	1	Male	/pu/	2	Anger	Tone 1	In isolation	Incorrect	
1	2	Female	/ci/	1	Anger	Tone 2	In isolation	Correct	
1	2	Female	/ci/	2	Anger	Tone 2	In isolation	Incorrect	
1	1	Male	/ci/	1	Anger	Tone 2	In isolation	Correct	
1	1	Male	/ci/	2	Anger	Tone 2	In isolation	Correct	
1	2	Female	/fa/	1	Anger	Tone 2	In isolation	Correct	
1	2	Female	/fa/	2	Anger	Tone 2	In isolation	Correct	
1	1	Male	/fa/	1	Anger	Tone 2	In isolation	Correct	
1	1	Male	/fa/	2	Anger	Tone 2	In isolation	Correct	
1	2	Female	/pu/	1	Anger	Tone 2	In isolation	Correct	
1	2	Female	/pu/	2	Anger	Tone 2	In isolation	Correct	
1	1	Male	/pu/	1	Anger	Tone 2	In isolation	Correct	
1	1	Male	/pu/	2	Anger	Tone 2	In isolation	Correct	
1	2	Female	/ci/	1	Anger	Tone 3	In isolation	Incorrect	
1	2	Female	/ci/	2	Anger	Tone 3	In isolation	Incorrect	
1	1	Male	/ci/	1	Anger	Tone 3	In isolation	Incorrect	
1	1	Male	/ci/	2	Anger	Tone 3	In isolation	Incorrect	
1	2	Female	/fa/	1	Anger	Tone 3	In isolation	Incorrect	
1	2	Female	/fa/	2	Anger	Tone 3	In isolation	Incorrect	
1	1	Male	/fa/	1	Anger	Tone 3	In isolation	Correct	
1	1	Male	/fa/	2	Anger	Tone 3	In isolation	Correct	
1	2	Female	/pu/	1	Anger	Tone 3	In isolation	Incorrect	
1	2	Female	/pu/	2	Anger	Tone 3	In isolation	Correct	
1	1	Male	/pu/	1	Anger	Tone 3	In isolation	Incorrect	
1	1	Male	/pu/	2	Anger	Tone 3	In isolation	Incorrect	
1	2	Female	/ci/	1	Anger	Tone 4	In isolation	Correct	
1	2	Female	/ci/	2	Anger	Tone 4	In isolation	Correct	
1	1	Male	/ci/	1	Anger	Tone 4	In isolation	Correct	
1	1	Male	/ci/	2	Anger	Tone 4	In isolation	Correct	
1	2	Female	/fa/	1	Anger	Tone 4	In isolation	Correct	
1	2	Female	/fa/	2	Anger	Tone 4	In isolation	Correct	
1	1	Male	/fa/	1	Anger	Tone 4	In isolation	Correct	
1	1	Male	/fa/	2	Anger	Tone 4	In isolation	Correct	
1	2	Female	/pu/	1	Anger	Tone 4	In isolation	Correct	
1	2	Female	/pu/	2	Anger	Tone 4	In isolation	Correct	
1	1	Male	/pu/	1	Anger	Tone 4	In isolation	Correct	
1	1	Male	/pu/	2	Anger	Tone 4	In isolation	Correct	
1	2	Female	/ci/	1	Fear	Tone 1	In isolation	Correct	

1	2	Female	/ci/	2	Fear	Tone 1	In isolation	Correct	
1	1	Male	/ci/	1	Fear	Tone 1	In isolation	Correct	
1	1	Male	/ci/	2	Fear	Tone 1	In isolation	Correct	
1	2	Female	/fa/	1	Fear	Tone 1	In isolation	Correct	
1	2	Female	/fa/	2	Fear	Tone 1	In isolation	Correct	
1	1	Male	/fa/	1	Fear	Tone 1	In isolation	Correct	
1	1	Male	/fa/	2	Fear	Tone 1	In isolation	Correct	
1	2	Female	/pu/	1	Fear	Tone 1	In isolation	Correct	
1	2	Female	/pu/	2	Fear	Tone 1	In isolation	Correct	
1	1	Male	/pu/	1	Fear	Tone 1	In isolation	Correct	
1	1	Male	/pu/	2	Fear	Tone 1	In isolation	Correct	
1	2	Female	/ci/	1	Fear	Tone 2	In isolation	Incorrect	
1	2	Female	/ci/	2	Fear	Tone 2	In isolation	Incorrect	
1	1	Male	/ci/	1	Fear	Tone 2	In isolation	Correct	
1	1	Male	/ci/	2	Fear	Tone 2	In isolation	Correct	
1	2	Female	/fa/	1	Fear	Tone 2	In isolation	Correct	
1	2	Female	/fa/	2	Fear	Tone 2	In isolation	Correct	
1	1	Male	/fa/	1	Fear	Tone 2	In isolation	Correct	
1	1	Male	/fa/	2	Fear	Tone 2	In isolation	Incorrect	
1	2	Female	/pu/	1	Fear	Tone 2	In isolation	Correct	
1	2	Female	/pu/	2	Fear	Tone 2	In isolation	Incorrect	
1	1	Male	/pu/	1	Fear	Tone 2	In isolation	Incorrect	
1	1	Male	/pu/	2	Fear	Tone 2	In isolation	Incorrect	
1	2	Female	/ci/	1	Fear	Tone 3	In isolation	Correct	
1	2	Female	/ci/	2	Fear	Tone 3	In isolation	Incorrect	
1	1	Male	/ci/	1	Fear	Tone 3	In isolation	Incorrect	
1	1	Male	/ci/	2	Fear	Tone 3	In isolation	Correct	
1	2	Female	/fa/	1	Fear	Tone 3	In isolation	Correct	
1	2	Female	/fa/	2	Fear	Tone 3	In isolation	Incorrect	
1	1	Male	/fa/	1	Fear	Tone 3	In isolation	Correct	
1	1	Male	/fa/	2	Fear	Tone 3	In isolation	Correct	
1	2	Female	/pu/	1	Fear	Tone 3	In isolation	Correct	
1	2	Female	/pu/	2	Fear	Tone 3	In isolation	Correct	
1	1	Male	/pu/	1	Fear	Tone 3	In isolation	Correct	
1	1	Male	/pu/	2	Fear	Tone 3	In isolation	Correct	
1	2	Female	/ci/	1	Fear	Tone 4	In isolation	Correct	
1	2	Female	/ci/	2	Fear	Tone 4	In isolation	Correct	
1	1	Male	/ci/	1	Fear	Tone 4	In isolation	Correct	
1	1	Male	/ci/	2	Fear	Tone 4	In isolation	Correct	
1	2	Female	/fa/	1	Fear	Tone 4	In isolation	Correct	
1	2	Female	/fa/	2	Fear	Tone 4	In isolation	Correct	
1	1	Male	/fa/	1	Fear	Tone 4	In isolation	Correct	
1	1	Male	/fa/	2	Fear	Tone 4	In isolation	Correct	
1	2	Female	/pu/	1	Fear	Tone 4	In isolation	Correct	
1	2	Female	/pu/	2	Fear	Tone 4	In isolation	Correct	
1	1	Male	/pu/	1	Fear	Tone 4	In isolation	Incorrect	
1	1	Male	/pu/	2	Fear	Tone 4	In isolation	Correct	
1	2	Female	/ci/	1	Happines	Tone 1	In isolation	Incorrect	
1	2	Female	/ci/	2	Happines	Tone 1	In isolation	Incorrect	
1	1	Male	/ci/	1	Happines	Tone 1	In isolation	Incorrect	
1	1	Male	/ci/	2	Happines	Tone 1	In isolation	Correct	

1	2	Female
1	2	Female
1	1	Male
1	1	Male
1	2	Female
1	2	Female
1	1	Male
1	1	Male
1	2	Female
1	2	Female
1	1	Male
1	1	Male
1	2	Female
1	2	Female
1	1	Male
1	1	Male
1	2	Female
1	2	Female
1	1	Male
1	1	Male
1	2	Female
1	2	Female
1	1	Male
1	1	Male
1	2	Female
1	2	Female
1	1	Male
1	1	Male
1	2	Female
1	1	Male
1	1	Male
1	2	Female
1	2	Female
1	1	Male
1	1	Male
1	2	Female
1	2	Female
1	1	Male
1	1	Male
1	2	Female
1	2	Female
1	1	Male
1	1	Male
/fa/
/fa/
/fa/
/fa/
/pu/
/pu/
/pu/
/pu/
/ci/
/ci/
/ci/
/ci/
/fa/
/fa/
/fa/
/fa/
/pu/
/pu/
/pu/
/pu/
/ci/
/ci/
/ci/
/ci/
/fa/
/fa/
/fa/
/fa/
/pu/
/pu/
/pu/
/ci/
/ci/
/ci/
/ci/
/fa/
/fa/
/fa/
/fa/
/pu/
/pu/
/pu/
/pu/
HappinesTone 1In isolation Incorrect
HappinesTone 1In isolation Incorrect
HappinesTone 1In isolation Correct
HappinesTone 1In isolation Correct
HappinesTone 1In isolation Incorrect
HappinesTone 1In isolation Incorrect
HappinesTone 1In isolation Correct
HappinesTone 1In isolation Correct
HappinesTone 2In isolation Correct
HappinesTone 2In isolation Correct
HappinesTone 2In isolation Correct
HappinesTone 2In isolation Correct
HappinesTone 2In isolation Correct
HappinesTone 2In isolation Correct
HappinesTone 2In isolation Correct
HappinesTone 2In isolation Correct
HappinesTone 2In isolation Correct
HappinesTone 2In isolation Correct
HappinesTone 2In isolation Correct
HappinesTone 2In isolation Correct
HappinesTone 3In isolation Incorrect
HappinesTone 3In isolation Incorrect
HappinesTone 3In isolation Correct
HappinesTone 3In isolation Incorrect
HappinesTone 3In isolation Incorrect
HappinesTone 3In isolation Correct
HappinesTone 3In isolation Correct
HappinesTone 3In isolation Correct
1	HappinesTone 3In isolation Incorrect
HappinesTone 3In isolation Correct
HappinesTone 3In isolation Correct
HappinesTone 4In isolation Incorrect
HappinesTone 4In isolation Incorrect
HappinesTone 4In isolation Correct
HappinesTone 4In isolation Correct
HappinesTone 4In isolation Incorrect
HappinesTone 4In isolation Correct
HappinesTone 4In isolation Correct
HappinesTone 4In isolation Correct
HappinesTone 4In isolation Incorrect
HappinesTone 4In isolation Incorrect
HappinesTone 4In isolation Correct
HappinesTone 4In isolation Correct

1	2	Female
1	2	Female
1	1	Male
1	1	Male
1	2	Female
1	2	Female
1	1	Male
1	1	Male
/ci/
/ci/
/ci/
/ci/
/fa/
/fa/
/fa/
/fa/
Neutral Tone 1In isolation Correct
Neutral Tone 1In isolation Correct
Neutral Tone 1In isolation Correct
Neutral Tone 1In isolation Correct
Neutral Tone 1In isolation Correct
Neutral Tone 1In isolation Correct
Neutral Tone 1In isolation Correct
Neutral Tone 1In isolation Correct

1	2	Female	/pu/	1	Neutral	Tone 1	In isolation	Correct	
1	2	Female	/pu/	2	Neutral	Tone 1	In isolation	Correct	
1	1	Male	/pu/	1	Neutral	Tone 1	In isolation	Correct	
1	1	Male	/pu/	2	Neutral	Tone 1	In isolation	Correct	
1	2	Female	/ci/	1	Neutral	Tone 2	In isolation	Correct	
1	2	Female	/ci/	2	Neutral	Tone 2	In isolation	Correct	
1	1	Male	/ci/	1	Neutral	Tone 2	In isolation	Correct	
1	1	Male	/ci/	2	Neutral	Tone 2	In isolation	Correct	
1	2	Female	/fa/	1	Neutral	Tone 2	In isolation	Correct	
1	2	Female	/fa/	2	Neutral	Tone 2	In isolation	Correct	
1	1	Male	/fa/	1	Neutral	Tone 2	In isolation	Correct	
1	1	Male	/fa/	2	Neutral	Tone 2	In isolation	Correct	
1	2	Female	/pu/	1	Neutral	Tone 2	In isolation	Correct	
1	2	Female	/pu/	2	Neutral	Tone 2	In isolation	Correct	
1	1	Male	/pu/	1	Neutral	Tone 2	In isolation	Correct	
1	1	Male	/pu/	2	Neutral	Tone 2	In isolation	Correct	
1	2	Female	/ci/	1	Neutral	Tone 3	In isolation	Correct	
1	2	Female	/ci/	2	Neutral	Tone 3	In isolation	Correct	
1	1	Male	/ci/	1	Neutral	Tone 3	In isolation	Correct	
1	1	Male	/ci/	2	Neutral	Tone 3	In isolation	Correct	
1	2	Female	/fa/	1	Neutral	Tone 3	In isolation	Correct	
1	2	Female	/fa/	2	Neutral	Tone 3	In isolation	Correct	
1	1	Male	/fa/	1	Neutral	Tone 3	In isolation	Correct	
1	1	Male	/fa/	2	Neutral	Tone 3	In isolation	Correct	
1	2	Female	/pu/	1	Neutral	Tone 3	In isolation	Correct	
1	2	Female	/pu/	2	Neutral	Tone 3	In isolation	Correct	
1	1	Male	/pu/	1	Neutral	Tone 3	In isolation	Correct	
1	1	Male	/pu/	2	Neutral	Tone 3	In isolation	Correct	
1	2	Female	/ci/	1	Neutral	Tone 4	In isolation	Correct	
1	2	Female	/ci/	2	Neutral	Tone 4	In isolation	Correct	
1	1	Male	/ci/	1	Neutral	Tone 4	In isolation	Correct	
1	1	Male	/ci/	2	Neutral	Tone 4	In isolation	Correct	
1	2	Female	/fa/	1	Neutral	Tone 4	In isolation	Correct	
1	2	Female	/fa/	2	Neutral	Tone 4	In isolation	Correct	
1	1	Male	/fa/	1	Neutral	Tone 4	In isolation	Correct	
1	1	Male	/fa/	2	Neutral	Tone 4	In isolation	Correct	
1	2	Female	/pu/	1	Neutral	Tone 4	In isolation	Correct	
1	2	Female	/pu/	2	Neutral	Tone 4	In isolation	Correct	
1	1	Male	/pu/	1	Neutral	Tone 4	In isolation	Correct	
1	1	Male	/pu/	2	Neutral	Tone 4	In isolation	Correct	
1	2	Female	/ci/	1	Sadness	Tone 1	In isolation	Correct	
1	2	Female	/ci/	2	Sadness	Tone 1	In isolation	Correct	
1	1	Male	/ci/	1	Sadness	Tone 1	In isolation	Correct	
1	1	Male	/ci/	2	Sadness	Tone 1	In isolation	Correct	
1	2	Female	/fa/	1	Sadness	Tone 1	In isolation	Incorrect	
1	2	Female	/fa/	2	Sadness	Tone 1	In isolation	Incorrect	
1	1	Male	/fa/	1	Sadness	Tone 1	In isolation	Incorrect	
1	1	Male	/fa/	2	Sadness	Tone 1	In isolation	Correct	
1	2	Female	/pu/	1	Sadness	Tone 1	In isolation	Correct	
1	2	Female	/pu/	2	Sadness	Tone 1	In isolation	Incorrect	
1	1	Male	/pu/	1	Sadness	Tone 1	In isolation	Correct	

1	1	Male	/pu/	2	Sadness	Tone 1	In isolation	Correct	
1	2	Female	/ci/	1	Sadness	Tone 2	In isolation	Correct	
1	2	Female	/ci/	2	Sadness	Tone 2	In isolation	Incorrect	
1	1	Male	/ci/	1	Sadness	Tone 2	In isolation	Correct	
1	1	Male	/ci/	2	Sadness	Tone 2	In isolation	Correct	
1	2	Female	/fa/	1	Sadness	Tone 2	In isolation	Correct	
1	2	Female	/fa/	2	Sadness	Tone 2	In isolation	Correct	
1	1	Male	/fa/	1	Sadness	Tone 2	In isolation	Correct	
1	1	Male	/fa/	2	Sadness	Tone 2	In isolation	Correct	
1	2	Female	/pu/	1	Sadness	Tone 2	In isolation	Incorrect	
1	2	Female	/pu/	2	Sadness	Tone 2	In isolation	Correct	
1	1	Male	/pu/	1	Sadness	Tone 2	In isolation	Correct	
1	1	Male	/pu/	2	Sadness	Tone 2	In isolation	Incorrect	
1	2	Female	/ci/	1	Sadness	Tone 3	In isolation	Correct	
1	2	Female	/ci/	2	Sadness	Tone 3	In isolation	Correct	
1	1	Male	/ci/	1	Sadness	Tone 3	In isolation	Correct	
1	1	Male	/ci/	2	Sadness	Tone 3	In isolation	Incorrect	
1	2	Female	/fa/	1	Sadness	Tone 3	In isolation	Correct	
1	2	Female	/fa/	2	Sadness	Tone 3	In isolation	Correct	
1	1	Male	/fa/	1	Sadness	Tone 3	In isolation	Correct	
1	1	Male	/fa/	2	Sadness	Tone 3	In isolation	Correct	
1	2	Female	/pu/	1	Sadness	Tone 3	In isolation	Correct	
1	2	Female	/pu/	2	Sadness	Tone 3	In isolation	Correct	
1	1	Male	/pu/	1	Sadness	Tone 3	In isolation	Correct	
1	1	Male	/pu/	2	Sadness	Tone 3	In isolation	Correct	
1	2	Female	/ci/	1	Sadness	Tone 4	In isolation	Correct	
1	2	Female	/ci/	2	Sadness	Tone 4	In isolation	Correct	
1	1	Male	/ci/	1	Sadness	Tone 4	In isolation	Correct	
1	1	Male	/ci/	2	Sadness	Tone 4	In isolation	Correct	
1	2	Female	/fa/	1	Sadness	Tone 4	In isolation	Correct	
1	2	Female	/fa/	2	Sadness	Tone 4	In isolation	Correct	
1	1	Male	/fa/	1	Sadness	Tone 4	In isolation	Incorrect	
1	1	Male	/fa/	2	Sadness	Tone 4	In isolation	Correct	
1	2	Female	/pu/	1	Sadness	Tone 4	In isolation	Correct	
1	2	Female	/pu/	2	Sadness	Tone 4	In isolation	Incorrect	
1	1	Male	/pu/	1	Sadness	Tone 4	In isolation	Incorrect	
1	1	Male	/pu/	2	Sadness	Tone 4	In isolation	Incorrect	
1	2	Female	/ci/	1	Anger	Tone 1	In context	Incorrect	
1	2	Female	/ci/	2	Anger	Tone 1	In context	Incorrect	
1	1	Male	/ci/	1	Anger	Tone 1	In context	Correct	
1	1	Male	/ci/	2	Anger	Tone 1	In context	Correct	
1	2	Female	/fa/	1	Anger	Tone 1	In context	Correct	
1	2	Female	/fa/	2	Anger	Tone 1	In context	Correct	
1	1	Male	/fa/	1	Anger	Tone 1	In context	Correct	
1	1	Male	/fa/	2	Anger	Tone 1	In context	Correct	
1	2	Female	/pu/	1	Anger	Tone 1	In context	Incorrect	
1	2	Female	/pu/	2	Anger	Tone 1	In context	Incorrect	
1	1	Male	/pu/	1	Anger	Tone 1	In context	Correct	
1	1	Male	/pu/	2	Anger	Tone 1	In context	Correct	
1	2	Female	/ci/	1	Anger	Tone 2	In context	Correct	
1	2	Female	/ci/	2	Anger	Tone 2	In context	Correct	

1	1	Male	/ci/	1	Anger	Tone 2	In context	Correct	
1	1	Male	/ci/	2	Anger	Tone 2	In context	Correct	
1	2	Female	/fa/	1	Anger	Tone 2	In context	Correct	
1	2	Female	/fa/	2	Anger	Tone 2	In context	Correct	
1	1	Male	/fa/	1	Anger	Tone 2	In context	Correct	
1	1	Male	/fa/	2	Anger	Tone 2	In context	Correct	
1	2	Female	/pu/	1	Anger	Tone 2	In context	Correct	
1	2	Female	/pu/	2	Anger	Tone 2	In context	Correct	
1	1	Male	/pu/	1	Anger	Tone 2	In context	Correct	
1	1	Male	/pu/	2	Anger	Tone 2	In context	Correct	
1	2	Female	/ci/	1	Anger	Tone 3	In context	Correct	
1	2	Female	/ci/	2	Anger	Tone 3	In context	Correct	
1	1	Male	/ci/	1	Anger	Tone 3	In context	Correct	
1	1	Male	/ci/	2	Anger	Tone 3	In context	Correct	
1	2	Female	/fa/	1	Anger	Tone 3	In context	Correct	
1	2	Female	/fa/	2	Anger	Tone 3	In context	Correct	
1	1	Male	/fa/	1	Anger	Tone 3	In context	Correct	
1	1	Male	/fa/	2	Anger	Tone 3	In context	Correct	
1	2	Female	/pu/	1	Anger	Tone 3	In context	Correct	
1	2	Female	/pu/	2	Anger	Tone 3	In context	Correct	
1	1	Male	/pu/	1	Anger	Tone 3	In context	Correct	
1	1	Male	/pu/	2	Anger	Tone 3	In context	Correct	
1	2	Female	/ci/	1	Anger	Tone 4	In context	Correct	
1	2	Female	/ci/	2	Anger	Tone 4	In context	Correct	
1	1	Male	/ci/	1	Anger	Tone 4	In context	Correct	
1	1	Male	/ci/	2	Anger	Tone 4	In context	Correct	
1	2	Female	/fa/	1	Anger	Tone 4	In context	Correct	
1	2	Female	/fa/	2	Anger	Tone 4	In context	Correct	
1	1	Male	/fa/	1	Anger	Tone 4	In context	Correct	
1	1	Male	/fa/	2	Anger	Tone 4	In context	Correct	
1	2	Female	/pu/	1	Anger	Tone 4	In context	Correct	
1	2	Female	/pu/	2	Anger	Tone 4	In context	Correct	
1	1	Male	/pu/	1	Anger	Tone 4	In context	Correct	
1	1	Male	/pu/	2	Anger	Tone 4	In context	Correct	
1	2	Female	/ci/	1	Fear	Tone 1	In context	Correct	
1	2	Female	/ci/	2	Fear	Tone 1	In context	Correct	
1	1	Male	/ci/	1	Fear	Tone 1	In context	Correct	
1	1	Male	/ci/	2	Fear	Tone 1	In context	Correct	
1	2	Female	/fa/	1	Fear	Tone 1	In context	Correct	
1	2	Female	/fa/	2	Fear	Tone 1	In context	Correct	
1	1	Male	/fa/	1	Fear	Tone 1	In context	Correct	
1	1	Male	/fa/	2	Fear	Tone 1	In context	Correct	
1	2	Female	/pu/	1	Fear	Tone 1	In context	Correct	
1	2	Female	/pu/	2	Fear	Tone 1	In context	Correct	
1	1	Male	/pu/	1	Fear	Tone 1	In context	Correct	
1	1	Male	/pu/	2	Fear	Tone 1	In context	Correct	
1	2	Female	/ci/	1	Fear	Tone 2	In context	Correct	
1	2	Female	/ci/	2	Fear	Tone 2	In context	Correct	
1	1	Male	/ci/	1	Fear	Tone 2	In context	Correct	
1	1	Male	/ci/	2	Fear	Tone 2	In context	Correct	
1	2	Female	/fa/	1	Fear	Tone 2	In context	Correct	

1	2	Female
1	1	Male
1	1	Male
1	2	Female
1	2	Female
1	1	Male
1	1	Male
/fa/
/fa/
/fa/
/pu/
/pu/
/pu/
/pu/
2	Fear
Fear
Fear
Fear
Fear
Fear
Fear
Tone 2 In context Tone 2 In context Tone 2 In context Tone 2 In context Tone 2 In context Tone 2 In context Tone 2 In context
Correct Correct Correct Correct Correct Correct Correct

1	2	Female
/ci/
Fear
Tone 3 In context Incorrect

1	2	Female
1	1	Male
1	1	Male
1	2	Female
1	2	Female
1	1	Male
1	1	Male
1	2	Female
1	2	Female
1	1	Male
1	1	Male
1	2	Female
1	2	Female
1	1	Male
1	1	Male
1	2	Female
1	2	Female
1	1	Male
1	1	Male
1	2	Female
1	2	Female
1	1	Male
1	1	Male
/ci/
/ci/
/ci/
/fa/
/fa/
/fa/
/fa/
/pu/
/pu/
/pu/
/pu/
/ci/
/ci/
/ci/
/ci/
/fa/
/fa/
/fa/
/fa/
/pu/
/pu/
/pu/
/pu/
Fear
Fear
Fear
Fear
Fear
Fear
Fear
Fear
Fear
Fear
Fear
Fear
Fear
Fear
Fear
Fear
Fear
Fear
Fear
Fear
Fear
Fear
Fear
Tone 3 In context Tone 3 In context Tone 3 In context Tone 3 In context Tone 3 In context Tone 3 In context Tone 3 In context Tone 3 In context Tone 3 In context Tone 3 In context Tone 3 In context Tone 4 In context Tone 4 In context Tone 4 In context Tone 4 In context Tone 4 In context Tone 4 In context Tone 4 In context Tone 4 In context Tone 4 In context Tone 4 In context Tone 4 In context Tone 4 In context
Correct Correct Correct Correct Correct Correct Correct Correct Correct Correct Correct Correct Correct Correct Correct Correct Correct Correct Correct Correct Correct Correct Correct

1	2	Female
1	2	Female
1	1	Male
/ci/
/ci/
/ci/
HappinesTone 1 In context
HappinesTone 1 In context
HappinesTone 1 In context
Correct
Correct Correct

1	1	Male
/ci/
HappinesTone 1 In context Incorrect

1	2	Female
1	2	Female
1	1	Male
1	1	Male
1	2	Female
1	2	Female
1	1	Male
/fa/
/fa/
/fa/
/fa/
/pu/
/pu/
/pu/
HappinesTone 1 In context
HappinesTone 1 In context
HappinesTone 1 In context
HappinesTone 1 In context
HappinesTone 1 In context
HappinesTone 1 In context
HappinesTone 1 In context
Correct Correct Correct Correct Correct Correct Correct

1	1	Male
/pu/
HappinesTone 1 In context Incorrect

1	2	Female
1	2	Female
1	1	Male
1	1	Male
1	2	Female
1	2	Female
1	1	Male
1	1	Male
/ci/
/ci/
/ci/
/ci/
/fa/
/fa/
/fa/
/fa/
HappinesTone 2 In context
HappinesTone 2 In context
HappinesTone 2 In context
HappinesTone 2 In context
HappinesTone 2 In context
HappinesTone 2 In context
HappinesTone 2 In context
HappinesTone 2 In context
Correct Correct Correct Correct Correct Correct Correct Correct

1	2	Female
1	2	Female
1	1	Male
1	1	Male
1	2	Female
1	2	Female
1	1	Male
1	1	Male
1	2	Female
1	2	Female
1	1	Male
1	1	Male
1	2	Female
1	2	Female
1	1	Male
1	1	Male
1	2	Female
1	2	Female
1	1	Male
1	1	Male
1	2	Female
1	2	Female
1	1	Male
1	1	Male
/pu/
/pu/
/pu/
/pu/
/ci/
/ci/
/ci/
/ci/
/fa/
/fa/
/fa/
/fa/
/pu/
/pu/
/pu/
/pu/
/ci/
/ci/
/ci/
/ci/
/fa/
/fa/
/fa/
/fa/
HappinesTone 2 In context
HappinesTone 2 In context
HappinesTone 2 In context
HappinesTone 2 In context
HappinesTone 3 In context
HappinesTone 3 In context
HappinesTone 3 In context
HappinesTone 3 In context
HappinesTone 3 In context
HappinesTone 3 In context
HappinesTone 3 In context
HappinesTone 3 In context
HappinesTone 3 In context
HappinesTone 3 In context
HappinesTone 3 In context
HappinesTone 3 In context
HappinesTone 4 In context
HappinesTone 4 In context
HappinesTone 4 In context
HappinesTone 4 In context
HappinesTone 4 In context
HappinesTone 4 In context
HappinesTone 4 In context
HappinesTone 4 In context
Correct Correct Correct Correct Correct Correct Correct Correct Correct Correct Correct Correct Correct Correct Correct Correct Correct Correct Correct Correct Correct Correct Correct Correct

1	2	Female
/pu/
HappinesTone 4 In context Incorrect

1	2	Female
1	1	Male
1	1	Male
/pu/
/pu/
/pu/
HappinesTone 4 In context
HappinesTone 4 In context
HappinesTone 4 In context
Correct Correct Correct

1	2	Female
1	2	Female
1	1	Male
1	1	Male
1	2	Female
1	2	Female
1	1	Male
1	1	Male
1	2	Female
1	2	Female
1	1	Male
1	1	Male
1	2	Female
1	2	Female
1	1	Male
1	1	Male
1	2	Female
1	2	Female
1	1	Male
1	1	Male
1	2	Female
1	2	Female
1	1	Male
/ci/
/ci/
/ci/
/ci/
/fa/
/fa/
/fa/
/fa/
/pu/
/pu/
/pu/
/pu/
/ci/
/ci/
/ci/
/ci/
/fa/
/fa/
/fa/
/fa/
/pu/
/pu/
/pu/
Neutral Tone 1 In context
Neutral Tone 1 In context
Neutral Tone 1 In context
Neutral Tone 1 In context
Neutral Tone 1 In context
Neutral Tone 1 In context
Neutral Tone 1 In context
Neutral Tone 1 In context
Neutral Tone 1 In context
Neutral Tone 1 In context
Neutral Tone 1 In context
Neutral Tone 1 In context
Neutral Tone 2 In context
Neutral Tone 2 In context
Neutral Tone 2 In context
Neutral Tone 2 In context
Neutral Tone 2 In context
Neutral Tone 2 In context
Neutral Tone 2 In context
Neutral Tone 2 In context
Neutral Tone 2 In context
Neutral Tone 2 In context
1	Neutral Tone 2 In context
Correct Correct Correct Correct Correct Correct Correct Correct Correct Correct Correct Correct Correct Correct Correct Correct Correct Correct Correct Correct Correct Correct Correct

1	1	Male	/pu/	2	Neutral	Tone 2	In context	Correct	
1	2	Female	/ci/	1	Neutral	Tone 3	In context	Correct	
1	2	Female	/ci/	2	Neutral	Tone 3	In context	Correct	
1	1	Male	/ci/	1	Neutral	Tone 3	In context	Correct	
1	1	Male	/ci/	2	Neutral	Tone 3	In context	Correct	
1	2	Female	/fa/	1	Neutral	Tone 3	In context	Correct	
1	2	Female	/fa/	2	Neutral	Tone 3	In context	Correct	
1	1	Male	/fa/	1	Neutral	Tone 3	In context	Correct	
1	1	Male	/fa/	2	Neutral	Tone 3	In context	Correct	
1	2	Female	/pu/	1	Neutral	Tone 3	In context	Correct	
1	2	Female	/pu/	2	Neutral	Tone 3	In context	Correct	
1	1	Male	/pu/	1	Neutral	Tone 3	In context	Correct	
1	1	Male	/pu/	2	Neutral	Tone 3	In context	Correct	
1	2	Female	/ci/	1	Neutral	Tone 4	In context	Correct	
1	2	Female	/ci/	2	Neutral	Tone 4	In context	Correct	
1	1	Male	/ci/	1	Neutral	Tone 4	In context	Correct	
1	1	Male	/ci/	2	Neutral	Tone 4	In context	Correct	
1	2	Female	/fa/	1	Neutral	Tone 4	In context	Correct	
1	2	Female	/fa/	2	Neutral	Tone 4	In context	Correct	
1	1	Male	/fa/	1	Neutral	Tone 4	In context	Correct	
1	1	Male	/fa/	2	Neutral	Tone 4	In context	Correct	
1	2	Female	/pu/	1	Neutral	Tone 4	In context	Correct	
1	2	Female	/pu/	2	Neutral	Tone 4	In context	Correct	
1	1	Male	/pu/	1	Neutral	Tone 4	In context	Correct	
1	1	Male	/pu/	2	Neutral	Tone 4	In context	Correct	
1	2	Female	/ci/	1	Sadness	Tone 1	In context	Correct	
1	2	Female	/ci/	2	Sadness	Tone 1	In context	Correct	
1	1	Male	/ci/	1	Sadness	Tone 1	In context	Correct	
1	1	Male	/ci/	2	Sadness	Tone 1	In context	Correct	
1	2	Female	/fa/	1	Sadness	Tone 1	In context	Correct	
1	2	Female	/fa/	2	Sadness	Tone 1	In context	Correct	
1	1	Male	/fa/	1	Sadness	Tone 1	In context	Correct	
1	1	Male	/fa/	2	Sadness	Tone 1	In context	Correct	
1	2	Female	/pu/	1	Sadness	Tone 1	In context	Correct	
1	2	Female	/pu/	2	Sadness	Tone 1	In context	Correct	
1	1	Male	/pu/	1	Sadness	Tone 1	In context	Correct	
1	1	Male	/pu/	2	Sadness	Tone 1	In context	Correct	
1	2	Female	/ci/	1	Sadness	Tone 2	In context	Correct	
1	2	Female	/ci/	2	Sadness	Tone 2	In context	Correct	
1	1	Male	/ci/	1	Sadness	Tone 2	In context	Correct	
1	1	Male	/ci/	2	Sadness	Tone 2	In context	Correct	
1	2	Female	/fa/	1	Sadness	Tone 2	In context	Correct	
1	2	Female	/fa/	2	Sadness	Tone 2	In context	Correct	
1	1	Male	/fa/	1	Sadness	Tone 2	In context	Correct	
1	1	Male	/fa/	2	Sadness	Tone 2	In context	Correct	
1	2	Female	/pu/	1	Sadness	Tone 2	In context	Correct	
1	2	Female	/pu/	2	Sadness	Tone 2	In context	Correct	
1	1	Male	/pu/	1	Sadness	Tone 2	In context	Correct	
1	1	Male	/pu/	2	Sadness	Tone 2	In context	Correct	
1	2	Female	/ci/	1	Sadness	Tone 3	In context	Correct	
1	2	Female	/ci/	2	Sadness	Tone 3	In context	Correct	

1	1	Male	/ci/	1	Sadness	Tone 3	In context	Correct	
1	1	Male	/ci/	2	Sadness	Tone 3	In context	Correct	
1	2	Female	/fa/	1	Sadness	Tone 3	In context	Correct	
1	2	Female	/fa/	2	Sadness	Tone 3	In context	Correct	
1	1	Male	/fa/	1	Sadness	Tone 3	In context	Correct	
1	1	Male	/fa/	2	Sadness	Tone 3	In context	Correct	
1	2	Female	/pu/	1	Sadness	Tone 3	In context	Correct	
1	2	Female	/pu/	2	Sadness	Tone 3	In context	Correct	
1	1	Male	/pu/	1	Sadness	Tone 3	In context	Correct	
1	1	Male	/pu/	2	Sadness	Tone 3	In context	Correct	
1	2	Female	/ci/	1	Sadness	Tone 4	In context	Correct	
1	2	Female	/ci/	2	Sadness	Tone 4	In context	Correct	
1	1	Male	/ci/	1	Sadness	Tone 4	In context	Correct	
1	1	Male	/ci/	2	Sadness	Tone 4	In context	Correct	
1	2	Female	/fa/	1	Sadness	Tone 4	In context	Correct	
1	2	Female	/fa/	2	Sadness	Tone 4	In context	Correct	
1	1	Male	/fa/	1	Sadness	Tone 4	In context	Correct	
1	1	Male	/fa/	2	Sadness	Tone 4	In context	Correct	
1	2	Female	/pu/	1	Sadness	Tone 4	In context	Correct	
1	2	Female	/pu/	2	Sadness	Tone 4	In context	Incorrect	
1	1	Male	/pu/	1	Sadness	Tone 4	In context	Correct	
1	1	Male	/pu/	2	Sadness	Tone 4	In context	Correct	
2	2	Female	/ci/	1	Anger	Tone 1	In isolation	Correct	
2	2	Female	/ci/	2	Anger	Tone 1	In isolation	Incorrect	
2	1	Male	/ci/	1	Anger	Tone 1	In isolation	Correct	
2	1	Male	/ci/	2	Anger	Tone 1	In isolation	Correct	
2	2	Female	/fa/	1	Anger	Tone 1	In isolation	Incorrect	
2	2	Female	/fa/	2	Anger	Tone 1	In isolation	Correct	
2	1	Male	/fa/	1	Anger	Tone 1	In isolation	Correct	
2	1	Male	/fa/	2	Anger	Tone 1	In isolation	Correct	
2	2	Female	/pu/	1	Anger	Tone 1	In isolation	Correct	
2	2	Female	/pu/	2	Anger	Tone 1	In isolation	Correct	
2	1	Male	/pu/	1	Anger	Tone 1	In isolation	Correct	
2	1	Male	/pu/	2	Anger	Tone 1	In isolation	Incorrect	
2	2	Female	/ci/	1	Anger	Tone 2	In isolation	Incorrect	
2	2	Female	/ci/	2	Anger	Tone 2	In isolation	Incorrect	
2	1	Male	/ci/	1	Anger	Tone 2	In isolation	Incorrect	
2	1	Male	/ci/	2	Anger	Tone 2	In isolation	Correct	
2	2	Female	/fa/	1	Anger	Tone 2	In isolation	Correct	
2	2	Female	/fa/	2	Anger	Tone 2	In isolation	Correct	
2	1	Male	/fa/	1	Anger	Tone 2	In isolation	Correct	
2	1	Male	/fa/	2	Anger	Tone 2	In isolation	Correct	
2	2	Female	/pu/	1	Anger	Tone 2	In isolation	Correct	
2	2	Female	/pu/	2	Anger	Tone 2	In isolation	Correct	
2	1	Male	/pu/	1	Anger	Tone 2	In isolation	Incorrect	
2	1	Male	/pu/	2	Anger	Tone 2	In isolation	Incorrect	
2	2	Female	/ci/	1	Anger	Tone 3	In isolation	Incorrect	
2	2	Female	/ci/	2	Anger	Tone 3	In isolation	Incorrect	
2	1	Male	/ci/	1	Anger	Tone 3	In isolation	Incorrect	
2	1	Male	/ci/	2	Anger	Tone 3	In isolation	Correct	
2	2	Female	/fa/	1	Anger	Tone 3	In isolation	Correct	

2	2	Female	/fa/	2	Anger	Tone 3	In isolation	Incorrect	
2	1	Male	/fa/	1	Anger	Tone 3	In isolation	Incorrect	
2	1	Male	/fa/	2	Anger	Tone 3	In isolation	Correct	
2	2	Female	/pu/	1	Anger	Tone 3	In isolation	Incorrect	
2	2	Female	/pu/	2	Anger	Tone 3	In isolation	Correct	
2	1	Male	/pu/	1	Anger	Tone 3	In isolation	Correct	
2	1	Male	/pu/	2	Anger	Tone 3	In isolation	Correct	
2	2	Female	/ci/	1	Anger	Tone 4	In isolation	Correct	
2	2	Female	/ci/	2	Anger	Tone 4	In isolation	Correct	
2	1	Male	/ci/	1	Anger	Tone 4	In isolation	Correct	
2	1	Male	/ci/	2	Anger	Tone 4	In isolation	Correct	
2	2	Female	/fa/	1	Anger	Tone 4	In isolation	Correct	
2	2	Female	/fa/	2	Anger	Tone 4	In isolation	Correct	
2	1	Male	/fa/	1	Anger	Tone 4	In isolation	Correct	
2	1	Male	/fa/	2	Anger	Tone 4	In isolation	Correct	
2	2	Female	/pu/	1	Anger	Tone 4	In isolation	Incorrect	
2	2	Female	/pu/	2	Anger	Tone 4	In isolation	Correct	
2	1	Male	/pu/	1	Anger	Tone 4	In isolation	Correct	
2	1	Male	/pu/	2	Anger	Tone 4	In isolation	Correct	
2	2	Female	/ci/	1	Fear	Tone 1	In isolation	Correct	
2	2	Female	/ci/	2	Fear	Tone 1	In isolation	Incorrect	
2	1	Male	/ci/	1	Fear	Tone 1	In isolation	Correct	
2	1	Male	/ci/	2	Fear	Tone 1	In isolation	Correct	
2	2	Female	/fa/	1	Fear	Tone 1	In isolation	Correct	
2	2	Female	/fa/	2	Fear	Tone 1	In isolation	Correct	
2	1	Male	/fa/	1	Fear	Tone 1	In isolation	Correct	
2	1	Male	/fa/	2	Fear	Tone 1	In isolation	Correct	
2	2	Female	/pu/	1	Fear	Tone 1	In isolation	Incorrect	
2	2	Female	/pu/	2	Fear	Tone 1	In isolation	Correct	
2	1	Male	/pu/	1	Fear	Tone 1	In isolation	Correct	
2	1	Male	/pu/	2	Fear	Tone 1	In isolation	Correct	
2	2	Female	/ci/	1	Fear	Tone 2	In isolation	Incorrect	
2	2	Female	/ci/	2	Fear	Tone 2	In isolation	Incorrect	
2	1	Male	/ci/	1	Fear	Tone 2	In isolation	Correct	
2	1	Male	/ci/	2	Fear	Tone 2	In isolation	Correct	
2	2	Female	/fa/	1	Fear	Tone 2	In isolation	Correct	
2	2	Female	/fa/	2	Fear	Tone 2	In isolation	Correct	
2	1	Male	/fa/	1	Fear	Tone 2	In isolation	Incorrect	
2	1	Male	/fa/	2	Fear	Tone 2	In isolation	Correct	
2	2	Female	/pu/	1	Fear	Tone 2	In isolation	Correct	
2	2	Female	/pu/	2	Fear	Tone 2	In isolation	Incorrect	
2	1	Male	/pu/	1	Fear	Tone 2	In isolation	Incorrect	
2	1	Male	/pu/	2	Fear	Tone 2	In isolation	Incorrect	
2	2	Female	/ci/	1	Fear	Tone 3	In isolation	Correct	
2	2	Female	/ci/	2	Fear	Tone 3	In isolation	Incorrect	
2	1	Male	/ci/	1	Fear	Tone 3	In isolation	Correct	
2	1	Male	/ci/	2	Fear	Tone 3	In isolation	Correct	
2	2	Female	/fa/	1	Fear	Tone 3	In isolation	Incorrect	
2	2	Female	/fa/	2	Fear	Tone 3	In isolation	Incorrect	
2	1	Male	/fa/	1	Fear	Tone 3	In isolation	Correct	
2	1	Male	/fa/	2	Fear	Tone 3	In isolation	Correct	

2	2	Female
2	2	Female
2	1	Male
2	1	Male
2	2	Female
2	2	Female
2	1	Male
2	1	Male
2	2	Female
2	2	Female
2	1	Male
2	1	Male
2	2	Female
2	2	Female
2	1	Male
2	1	Male
/pu/
/pu/
/pu/
/pu/
/ci/
/ci/
/ci/
/ci/
/fa/
/fa/
/fa/
/fa/
/pu/
/pu/
/pu/
/pu/
Fear
Fear
Fear
Fear
Fear
Fear
Fear
Fear
Fear
Fear
Fear
Fear
Fear
Fear
Fear
Fear
Tone 3In isolation Incorrect Tone 3In isolation Incorrect Tone 3In isolation Incorrect Tone 3In isolation Incorrect Tone 4In isolation Incorrect Tone 4In isolation Correct Tone 4In isolation Correct Tone 4In isolation Correct Tone 4In isolation Correct Tone 4In isolation Correct Tone 4In isolation Correct Tone 4In isolation Correct Tone 4In isolation Correct Tone 4In isolation Incorrect Tone 4In isolation Incorrect Tone 4In isolation Correct

2	2	Female
2	2	Female
2	1	Male
2	1	Male
2	2	Female
2	2	Female
2	1	Male
2	1	Male
2	2	Female
2	2	Female
2	1	Male
2	1	Male
2	2	Female
2	2	Female
2	1	Male
2	1	Male
2	2	Female
2	2	Female
2	1	Male
2	1	Male
2	2	Female
2	2	Female
2	1	Male
2	1	Male
2	2	Female
2	2	Female
2	1	Male
2	1	Male
2	2	Female
2	2	Female
2	1	Male
2	1	Male
2	2	Female
2	2	Female
2	1	Male
/ci/
/ci/
/ci/
/ci/
/fa/
/fa/
/fa/
/fa/
/pu/
/pu/
/pu/
/pu/
/ci/
/ci/
/ci/
/ci/
/fa/
/fa/
/fa/
/fa/
/pu/
/pu/
/pu/
/pu/
/ci/
/ci/
/ci/
/ci/
/fa/
/fa/
/fa/
/fa/
/pu/
/pu/
/pu/
HappinesTone 1In isolation Correct
HappinesTone 1In isolation Incorrect
HappinesTone 1In isolation Correct
HappinesTone 1In isolation Correct
HappinesTone 1In isolation Incorrect
HappinesTone 1In isolation Incorrect
HappinesTone 1In isolation Correct
HappinesTone 1In isolation Correct
HappinesTone 1In isolation Incorrect
HappinesTone 1In isolation Correct
HappinesTone 1In isolation Correct
HappinesTone 1In isolation Correct
HappinesTone 2In isolation Incorrect
HappinesTone 2In isolation Correct
HappinesTone 2In isolation Correct
HappinesTone 2In isolation Correct
HappinesTone 2In isolation Incorrect
HappinesTone 2In isolation Correct
HappinesTone 2In isolation Incorrect
HappinesTone 2In isolation Correct
HappinesTone 2In isolation Correct
HappinesTone 2In isolation Incorrect
HappinesTone 2In isolation Correct
HappinesTone 2In isolation Correct
HappinesTone 3In isolation Correct
HappinesTone 3In isolation Incorrect
HappinesTone 3In isolation Correct
HappinesTone 3In isolation Correct
HappinesTone 3In isolation Correct
HappinesTone 3In isolation Correct
HappinesTone 3In isolation Correct
HappinesTone 3In isolation Correct
HappinesTone 3In isolation Correct
HappinesTone 3In isolation Incorrect
HappinesTone 3In isolation Correct

2	1	Male
2	2	Female
2	2	Female
2	1	Male
2	1	Male
2	2	Female
2	2	Female
2	1	Male
2	1	Male
2	2	Female
2	2	Female
2	1	Male
2	1	Male
/pu/
/ci/
/ci/
/ci/
/ci/
/fa/
/fa/
/fa/
/fa/
/pu/
/pu/
/pu/
/pu/
HappinesTone 3In isolation Incorrect
HappinesTone 4In isolation Incorrect
HappinesTone 4In isolation Incorrect
HappinesTone 4In isolation Correct
HappinesTone 4In isolation Correct
HappinesTone 4In isolation Correct
HappinesTone 4In isolation Correct
HappinesTone 4In isolation Correct
HappinesTone 4In isolation Correct
HappinesTone 4In isolation Incorrect
HappinesTone 4In isolation Incorrect
HappinesTone 4In isolation Correct
HappinesTone 4In isolation Correct

2	2	Female
2	2	Female
2	1	Male
2	1	Male
2	2	Female
2	2	Female
2	1	Male
2	1	Male
2	2	Female
2	2	Female
2	1	Male
2	1	Male
2	2	Female
2	2	Female
2	1	Male
2	1	Male
2	2	Female
2	2	Female
2	1	Male
2	1	Male
2	2	Female
2	2	Female
2	1	Male
2	1	Male
2	2	Female
2	2	Female
2	1	Male
2	1	Male
2	2	Female
2	2	Female
2	1	Male
2	1	Male
2	2	Female
2	2	Female
2	1	Male
2	1	Male
2	2	Female
2	2	Female
/ci/
/ci/
/ci/
/ci/
/fa/
/fa/
/fa/
/fa/
/pu/
/pu/
/pu/
/pu/
/ci/
/ci/
/ci/
/ci/
/fa/
/fa/
/fa/
/fa/
/pu/
/pu/
/pu/
/pu/
/ci/
/ci/
/ci/
/ci/
/fa/
/fa/
/fa/
/fa/
/pu/
/pu/
/pu/
/pu/
/ci/
/ci/
Neutral Tone 1In isolation Correct
Neutral Tone 1In isolation Correct
Neutral Tone 1In isolation Correct
Neutral Tone 1In isolation Correct
Neutral Tone 1In isolation Correct
Neutral Tone 1In isolation Correct
Neutral Tone 1In isolation Correct
Neutral Tone 1In isolation Correct
Neutral Tone 1In isolation Correct
Neutral Tone 1In isolation Correct
Neutral Tone 1In isolation Correct
Neutral Tone 1In isolation Correct
Neutral Tone 2In isolation Correct
Neutral Tone 2In isolation Correct
Neutral Tone 2In isolation Correct
Neutral Tone 2In isolation Correct
Neutral Tone 2In isolation Correct
Neutral Tone 2In isolation Correct
Neutral Tone 2In isolation Correct
Neutral Tone 2In isolation Correct
Neutral Tone 2In isolation Correct
Neutral Tone 2In isolation Correct
Neutral Tone 2In isolation Correct
Neutral Tone 2In isolation Correct
Neutral Tone 3In isolation Correct
Neutral Tone 3In isolation Correct
Neutral Tone 3In isolation Correct
Neutral Tone 3In isolation Correct
Neutral Tone 3In isolation Correct
Neutral Tone 3In isolation Correct
Neutral Tone 3In isolation Correct
Neutral Tone 3In isolation Correct
Neutral Tone 3In isolation Correct
Neutral Tone 3In isolation Correct
Neutral Tone 3In isolation Correct
Neutral Tone 3In isolation Correct
Neutral Tone 4In isolation Correct
Neutral Tone 4In isolation Correct

2	1	Male	/ci/	1	Neutral	Tone 4	In isolation	Correct	
2	1	Male	/ci/	2	Neutral	Tone 4	In isolation	Correct	
2	2	Female	/fa/	1	Neutral	Tone 4	In isolation	Correct	
2	2	Female	/fa/	2	Neutral	Tone 4	In isolation	Correct	
2	1	Male	/fa/	1	Neutral	Tone 4	In isolation	Correct	
2	1	Male	/fa/	2	Neutral	Tone 4	In isolation	Correct	
2	2	Female	/pu/	1	Neutral	Tone 4	In isolation	Correct	
2	2	Female	/pu/	2	Neutral	Tone 4	In isolation	Correct	
2	1	Male	/pu/	1	Neutral	Tone 4	In isolation	Correct	
2	1	Male	/pu/	2	Neutral	Tone 4	In isolation	Correct	
2	2	Female	/ci/	1	Sadness	Tone 1	In isolation	Correct	
2	2	Female	/ci/	2	Sadness	Tone 1	In isolation	Correct	
2	1	Male	/ci/	1	Sadness	Tone 1	In isolation	Correct	
2	1	Male	/ci/	2	Sadness	Tone 1	In isolation	Correct	
2	2	Female	/fa/	1	Sadness	Tone 1	In isolation	Correct	
2	2	Female	/fa/	2	Sadness	Tone 1	In isolation	Correct	
2	1	Male	/fa/	1	Sadness	Tone 1	In isolation	Correct	
2	1	Male	/fa/	2	Sadness	Tone 1	In isolation	Correct	
2	2	Female	/pu/	1	Sadness	Tone 1	In isolation	Correct	
2	2	Female	/pu/	2	Sadness	Tone 1	In isolation	Correct	
2	1	Male	/pu/	1	Sadness	Tone 1	In isolation	Correct	
2	1	Male	/pu/	2	Sadness	Tone 1	In isolation	Correct	
2	2	Female	/ci/	1	Sadness	Tone 2	In isolation	Incorrect	
2	2	Female	/ci/	2	Sadness	Tone 2	In isolation	Incorrect	
2	1	Male	/ci/	1	Sadness	Tone 2	In isolation	Incorrect	
2	1	Male	/ci/	2	Sadness	Tone 2	In isolation	Correct	
2	2	Female	/fa/	1	Sadness	Tone 2	In isolation	Correct	
2	2	Female	/fa/	2	Sadness	Tone 2	In isolation	Correct	
2	1	Male	/fa/	1	Sadness	Tone 2	In isolation	Incorrect	
2	1	Male	/fa/	2	Sadness	Tone 2	In isolation	Correct	
2	2	Female	/pu/	1	Sadness	Tone 2	In isolation	Correct	
2	2	Female	/pu/	2	Sadness	Tone 2	In isolation	Incorrect	
2	1	Male	/pu/	1	Sadness	Tone 2	In isolation	Incorrect	
2	1	Male	/pu/	2	Sadness	Tone 2	In isolation	Incorrect	
2	2	Female	/ci/	1	Sadness	Tone 3	In isolation	Incorrect	
2	2	Female	/ci/	2	Sadness	Tone 3	In isolation	Incorrect	
2	1	Male	/ci/	1	Sadness	Tone 3	In isolation	Correct	
2	1	Male	/ci/	2	Sadness	Tone 3	In isolation	Correct	
2	2	Female	/fa/	1	Sadness	Tone 3	In isolation	Correct	
2	2	Female	/fa/	2	Sadness	Tone 3	In isolation	Incorrect	
2	1	Male	/fa/	1	Sadness	Tone 3	In isolation	Correct	
2	1	Male	/fa/	2	Sadness	Tone 3	In isolation	Incorrect	
2	2	Female	/pu/	1	Sadness	Tone 3	In isolation	Correct	
2	2	Female	/pu/	2	Sadness	Tone 3	In isolation	Correct	
2	1	Male	/pu/	1	Sadness	Tone 3	In isolation	Correct	
2	1	Male	/pu/	2	Sadness	Tone 3	In isolation	Correct	
2	2	Female	/ci/	1	Sadness	Tone 4	In isolation	Correct	
2	2	Female	/ci/	2	Sadness	Tone 4	In isolation	Correct	
2	1	Male	/ci/	1	Sadness	Tone 4	In isolation	Correct	
2	1	Male	/ci/	2	Sadness	Tone 4	In isolation	Correct	
2	2	Female	/fa/	1	Sadness	Tone 4	In isolation	Incorrect	

2	2	Female	/fa/	2	Sadness	Tone 4	In isolation	Incorrect	
2	1	Male	/fa/	1	Sadness	Tone 4	In isolation	Incorrect	
2	1	Male	/fa/	2	Sadness	Tone 4	In isolation	Incorrect	
2	2	Female	/pu/	1	Sadness	Tone 4	In isolation	Correct	
2	2	Female	/pu/	2	Sadness	Tone 4	In isolation	Incorrect	
2	1	Male	/pu/	1	Sadness	Tone 4	In isolation	Correct	
2	1	Male	/pu/	2	Sadness	Tone 4	In isolation	Incorrect	
2	2	Female	/ci/	1	Anger	Tone 1	In context	Correct	
2	2	Female	/ci/	2	Anger	Tone 1	In context	Correct	
2	1	Male	/ci/	1	Anger	Tone 1	In context	Correct	
2	1	Male	/ci/	2	Anger	Tone 1	In context	Correct	
2	2	Female	/fa/	1	Anger	Tone 1	In context	Correct	
2	2	Female	/fa/	2	Anger	Tone 1	In context	Correct	
2	1	Male	/fa/	1	Anger	Tone 1	In context	Correct	
2	1	Male	/fa/	2	Anger	Tone 1	In context	Correct	
2	2	Female	/pu/	1	Anger	Tone 1	In context	Incorrect	
2	2	Female	/pu/	2	Anger	Tone 1	In context	Incorrect	
2	1	Male	/pu/	1	Anger	Tone 1	In context	Correct	
2	1	Male	/pu/	2	Anger	Tone 1	In context	Correct	
2	2	Female	/ci/	1	Anger	Tone 2	In context	Correct	
2	2	Female	/ci/	2	Anger	Tone 2	In context	Correct	
2	1	Male	/ci/	1	Anger	Tone 2	In context	Correct	
2	1	Male	/ci/	2	Anger	Tone 2	In context	Correct	
2	2	Female	/fa/	1	Anger	Tone 2	In context	Correct	
2	2	Female	/fa/	2	Anger	Tone 2	In context	Correct	
2	1	Male	/fa/	1	Anger	Tone 2	In context	Correct	
2	1	Male	/fa/	2	Anger	Tone 2	In context	Correct	
2	2	Female	/pu/	1	Anger	Tone 2	In context	Correct	
2	2	Female	/pu/	2	Anger	Tone 2	In context	Correct	
2	1	Male	/pu/	1	Anger	Tone 2	In context	Correct	
2	1	Male	/pu/	2	Anger	Tone 2	In context	Correct	
2	2	Female	/ci/	1	Anger	Tone 3	In context	Correct	
2	2	Female	/ci/	2	Anger	Tone 3	In context	Correct	
2	1	Male	/ci/	1	Anger	Tone 3	In context	Correct	
2	1	Male	/ci/	2	Anger	Tone 3	In context	Correct	
2	2	Female	/fa/	1	Anger	Tone 3	In context	Correct	
2	2	Female	/fa/	2	Anger	Tone 3	In context	Correct	
2	1	Male	/fa/	1	Anger	Tone 3	In context	Correct	
2	1	Male	/fa/	2	Anger	Tone 3	In context	Correct	
2	2	Female	/pu/	1	Anger	Tone 3	In context	Correct	
2	2	Female	/pu/	2	Anger	Tone 3	In context	Correct	
2	1	Male	/pu/	1	Anger	Tone 3	In context	Correct	
2	1	Male	/pu/	2	Anger	Tone 3	In context	Correct	
2	2	Female	/ci/	1	Anger	Tone 4	In context	Correct	
2	2	Female	/ci/	2	Anger	Tone 4	In context	Correct	
2	1	Male	/ci/	1	Anger	Tone 4	In context	Correct	
2	1	Male	/ci/	2	Anger	Tone 4	In context	Correct	
2	2	Female	/fa/	1	Anger	Tone 4	In context	Correct	
2	2	Female	/fa/	2	Anger	Tone 4	In context	Correct	
2	1	Male	/fa/	1	Anger	Tone 4	In context	Correct	
2	1	Male	/fa/	2	Anger	Tone 4	In context	Correct	

2	2	Female	/pu/	1	Anger	Tone 4	In context	Correct	
2	2	Female	/pu/	2	Anger	Tone 4	In context	Correct	
2	1	Male	/pu/	1	Anger	Tone 4	In context	Correct	
2	1	Male	/pu/	2	Anger	Tone 4	In context	Correct	
2	2	Female	/ci/	1	Fear	Tone 1	In context	Correct	
2	2	Female	/ci/	2	Fear	Tone 1	In context	Correct	
2	1	Male	/ci/	1	Fear	Tone 1	In context	Correct	
2	1	Male	/ci/	2	Fear	Tone 1	In context	Correct	
2	2	Female	/fa/	1	Fear	Tone 1	In context	Correct	
2	2	Female	/fa/	2	Fear	Tone 1	In context	Correct	
2	1	Male	/fa/	1	Fear	Tone 1	In context	Correct	
2	1	Male	/fa/	2	Fear	Tone 1	In context	Correct	
2	2	Female	/pu/	1	Fear	Tone 1	In context	Correct	
2	2	Female	/pu/	2	Fear	Tone 1	In context	Correct	
2	1	Male	/pu/	1	Fear	Tone 1	In context	Correct	
2	1	Male	/pu/	2	Fear	Tone 1	In context	Correct	
2	2	Female	/ci/	1	Fear	Tone 2	In context	Correct	
2	2	Female	/ci/	2	Fear	Tone 2	In context	Correct	
2	1	Male	/ci/	1	Fear	Tone 2	In context	Correct	
2	1	Male	/ci/	2	Fear	Tone 2	In context	Correct	
2	2	Female	/fa/	1	Fear	Tone 2	In context	Correct	
2	2	Female	/fa/	2	Fear	Tone 2	In context	Correct	
2	1	Male	/fa/	1	Fear	Tone 2	In context	Correct	
2	1	Male	/fa/	2	Fear	Tone 2	In context	Correct	
2	2	Female	/pu/	1	Fear	Tone 2	In context	Correct	
2	2	Female	/pu/	2	Fear	Tone 2	In context	Correct	
2	1	Male	/pu/	1	Fear	Tone 2	In context	Correct	
2	1	Male	/pu/	2	Fear	Tone 2	In context	Correct	
2	2	Female	/ci/	1	Fear	Tone 3	In context	Incorrect	
2	2	Female	/ci/	2	Fear	Tone 3	In context	Correct	
2	1	Male	/ci/	1	Fear	Tone 3	In context	Correct	
2	1	Male	/ci/	2	Fear	Tone 3	In context	Correct	
2	2	Female	/fa/	1	Fear	Tone 3	In context	Correct	
2	2	Female	/fa/	2	Fear	Tone 3	In context	Correct	
2	1	Male	/fa/	1	Fear	Tone 3	In context	Correct	
2	1	Male	/fa/	2	Fear	Tone 3	In context	Correct	
2	2	Female	/pu/	1	Fear	Tone 3	In context	Incorrect	
2	2	Female	/pu/	2	Fear	Tone 3	In context	Correct	
2	1	Male	/pu/	1	Fear	Tone 3	In context	Correct	
2	1	Male	/pu/	2	Fear	Tone 3	In context	Correct	
2	2	Female	/ci/	1	Fear	Tone 4	In context	Correct	
2	2	Female	/ci/	2	Fear	Tone 4	In context	Correct	
2	1	Male	/ci/	1	Fear	Tone 4	In context	Correct	
2	1	Male	/ci/	2	Fear	Tone 4	In context	Correct	
2	2	Female	/fa/	1	Fear	Tone 4	In context	Correct	
2	2	Female	/fa/	2	Fear	Tone 4	In context	Correct	
2	1	Male	/fa/	1	Fear	Tone 4	In context	Correct	
2	1	Male	/fa/	2	Fear	Tone 4	In context	Correct	
2	2	Female	/pu/	1	Fear	Tone 4	In context	Correct	
2	2	Female	/pu/	2	Fear	Tone 4	In context	Correct	
2	1	Male	/pu/	1	Fear	Tone 4	In context	Correct	

2	1	Male
/pu/
2	Fear
Tone 4 In context
Correct

2	2	Female
2	2	Female
2	1	Male
2	1	Male
2	2	Female
2	2	Female
2	1	Male
2	1	Male
2	2	Female
2	2	Female
2	1	Male
/ci/
/ci/
/ci/
/ci/
/fa/
/fa/
/fa/
/fa/
/pu/
/pu/
/pu/
HappinesTone 1 In context
HappinesTone 1 In context
HappinesTone 1 In context
HappinesTone 1 In context
HappinesTone 1 In context
HappinesTone 1 In context
HappinesTone 1 In context
HappinesTone 1 In context
HappinesTone 1 In context
HappinesTone 1 In context
HappinesTone 1 In context
Correct Correct Correct Correct Correct Correct Correct Correct Correct Correct Correct

2	1	Male
/pu/
HappinesTone 1 In context Incorrect

2	2	Female
2	2	Female
2	1	Male
2	1	Male
2	2	Female
2	2	Female
2	1	Male
2	1	Male
2	2	Female
2	2	Female
2	1	Male
2	1	Male
2	2	Female
2	2	Female
2	1	Male
2	1	Male
2	2	Female
2	2	Female
2	1	Male
2	1	Male
2	2	Female
2	2	Female
2	1	Male
2	1	Male
2	2	Female
2	2	Female
2	1	Male
2	1	Male
2	2	Female
2	2	Female
2	1	Male
2	1	Male
2	2	Female
2	2	Female
2	1	Male
2	1	Male
/ci/
/ci/
/ci/
/ci/
/fa/
/fa/
/fa/
/fa/
/pu/
/pu/
/pu/
/pu/
/ci/
/ci/
/ci/
/ci/
/fa/
/fa/
/fa/
/fa/
/pu/
/pu/
/pu/
/pu/
/ci/
/ci/
/ci/
/ci/
/fa/
/fa/
/fa/
/fa/
/pu/
/pu/
/pu/
/pu/
HappinesTone 2 In context
HappinesTone 2 In context
HappinesTone 2 In context
HappinesTone 2 In context
HappinesTone 2 In context
HappinesTone 2 In context
HappinesTone 2 In context
HappinesTone 2 In context
HappinesTone 2 In context
HappinesTone 2 In context
HappinesTone 2 In context
HappinesTone 2 In context
HappinesTone 3 In context
HappinesTone 3 In context
HappinesTone 3 In context
HappinesTone 3 In context
HappinesTone 3 In context
HappinesTone 3 In context
HappinesTone 3 In context
HappinesTone 3 In context
HappinesTone 3 In context
HappinesTone 3 In context
HappinesTone 3 In context
HappinesTone 3 In context
HappinesTone 4 In context
HappinesTone 4 In context
HappinesTone 4 In context
HappinesTone 4 In context
HappinesTone 4 In context
HappinesTone 4 In context
HappinesTone 4 In context
HappinesTone 4 In context
HappinesTone 4 In context
HappinesTone 4 In context
HappinesTone 4 In context
HappinesTone 4 In context
Correct Correct Correct Correct Correct Correct Correct Correct Correct Correct Correct Correct Correct Correct Correct Correct Correct Correct Correct Correct Correct Correct Correct Correct Correct Correct Correct Correct Correct Correct Correct Correct Correct Correct Correct Correct

2	2	Female
2	2	Female
/ci/
/ci/
Neutral Tone 1 In context
Neutral Tone 1 In context
Correct
Correct

2	1	Male	/ci/	1	Neutral	Tone 1	In context	Correct	
2	1	Male	/ci/	2	Neutral	Tone 1	In context	Correct	
2	2	Female	/fa/	1	Neutral	Tone 1	In context	Correct	
2	2	Female	/fa/	2	Neutral	Tone 1	In context	Correct	
2	1	Male	/fa/	1	Neutral	Tone 1	In context	Correct	
2	1	Male	/fa/	2	Neutral	Tone 1	In context	Correct	
2	2	Female	/pu/	1	Neutral	Tone 1	In context	Correct	
2	2	Female	/pu/	2	Neutral	Tone 1	In context	Correct	
2	1	Male	/pu/	1	Neutral	Tone 1	In context	Correct	
2	1	Male	/pu/	2	Neutral	Tone 1	In context	Correct	
2	2	Female	/ci/	1	Neutral	Tone 2	In context	Correct	
2	2	Female	/ci/	2	Neutral	Tone 2	In context	Correct	
2	1	Male	/ci/	1	Neutral	Tone 2	In context	Correct	
2	1	Male	/ci/	2	Neutral	Tone 2	In context	Correct	
2	2	Female	/fa/	1	Neutral	Tone 2	In context	Correct	
2	2	Female	/fa/	2	Neutral	Tone 2	In context	Correct	
2	1	Male	/fa/	1	Neutral	Tone 2	In context	Correct	
2	1	Male	/fa/	2	Neutral	Tone 2	In context	Correct	
2	2	Female	/pu/	1	Neutral	Tone 2	In context	Correct	
2	2	Female	/pu/	2	Neutral	Tone 2	In context	Correct	
2	1	Male	/pu/	1	Neutral	Tone 2	In context	Correct	
2	1	Male	/pu/	2	Neutral	Tone 2	In context	Correct	
2	2	Female	/ci/	1	Neutral	Tone 3	In context	Correct	
2	2	Female	/ci/	2	Neutral	Tone 3	In context	Correct	
2	1	Male	/ci/	1	Neutral	Tone 3	In context	Correct	
2	1	Male	/ci/	2	Neutral	Tone 3	In context	Correct	
2	2	Female	/fa/	1	Neutral	Tone 3	In context	Correct	
2	2	Female	/fa/	2	Neutral	Tone 3	In context	Correct	
2	1	Male	/fa/	1	Neutral	Tone 3	In context	Correct	
2	1	Male	/fa/	2	Neutral	Tone 3	In context	Correct	
2	2	Female	/pu/	1	Neutral	Tone 3	In context	Correct	
2	2	Female	/pu/	2	Neutral	Tone 3	In context	Correct	
2	1	Male	/pu/	1	Neutral	Tone 3	In context	Correct	
2	1	Male	/pu/	2	Neutral	Tone 3	In context	Correct	
2	2	Female	/ci/	1	Neutral	Tone 4	In context	Correct	
2	2	Female	/ci/	2	Neutral	Tone 4	In context	Correct	
2	1	Male	/ci/	1	Neutral	Tone 4	In context	Correct	
2	1	Male	/ci/	2	Neutral	Tone 4	In context	Correct	
2	2	Female	/fa/	1	Neutral	Tone 4	In context	Correct	
2	2	Female	/fa/	2	Neutral	Tone 4	In context	Correct	
2	1	Male	/fa/	1	Neutral	Tone 4	In context	Correct	
2	1	Male	/fa/	2	Neutral	Tone 4	In context	Correct	
2	2	Female	/pu/	1	Neutral	Tone 4	In context	Correct	
2	2	Female	/pu/	2	Neutral	Tone 4	In context	Correct	
2	1	Male	/pu/	1	Neutral	Tone 4	In context	Correct	
2	1	Male	/pu/	2	Neutral	Tone 4	In context	Correct	
2	2	Female	/ci/	1	Sadness	Tone 1	In context	Correct	
2	2	Female	/ci/	2	Sadness	Tone 1	In context	Correct	
2	1	Male	/ci/	1	Sadness	Tone 1	In context	Correct	
2	1	Male	/ci/	2	Sadness	Tone 1	In context	Correct	
2	2	Female	/fa/	1	Sadness	Tone 1	In context	Correct	

2	2	Female	/fa/	2	Sadness	Tone 1	In context	Correct	
2	1	Male	/fa/	1	Sadness	Tone 1	In context	Incorrect	
2	1	Male	/fa/	2	Sadness	Tone 1	In context	Correct	
2	2	Female	/pu/	1	Sadness	Tone 1	In context	Correct	
2	2	Female	/pu/	2	Sadness	Tone 1	In context	Correct	
2	1	Male	/pu/	1	Sadness	Tone 1	In context	Correct	
2	1	Male	/pu/	2	Sadness	Tone 1	In context	Correct	
2	2	Female	/ci/	1	Sadness	Tone 2	In context	Correct	
2	2	Female	/ci/	2	Sadness	Tone 2	In context	Correct	
2	1	Male	/ci/	1	Sadness	Tone 2	In context	Correct	
2	1	Male	/ci/	2	Sadness	Tone 2	In context	Correct	
2	2	Female	/fa/	1	Sadness	Tone 2	In context	Correct	
2	2	Female	/fa/	2	Sadness	Tone 2	In context	Correct	
2	1	Male	/fa/	1	Sadness	Tone 2	In context	Correct	
2	1	Male	/fa/	2	Sadness	Tone 2	In context	Correct	
2	2	Female	/pu/	1	Sadness	Tone 2	In context	Correct	
2	2	Female	/pu/	2	Sadness	Tone 2	In context	Correct	
2	1	Male	/pu/	1	Sadness	Tone 2	In context	Correct	
2	1	Male	/pu/	2	Sadness	Tone 2	In context	Correct	
2	2	Female	/ci/	1	Sadness	Tone 3	In context	Correct	
2	2	Female	/ci/	2	Sadness	Tone 3	In context	Correct	
2	1	Male	/ci/	1	Sadness	Tone 3	In context	Correct	
2	1	Male	/ci/	2	Sadness	Tone 3	In context	Correct	
2	2	Female	/fa/	1	Sadness	Tone 3	In context	Correct	
2	2	Female	/fa/	2	Sadness	Tone 3	In context	Correct	
2	1	Male	/fa/	1	Sadness	Tone 3	In context	Correct	
2	1	Male	/fa/	2	Sadness	Tone 3	In context	Correct	
2	2	Female	/pu/	1	Sadness	Tone 3	In context	Correct	
2	2	Female	/pu/	2	Sadness	Tone 3	In context	Correct	
2	1	Male	/pu/	1	Sadness	Tone 3	In context	Correct	
2	1	Male	/pu/	2	Sadness	Tone 3	In context	Correct	
2	2	Female	/ci/	1	Sadness	Tone 4	In context	Correct	
2	2	Female	/ci/	2	Sadness	Tone 4	In context	Correct	
2	1	Male	/ci/	1	Sadness	Tone 4	In context	Correct	
2	1	Male	/ci/	2	Sadness	Tone 4	In context	Correct	
2	2	Female	/fa/	1	Sadness	Tone 4	In context	Correct	
2	2	Female	/fa/	2	Sadness	Tone 4	In context	Correct	
2	1	Male	/fa/	1	Sadness	Tone 4	In context	Correct	
2	1	Male	/fa/	2	Sadness	Tone 4	In context	Correct	
2	2	Female	/pu/	1	Sadness	Tone 4	In context	Correct	
2	2	Female	/pu/	2	Sadness	Tone 4	In context	Correct	
2	1	Male	/pu/	1	Sadness	Tone 4	In context	Correct	
2	1	Male	/pu/	2	Sadness	Tone 4	In context	Correct	
3	2	Female	/ci/	1	Anger	Tone 1	In isolation	Correct	
3	2	Female	/ci/	2	Anger	Tone 1	In isolation	Correct	
3	1	Male	/ci/	1	Anger	Tone 1	In isolation	Correct	
3	1	Male	/ci/	2	Anger	Tone 1	In isolation	Correct	
3	2	Female	/fa/	1	Anger	Tone 1	In isolation	Correct	
3	2	Female	/fa/	2	Anger	Tone 1	In isolation	Correct	
3	1	Male	/fa/	1	Anger	Tone 1	In isolation	Correct	
3	1	Male	/fa/	2	Anger	Tone 1	In isolation	Incorrect	

3	2	Female	/pu/	1	Anger	Tone 1	In isolation	Incorrect	
3	2	Female	/pu/	2	Anger	Tone 1	In isolation	Correct	
3	1	Male	/pu/	1	Anger	Tone 1	In isolation	Incorrect	
3	1	Male	/pu/	2	Anger	Tone 1	In isolation	Correct	
3	2	Female	/ci/	1	Anger	Tone 2	In isolation	Incorrect	
3	2	Female	/ci/	2	Anger	Tone 2	In isolation	Incorrect	
3	1	Male	/ci/	1	Anger	Tone 2	In isolation	Incorrect	
3	1	Male	/ci/	2	Anger	Tone 2	In isolation	Incorrect	
3	2	Female	/fa/	1	Anger	Tone 2	In isolation	Correct	
3	2	Female	/fa/	2	Anger	Tone 2	In isolation	Correct	
3	1	Male	/fa/	1	Anger	Tone 2	In isolation	Correct	
3	1	Male	/fa/	2	Anger	Tone 2	In isolation	Correct	
3	2	Female	/pu/	1	Anger	Tone 2	In isolation	Correct	
3	2	Female	/pu/	2	Anger	Tone 2	In isolation	Correct	
3	1	Male	/pu/	1	Anger	Tone 2	In isolation	Correct	
3	1	Male	/pu/	2	Anger	Tone 2	In isolation	Correct	
3	2	Female	/ci/	1	Anger	Tone 3	In isolation	Incorrect	
3	2	Female	/ci/	2	Anger	Tone 3	In isolation	Incorrect	
3	1	Male	/ci/	1	Anger	Tone 3	In isolation	Incorrect	
3	1	Male	/ci/	2	Anger	Tone 3	In isolation	Incorrect	
3	2	Female	/fa/	1	Anger	Tone 3	In isolation	Incorrect	
3	2	Female	/fa/	2	Anger	Tone 3	In isolation	Incorrect	
3	1	Male	/fa/	1	Anger	Tone 3	In isolation	Incorrect	
3	1	Male	/fa/	2	Anger	Tone 3	In isolation	Correct	
3	2	Female	/pu/	1	Anger	Tone 3	In isolation	Incorrect	
3	2	Female	/pu/	2	Anger	Tone 3	In isolation	Incorrect	
3	1	Male	/pu/	1	Anger	Tone 3	In isolation	Incorrect	
3	1	Male	/pu/	2	Anger	Tone 3	In isolation	Incorrect	
3	2	Female	/ci/	1	Anger	Tone 4	In isolation	Incorrect	
3	2	Female	/ci/	2	Anger	Tone 4	In isolation	Incorrect	
3	1	Male	/ci/	1	Anger	Tone 4	In isolation	Incorrect	
3	1	Male	/ci/	2	Anger	Tone 4	In isolation	Correct	
3	2	Female	/fa/	1	Anger	Tone 4	In isolation	Correct	
3	2	Female	/fa/	2	Anger	Tone 4	In isolation	Correct	
3	1	Male	/fa/	1	Anger	Tone 4	In isolation	Correct	
3	1	Male	/fa/	2	Anger	Tone 4	In isolation	Correct	
3	2	Female	/pu/	1	Anger	Tone 4	In isolation	Correct	
3	2	Female	/pu/	2	Anger	Tone 4	In isolation	Correct	
3	1	Male	/pu/	1	Anger	Tone 4	In isolation	Correct	
3	1	Male	/pu/	2	Anger	Tone 4	In isolation	Correct	
3	2	Female	/ci/	1	Fear	Tone 1	In isolation	Incorrect	
3	2	Female	/ci/	2	Fear	Tone 1	In isolation	Correct	
3	1	Male	/ci/	1	Fear	Tone 1	In isolation	Correct	
3	1	Male	/ci/	2	Fear	Tone 1	In isolation	Correct	
3	2	Female	/fa/	1	Fear	Tone 1	In isolation	Correct	
3	2	Female	/fa/	2	Fear	Tone 1	In isolation	Correct	
3	1	Male	/fa/	1	Fear	Tone 1	In isolation	Correct	
3	1	Male	/fa/	2	Fear	Tone 1	In isolation	Correct	
3	2	Female	/pu/	1	Fear	Tone 1	In isolation	Correct	
3	2	Female	/pu/	2	Fear	Tone 1	In isolation	Correct	
3	1	Male	/pu/	1	Fear	Tone 1	In isolation	Correct	

1	Male
3	2	Female
3	2	Female
3	1	Male
3	1	Male
3	2	Female
3	2	Female
3	1	Male
3	1	Male
3	2	Female
3	2	Female
3	1	Male
3	1	Male
3	2	Female
3	2	Female
3	1	Male
3	1	Male
3	2	Female
3	2	Female
3	1	Male
3	1	Male
3	2	Female
3	2	Female
3	1	Male
3	1	Male
3	2	Female
3	2	Female
3	1	Male
3	1	Male
3	2	Female
3	2	Female
3	1	Male
3	1	Male
3	2	Female
3	2	Female
3	1	Male
3	1	Male
/pu/
/ci/
/ci/
/ci/
/ci/
/fa/
/fa/
/fa/
/fa/
/pu/
/pu/
/pu/
/pu/
/ci/
/ci/
/ci/
/ci/
/fa/
/fa/
/fa/
/fa/
/pu/
/pu/
/pu/
/pu/
/ci/
/ci/
/ci/
/ci/
/fa/
/fa/
/fa/
/fa/
/pu/
/pu/
/pu/
/pu/
2	Fear
Fear
Fear
Fear
Fear
Fear
Fear
Fear
Fear
Fear
Fear
Fear
Fear
Fear
Fear
Fear
Fear
Fear
Fear
Fear
Fear
Fear
Fear
Fear
Fear
Fear
Fear
Fear
Fear
Fear
Fear
Fear
Fear
Fear
Fear
Fear
Fear
Tone 1In isolation Correct Tone 2In isolation Incorrect Tone 2In isolation Incorrect Tone 2In isolation Correct Tone 2In isolation Incorrect Tone 2In isolation Incorrect Tone 2In isolation Correct Tone 2In isolation Correct Tone 2In isolation Correct Tone 2In isolation Incorrect Tone 2In isolation Incorrect Tone 2In isolation Correct Tone 2In isolation Incorrect Tone 3In isolation Incorrect Tone 3In isolation Incorrect Tone 3In isolation Incorrect Tone 3In isolation Incorrect Tone 3In isolation Incorrect Tone 3In isolation Correct Tone 3In isolation Incorrect Tone 3In isolation Incorrect Tone 3In isolation Incorrect Tone 3In isolation Incorrect Tone 3In isolation Incorrect Tone 3In isolation Incorrect Tone 4In isolation Incorrect Tone 4In isolation Incorrect Tone 4In isolation Incorrect Tone 4In isolation Incorrect Tone 4In isolation Correct Tone 4In isolation Incorrect Tone 4In isolation Correct Tone 4In isolation Incorrect Tone 4In isolation Incorrect Tone 4In isolation Correct Tone 4In isolation Incorrect Tone 4In isolation Incorrect

2	Female
3	2	Female
3	1	Male
3	1	Male
3	2	Female
3	2	Female
3	1	Male
3	1	Male
3	2	Female
3	2	Female
3	1	Male
3	1	Male
3	2	Female
3	2	Female
/ci/
/ci/
/ci/
/ci/
/fa/
/fa/
/fa/
/fa/
/pu/
/pu/
/pu/
/pu/
/ci/
/ci/
HappinesTone 1In isolation Correct
HappinesTone 1In isolation Incorrect
HappinesTone 1In isolation Correct
HappinesTone 1In isolation Correct
HappinesTone 1In isolation Incorrect
HappinesTone 1In isolation Incorrect
HappinesTone 1In isolation Correct
HappinesTone 1In isolation Correct
HappinesTone 1In isolation Incorrect
HappinesTone 1In isolation Incorrect
HappinesTone 1In isolation Correct
HappinesTone 1In isolation Incorrect
HappinesTone 2In isolation Incorrect
HappinesTone 2In isolation Correct

1	Male
3	1	Male
3	2	Female
3	2	Female
3	1	Male
3	1	Male
3	2	Female
3	2	Female
3	1	Male
3	1	Male
3	2	Female
3	2	Female
3	1	Male
3	1	Male
3	2	Female
3	2	Female
3	1	Male
3	1	Male
3	2	Female
1	2	Female
3	2	Female
3	1	Male
3	1	Male
3	2	Female
3	2	Female
3	1	Male
3	1	Male
3	2	Female
3	2	Female
3	1	Male
3	1	Male
3	2	Female
3	2	Female
3	1	Male
3	1	Male
/ci/
/ci/
/fa/
/fa/
/fa/
/fa/
/pu/
/pu/
/pu/
/pu/
/ci/
/ci/
/ci/
/ci/
/fa/
/fa/
/fa/
/fa/
/pu/
/pu/
/pu/
/pu/
/pu/
/ci/
/ci/
/ci/
/ci/
/fa/
/fa/
/fa/
/fa/
/pu/
/pu/
/pu/
/pu/
HappinesTone 2In isolation Incorrect
HappinesTone 2In isolation Correct
HappinesTone 2In isolation Correct
HappinesTone 2In isolation Correct
HappinesTone 2In isolation Correct
HappinesTone 2In isolation Correct
HappinesTone 2In isolation Correct
HappinesTone 2In isolation Correct
HappinesTone 2In isolation Correct
HappinesTone 2In isolation Correct
HappinesTone 3In isolation Incorrect
HappinesTone 3In isolation Incorrect
HappinesTone 3In isolation Correct
HappinesTone 3In isolation Incorrect
HappinesTone 3In isolation Incorrect
HappinesTone 3In isolation Incorrect
HappinesTone 3In isolation Incorrect
HappinesTone 3In isolation Incorrect
HappinesTone 3In isolation Incorrect
HappinesTone 3In isolation Correct
2	HappinesTone 3In isolation Incorrect
HappinesTone 3In isolation Incorrect
HappinesTone 3In isolation Incorrect
HappinesTone 4In isolation Incorrect
HappinesTone 4In isolation Incorrect
HappinesTone 4In isolation Correct
HappinesTone 4In isolation Incorrect
HappinesTone 4In isolation Correct
HappinesTone 4In isolation Incorrect
HappinesTone 4In isolation Correct
HappinesTone 4In isolation Correct
HappinesTone 4In isolation Incorrect
HappinesTone 4In isolation Incorrect
HappinesTone 4In isolation Correct
HappinesTone 4In isolation Incorrect

2	Female
3	2	Female
3	1	Male
3	1	Male
3	2	Female
3	2	Female
3	1	Male
3	1	Male
3	2	Female
3	2	Female
3	1	Male
3	1	Male
3	2	Female
3	2	Female
3	1	Male
3	1	Male
/ci/
/ci/
/ci/
/ci/
/fa/
/fa/
/fa/
/fa/
/pu/
/pu/
/pu/
/pu/
/ci/
/ci/
/ci/
/ci/
Neutral Tone 1In isolation Correct
Neutral Tone 1In isolation Correct
Neutral Tone 1In isolation Correct
Neutral Tone 1In isolation Correct
Neutral Tone 1In isolation Correct
Neutral Tone 1In isolation Correct
Neutral Tone 1In isolation Correct
Neutral Tone 1In isolation Correct
Neutral Tone 1In isolation Correct
Neutral Tone 1In isolation Correct
Neutral Tone 1In isolation Correct
Neutral Tone 1In isolation Correct
Neutral Tone 2In isolation Correct
Neutral Tone 2In isolation Correct
Neutral Tone 2In isolation Correct
Neutral Tone 2In isolation Correct

3	2	Female	/fa/	1	Neutral	Tone 2	In isolation	Correct	
3	2	Female	/fa/	2	Neutral	Tone 2	In isolation	Correct	
3	1	Male	/fa/	1	Neutral	Tone 2	In isolation	Correct	
3	1	Male	/fa/	2	Neutral	Tone 2	In isolation	Correct	
3	2	Female	/pu/	1	Neutral	Tone 2	In isolation	Correct	
3	2	Female	/pu/	2	Neutral	Tone 2	In isolation	Correct	
3	1	Male	/pu/	1	Neutral	Tone 2	In isolation	Correct	
3	1	Male	/pu/	2	Neutral	Tone 2	In isolation	Correct	
3	2	Female	/ci/	1	Neutral	Tone 3	In isolation	Correct	
3	2	Female	/ci/	2	Neutral	Tone 3	In isolation	Correct	
3	1	Male	/ci/	1	Neutral	Tone 3	In isolation	Correct	
3	1	Male	/ci/	2	Neutral	Tone 3	In isolation	Correct	
3	2	Female	/fa/	1	Neutral	Tone 3	In isolation	Correct	
3	2	Female	/fa/	2	Neutral	Tone 3	In isolation	Correct	
3	1	Male	/fa/	1	Neutral	Tone 3	In isolation	Correct	
3	1	Male	/fa/	2	Neutral	Tone 3	In isolation	Correct	
3	2	Female	/pu/	1	Neutral	Tone 3	In isolation	Correct	
3	2	Female	/pu/	2	Neutral	Tone 3	In isolation	Correct	
3	1	Male	/pu/	1	Neutral	Tone 3	In isolation	Correct	
3	1	Male	/pu/	2	Neutral	Tone 3	In isolation	Correct	
3	2	Female	/ci/	1	Neutral	Tone 4	In isolation	Correct	
3	2	Female	/ci/	2	Neutral	Tone 4	In isolation	Correct	
3	1	Male	/ci/	1	Neutral	Tone 4	In isolation	Correct	
3	1	Male	/ci/	2	Neutral	Tone 4	In isolation	Correct	
3	2	Female	/fa/	1	Neutral	Tone 4	In isolation	Correct	
3	2	Female	/fa/	2	Neutral	Tone 4	In isolation	Correct	
3	1	Male	/fa/	1	Neutral	Tone 4	In isolation	Correct	
3	1	Male	/fa/	2	Neutral	Tone 4	In isolation	Correct	
3	2	Female	/pu/	1	Neutral	Tone 4	In isolation	Correct	
3	2	Female	/pu/	2	Neutral	Tone 4	In isolation	Correct	
3	1	Male	/pu/	1	Neutral	Tone 4	In isolation	Correct	
3	1	Male	/pu/	2	Neutral	Tone 4	In isolation	Correct	
3	2	Female	/ci/	1	Sadness	Tone 1	In isolation	Correct	
3	2	Female	/ci/	2	Sadness	Tone 1	In isolation	Correct	
3	1	Male	/ci/	1	Sadness	Tone 1	In isolation	Correct	
3	1	Male	/ci/	2	Sadness	Tone 1	In isolation	Correct	
3	2	Female	/fa/	1	Sadness	Tone 1	In isolation	Correct	
3	2	Female	/fa/	2	Sadness	Tone 1	In isolation	Incorrect	
3	1	Male	/fa/	1	Sadness	Tone 1	In isolation	Correct	
3	1	Male	/fa/	2	Sadness	Tone 1	In isolation	Correct	
3	2	Female	/pu/	1	Sadness	Tone 1	In isolation	Correct	
3	2	Female	/pu/	2	Sadness	Tone 1	In isolation	Correct	
3	1	Male	/pu/	1	Sadness	Tone 1	In isolation	Incorrect	
3	1	Male	/pu/	2	Sadness	Tone 1	In isolation	Correct	
3	2	Female	/ci/	1	Sadness	Tone 2	In isolation	Incorrect	
3	2	Female	/ci/	2	Sadness	Tone 2	In isolation	Incorrect	
3	1	Male	/ci/	1	Sadness	Tone 2	In isolation	Incorrect	
3	1	Male	/ci/	2	Sadness	Tone 2	In isolation	Incorrect	
3	2	Female	/fa/	1	Sadness	Tone 2	In isolation	Correct	
3	2	Female	/fa/	2	Sadness	Tone 2	In isolation	Incorrect	
3	1	Male	/fa/	1	Sadness	Tone 2	In isolation	Correct	

3	1	Male	/fa/	2	Sadness	Tone 2	In isolation	Correct	
3	2	Female	/pu/	1	Sadness	Tone 2	In isolation	Correct	
3	2	Female	/pu/	2	Sadness	Tone 2	In isolation	Correct	
3	1	Male	/pu/	1	Sadness	Tone 2	In isolation	Incorrect	
3	1	Male	/pu/	2	Sadness	Tone 2	In isolation	Correct	
3	2	Female	/ci/	1	Sadness	Tone 3	In isolation	Incorrect	
3	2	Female	/ci/	2	Sadness	Tone 3	In isolation	Incorrect	
3	1	Male	/ci/	1	Sadness	Tone 3	In isolation	Incorrect	
3	1	Male	/ci/	2	Sadness	Tone 3	In isolation	Incorrect	
3	2	Female	/fa/	1	Sadness	Tone 3	In isolation	Incorrect	
3	2	Female	/fa/	2	Sadness	Tone 3	In isolation	Incorrect	
3	1	Male	/fa/	1	Sadness	Tone 3	In isolation	Incorrect	
3	1	Male	/fa/	2	Sadness	Tone 3	In isolation	Incorrect	
3	2	Female	/pu/	1	Sadness	Tone 3	In isolation	Incorrect	
3	2	Female	/pu/	2	Sadness	Tone 3	In isolation	Incorrect	
3	1	Male	/pu/	1	Sadness	Tone 3	In isolation	Incorrect	
3	1	Male	/pu/	2	Sadness	Tone 3	In isolation	Incorrect	
3	2	Female	/ci/	1	Sadness	Tone 4	In isolation	Incorrect	
3	2	Female	/ci/	2	Sadness	Tone 4	In isolation	Incorrect	
3	1	Male	/ci/	1	Sadness	Tone 4	In isolation	Incorrect	
3	1	Male	/ci/	2	Sadness	Tone 4	In isolation	Correct	
3	2	Female	/fa/	1	Sadness	Tone 4	In isolation	Correct	
3	2	Female	/fa/	2	Sadness	Tone 4	In isolation	Incorrect	
3	1	Male	/fa/	1	Sadness	Tone 4	In isolation	Incorrect	
3	1	Male	/fa/	2	Sadness	Tone 4	In isolation	Correct	
3	2	Female	/pu/	1	Sadness	Tone 4	In isolation	Incorrect	
3	2	Female	/pu/	2	Sadness	Tone 4	In isolation	Incorrect	
3	1	Male	/pu/	1	Sadness	Tone 4	In isolation	Incorrect	
3	1	Male	/pu/	2	Sadness	Tone 4	In isolation	Incorrect	
3	2	Female	/ci/	1	Anger	Tone 1	In context	Correct	
3	2	Female	/ci/	2	Anger	Tone 1	In context	Incorrect	
3	1	Male	/ci/	1	Anger	Tone 1	In context	Correct	
3	1	Male	/ci/	2	Anger	Tone 1	In context	Correct	
3	2	Female	/fa/	1	Anger	Tone 1	In context	Correct	
3	2	Female	/fa/	2	Anger	Tone 1	In context	Correct	
3	1	Male	/fa/	1	Anger	Tone 1	In context	Correct	
3	1	Male	/fa/	2	Anger	Tone 1	In context	Incorrect	
3	2	Female	/pu/	1	Anger	Tone 1	In context	Incorrect	
3	2	Female	/pu/	2	Anger	Tone 1	In context	Correct	
3	1	Male	/pu/	1	Anger	Tone 1	In context	Correct	
3	1	Male	/pu/	2	Anger	Tone 1	In context	Correct	
3	2	Female	/ci/	1	Anger	Tone 2	In context	Correct	
3	2	Female	/ci/	2	Anger	Tone 2	In context	Incorrect	
3	1	Male	/ci/	1	Anger	Tone 2	In context	Correct	
3	1	Male	/ci/	2	Anger	Tone 2	In context	Correct	
3	2	Female	/fa/	1	Anger	Tone 2	In context	Correct	
3	2	Female	/fa/	2	Anger	Tone 2	In context	Correct	
3	1	Male	/fa/	1	Anger	Tone 2	In context	Incorrect	
3	1	Male	/fa/	2	Anger	Tone 2	In context	Incorrect	
3	2	Female	/pu/	1	Anger	Tone 2	In context	Correct	
3	2	Female	/pu/	2	Anger	Tone 2	In context	Correct	

3	1	Male	/pu/	1	Anger	Tone 2	In context	Correct	
3	1	Male	/pu/	2	Anger	Tone 2	In context	Correct	
3	2	Female	/ci/	1	Anger	Tone 3	In context	Correct	
3	2	Female	/ci/	2	Anger	Tone 3	In context	Correct	
3	1	Male	/ci/	1	Anger	Tone 3	In context	Correct	
3	1	Male	/ci/	2	Anger	Tone 3	In context	Correct	
3	2	Female	/fa/	1	Anger	Tone 3	In context	Correct	
3	2	Female	/fa/	2	Anger	Tone 3	In context	Correct	
3	1	Male	/fa/	1	Anger	Tone 3	In context	Correct	
3	1	Male	/fa/	2	Anger	Tone 3	In context	Correct	
3	2	Female	/pu/	1	Anger	Tone 3	In context	Incorrect	
3	2	Female	/pu/	2	Anger	Tone 3	In context	Incorrect	
3	1	Male	/pu/	1	Anger	Tone 3	In context	Correct	
3	1	Male	/pu/	2	Anger	Tone 3	In context	Incorrect	
3	2	Female	/ci/	1	Anger	Tone 4	In context	Correct	
3	2	Female	/ci/	2	Anger	Tone 4	In context	Correct	
3	1	Male	/ci/	1	Anger	Tone 4	In context	Correct	
3	1	Male	/ci/	2	Anger	Tone 4	In context	Correct	
3	2	Female	/fa/	1	Anger	Tone 4	In context	Correct	
3	2	Female	/fa/	2	Anger	Tone 4	In context	Correct	
3	1	Male	/fa/	1	Anger	Tone 4	In context	Correct	
3	1	Male	/fa/	2	Anger	Tone 4	In context	Correct	
3	2	Female	/pu/	1	Anger	Tone 4	In context	Correct	
3	2	Female	/pu/	2	Anger	Tone 4	In context	Correct	
3	1	Male	/pu/	1	Anger	Tone 4	In context	Correct	
3	1	Male	/pu/	2	Anger	Tone 4	In context	Correct	
3	2	Female	/ci/	1	Fear	Tone 1	In context	Correct	
3	2	Female	/ci/	2	Fear	Tone 1	In context	Correct	
3	1	Male	/ci/	1	Fear	Tone 1	In context	Correct	
3	1	Male	/ci/	2	Fear	Tone 1	In context	Correct	
3	2	Female	/fa/	1	Fear	Tone 1	In context	Correct	
3	2	Female	/fa/	2	Fear	Tone 1	In context	Correct	
3	1	Male	/fa/	1	Fear	Tone 1	In context	Correct	
3	1	Male	/fa/	2	Fear	Tone 1	In context	Correct	
3	2	Female	/pu/	1	Fear	Tone 1	In context	Correct	
3	2	Female	/pu/	2	Fear	Tone 1	In context	Correct	
3	1	Male	/pu/	1	Fear	Tone 1	In context	Incorrect	
3	1	Male	/pu/	2	Fear	Tone 1	In context	Correct	
3	2	Female	/ci/	1	Fear	Tone 2	In context	Correct	
3	2	Female	/ci/	2	Fear	Tone 2	In context	Incorrect	
3	1	Male	/ci/	1	Fear	Tone 2	In context	Correct	
3	1	Male	/ci/	2	Fear	Tone 2	In context	Correct	
3	2	Female	/fa/	1	Fear	Tone 2	In context	Incorrect	
3	2	Female	/fa/	2	Fear	Tone 2	In context	Incorrect	
3	1	Male	/fa/	1	Fear	Tone 2	In context	Correct	
3	1	Male	/fa/	2	Fear	Tone 2	In context	Incorrect	
3	2	Female	/pu/	1	Fear	Tone 2	In context	Correct	
3	2	Female	/pu/	2	Fear	Tone 2	In context	Correct	
3	1	Male	/pu/	1	Fear	Tone 2	In context	Correct	
3	1	Male	/pu/	2	Fear	Tone 2	In context	Correct	
3	2	Female	/ci/	1	Fear	Tone 3	In context	Correct	

2	Female
3	1	Male
3	1	Male
3	2	Female
3	2	Female
3	1	Male
3	1	Male
/ci/
/ci/
/ci/
/fa/
/fa/
/fa/
/fa/
2	Fear
Fear
Fear
Fear
Fear
Fear
Fear
Tone 3 In context Tone 3 In context Tone 3 In context Tone 3 In context Tone 3 In context Tone 3 In context Tone 3 In context
Correct Correct Correct Correct Correct Correct Correct

2	Female
3	2	Female
3	1	Male
3	1	Male
3	2	Female
/pu/
/pu/
/pu/
/pu/
/ci/
Fear
Fear
Fear
Fear
Fear
Tone 3 In context Incorrect Tone 3 In context Incorrect Tone 3 In context Incorrect Tone 3 In context Incorrect Tone 4 In context Incorrect

3	2	Female
3	1	Male
3	1	Male
3	2	Female
3	2	Female
3	1	Male
3	1	Male
3	2	Female
3	2	Female
3	1	Male
3	1	Male
/ci/
/ci/
/ci/
/fa/
/fa/
/fa/
/fa/
/pu/
/pu/
/pu/
/pu/
Fear
Fear
Fear
Fear
Fear
Fear
Fear
Fear
Fear
Fear
Fear
Tone 4 In context Tone 4 In context Tone 4 In context Tone 4 In context Tone 4 In context Tone 4 In context Tone 4 In context Tone 4 In context Tone 4 In context Tone 4 In context Tone 4 In context
Correct Correct Correct Correct Correct Correct Correct Correct Correct Correct Correct

2	Female
3	2	Female
3	1	Male
3	1	Male
3	2	Female
3	2	Female
3	1	Male
3	1	Male
3	2	Female
3	2	Female
3	1	Male
/ci/
/ci/
/ci/
/ci/
/fa/
/fa/
/fa/
/fa/
/pu/
/pu/
/pu/
HappinesTone 1 In context
HappinesTone 1 In context
HappinesTone 1 In context
HappinesTone 1 In context
HappinesTone 1 In context
HappinesTone 1 In context
HappinesTone 1 In context
HappinesTone 1 In context
HappinesTone 1 In context
HappinesTone 1 In context
HappinesTone 1 In context
Correct Correct Correct Correct Correct Correct Correct Correct Correct Correct Correct

3	1	Male
/pu/
HappinesTone 1 In context Incorrect

2	Female
3	2	Female
/ci/
/ci/
HappinesTone 2 In context
HappinesTone 2 In context
Correct Correct

1	Male
/ci/
HappinesTone 2 In context Incorrect

3	1	Male
3	2	Female
3	2	Female
3	1	Male
3	1	Male
3	2	Female
3	2	Female
3	1	Male
3	1	Male
3	2	Female
3	2	Female
3	1	Male
3	1	Male
/ci/
/fa/
/fa/
/fa/
/fa/
/pu/
/pu/
/pu/
/pu/
/ci/
/ci/
/ci/
/ci/
HappinesTone 2 In context
HappinesTone 2 In context
HappinesTone 2 In context
HappinesTone 2 In context
HappinesTone 2 In context
HappinesTone 2 In context
HappinesTone 2 In context
HappinesTone 2 In context
HappinesTone 2 In context
HappinesTone 3 In context
HappinesTone 3 In context
HappinesTone 3 In context
HappinesTone 3 In context
Correct Correct Correct Correct Correct Correct Correct Correct Correct Correct Correct Correct Correct

2	Female
3	2	Female
3	1	Male
3	1	Male
/fa/
/fa/
/fa/
/fa/
HappinesTone 3 In context
HappinesTone 3 In context
HappinesTone 3 In context
HappinesTone 3 In context
Correct Correct Correct Correct

2	Female
3	2	Female
3	1	Male
3	1	Male
/pu/
/pu/
/pu/
/pu/
HappinesTone 3 In context Incorrect
HappinesTone 3 In context Incorrect
HappinesTone 3 In context Incorrect
HappinesTone 3 In context Incorrect

2	Female
3	2	Female
/ci/
/ci/
HappinesTone 4 In context
HappinesTone 4 In context
Correct Correct

1	Male
/ci/
HappinesTone 4 In context Incorrect

3	1	Male
3	2	Female
3	2	Female
/ci/
/fa/
/fa/
HappinesTone 4 In context
HappinesTone 4 In context
HappinesTone 4 In context
Correct Correct Correct

1	Male
/fa/
HappinesTone 4 In context Incorrect

3	1	Male
/fa/
HappinesTone 4 In context
Correct

2	Female
3	2	Female
/pu/
/pu/
HappinesTone 4 In context Incorrect
HappinesTone 4 In context Incorrect

1	Male
3	1	Male
/pu/
/pu/
HappinesTone 4 In context
HappinesTone 4 In context
Correct Correct

2	Female
3	2	Female
3	1	Male
3	1	Male
3	2	Female
3	2	Female
3	1	Male
3	1	Male
3	2	Female
3	2	Female
3	1	Male
3	1	Male
3	2	Female
3	2	Female
3	1	Male
3	1	Male
3	2	Female
3	2	Female
3	1	Male
3	1	Male
3	2	Female
3	2	Female
3	1	Male
3	1	Male
3	2	Female
3	2	Female
3	1	Male
3	1	Male
3	2	Female
3	2	Female
3	1	Male
/ci/
/ci/
/ci/
/ci/
/fa/
/fa/
/fa/
/fa/
/pu/
/pu/
/pu/
/pu/
/ci/
/ci/
/ci/
/ci/
/fa/
/fa/
/fa/
/fa/
/pu/
/pu/
/pu/
/pu/
/ci/
/ci/
/ci/
/ci/
/fa/
/fa/
/fa/
Neutral Tone 1 In context
Neutral Tone 1 In context
Neutral Tone 1 In context
Neutral Tone 1 In context
Neutral Tone 1 In context
Neutral Tone 1 In context
Neutral Tone 1 In context
Neutral Tone 1 In context
Neutral Tone 1 In context
Neutral Tone 1 In context
Neutral Tone 1 In context
Neutral Tone 1 In context
Neutral Tone 2 In context
Neutral Tone 2 In context
Neutral Tone 2 In context
Neutral Tone 2 In context
Neutral Tone 2 In context
Neutral Tone 2 In context
Neutral Tone 2 In context
Neutral Tone 2 In context
Neutral Tone 2 In context
Neutral Tone 2 In context
Neutral Tone 2 In context
Neutral Tone 2 In context
Neutral Tone 3 In context
Neutral Tone 3 In context
Neutral Tone 3 In context
Neutral Tone 3 In context
Neutral Tone 3 In context
Neutral Tone 3 In context
1	Neutral Tone 3 In context
Correct Correct Correct Correct Correct Correct Correct Correct Correct Correct Correct Correct Correct Correct Correct Correct Correct Correct Correct Correct Correct Correct Correct Correct Correct Correct Correct Correct Correct Correct Correct

3	1	Male	/fa/	2	Neutral	Tone 3	In context	Correct	
3	2	Female	/pu/	1	Neutral	Tone 3	In context	Correct	
3	2	Female	/pu/	2	Neutral	Tone 3	In context	Correct	
3	1	Male	/pu/	1	Neutral	Tone 3	In context	Correct	
3	1	Male	/pu/	2	Neutral	Tone 3	In context	Correct	
3	2	Female	/ci/	1	Neutral	Tone 4	In context	Correct	
3	2	Female	/ci/	2	Neutral	Tone 4	In context	Correct	
3	1	Male	/ci/	1	Neutral	Tone 4	In context	Correct	
3	1	Male	/ci/	2	Neutral	Tone 4	In context	Correct	
3	2	Female	/fa/	1	Neutral	Tone 4	In context	Correct	
3	2	Female	/fa/	2	Neutral	Tone 4	In context	Correct	
3	1	Male	/fa/	1	Neutral	Tone 4	In context	Correct	
3	1	Male	/fa/	2	Neutral	Tone 4	In context	Correct	
3	2	Female	/pu/	1	Neutral	Tone 4	In context	Correct	
3	2	Female	/pu/	2	Neutral	Tone 4	In context	Correct	
3	1	Male	/pu/	1	Neutral	Tone 4	In context	Correct	
3	1	Male	/pu/	2	Neutral	Tone 4	In context	Correct	
3	2	Female	/ci/	1	Sadness	Tone 1	In context	Correct	
3	2	Female	/ci/	2	Sadness	Tone 1	In context	Correct	
3	1	Male	/ci/	1	Sadness	Tone 1	In context	Correct	
3	1	Male	/ci/	2	Sadness	Tone 1	In context	Correct	
3	2	Female	/fa/	1	Sadness	Tone 1	In context	Correct	
3	2	Female	/fa/	2	Sadness	Tone 1	In context	Correct	
3	1	Male	/fa/	1	Sadness	Tone 1	In context	Incorrect	
3	1	Male	/fa/	2	Sadness	Tone 1	In context	Correct	
3	2	Female	/pu/	1	Sadness	Tone 1	In context	Correct	
3	2	Female	/pu/	2	Sadness	Tone 1	In context	Correct	
3	1	Male	/pu/	1	Sadness	Tone 1	In context	Correct	
3	1	Male	/pu/	2	Sadness	Tone 1	In context	Correct	
3	2	Female	/ci/	1	Sadness	Tone 2	In context	Correct	
3	2	Female	/ci/	2	Sadness	Tone 2	In context	Correct	
3	1	Male	/ci/	1	Sadness	Tone 2	In context	Correct	
3	1	Male	/ci/	2	Sadness	Tone 2	In context	Correct	
3	2	Female	/fa/	1	Sadness	Tone 2	In context	Incorrect	
3	2	Female	/fa/	2	Sadness	Tone 2	In context	Correct	
3	1	Male	/fa/	1	Sadness	Tone 2	In context	Incorrect	
3	1	Male	/fa/	2	Sadness	Tone 2	In context	Incorrect	
3	2	Female	/pu/	1	Sadness	Tone 2	In context	Correct	
3	2	Female	/pu/	2	Sadness	Tone 2	In context	Correct	
3	1	Male	/pu/	1	Sadness	Tone 2	In context	Correct	
3	1	Male	/pu/	2	Sadness	Tone 2	In context	Correct	
3	2	Female	/ci/	1	Sadness	Tone 3	In context	Correct	
3	2	Female	/ci/	2	Sadness	Tone 3	In context	Correct	
3	1	Male	/ci/	1	Sadness	Tone 3	In context	Correct	
3	1	Male	/ci/	2	Sadness	Tone 3	In context	Correct	
3	2	Female	/fa/	1	Sadness	Tone 3	In context	Correct	
3	2	Female	/fa/	2	Sadness	Tone 3	In context	Correct	
3	1	Male	/fa/	1	Sadness	Tone 3	In context	Correct	
3	1	Male	/fa/	2	Sadness	Tone 3	In context	Correct	
3	2	Female	/pu/	1	Sadness	Tone 3	In context	Incorrect	
3	2	Female	/pu/	2	Sadness	Tone 3	In context	Incorrect	

3	1	Male	/pu/	1	Sadness	Tone 3	In context	Correct	
3	1	Male	/pu/	2	Sadness	Tone 3	In context	Incorrect	
3	2	Female	/ci/	1	Sadness	Tone 4	In context	Correct	
3	2	Female	/ci/	2	Sadness	Tone 4	In context	Correct	
3	1	Male	/ci/	1	Sadness	Tone 4	In context	Correct	
3	1	Male	/ci/	2	Sadness	Tone 4	In context	Correct	
3	2	Female	/fa/	1	Sadness	Tone 4	In context	Correct	
3	2	Female	/fa/	2	Sadness	Tone 4	In context	Correct	
3	1	Male	/fa/	1	Sadness	Tone 4	In context	Correct	
3	1	Male	/fa/	2	Sadness	Tone 4	In context	Correct	
3	2	Female	/pu/	1	Sadness	Tone 4	In context	Correct	
3	2	Female	/pu/	2	Sadness	Tone 4	In context	Correct	
3	1	Male	/pu/	1	Sadness	Tone 4	In context	Correct	
3	1	Male	/pu/	2	Sadness	Tone 4	In context	Correct	
4	2	Female	/ci/	1	Anger	Tone 1	In isolation	Incorrect	
4	2	Female	/ci/	2	Anger	Tone 1	In isolation	Incorrect	
4	1	Male	/ci/	1	Anger	Tone 1	In isolation	Incorrect	
4	1	Male	/ci/	2	Anger	Tone 1	In isolation	Incorrect	
4	2	Female	/fa/	1	Anger	Tone 1	In isolation	Correct	
4	2	Female	/fa/	2	Anger	Tone 1	In isolation	Correct	
4	1	Male	/fa/	1	Anger	Tone 1	In isolation	Correct	
4	1	Male	/fa/	2	Anger	Tone 1	In isolation	Correct	
4	2	Female	/pu/	1	Anger	Tone 1	In isolation	Incorrect	
4	2	Female	/pu/	2	Anger	Tone 1	In isolation	Incorrect	
4	1	Male	/pu/	1	Anger	Tone 1	In isolation	Incorrect	
4	1	Male	/pu/	2	Anger	Tone 1	In isolation	Incorrect	
4	2	Female	/ci/	1	Anger	Tone 2	In isolation	Incorrect	
4	2	Female	/ci/	2	Anger	Tone 2	In isolation	Incorrect	
4	1	Male	/ci/	1	Anger	Tone 2	In isolation	Correct	
4	1	Male	/ci/	2	Anger	Tone 2	In isolation	Incorrect	
4	2	Female	/fa/	1	Anger	Tone 2	In isolation	Correct	
4	2	Female	/fa/	2	Anger	Tone 2	In isolation	Correct	
4	1	Male	/fa/	1	Anger	Tone 2	In isolation	Correct	
4	1	Male	/fa/	2	Anger	Tone 2	In isolation	Correct	
4	2	Female	/pu/	1	Anger	Tone 2	In isolation	Correct	
4	2	Female	/pu/	2	Anger	Tone 2	In isolation	Correct	
4	1	Male	/pu/	1	Anger	Tone 2	In isolation	Correct	
4	1	Male	/pu/	2	Anger	Tone 2	In isolation	Correct	
4	2	Female	/ci/	1	Anger	Tone 3	In isolation	Incorrect	
4	2	Female	/ci/	2	Anger	Tone 3	In isolation	Incorrect	
4	1	Male	/ci/	1	Anger	Tone 3	In isolation	Incorrect	
4	1	Male	/ci/	2	Anger	Tone 3	In isolation	Incorrect	
4	2	Female	/fa/	1	Anger	Tone 3	In isolation	Correct	
4	2	Female	/fa/	2	Anger	Tone 3	In isolation	Correct	
4	1	Male	/fa/	1	Anger	Tone 3	In isolation	Correct	
4	1	Male	/fa/	2	Anger	Tone 3	In isolation	Correct	
4	2	Female	/pu/	1	Anger	Tone 3	In isolation	Incorrect	
4	2	Female	/pu/	2	Anger	Tone 3	In isolation	Incorrect	
4	1	Male	/pu/	1	Anger	Tone 3	In isolation	Incorrect	
4	1	Male	/pu/	2	Anger	Tone 3	In isolation	Incorrect	
4	2	Female	/ci/	1	Anger	Tone 4	In isolation	Correct	

4	2	Female	/ci/	2	Anger	Tone 4	In isolation	Correct	
4	1	Male	/ci/	1	Anger	Tone 4	In isolation	Correct	
4	1	Male	/ci/	2	Anger	Tone 4	In isolation	Correct	
4	2	Female	/fa/	1	Anger	Tone 4	In isolation	Correct	
4	2	Female	/fa/	2	Anger	Tone 4	In isolation	Correct	
4	1	Male	/fa/	1	Anger	Tone 4	In isolation	Correct	
4	1	Male	/fa/	2	Anger	Tone 4	In isolation	Correct	
4	2	Female	/pu/	1	Anger	Tone 4	In isolation	Correct	
4	2	Female	/pu/	2	Anger	Tone 4	In isolation	Correct	
4	1	Male	/pu/	1	Anger	Tone 4	In isolation	Correct	
4	1	Male	/pu/	2	Anger	Tone 4	In isolation	Correct	
4	2	Female	/ci/	1	Fear	Tone 1	In isolation	Incorrect	
4	2	Female	/ci/	2	Fear	Tone 1	In isolation	Incorrect	
4	1	Male	/ci/	1	Fear	Tone 1	In isolation	Incorrect	
4	1	Male	/ci/	2	Fear	Tone 1	In isolation	Incorrect	
4	2	Female	/fa/	1	Fear	Tone 1	In isolation	Correct	
4	2	Female	/fa/	2	Fear	Tone 1	In isolation	Correct	
4	1	Male	/fa/	1	Fear	Tone 1	In isolation	Correct	
4	1	Male	/fa/	2	Fear	Tone 1	In isolation	Correct	
4	2	Female	/pu/	1	Fear	Tone 1	In isolation	Correct	
4	2	Female	/pu/	2	Fear	Tone 1	In isolation	Correct	
4	1	Male	/pu/	1	Fear	Tone 1	In isolation	Correct	
4	1	Male	/pu/	2	Fear	Tone 1	In isolation	Correct	
4	2	Female	/ci/	1	Fear	Tone 2	In isolation	Incorrect	
4	2	Female	/ci/	2	Fear	Tone 2	In isolation	Incorrect	
4	1	Male	/ci/	1	Fear	Tone 2	In isolation	Correct	
4	1	Male	/ci/	2	Fear	Tone 2	In isolation	Correct	
4	2	Female	/fa/	1	Fear	Tone 2	In isolation	Incorrect	
4	2	Female	/fa/	2	Fear	Tone 2	In isolation	Correct	
4	1	Male	/fa/	1	Fear	Tone 2	In isolation	Correct	
4	1	Male	/fa/	2	Fear	Tone 2	In isolation	Correct	
4	2	Female	/pu/	1	Fear	Tone 2	In isolation	Incorrect	
4	2	Female	/pu/	2	Fear	Tone 2	In isolation	Incorrect	
4	1	Male	/pu/	1	Fear	Tone 2	In isolation	Correct	
4	1	Male	/pu/	2	Fear	Tone 2	In isolation	Correct	
4	2	Female	/ci/	1	Fear	Tone 3	In isolation	Incorrect	
4	2	Female	/ci/	2	Fear	Tone 3	In isolation	Incorrect	
4	1	Male	/ci/	1	Fear	Tone 3	In isolation	Incorrect	
4	1	Male	/ci/	2	Fear	Tone 3	In isolation	Incorrect	
4	2	Female	/fa/	1	Fear	Tone 3	In isolation	Incorrect	
4	2	Female	/fa/	2	Fear	Tone 3	In isolation	Correct	
4	1	Male	/fa/	1	Fear	Tone 3	In isolation	Correct	
4	1	Male	/fa/	2	Fear	Tone 3	In isolation	Incorrect	
4	2	Female	/pu/	1	Fear	Tone 3	In isolation	Incorrect	
4	2	Female	/pu/	2	Fear	Tone 3	In isolation	Incorrect	
4	1	Male	/pu/	1	Fear	Tone 3	In isolation	Incorrect	
4	1	Male	/pu/	2	Fear	Tone 3	In isolation	Incorrect	
4	2	Female	/ci/	1	Fear	Tone 4	In isolation	Correct	
4	2	Female	/ci/	2	Fear	Tone 4	In isolation	Correct	
4	1	Male	/ci/	1	Fear	Tone 4	In isolation	Correct	
4	1	Male	/ci/	2	Fear	Tone 4	In isolation	Correct	

4	2	Female
4	2	Female
4	1	Male
4	1	Male
4	2	Female
4	2	Female
4	1	Male
4	1	Male
/fa/
/fa/
/fa/
/fa/
/pu/
/pu/
/pu/
/pu/
Fear
Fear
Fear
Fear
Fear
Fear
Fear
Fear
Tone 4In isolation Correct Tone 4In isolation Incorrect Tone 4In isolation Correct Tone 4In isolation Correct Tone 4In isolation Correct Tone 4In isolation Correct Tone 4In isolation Correct Tone 4In isolation Correct

4	2	Female
4	2	Female
4	1	Male
4	1	Male
4	2	Female
4	2	Female
4	1	Male
4	1	Male
4	2	Female
4	2	Female
4	1	Male
4	1	Male
4	2	Female
4	2	Female
4	1	Male
4	1	Male
4	2	Female
4	2	Female
4	1	Male
4	1	Male
4	2	Female
4	2	Female
4	1	Male
4	1	Male
4	2	Female
4	2	Female
4	1	Male
4	1	Male
4	2	Female
4	2	Female
4	1	Male
4	1	Male
4	2	Female
4	2	Female
4	1	Male
4	1	Male
4	2	Female
4	2	Female
4	1	Male
4	1	Male
4	2	Female
4	2	Female
4	1	Male
/ci/
/ci/
/ci/
/ci/
/fa/
/fa/
/fa/
/fa/
/pu/
/pu/
/pu/
/pu/
/ci/
/ci/
/ci/
/ci/
/fa/
/fa/
/fa/
/fa/
/pu/
/pu/
/pu/
/pu/
/ci/
/ci/
/ci/
/ci/
/fa/
/fa/
/fa/
/fa/
/pu/
/pu/
/pu/
/pu/
/ci/
/ci/
/ci/
/ci/
/fa/
/fa/
/fa/
HappinesTone 1In isolation Correct
HappinesTone 1In isolation Correct
HappinesTone 1In isolation Incorrect
HappinesTone 1In isolation Incorrect
HappinesTone 1In isolation Incorrect
HappinesTone 1In isolation Incorrect
HappinesTone 1In isolation Correct
HappinesTone 1In isolation Correct
HappinesTone 1In isolation Incorrect
HappinesTone 1In isolation Correct
HappinesTone 1In isolation Correct
HappinesTone 1In isolation Incorrect
HappinesTone 2In isolation Incorrect
HappinesTone 2In isolation Incorrect
HappinesTone 2In isolation Correct
HappinesTone 2In isolation Correct
HappinesTone 2In isolation Correct
HappinesTone 2In isolation Correct
HappinesTone 2In isolation Correct
HappinesTone 2In isolation Correct
HappinesTone 2In isolation Incorrect
HappinesTone 2In isolation Incorrect
HappinesTone 2In isolation Correct
HappinesTone 2In isolation Correct
HappinesTone 3In isolation Incorrect
HappinesTone 3In isolation Incorrect
HappinesTone 3In isolation Incorrect
HappinesTone 3In isolation Incorrect
HappinesTone 3In isolation Incorrect
HappinesTone 3In isolation Correct
HappinesTone 3In isolation Correct
HappinesTone 3In isolation Correct
HappinesTone 3In isolation Incorrect
HappinesTone 3In isolation Incorrect
HappinesTone 3In isolation Correct
HappinesTone 3In isolation Incorrect
HappinesTone 4In isolation Correct
HappinesTone 4In isolation Incorrect
HappinesTone 4In isolation Correct
HappinesTone 4In isolation Correct
HappinesTone 4In isolation Incorrect
HappinesTone 4In isolation Correct
HappinesTone 4In isolation Correct

4	1	Male	/fa/	2	Happines	Tone 4	In isolation	Correct	
4	2	Female	/pu/	1	Happines	Tone 4	In isolation	Incorrect	
4	2	Female	/pu/	2	Happines	Tone 4	In isolation	Incorrect	
4	1	Male	/pu/	1	Happines	Tone 4	In isolation	Correct	
4	1	Male	/pu/	2	Happines	Tone 4	In isolation	Correct	
4	2	Female	/ci/	1	Neutral	Tone 1	In isolation	Correct	
4	2	Female	/ci/	2	Neutral	Tone 1	In isolation	Correct	
4	1	Male	/ci/	1	Neutral	Tone 1	In isolation	Correct	
4	1	Male	/ci/	2	Neutral	Tone 1	In isolation	Correct	
4	2	Female	/fa/	1	Neutral	Tone 1	In isolation	Correct	
4	2	Female	/fa/	2	Neutral	Tone 1	In isolation	Correct	
4	1	Male	/fa/	1	Neutral	Tone 1	In isolation	Correct	
4	1	Male	/fa/	2	Neutral	Tone 1	In isolation	Correct	
4	2	Female	/pu/	1	Neutral	Tone 1	In isolation	Correct	
4	2	Female	/pu/	2	Neutral	Tone 1	In isolation	Correct	
4	1	Male	/pu/	1	Neutral	Tone 1	In isolation	Correct	
4	1	Male	/pu/	2	Neutral	Tone 1	In isolation	Correct	
4	2	Female	/ci/	1	Neutral	Tone 2	In isolation	Correct	
4	2	Female	/ci/	2	Neutral	Tone 2	In isolation	Correct	
4	1	Male	/ci/	1	Neutral	Tone 2	In isolation	Correct	
4	1	Male	/ci/	2	Neutral	Tone 2	In isolation	Correct	
4	2	Female	/fa/	1	Neutral	Tone 2	In isolation	Correct	
4	2	Female	/fa/	2	Neutral	Tone 2	In isolation	Correct	
4	1	Male	/fa/	1	Neutral	Tone 2	In isolation	Correct	
4	1	Male	/fa/	2	Neutral	Tone 2	In isolation	Correct	
4	2	Female	/pu/	1	Neutral	Tone 2	In isolation	Correct	
4	2	Female	/pu/	2	Neutral	Tone 2	In isolation	Correct	
4	1	Male	/pu/	1	Neutral	Tone 2	In isolation	Correct	
4	1	Male	/pu/	2	Neutral	Tone 2	In isolation	Correct	
4	2	Female	/ci/	1	Neutral	Tone 3	In isolation	Correct	
4	2	Female	/ci/	2	Neutral	Tone 3	In isolation	Correct	
4	1	Male	/ci/	1	Neutral	Tone 3	In isolation	Correct	
4	1	Male	/ci/	2	Neutral	Tone 3	In isolation	Correct	
4	2	Female	/fa/	1	Neutral	Tone 3	In isolation	Correct	
4	2	Female	/fa/	2	Neutral	Tone 3	In isolation	Correct	
4	1	Male	/fa/	1	Neutral	Tone 3	In isolation	Correct	
4	1	Male	/fa/	2	Neutral	Tone 3	In isolation	Correct	
4	2	Female	/pu/	1	Neutral	Tone 3	In isolation	Correct	
4	2	Female	/pu/	2	Neutral	Tone 3	In isolation	Correct	
4	1	Male	/pu/	1	Neutral	Tone 3	In isolation	Correct	
4	1	Male	/pu/	2	Neutral	Tone 3	In isolation	Correct	
4	2	Female	/ci/	1	Neutral	Tone 4	In isolation	Correct	
4	2	Female	/ci/	2	Neutral	Tone 4	In isolation	Correct	
4	1	Male	/ci/	1	Neutral	Tone 4	In isolation	Correct	
4	1	Male	/ci/	2	Neutral	Tone 4	In isolation	Correct	
4	2	Female	/fa/	1	Neutral	Tone 4	In isolation	Correct	
4	2	Female	/fa/	2	Neutral	Tone 4	In isolation	Correct	
4	1	Male	/fa/	1	Neutral	Tone 4	In isolation	Correct	
4	1	Male	/fa/	2	Neutral	Tone 4	In isolation	Correct	
4	2	Female	/pu/	1	Neutral	Tone 4	In isolation	Correct	
4	2	Female	/pu/	2	Neutral	Tone 4	In isolation	Correct	

4	1	Male	/pu/	1	Neutral	Tone 4	In isolation	Correct	
4	1	Male	/pu/	2	Neutral	Tone 4	In isolation	Correct	
4	2	Female	/ci/	1	Sadness	Tone 1	In isolation	Correct	
4	2	Female	/ci/	2	Sadness	Tone 1	In isolation	Correct	
4	1	Male	/ci/	1	Sadness	Tone 1	In isolation	Incorrect	
4	1	Male	/ci/	2	Sadness	Tone 1	In isolation	Incorrect	
4	2	Female	/fa/	1	Sadness	Tone 1	In isolation	Correct	
4	2	Female	/fa/	2	Sadness	Tone 1	In isolation	Correct	
4	1	Male	/fa/	1	Sadness	Tone 1	In isolation	Correct	
4	1	Male	/fa/	2	Sadness	Tone 1	In isolation	Correct	
4	2	Female	/pu/	1	Sadness	Tone 1	In isolation	Correct	
4	2	Female	/pu/	2	Sadness	Tone 1	In isolation	Correct	
4	1	Male	/pu/	1	Sadness	Tone 1	In isolation	Correct	
4	1	Male	/pu/	2	Sadness	Tone 1	In isolation	Correct	
4	2	Female	/ci/	1	Sadness	Tone 2	In isolation	Incorrect	
4	2	Female	/ci/	2	Sadness	Tone 2	In isolation	Incorrect	
4	1	Male	/ci/	1	Sadness	Tone 2	In isolation	Correct	
4	1	Male	/ci/	2	Sadness	Tone 2	In isolation	Correct	
4	2	Female	/fa/	1	Sadness	Tone 2	In isolation	Correct	
4	2	Female	/fa/	2	Sadness	Tone 2	In isolation	Correct	
4	1	Male	/fa/	1	Sadness	Tone 2	In isolation	Incorrect	
4	1	Male	/fa/	2	Sadness	Tone 2	In isolation	Correct	
4	2	Female	/pu/	1	Sadness	Tone 2	In isolation	Incorrect	
4	2	Female	/pu/	2	Sadness	Tone 2	In isolation	Incorrect	
4	1	Male	/pu/	1	Sadness	Tone 2	In isolation	Incorrect	
4	1	Male	/pu/	2	Sadness	Tone 2	In isolation	Correct	
4	2	Female	/ci/	1	Sadness	Tone 3	In isolation	Incorrect	
4	2	Female	/ci/	2	Sadness	Tone 3	In isolation	Incorrect	
4	1	Male	/ci/	1	Sadness	Tone 3	In isolation	Incorrect	
4	1	Male	/ci/	2	Sadness	Tone 3	In isolation	Incorrect	
4	2	Female	/fa/	1	Sadness	Tone 3	In isolation	Correct	
4	2	Female	/fa/	2	Sadness	Tone 3	In isolation	Correct	
4	1	Male	/fa/	1	Sadness	Tone 3	In isolation	Correct	
4	1	Male	/fa/	2	Sadness	Tone 3	In isolation	Correct	
4	2	Female	/pu/	1	Sadness	Tone 3	In isolation	Incorrect	
4	2	Female	/pu/	2	Sadness	Tone 3	In isolation	Incorrect	
4	1	Male	/pu/	1	Sadness	Tone 3	In isolation	Incorrect	
4	1	Male	/pu/	2	Sadness	Tone 3	In isolation	Correct	
4	2	Female	/ci/	1	Sadness	Tone 4	In isolation	Correct	
4	2	Female	/ci/	2	Sadness	Tone 4	In isolation	Correct	
4	1	Male	/ci/	1	Sadness	Tone 4	In isolation	Correct	
4	1	Male	/ci/	2	Sadness	Tone 4	In isolation	Correct	
4	2	Female	/fa/	1	Sadness	Tone 4	In isolation	Correct	
4	2	Female	/fa/	2	Sadness	Tone 4	In isolation	Incorrect	
4	1	Male	/fa/	1	Sadness	Tone 4	In isolation	Correct	
4	1	Male	/fa/	2	Sadness	Tone 4	In isolation	Correct	
4	2	Female	/pu/	1	Sadness	Tone 4	In isolation	Correct	
4	2	Female	/pu/	2	Sadness	Tone 4	In isolation	Correct	
4	1	Male	/pu/	1	Sadness	Tone 4	In isolation	Correct	
4	1	Male	/pu/	2	Sadness	Tone 4	In isolation	Correct	
4	2	Female	/ci/	1	Anger	Tone 1	In context	Incorrect	

4	2	Female	/ci/	2	Anger	Tone 1	In context	Incorrect	
4	1	Male	/ci/	1	Anger	Tone 1	In context	Correct	
4	1	Male	/ci/	2	Anger	Tone 1	In context	Correct	
4	2	Female	/fa/	1	Anger	Tone 1	In context	Correct	
4	2	Female	/fa/	2	Anger	Tone 1	In context	Correct	
4	1	Male	/fa/	1	Anger	Tone 1	In context	Correct	
4	1	Male	/fa/	2	Anger	Tone 1	In context	Correct	
4	2	Female	/pu/	1	Anger	Tone 1	In context	Incorrect	
4	2	Female	/pu/	2	Anger	Tone 1	In context	Incorrect	
4	1	Male	/pu/	1	Anger	Tone 1	In context	Correct	
4	1	Male	/pu/	2	Anger	Tone 1	In context	Correct	
4	2	Female	/ci/	1	Anger	Tone 2	In context	Correct	
4	2	Female	/ci/	2	Anger	Tone 2	In context	Correct	
4	1	Male	/ci/	1	Anger	Tone 2	In context	Correct	
4	1	Male	/ci/	2	Anger	Tone 2	In context	Correct	
4	2	Female	/fa/	1	Anger	Tone 2	In context	Correct	
4	2	Female	/fa/	2	Anger	Tone 2	In context	Correct	
4	1	Male	/fa/	1	Anger	Tone 2	In context	Correct	
4	1	Male	/fa/	2	Anger	Tone 2	In context	Correct	
4	2	Female	/pu/	1	Anger	Tone 2	In context	Correct	
4	2	Female	/pu/	2	Anger	Tone 2	In context	Correct	
4	1	Male	/pu/	1	Anger	Tone 2	In context	Correct	
4	1	Male	/pu/	2	Anger	Tone 2	In context	Correct	
4	2	Female	/ci/	1	Anger	Tone 3	In context	Correct	
4	2	Female	/ci/	2	Anger	Tone 3	In context	Correct	
4	1	Male	/ci/	1	Anger	Tone 3	In context	Correct	
4	1	Male	/ci/	2	Anger	Tone 3	In context	Correct	
4	2	Female	/fa/	1	Anger	Tone 3	In context	Correct	
4	2	Female	/fa/	2	Anger	Tone 3	In context	Correct	
4	1	Male	/fa/	1	Anger	Tone 3	In context	Correct	
4	1	Male	/fa/	2	Anger	Tone 3	In context	Correct	
4	2	Female	/pu/	1	Anger	Tone 3	In context	Correct	
4	2	Female	/pu/	2	Anger	Tone 3	In context	Correct	
4	1	Male	/pu/	1	Anger	Tone 3	In context	Correct	
4	1	Male	/pu/	2	Anger	Tone 3	In context	Correct	
4	2	Female	/ci/	1	Anger	Tone 4	In context	Correct	
4	2	Female	/ci/	2	Anger	Tone 4	In context	Correct	
4	1	Male	/ci/	1	Anger	Tone 4	In context	Correct	
4	1	Male	/ci/	2	Anger	Tone 4	In context	Correct	
4	2	Female	/fa/	1	Anger	Tone 4	In context	Correct	
4	2	Female	/fa/	2	Anger	Tone 4	In context	Correct	
4	1	Male	/fa/	1	Anger	Tone 4	In context	Correct	
4	1	Male	/fa/	2	Anger	Tone 4	In context	Correct	
4	2	Female	/pu/	1	Anger	Tone 4	In context	Correct	
4	2	Female	/pu/	2	Anger	Tone 4	In context	Correct	
4	1	Male	/pu/	1	Anger	Tone 4	In context	Correct	
4	1	Male	/pu/	2	Anger	Tone 4	In context	Correct	
4	2	Female	/ci/	1	Fear	Tone 1	In context	Correct	
4	2	Female	/ci/	2	Fear	Tone 1	In context	Correct	
4	1	Male	/ci/	1	Fear	Tone 1	In context	Correct	
4	1	Male	/ci/	2	Fear	Tone 1	In context	Correct	

4	2	Female	/fa/	1	Fear	Tone 1	In context	Correct	
4	2	Female	/fa/	2	Fear	Tone 1	In context	Correct	
4	1	Male	/fa/	1	Fear	Tone 1	In context	Correct	
4	1	Male	/fa/	2	Fear	Tone 1	In context	Correct	
4	2	Female	/pu/	1	Fear	Tone 1	In context	Incorrect	
4	2	Female	/pu/	2	Fear	Tone 1	In context	Correct	
4	1	Male	/pu/	1	Fear	Tone 1	In context	Correct	
4	1	Male	/pu/	2	Fear	Tone 1	In context	Correct	
4	2	Female	/ci/	1	Fear	Tone 2	In context	Correct	
4	2	Female	/ci/	2	Fear	Tone 2	In context	Incorrect	
4	1	Male	/ci/	1	Fear	Tone 2	In context	Correct	
4	1	Male	/ci/	2	Fear	Tone 2	In context	Correct	
4	2	Female	/fa/	1	Fear	Tone 2	In context	Correct	
4	2	Female	/fa/	2	Fear	Tone 2	In context	Correct	
4	1	Male	/fa/	1	Fear	Tone 2	In context	Correct	
4	1	Male	/fa/	2	Fear	Tone 2	In context	Correct	
4	2	Female	/pu/	1	Fear	Tone 2	In context	Correct	
4	2	Female	/pu/	2	Fear	Tone 2	In context	Correct	
4	1	Male	/pu/	1	Fear	Tone 2	In context	Correct	
4	1	Male	/pu/	2	Fear	Tone 2	In context	Correct	
4	2	Female	/ci/	1	Fear	Tone 3	In context	Incorrect	
4	2	Female	/ci/	2	Fear	Tone 3	In context	Incorrect	
4	1	Male	/ci/	1	Fear	Tone 3	In context	Correct	
4	1	Male	/ci/	2	Fear	Tone 3	In context	Correct	
4	2	Female	/fa/	1	Fear	Tone 3	In context	Correct	
4	2	Female	/fa/	2	Fear	Tone 3	In context	Correct	
4	1	Male	/fa/	1	Fear	Tone 3	In context	Correct	
4	1	Male	/fa/	2	Fear	Tone 3	In context	Correct	
4	2	Female	/pu/	1	Fear	Tone 3	In context	Correct	
4	2	Female	/pu/	2	Fear	Tone 3	In context	Incorrect	
4	1	Male	/pu/	1	Fear	Tone 3	In context	Correct	
4	1	Male	/pu/	2	Fear	Tone 3	In context	Correct	
4	2	Female	/ci/	1	Fear	Tone 4	In context	Correct	
4	2	Female	/ci/	2	Fear	Tone 4	In context	Correct	
4	1	Male	/ci/	1	Fear	Tone 4	In context	Correct	
4	1	Male	/ci/	2	Fear	Tone 4	In context	Correct	
4	2	Female	/fa/	1	Fear	Tone 4	In context	Correct	
4	2	Female	/fa/	2	Fear	Tone 4	In context	Correct	
4	1	Male	/fa/	1	Fear	Tone 4	In context	Correct	
4	1	Male	/fa/	2	Fear	Tone 4	In context	Correct	
4	2	Female	/pu/	1	Fear	Tone 4	In context	Correct	
4	2	Female	/pu/	2	Fear	Tone 4	In context	Correct	
4	1	Male	/pu/	1	Fear	Tone 4	In context	Correct	
4	1	Male	/pu/	2	Fear	Tone 4	In context	Correct	
4	2	Female	/ci/	1	Happines	Tone 1	In context	Correct	
4	2	Female	/ci/	2	Happines	Tone 1	In context	Correct	
4	1	Male	/ci/	1	Happines	Tone 1	In context	Correct	
4	1	Male	/ci/	2	Happines	Tone 1	In context	Correct	
4	2	Female	/fa/	1	Happines	Tone 1	In context	Correct	
4	2	Female	/fa/	2	Happines	Tone 1	In context	Correct	
4	1	Male	/fa/	1	Happines	Tone 1	In context	Correct	

4	1	Male
4	2	Female
4	2	Female
4	1	Male
4	1	Male
4	2	Female
/fa/
/pu/
/pu/
/pu/
/pu/
/ci/
HappinesTone 1 In context
HappinesTone 1 In context
HappinesTone 1 In context
HappinesTone 1 In context
HappinesTone 1 In context
HappinesTone 2 In context
Correct Correct Correct Correct Correct Correct

4	2	Female
/ci/
HappinesTone 2 In context Incorrect

4	1	Male
4	1	Male
4	2	Female
4	2	Female
4	1	Male
4	1	Male
/ci/
/ci/
/fa/
/fa/
/fa/
/fa/
HappinesTone 2 In context
HappinesTone 2 In context
HappinesTone 2 In context
HappinesTone 2 In context
HappinesTone 2 In context
HappinesTone 2 In context
Correct Correct Correct Correct Correct Correct

4	2	Female
4	2	Female
/pu/
/pu/
HappinesTone 2 In context Incorrect
HappinesTone 2 In context Incorrect

4	1	Male
4	1	Male
4	2	Female
4	2	Female
4	1	Male
4	1	Male
4	2	Female
4	2	Female
4	1	Male
4	1	Male
4	2	Female
4	2	Female
4	1	Male
4	1	Male
4	2	Female
4	2	Female
4	1	Male
4	1	Male
4	2	Female
4	2	Female
4	1	Male
4	1	Male
4	2	Female
4	2	Female
4	1	Male
4	1	Male
/pu/
/pu/
/ci/
/ci/
/ci/
/ci/
/fa/
/fa/
/fa/
/fa/
/pu/
/pu/
/pu/
/pu/
/ci/
/ci/
/ci/
/ci/
/fa/
/fa/
/fa/
/fa/
/pu/
/pu/
/pu/
/pu/
HappinesTone 2 In context
HappinesTone 2 In context
HappinesTone 3 In context
HappinesTone 3 In context
HappinesTone 3 In context
HappinesTone 3 In context
HappinesTone 3 In context
HappinesTone 3 In context
HappinesTone 3 In context
HappinesTone 3 In context
HappinesTone 3 In context
HappinesTone 3 In context
HappinesTone 3 In context
HappinesTone 3 In context
HappinesTone 4 In context
HappinesTone 4 In context
HappinesTone 4 In context
HappinesTone 4 In context
HappinesTone 4 In context
HappinesTone 4 In context
HappinesTone 4 In context
HappinesTone 4 In context
HappinesTone 4 In context
HappinesTone 4 In context
HappinesTone 4 In context
HappinesTone 4 In context
Correct Correct Correct Correct Correct Correct Correct Correct Correct Correct Correct Correct Correct Correct Correct Correct Correct Correct Correct Correct Correct Correct Correct Correct Correct Correct

4	2	Female
4	2	Female
4	1	Male
4	1	Male
4	2	Female
4	2	Female
4	1	Male
4	1	Male
4	2	Female
4	2	Female
/ci/
/ci/
/ci/
/ci/
/fa/
/fa/
/fa/
/fa/
/pu/
/pu/
Neutral Tone 1 In context
Neutral Tone 1 In context
Neutral Tone 1 In context
Neutral Tone 1 In context
Neutral Tone 1 In context
Neutral Tone 1 In context
Neutral Tone 1 In context
Neutral Tone 1 In context
Neutral Tone 1 In context
Neutral Tone 1 In context
Correct
Correct Correct Correct Correct Correct Correct Correct Correct Correct

4	1	Male	/pu/	1	Neutral	Tone 1	In context	Correct	
4	1	Male	/pu/	2	Neutral	Tone 1	In context	Correct	
4	2	Female	/ci/	1	Neutral	Tone 2	In context	Correct	
4	2	Female	/ci/	2	Neutral	Tone 2	In context	Correct	
4	1	Male	/ci/	1	Neutral	Tone 2	In context	Correct	
4	1	Male	/ci/	2	Neutral	Tone 2	In context	Correct	
4	2	Female	/fa/	1	Neutral	Tone 2	In context	Correct	
4	2	Female	/fa/	2	Neutral	Tone 2	In context	Correct	
4	1	Male	/fa/	1	Neutral	Tone 2	In context	Correct	
4	1	Male	/fa/	2	Neutral	Tone 2	In context	Correct	
4	2	Female	/pu/	1	Neutral	Tone 2	In context	Correct	
4	2	Female	/pu/	2	Neutral	Tone 2	In context	Correct	
4	1	Male	/pu/	1	Neutral	Tone 2	In context	Correct	
4	1	Male	/pu/	2	Neutral	Tone 2	In context	Correct	
4	2	Female	/ci/	1	Neutral	Tone 3	In context	Correct	
4	2	Female	/ci/	2	Neutral	Tone 3	In context	Correct	
4	1	Male	/ci/	1	Neutral	Tone 3	In context	Correct	
4	1	Male	/ci/	2	Neutral	Tone 3	In context	Correct	
4	2	Female	/fa/	1	Neutral	Tone 3	In context	Correct	
4	2	Female	/fa/	2	Neutral	Tone 3	In context	Correct	
4	1	Male	/fa/	1	Neutral	Tone 3	In context	Correct	
4	1	Male	/fa/	2	Neutral	Tone 3	In context	Correct	
4	2	Female	/pu/	1	Neutral	Tone 3	In context	Correct	
4	2	Female	/pu/	2	Neutral	Tone 3	In context	Correct	
4	1	Male	/pu/	1	Neutral	Tone 3	In context	Correct	
4	1	Male	/pu/	2	Neutral	Tone 3	In context	Correct	
4	2	Female	/ci/	1	Neutral	Tone 4	In context	Correct	
4	2	Female	/ci/	2	Neutral	Tone 4	In context	Correct	
4	1	Male	/ci/	1	Neutral	Tone 4	In context	Correct	
4	1	Male	/ci/	2	Neutral	Tone 4	In context	Correct	
4	2	Female	/fa/	1	Neutral	Tone 4	In context	Correct	
4	2	Female	/fa/	2	Neutral	Tone 4	In context	Correct	
4	1	Male	/fa/	1	Neutral	Tone 4	In context	Correct	
4	1	Male	/fa/	2	Neutral	Tone 4	In context	Correct	
4	2	Female	/pu/	1	Neutral	Tone 4	In context	Correct	
4	2	Female	/pu/	2	Neutral	Tone 4	In context	Correct	
4	1	Male	/pu/	1	Neutral	Tone 4	In context	Correct	
4	1	Male	/pu/	2	Neutral	Tone 4	In context	Correct	
4	2	Female	/ci/	1	Sadness	Tone 1	In context	Correct	
4	2	Female	/ci/	2	Sadness	Tone 1	In context	Correct	
4	1	Male	/ci/	1	Sadness	Tone 1	In context	Correct	
4	1	Male	/ci/	2	Sadness	Tone 1	In context	Correct	
4	2	Female	/fa/	1	Sadness	Tone 1	In context	Correct	
4	2	Female	/fa/	2	Sadness	Tone 1	In context	Correct	
4	1	Male	/fa/	1	Sadness	Tone 1	In context	Correct	
4	1	Male	/fa/	2	Sadness	Tone 1	In context	Correct	
4	2	Female	/pu/	1	Sadness	Tone 1	In context	Correct	
4	2	Female	/pu/	2	Sadness	Tone 1	In context	Correct	
4	1	Male	/pu/	1	Sadness	Tone 1	In context	Correct	
4	1	Male	/pu/	2	Sadness	Tone 1	In context	Correct	
4	2	Female	/ci/	1	Sadness	Tone 2	In context	Correct	

4	2	Female	/ci/	2	Sadness	Tone 2	In context	Incorrect	
4	1	Male	/ci/	1	Sadness	Tone 2	In context	Correct	
4	1	Male	/ci/	2	Sadness	Tone 2	In context	Correct	
4	2	Female	/fa/	1	Sadness	Tone 2	In context	Correct	
4	2	Female	/fa/	2	Sadness	Tone 2	In context	Correct	
4	1	Male	/fa/	1	Sadness	Tone 2	In context	Correct	
4	1	Male	/fa/	2	Sadness	Tone 2	In context	Correct	
4	2	Female	/pu/	1	Sadness	Tone 2	In context	Incorrect	
4	2	Female	/pu/	2	Sadness	Tone 2	In context	Correct	
4	1	Male	/pu/	1	Sadness	Tone 2	In context	Correct	
4	1	Male	/pu/	2	Sadness	Tone 2	In context	Correct	
4	2	Female	/ci/	1	Sadness	Tone 3	In context	Correct	
4	2	Female	/ci/	2	Sadness	Tone 3	In context	Correct	
4	1	Male	/ci/	1	Sadness	Tone 3	In context	Correct	
4	1	Male	/ci/	2	Sadness	Tone 3	In context	Correct	
4	2	Female	/fa/	1	Sadness	Tone 3	In context	Correct	
4	2	Female	/fa/	2	Sadness	Tone 3	In context	Correct	
4	1	Male	/fa/	1	Sadness	Tone 3	In context	Correct	
4	1	Male	/fa/	2	Sadness	Tone 3	In context	Correct	
4	2	Female	/pu/	1	Sadness	Tone 3	In context	Correct	
4	2	Female	/pu/	2	Sadness	Tone 3	In context	Correct	
4	1	Male	/pu/	1	Sadness	Tone 3	In context	Correct	
4	1	Male	/pu/	2	Sadness	Tone 3	In context	Correct	
4	2	Female	/ci/	1	Sadness	Tone 4	In context	Correct	
4	2	Female	/ci/	2	Sadness	Tone 4	In context	Correct	
4	1	Male	/ci/	1	Sadness	Tone 4	In context	Correct	
4	1	Male	/ci/	2	Sadness	Tone 4	In context	Correct	
4	2	Female	/fa/	1	Sadness	Tone 4	In context	Correct	
4	2	Female	/fa/	2	Sadness	Tone 4	In context	Correct	
4	1	Male	/fa/	1	Sadness	Tone 4	In context	Correct	
4	1	Male	/fa/	2	Sadness	Tone 4	In context	Correct	
4	2	Female	/pu/	1	Sadness	Tone 4	In context	Incorrect	
4	2	Female	/pu/	2	Sadness	Tone 4	In context	Correct	
4	1	Male	/pu/	1	Sadness	Tone 4	In context	Correct	
4	1	Male	/pu/	2	Sadness	Tone 4	In context	Correct	
5	2	Female	/ci/	1	Anger	Tone 1	In isolation	Incorrect	
5	2	Female	/ci/	2	Anger	Tone 1	In isolation	Correct	
5	1	Male	/ci/	1	Anger	Tone 1	In isolation	Correct	
5	1	Male	/ci/	2	Anger	Tone 1	In isolation	Correct	
5	2	Female	/fa/	1	Anger	Tone 1	In isolation	Correct	
5	2	Female	/fa/	2	Anger	Tone 1	In isolation	Correct	
5	1	Male	/fa/	1	Anger	Tone 1	In isolation	Correct	
5	1	Male	/fa/	2	Anger	Tone 1	In isolation	Correct	
5	2	Female	/pu/	1	Anger	Tone 1	In isolation	Incorrect	
5	2	Female	/pu/	2	Anger	Tone 1	In isolation	Correct	
5	1	Male	/pu/	1	Anger	Tone 1	In isolation	Correct	
5	1	Male	/pu/	2	Anger	Tone 1	In isolation	Correct	
5	2	Female	/ci/	1	Anger	Tone 2	In isolation	Incorrect	
5	2	Female	/ci/	2	Anger	Tone 2	In isolation	Incorrect	
5	1	Male	/ci/	1	Anger	Tone 2	In isolation	Incorrect	
5	1	Male	/ci/	2	Anger	Tone 2	In isolation	Incorrect	

5	2	Female	/fa/	1	Anger	Tone 2	In isolation	Correct	
5	2	Female	/fa/	2	Anger	Tone 2	In isolation	Correct	
5	1	Male	/fa/	1	Anger	Tone 2	In isolation	Correct	
5	1	Male	/fa/	2	Anger	Tone 2	In isolation	Correct	
5	2	Female	/pu/	1	Anger	Tone 2	In isolation	Correct	
5	2	Female	/pu/	2	Anger	Tone 2	In isolation	Correct	
5	1	Male	/pu/	1	Anger	Tone 2	In isolation	Correct	
5	1	Male	/pu/	2	Anger	Tone 2	In isolation	Correct	
5	2	Female	/ci/	1	Anger	Tone 3	In isolation	Incorrect	
5	2	Female	/ci/	2	Anger	Tone 3	In isolation	Incorrect	
5	1	Male	/ci/	1	Anger	Tone 3	In isolation	Incorrect	
5	1	Male	/ci/	2	Anger	Tone 3	In isolation	Correct	
5	2	Female	/fa/	1	Anger	Tone 3	In isolation	Incorrect	
5	2	Female	/fa/	2	Anger	Tone 3	In isolation	Incorrect	
5	1	Male	/fa/	1	Anger	Tone 3	In isolation	Correct	
5	1	Male	/fa/	2	Anger	Tone 3	In isolation	Correct	
5	2	Female	/pu/	1	Anger	Tone 3	In isolation	Incorrect	
5	2	Female	/pu/	2	Anger	Tone 3	In isolation	Incorrect	
5	1	Male	/pu/	1	Anger	Tone 3	In isolation	Incorrect	
5	1	Male	/pu/	2	Anger	Tone 3	In isolation	Correct	
5	2	Female	/ci/	1	Anger	Tone 4	In isolation	Correct	
5	2	Female	/ci/	2	Anger	Tone 4	In isolation	Correct	
5	1	Male	/ci/	1	Anger	Tone 4	In isolation	Correct	
5	1	Male	/ci/	2	Anger	Tone 4	In isolation	Correct	
5	2	Female	/fa/	1	Anger	Tone 4	In isolation	Correct	
5	2	Female	/fa/	2	Anger	Tone 4	In isolation	Correct	
5	1	Male	/fa/	1	Anger	Tone 4	In isolation	Correct	
5	1	Male	/fa/	2	Anger	Tone 4	In isolation	Correct	
5	2	Female	/pu/	1	Anger	Tone 4	In isolation	Incorrect	
5	2	Female	/pu/	2	Anger	Tone 4	In isolation	Correct	
5	1	Male	/pu/	1	Anger	Tone 4	In isolation	Correct	
5	1	Male	/pu/	2	Anger	Tone 4	In isolation	Correct	
5	2	Female	/ci/	1	Fear	Tone 1	In isolation	Correct	
5	2	Female	/ci/	2	Fear	Tone 1	In isolation	Correct	
5	1	Male	/ci/	1	Fear	Tone 1	In isolation	Correct	
5	1	Male	/ci/	2	Fear	Tone 1	In isolation	Correct	
5	2	Female	/fa/	1	Fear	Tone 1	In isolation	Correct	
5	2	Female	/fa/	2	Fear	Tone 1	In isolation	Correct	
5	1	Male	/fa/	1	Fear	Tone 1	In isolation	Correct	
5	1	Male	/fa/	2	Fear	Tone 1	In isolation	Correct	
5	2	Female	/pu/	1	Fear	Tone 1	In isolation	Correct	
5	2	Female	/pu/	2	Fear	Tone 1	In isolation	Correct	
5	1	Male	/pu/	1	Fear	Tone 1	In isolation	Correct	
5	1	Male	/pu/	2	Fear	Tone 1	In isolation	Correct	
5	2	Female	/ci/	1	Fear	Tone 2	In isolation	Incorrect	
5	2	Female	/ci/	2	Fear	Tone 2	In isolation	Incorrect	
5	1	Male	/ci/	1	Fear	Tone 2	In isolation	Incorrect	
5	1	Male	/ci/	2	Fear	Tone 2	In isolation	Incorrect	
5	2	Female	/fa/	1	Fear	Tone 2	In isolation	Incorrect	
5	2	Female	/fa/	2	Fear	Tone 2	In isolation	Incorrect	
5	1	Male	/fa/	1	Fear	Tone 2	In isolation	Correct	

5	1	Male
5	2	Female
5	2	Female
5	1	Male
5	1	Male
5	2	Female
5	2	Female
5	1	Male
5	1	Male
5	2	Female
5	2	Female
5	1	Male
5	1	Male
5	2	Female
5	2	Female
5	1	Male
5	1	Male
5	2	Female
5	2	Female
5	1	Male
5	1	Male
5	2	Female
5	2	Female
5	1	Male
5	1	Male
5	2	Female
5	2	Female
5	1	Male
5	1	Male
/fa/
/pu/
/pu/
/pu/
/pu/
/ci/
/ci/
/ci/
/ci/
/fa/
/fa/
/fa/
/fa/
/pu/
/pu/
/pu/
/pu/
/ci/
/ci/
/ci/
/ci/
/fa/
/fa/
/fa/
/fa/
/pu/
/pu/
/pu/
/pu/
2	Fear
Fear
Fear
Fear
Fear
Fear
Fear
Fear
Fear
Fear
Fear
Fear
Fear
Fear
Fear
Fear
Fear
Fear
Fear
Fear
Fear
Fear
Fear
Fear
Fear
Fear
Fear
Fear
Fear
Tone 2In isolation Correct Tone 2In isolation Correct Tone 2In isolation Incorrect Tone 2In isolation Incorrect Tone 2In isolation Incorrect Tone 3In isolation Incorrect Tone 3In isolation Incorrect Tone 3In isolation Correct Tone 3In isolation Correct Tone 3In isolation Incorrect Tone 3In isolation Incorrect Tone 3In isolation Correct Tone 3In isolation Correct Tone 3In isolation Incorrect Tone 3In isolation Incorrect Tone 3In isolation Incorrect Tone 3In isolation Incorrect Tone 4In isolation Correct Tone 4In isolation Correct Tone 4In isolation Correct Tone 4In isolation Correct Tone 4In isolation Correct Tone 4In isolation Correct Tone 4In isolation Correct Tone 4In isolation Incorrect Tone 4In isolation Correct Tone 4In isolation Correct Tone 4In isolation Correct Tone 4In isolation Correct

5	2	Female
5	2	Female
5	1	Male
5	1	Male
5	2	Female
5	2	Female
5	1	Male
5	1	Male
5	2	Female
5	2	Female
5	1	Male
5	1	Male
5	2	Female
5	2	Female
5	1	Male
5	1	Male
5	2	Female
5	2	Female
5	1	Male
5	1	Male
5	2	Female
5	2	Female
/ci/
/ci/
/ci/
/ci/
/fa/
/fa/
/fa/
/fa/
/pu/
/pu/
/pu/
/pu/
/ci/
/ci/
/ci/
/ci/
/fa/
/fa/
/fa/
/fa/
/pu/
/pu/
HappinesTone 1In isolation Correct
HappinesTone 1In isolation Incorrect
HappinesTone 1In isolation Correct
HappinesTone 1In isolation Correct
HappinesTone 1In isolation Incorrect
HappinesTone 1In isolation Incorrect
HappinesTone 1In isolation Correct
HappinesTone 1In isolation Correct
HappinesTone 1In isolation Incorrect
HappinesTone 1In isolation Incorrect
HappinesTone 1In isolation Correct
HappinesTone 1In isolation Correct
HappinesTone 2In isolation Correct
HappinesTone 2In isolation Correct
HappinesTone 2In isolation Incorrect
HappinesTone 2In isolation Incorrect
HappinesTone 2In isolation Correct
HappinesTone 2In isolation Correct
HappinesTone 2In isolation Correct
HappinesTone 2In isolation Correct
HappinesTone 2In isolation Correct
HappinesTone 2In isolation Correct

5	1	Male
5	1	Male
5	2	Female
5	2	Female
5	1	Male
5	1	Male
5	2	Female
5	2	Female
5	1	Male
5	1	Male
5	2	Female
5	2	Female
5	1	Male
5	1	Male
5	2	Female
5	2	Female
5	1	Male
5	1	Male
5	2	Female
5	2	Female
5	1	Male
5	1	Male
5	2	Female
5	2	Female
5	1	Male
5	1	Male
/pu/
/pu/
/ci/
/ci/
/ci/
/ci/
/fa/
/fa/
/fa/
/fa/
/pu/
/pu/
/pu/
/pu/
/ci/
/ci/
/ci/
/ci/
/fa/
/fa/
/fa/
/fa/
/pu/
/pu/
/pu/
/pu/
HappinesTone 2In isolation Correct
HappinesTone 2In isolation Incorrect
HappinesTone 3In isolation Incorrect
HappinesTone 3In isolation Incorrect
HappinesTone 3In isolation Correct
HappinesTone 3In isolation Correct
HappinesTone 3In isolation Correct
HappinesTone 3In isolation Incorrect
HappinesTone 3In isolation Correct
HappinesTone 3In isolation Correct
HappinesTone 3In isolation Incorrect
HappinesTone 3In isolation Incorrect
HappinesTone 3In isolation Incorrect
HappinesTone 3In isolation Correct
HappinesTone 4In isolation Incorrect
HappinesTone 4In isolation Incorrect
HappinesTone 4In isolation Correct
HappinesTone 4In isolation Correct
HappinesTone 4In isolation Incorrect
HappinesTone 4In isolation Correct
HappinesTone 4In isolation Correct
HappinesTone 4In isolation Correct
HappinesTone 4In isolation Incorrect
HappinesTone 4In isolation Incorrect
HappinesTone 4In isolation Correct
HappinesTone 4In isolation Correct

5	2	Female
5	2	Female
5	1	Male
5	1	Male
5	2	Female
5	2	Female
5	1	Male
5	1	Male
5	2	Female
5	2	Female
5	1	Male
5	1	Male
5	2	Female
5	2	Female
5	1	Male
5	1	Male
5	2	Female
5	2	Female
5	1	Male
5	1	Male
5	2	Female
5	2	Female
5	1	Male
5	1	Male
5	2	Female
/ci/
/ci/
/ci/
/ci/
/fa/
/fa/
/fa/
/fa/
/pu/
/pu/
/pu/
/pu/
/ci/
/ci/
/ci/
/ci/
/fa/
/fa/
/fa/
/fa/
/pu/
/pu/
/pu/
/pu/
/ci/
Neutral Tone 1In isolation Correct
Neutral Tone 1In isolation Correct
Neutral Tone 1In isolation Correct
Neutral Tone 1In isolation Correct
Neutral Tone 1In isolation Correct
Neutral Tone 1In isolation Correct
Neutral Tone 1In isolation Correct
Neutral Tone 1In isolation Correct
Neutral Tone 1In isolation Correct
Neutral Tone 1In isolation Correct
Neutral Tone 1In isolation Correct
Neutral Tone 1In isolation Correct
Neutral Tone 2In isolation Correct
Neutral Tone 2In isolation Correct
Neutral Tone 2In isolation Correct
Neutral Tone 2In isolation Correct
Neutral Tone 2In isolation Correct
Neutral Tone 2In isolation Correct
Neutral Tone 2In isolation Correct
Neutral Tone 2In isolation Correct
Neutral Tone 2In isolation Correct
Neutral Tone 2In isolation Correct
Neutral Tone 2In isolation Correct
Neutral Tone 2In isolation Correct
1	Neutral Tone 3In isolation Correct

5	2	Female	/ci/	2	Neutral	Tone 3	In isolation	Correct	
5	1	Male	/ci/	1	Neutral	Tone 3	In isolation	Correct	
5	1	Male	/ci/	2	Neutral	Tone 3	In isolation	Correct	
5	2	Female	/fa/	1	Neutral	Tone 3	In isolation	Correct	
5	2	Female	/fa/	2	Neutral	Tone 3	In isolation	Correct	
5	1	Male	/fa/	1	Neutral	Tone 3	In isolation	Correct	
5	1	Male	/fa/	2	Neutral	Tone 3	In isolation	Correct	
5	2	Female	/pu/	1	Neutral	Tone 3	In isolation	Correct	
5	2	Female	/pu/	2	Neutral	Tone 3	In isolation	Correct	
5	1	Male	/pu/	1	Neutral	Tone 3	In isolation	Correct	
5	1	Male	/pu/	2	Neutral	Tone 3	In isolation	Correct	
5	2	Female	/ci/	1	Neutral	Tone 4	In isolation	Correct	
5	2	Female	/ci/	2	Neutral	Tone 4	In isolation	Correct	
5	1	Male	/ci/	1	Neutral	Tone 4	In isolation	Correct	
5	1	Male	/ci/	2	Neutral	Tone 4	In isolation	Correct	
5	2	Female	/fa/	1	Neutral	Tone 4	In isolation	Correct	
5	2	Female	/fa/	2	Neutral	Tone 4	In isolation	Correct	
5	1	Male	/fa/	1	Neutral	Tone 4	In isolation	Correct	
5	1	Male	/fa/	2	Neutral	Tone 4	In isolation	Correct	
5	2	Female	/pu/	1	Neutral	Tone 4	In isolation	Correct	
5	2	Female	/pu/	2	Neutral	Tone 4	In isolation	Correct	
5	1	Male	/pu/	1	Neutral	Tone 4	In isolation	Correct	
5	1	Male	/pu/	2	Neutral	Tone 4	In isolation	Correct	
5	2	Female	/ci/	1	Sadness	Tone 1	In isolation	Correct	
5	2	Female	/ci/	2	Sadness	Tone 1	In isolation	Correct	
5	1	Male	/ci/	1	Sadness	Tone 1	In isolation	Correct	
5	1	Male	/ci/	2	Sadness	Tone 1	In isolation	Correct	
5	2	Female	/fa/	1	Sadness	Tone 1	In isolation	Correct	
5	2	Female	/fa/	2	Sadness	Tone 1	In isolation	Incorrect	
5	1	Male	/fa/	1	Sadness	Tone 1	In isolation	Correct	
5	1	Male	/fa/	2	Sadness	Tone 1	In isolation	Correct	
5	2	Female	/pu/	1	Sadness	Tone 1	In isolation	Correct	
5	2	Female	/pu/	2	Sadness	Tone 1	In isolation	Correct	
5	1	Male	/pu/	1	Sadness	Tone 1	In isolation	Correct	
5	1	Male	/pu/	2	Sadness	Tone 1	In isolation	Correct	
5	2	Female	/ci/	1	Sadness	Tone 2	In isolation	Incorrect	
5	2	Female	/ci/	2	Sadness	Tone 2	In isolation	Incorrect	
5	1	Male	/ci/	1	Sadness	Tone 2	In isolation	Incorrect	
5	1	Male	/ci/	2	Sadness	Tone 2	In isolation	Incorrect	
5	2	Female	/fa/	1	Sadness	Tone 2	In isolation	Correct	
5	2	Female	/fa/	2	Sadness	Tone 2	In isolation	Correct	
5	1	Male	/fa/	1	Sadness	Tone 2	In isolation	Incorrect	
5	1	Male	/fa/	2	Sadness	Tone 2	In isolation	Correct	
5	2	Female	/pu/	1	Sadness	Tone 2	In isolation	Correct	
5	2	Female	/pu/	2	Sadness	Tone 2	In isolation	Correct	
5	1	Male	/pu/	1	Sadness	Tone 2	In isolation	Incorrect	
5	1	Male	/pu/	2	Sadness	Tone 2	In isolation	Correct	
5	2	Female	/ci/	1	Sadness	Tone 3	In isolation	Correct	
5	2	Female	/ci/	2	Sadness	Tone 3	In isolation	Incorrect	
5	1	Male	/ci/	1	Sadness	Tone 3	In isolation	Correct	
5	1	Male	/ci/	2	Sadness	Tone 3	In isolation	Correct	

5	2	Female	/fa/	1	Sadness	Tone 3	In isolation	Correct	
5	2	Female	/fa/	2	Sadness	Tone 3	In isolation	Correct	
5	1	Male	/fa/	1	Sadness	Tone 3	In isolation	Incorrect	
5	1	Male	/fa/	2	Sadness	Tone 3	In isolation	Correct	
5	2	Female	/pu/	1	Sadness	Tone 3	In isolation	Correct	
5	2	Female	/pu/	2	Sadness	Tone 3	In isolation	Incorrect	
5	1	Male	/pu/	1	Sadness	Tone 3	In isolation	Correct	
5	1	Male	/pu/	2	Sadness	Tone 3	In isolation	Correct	
5	2	Female	/ci/	1	Sadness	Tone 4	In isolation	Correct	
5	2	Female	/ci/	2	Sadness	Tone 4	In isolation	Correct	
5	1	Male	/ci/	1	Sadness	Tone 4	In isolation	Correct	
5	1	Male	/ci/	2	Sadness	Tone 4	In isolation	Correct	
5	2	Female	/fa/	1	Sadness	Tone 4	In isolation	Correct	
5	2	Female	/fa/	2	Sadness	Tone 4	In isolation	Incorrect	
5	1	Male	/fa/	1	Sadness	Tone 4	In isolation	Incorrect	
5	1	Male	/fa/	2	Sadness	Tone 4	In isolation	Correct	
5	2	Female	/pu/	1	Sadness	Tone 4	In isolation	Correct	
5	2	Female	/pu/	2	Sadness	Tone 4	In isolation	Correct	
5	1	Male	/pu/	1	Sadness	Tone 4	In isolation	Correct	
5	1	Male	/pu/	2	Sadness	Tone 4	In isolation	Incorrect	
5	2	Female	/ci/	1	Anger	Tone 1	In context	Correct	
5	2	Female	/ci/	2	Anger	Tone 1	In context	Correct	
5	1	Male	/ci/	1	Anger	Tone 1	In context	Correct	
5	1	Male	/ci/	2	Anger	Tone 1	In context	Correct	
5	2	Female	/fa/	1	Anger	Tone 1	In context	Correct	
5	2	Female	/fa/	2	Anger	Tone 1	In context	Correct	
5	1	Male	/fa/	1	Anger	Tone 1	In context	Correct	
5	1	Male	/fa/	2	Anger	Tone 1	In context	Correct	
5	2	Female	/pu/	1	Anger	Tone 1	In context	Correct	
5	2	Female	/pu/	2	Anger	Tone 1	In context	Incorrect	
5	1	Male	/pu/	1	Anger	Tone 1	In context	Correct	
5	1	Male	/pu/	2	Anger	Tone 1	In context	Correct	
5	2	Female	/ci/	1	Anger	Tone 2	In context	Correct	
5	2	Female	/ci/	2	Anger	Tone 2	In context	Correct	
5	1	Male	/ci/	1	Anger	Tone 2	In context	Correct	
5	1	Male	/ci/	2	Anger	Tone 2	In context	Correct	
5	2	Female	/fa/	1	Anger	Tone 2	In context	Correct	
5	2	Female	/fa/	2	Anger	Tone 2	In context	Correct	
5	1	Male	/fa/	1	Anger	Tone 2	In context	Correct	
5	1	Male	/fa/	2	Anger	Tone 2	In context	Correct	
5	2	Female	/pu/	1	Anger	Tone 2	In context	Correct	
5	2	Female	/pu/	2	Anger	Tone 2	In context	Correct	
5	1	Male	/pu/	1	Anger	Tone 2	In context	Correct	
5	1	Male	/pu/	2	Anger	Tone 2	In context	Correct	
5	2	Female	/ci/	1	Anger	Tone 3	In context	Correct	
5	2	Female	/ci/	2	Anger	Tone 3	In context	Correct	
5	1	Male	/ci/	1	Anger	Tone 3	In context	Correct	
5	1	Male	/ci/	2	Anger	Tone 3	In context	Correct	
5	2	Female	/fa/	1	Anger	Tone 3	In context	Correct	
5	2	Female	/fa/	2	Anger	Tone 3	In context	Correct	
5	1	Male	/fa/	1	Anger	Tone 3	In context	Correct	

5	1	Male	/fa/	2	Anger	Tone 3	In context	Correct	
5	2	Female	/pu/	1	Anger	Tone 3	In context	Correct	
5	2	Female	/pu/	2	Anger	Tone 3	In context	Correct	
5	1	Male	/pu/	1	Anger	Tone 3	In context	Correct	
5	1	Male	/pu/	2	Anger	Tone 3	In context	Correct	
5	2	Female	/ci/	1	Anger	Tone 4	In context	Correct	
5	2	Female	/ci/	2	Anger	Tone 4	In context	Correct	
5	1	Male	/ci/	1	Anger	Tone 4	In context	Correct	
5	1	Male	/ci/	2	Anger	Tone 4	In context	Correct	
5	2	Female	/fa/	1	Anger	Tone 4	In context	Correct	
5	2	Female	/fa/	2	Anger	Tone 4	In context	Correct	
5	1	Male	/fa/	1	Anger	Tone 4	In context	Correct	
5	1	Male	/fa/	2	Anger	Tone 4	In context	Correct	
5	2	Female	/pu/	1	Anger	Tone 4	In context	Correct	
5	2	Female	/pu/	2	Anger	Tone 4	In context	Correct	
5	1	Male	/pu/	1	Anger	Tone 4	In context	Correct	
5	1	Male	/pu/	2	Anger	Tone 4	In context	Correct	
5	2	Female	/ci/	1	Fear	Tone 1	In context	Correct	
5	2	Female	/ci/	2	Fear	Tone 1	In context	Correct	
5	1	Male	/ci/	1	Fear	Tone 1	In context	Correct	
5	1	Male	/ci/	2	Fear	Tone 1	In context	Correct	
5	2	Female	/fa/	1	Fear	Tone 1	In context	Correct	
5	2	Female	/fa/	2	Fear	Tone 1	In context	Correct	
5	1	Male	/fa/	1	Fear	Tone 1	In context	Correct	
5	1	Male	/fa/	2	Fear	Tone 1	In context	Correct	
5	2	Female	/pu/	1	Fear	Tone 1	In context	Correct	
5	2	Female	/pu/	2	Fear	Tone 1	In context	Correct	
5	1	Male	/pu/	1	Fear	Tone 1	In context	Correct	
5	1	Male	/pu/	2	Fear	Tone 1	In context	Correct	
5	2	Female	/ci/	1	Fear	Tone 2	In context	Correct	
5	2	Female	/ci/	2	Fear	Tone 2	In context	Correct	
5	1	Male	/ci/	1	Fear	Tone 2	In context	Correct	
5	1	Male	/ci/	2	Fear	Tone 2	In context	Incorrect	
5	2	Female	/fa/	1	Fear	Tone 2	In context	Correct	
5	2	Female	/fa/	2	Fear	Tone 2	In context	Correct	
5	1	Male	/fa/	1	Fear	Tone 2	In context	Correct	
5	1	Male	/fa/	2	Fear	Tone 2	In context	Correct	
5	2	Female	/pu/	1	Fear	Tone 2	In context	Correct	
5	2	Female	/pu/	2	Fear	Tone 2	In context	Correct	
5	1	Male	/pu/	1	Fear	Tone 2	In context	Correct	
5	1	Male	/pu/	2	Fear	Tone 2	In context	Correct	
5	2	Female	/ci/	1	Fear	Tone 3	In context	Correct	
5	2	Female	/ci/	2	Fear	Tone 3	In context	Correct	
5	1	Male	/ci/	1	Fear	Tone 3	In context	Correct	
5	1	Male	/ci/	2	Fear	Tone 3	In context	Correct	
5	2	Female	/fa/	1	Fear	Tone 3	In context	Correct	
5	2	Female	/fa/	2	Fear	Tone 3	In context	Correct	
5	1	Male	/fa/	1	Fear	Tone 3	In context	Correct	
5	1	Male	/fa/	2	Fear	Tone 3	In context	Correct	
5	2	Female	/pu/	1	Fear	Tone 3	In context	Correct	
5	2	Female	/pu/	2	Fear	Tone 3	In context	Correct	

5	1	Male
5	1	Male
5	2	Female
5	2	Female
5	1	Male
5	1	Male
5	2	Female
5	2	Female
5	1	Male
5	1	Male
5	2	Female
5	2	Female
5	1	Male
5	1	Male
/pu/
/pu/
/ci/
/ci/
/ci/
/ci/
/fa/
/fa/
/fa/
/fa/
/pu/
/pu/
/pu/
/pu/
Fear
Fear
Fear
Fear
Fear
Fear
Fear
Fear
Fear
Fear
Fear
Fear
Fear
Fear
Tone 3 In context Tone 3 In context Tone 4 In context Tone 4 In context Tone 4 In context Tone 4 In context Tone 4 In context Tone 4 In context Tone 4 In context Tone 4 In context Tone 4 In context Tone 4 In context Tone 4 In context Tone 4 In context
Correct Correct Correct Correct Correct Correct Correct Correct Correct Correct Correct Correct Correct Correct

5	2	Female
5	2	Female
5	1	Male
5	1	Male
5	2	Female
5	2	Female
5	1	Male
5	1	Male
5	2	Female
5	2	Female
5	1	Male
/ci/
/ci/
/ci/
/ci/
/fa/
/fa/
/fa/
/fa/
/pu/
/pu/
/pu/
HappinesTone 1 In context
HappinesTone 1 In context
HappinesTone 1 In context
HappinesTone 1 In context
HappinesTone 1 In context
HappinesTone 1 In context
HappinesTone 1 In context
HappinesTone 1 In context
HappinesTone 1 In context
HappinesTone 1 In context
HappinesTone 1 In context
Correct Correct Correct Correct Correct Correct Correct Correct Correct Correct Correct

5	1	Male
/pu/
HappinesTone 1 In context Incorrect

5	2	Female
5	2	Female
5	1	Male
5	1	Male
5	2	Female
5	2	Female
5	1	Male
5	1	Male
5	2	Female
5	2	Female
5	1	Male
5	1	Male
5	2	Female
5	2	Female
5	1	Male
5	1	Male
5	2	Female
5	2	Female
5	1	Male
5	1	Male
5	2	Female
5	2	Female
5	1	Male
5	1	Male
/ci/
/ci/
/ci/
/ci/
/fa/
/fa/
/fa/
/fa/
/pu/
/pu/
/pu/
/pu/
/ci/
/ci/
/ci/
/ci/
/fa/
/fa/
/fa/
/fa/
/pu/
/pu/
/pu/
/pu/
HappinesTone 2 In context
HappinesTone 2 In context
HappinesTone 2 In context
HappinesTone 2 In context
HappinesTone 2 In context
HappinesTone 2 In context
HappinesTone 2 In context
HappinesTone 2 In context
HappinesTone 2 In context
HappinesTone 2 In context
HappinesTone 2 In context
HappinesTone 2 In context
HappinesTone 3 In context
HappinesTone 3 In context
HappinesTone 3 In context
HappinesTone 3 In context
HappinesTone 3 In context
HappinesTone 3 In context
HappinesTone 3 In context
HappinesTone 3 In context
HappinesTone 3 In context
HappinesTone 3 In context
HappinesTone 3 In context
HappinesTone 3 In context
Correct Correct Correct Correct Correct Correct Correct Correct Correct Correct Correct Correct Correct Correct Correct Correct Correct Correct Correct Correct Correct Correct Correct Correct

5	2	Female
/ci/
HappinesTone 4 In context Incorrect

5	2	Female
5	1	Male
5	1	Male
5	2	Female
5	2	Female
5	1	Male
5	1	Male
5	2	Female
5	2	Female
5	1	Male
5	1	Male
/ci/
/ci/
/ci/
/fa/
/fa/
/fa/
/fa/
/pu/
/pu/
/pu/
/pu/
HappinesTone 4 In context
HappinesTone 4 In context
HappinesTone 4 In context
HappinesTone 4 In context
HappinesTone 4 In context
HappinesTone 4 In context
HappinesTone 4 In context
HappinesTone 4 In context
HappinesTone 4 In context
HappinesTone 4 In context
HappinesTone 4 In context
Correct Correct Correct Correct Correct Correct Correct Correct Correct Correct Correct

5	2	Female
5	2	Female
5	1	Male
5	1	Male
5	2	Female
5	2	Female
5	1	Male
5	1	Male
5	2	Female
5	2	Female
5	1	Male
5	1	Male
5	2	Female
5	2	Female
5	1	Male
5	1	Male
5	2	Female
5	2	Female
5	1	Male
5	1	Male
5	2	Female
5	2	Female
5	1	Male
5	1	Male
5	2	Female
5	2	Female
5	1	Male
5	1	Male
5	2	Female
5	2	Female
5	1	Male
5	1	Male
5	2	Female
5	2	Female
5	1	Male
5	1	Male
5	2	Female
5	2	Female
5	1	Male
5	1	Male
/ci/
/ci/
/ci/
/ci/
/fa/
/fa/
/fa/
/fa/
/pu/
/pu/
/pu/
/pu/
/ci/
/ci/
/ci/
/ci/
/fa/
/fa/
/fa/
/fa/
/pu/
/pu/
/pu/
/pu/
/ci/
/ci/
/ci/
/ci/
/fa/
/fa/
/fa/
/fa/
/pu/
/pu/
/pu/
/pu/
/ci/
/ci/
/ci/
/ci/
Neutral Tone 1 In context
Neutral Tone 1 In context
Neutral Tone 1 In context
Neutral Tone 1 In context
Neutral Tone 1 In context
Neutral Tone 1 In context
Neutral Tone 1 In context
Neutral Tone 1 In context
Neutral Tone 1 In context
Neutral Tone 1 In context
Neutral Tone 1 In context
Neutral Tone 1 In context
Neutral Tone 2 In context
Neutral Tone 2 In context
Neutral Tone 2 In context
Neutral Tone 2 In context
Neutral Tone 2 In context
Neutral Tone 2 In context
Neutral Tone 2 In context
Neutral Tone 2 In context
Neutral Tone 2 In context
Neutral Tone 2 In context
Neutral Tone 2 In context
Neutral Tone 2 In context
Neutral Tone 3 In context
Neutral Tone 3 In context
Neutral Tone 3 In context
Neutral Tone 3 In context
Neutral Tone 3 In context
Neutral Tone 3 In context
Neutral Tone 3 In context
Neutral Tone 3 In context
Neutral Tone 3 In context
Neutral Tone 3 In context
Neutral Tone 3 In context
Neutral Tone 3 In context
Neutral Tone 4 In context
Neutral Tone 4 In context
Neutral Tone 4 In context
Neutral Tone 4 In context
Correct Correct Correct Correct Correct Correct Correct Correct Correct Correct Correct Correct Correct Correct Correct Correct Correct Correct Correct Correct Correct Correct Correct Correct Correct Correct Correct Correct Correct Correct Correct Correct Correct Correct Correct Correct Correct Correct Correct Correct

5	2	Female	/fa/	1	Neutral	Tone 4	In context	Correct	
5	2	Female	/fa/	2	Neutral	Tone 4	In context	Correct	
5	1	Male	/fa/	1	Neutral	Tone 4	In context	Correct	
5	1	Male	/fa/	2	Neutral	Tone 4	In context	Correct	
5	2	Female	/pu/	1	Neutral	Tone 4	In context	Correct	
5	2	Female	/pu/	2	Neutral	Tone 4	In context	Correct	
5	1	Male	/pu/	1	Neutral	Tone 4	In context	Correct	
5	1	Male	/pu/	2	Neutral	Tone 4	In context	Correct	
5	2	Female	/ci/	1	Sadness	Tone 1	In context	Correct	
5	2	Female	/ci/	2	Sadness	Tone 1	In context	Correct	
5	1	Male	/ci/	1	Sadness	Tone 1	In context	Correct	
5	1	Male	/ci/	2	Sadness	Tone 1	In context	Correct	
5	2	Female	/fa/	1	Sadness	Tone 1	In context	Correct	
5	2	Female	/fa/	2	Sadness	Tone 1	In context	Correct	
5	1	Male	/fa/	1	Sadness	Tone 1	In context	Correct	
5	1	Male	/fa/	2	Sadness	Tone 1	In context	Correct	
5	2	Female	/pu/	1	Sadness	Tone 1	In context	Correct	
5	2	Female	/pu/	2	Sadness	Tone 1	In context	Correct	
5	1	Male	/pu/	1	Sadness	Tone 1	In context	Correct	
5	1	Male	/pu/	2	Sadness	Tone 1	In context	Correct	
5	2	Female	/ci/	1	Sadness	Tone 2	In context	Correct	
5	2	Female	/ci/	2	Sadness	Tone 2	In context	Correct	
5	1	Male	/ci/	1	Sadness	Tone 2	In context	Correct	
5	1	Male	/ci/	2	Sadness	Tone 2	In context	Correct	
5	2	Female	/fa/	1	Sadness	Tone 2	In context	Correct	
5	2	Female	/fa/	2	Sadness	Tone 2	In context	Correct	
5	1	Male	/fa/	1	Sadness	Tone 2	In context	Correct	
5	1	Male	/fa/	2	Sadness	Tone 2	In context	Correct	
5	2	Female	/pu/	1	Sadness	Tone 2	In context	Correct	
5	2	Female	/pu/	2	Sadness	Tone 2	In context	Correct	
5	1	Male	/pu/	1	Sadness	Tone 2	In context	Correct	
5	1	Male	/pu/	2	Sadness	Tone 2	In context	Correct	
5	2	Female	/ci/	1	Sadness	Tone 3	In context	Correct	
5	2	Female	/ci/	2	Sadness	Tone 3	In context	Correct	
5	1	Male	/ci/	1	Sadness	Tone 3	In context	Correct	
5	1	Male	/ci/	2	Sadness	Tone 3	In context	Correct	
5	2	Female	/fa/	1	Sadness	Tone 3	In context	Correct	
5	2	Female	/fa/	2	Sadness	Tone 3	In context	Correct	
5	1	Male	/fa/	1	Sadness	Tone 3	In context	Correct	
5	1	Male	/fa/	2	Sadness	Tone 3	In context	Correct	
5	2	Female	/pu/	1	Sadness	Tone 3	In context	Correct	
5	2	Female	/pu/	2	Sadness	Tone 3	In context	Correct	
5	1	Male	/pu/	1	Sadness	Tone 3	In context	Correct	
5	1	Male	/pu/	2	Sadness	Tone 3	In context	Correct	
5	2	Female	/ci/	1	Sadness	Tone 4	In context	Correct	
5	2	Female	/ci/	2	Sadness	Tone 4	In context	Correct	
5	1	Male	/ci/	1	Sadness	Tone 4	In context	Correct	
5	1	Male	/ci/	2	Sadness	Tone 4	In context	Correct	
5	2	Female	/fa/	1	Sadness	Tone 4	In context	Correct	
5	2	Female	/fa/	2	Sadness	Tone 4	In context	Correct	
5	1	Male	/fa/	1	Sadness	Tone 4	In context	Correct	

5	1	Male	/fa/	2	Sadness	Tone 4	In context	Correct	
5	2	Female	/pu/	1	Sadness	Tone 4	In context	Correct	
5	2	Female	/pu/	2	Sadness	Tone 4	In context	Correct	
5	1	Male	/pu/	1	Sadness	Tone 4	In context	Correct	
5	1	Male	/pu/	2	Sadness	Tone 4	In context	Correct	
6	2	Female	/ci/	1	Anger	Tone 1	In isolation	Correct	
6	2	Female	/ci/	2	Anger	Tone 1	In isolation	Correct	
6	1	Male	/ci/	1	Anger	Tone 1	In isolation	Correct	
6	1	Male	/ci/	2	Anger	Tone 1	In isolation	Correct	
6	2	Female	/fa/	1	Anger	Tone 1	In isolation	Correct	
6	2	Female	/fa/	2	Anger	Tone 1	In isolation	Correct	
6	1	Male	/fa/	1	Anger	Tone 1	In isolation	Correct	
6	1	Male	/fa/	2	Anger	Tone 1	In isolation	Correct	
6	2	Female	/pu/	1	Anger	Tone 1	In isolation	Incorrect	
6	2	Female	/pu/	2	Anger	Tone 1	In isolation	Incorrect	
6	1	Male	/pu/	1	Anger	Tone 1	In isolation	Correct	
6	1	Male	/pu/	2	Anger	Tone 1	In isolation	Incorrect	
6	2	Female	/ci/	1	Anger	Tone 2	In isolation	Incorrect	
6	2	Female	/ci/	2	Anger	Tone 2	In isolation	Incorrect	
6	1	Male	/ci/	1	Anger	Tone 2	In isolation	Correct	
6	1	Male	/ci/	2	Anger	Tone 2	In isolation	Correct	
6	2	Female	/fa/	1	Anger	Tone 2	In isolation	Correct	
6	2	Female	/fa/	2	Anger	Tone 2	In isolation	Incorrect	
6	1	Male	/fa/	1	Anger	Tone 2	In isolation	Correct	
6	1	Male	/fa/	2	Anger	Tone 2	In isolation	Correct	
6	2	Female	/pu/	1	Anger	Tone 2	In isolation	Correct	
6	2	Female	/pu/	2	Anger	Tone 2	In isolation	Correct	
6	1	Male	/pu/	1	Anger	Tone 2	In isolation	Correct	
6	1	Male	/pu/	2	Anger	Tone 2	In isolation	Correct	
6	2	Female	/ci/	1	Anger	Tone 3	In isolation	Incorrect	
6	2	Female	/ci/	2	Anger	Tone 3	In isolation	Incorrect	
6	1	Male	/ci/	1	Anger	Tone 3	In isolation	Incorrect	
6	1	Male	/ci/	2	Anger	Tone 3	In isolation	Incorrect	
6	2	Female	/fa/	1	Anger	Tone 3	In isolation	Incorrect	
6	2	Female	/fa/	2	Anger	Tone 3	In isolation	Correct	
6	1	Male	/fa/	1	Anger	Tone 3	In isolation	Correct	
6	1	Male	/fa/	2	Anger	Tone 3	In isolation	Incorrect	
6	2	Female	/pu/	1	Anger	Tone 3	In isolation	Incorrect	
6	2	Female	/pu/	2	Anger	Tone 3	In isolation	Incorrect	
6	1	Male	/pu/	1	Anger	Tone 3	In isolation	Incorrect	
6	1	Male	/pu/	2	Anger	Tone 3	In isolation	Incorrect	
6	2	Female	/ci/	1	Anger	Tone 4	In isolation	Correct	
6	2	Female	/ci/	2	Anger	Tone 4	In isolation	Correct	
6	1	Male	/ci/	1	Anger	Tone 4	In isolation	Correct	
6	1	Male	/ci/	2	Anger	Tone 4	In isolation	Correct	
6	2	Female	/fa/	1	Anger	Tone 4	In isolation	Correct	
6	2	Female	/fa/	2	Anger	Tone 4	In isolation	Correct	
6	1	Male	/fa/	1	Anger	Tone 4	In isolation	Correct	
6	1	Male	/fa/	2	Anger	Tone 4	In isolation	Correct	
6	2	Female	/pu/	1	Anger	Tone 4	In isolation	Incorrect	
6	2	Female	/pu/	2	Anger	Tone 4	In isolation	Correct	

6	1	Male	/pu/	1	Anger	Tone 4	In isolation	Incorrect	
6	1	Male	/pu/	2	Anger	Tone 4	In isolation	Correct	
6	2	Female	/ci/	1	Fear	Tone 1	In isolation	Correct	
6	2	Female	/ci/	2	Fear	Tone 1	In isolation	Correct	
6	1	Male	/ci/	1	Fear	Tone 1	In isolation	Correct	
6	1	Male	/ci/	2	Fear	Tone 1	In isolation	Correct	
6	2	Female	/fa/	1	Fear	Tone 1	In isolation	Correct	
6	2	Female	/fa/	2	Fear	Tone 1	In isolation	Correct	
6	1	Male	/fa/	1	Fear	Tone 1	In isolation	Correct	
6	1	Male	/fa/	2	Fear	Tone 1	In isolation	Correct	
6	2	Female	/pu/	1	Fear	Tone 1	In isolation	Correct	
6	2	Female	/pu/	2	Fear	Tone 1	In isolation	Correct	
6	1	Male	/pu/	1	Fear	Tone 1	In isolation	Correct	
6	1	Male	/pu/	2	Fear	Tone 1	In isolation	Correct	
6	2	Female	/ci/	1	Fear	Tone 2	In isolation	Incorrect	
6	2	Female	/ci/	2	Fear	Tone 2	In isolation	Incorrect	
6	1	Male	/ci/	1	Fear	Tone 2	In isolation	Correct	
6	1	Male	/ci/	2	Fear	Tone 2	In isolation	Correct	
6	2	Female	/fa/	1	Fear	Tone 2	In isolation	Incorrect	
6	2	Female	/fa/	2	Fear	Tone 2	In isolation	Correct	
6	1	Male	/fa/	1	Fear	Tone 2	In isolation	Correct	
6	1	Male	/fa/	2	Fear	Tone 2	In isolation	Incorrect	
6	2	Female	/pu/	1	Fear	Tone 2	In isolation	Incorrect	
6	2	Female	/pu/	2	Fear	Tone 2	In isolation	Incorrect	
6	1	Male	/pu/	1	Fear	Tone 2	In isolation	Correct	
6	1	Male	/pu/	2	Fear	Tone 2	In isolation	Incorrect	
6	2	Female	/ci/	1	Fear	Tone 3	In isolation	Incorrect	
6	2	Female	/ci/	2	Fear	Tone 3	In isolation	Incorrect	
6	1	Male	/ci/	1	Fear	Tone 3	In isolation	Incorrect	
6	1	Male	/ci/	2	Fear	Tone 3	In isolation	Incorrect	
6	2	Female	/fa/	1	Fear	Tone 3	In isolation	Incorrect	
6	2	Female	/fa/	2	Fear	Tone 3	In isolation	Incorrect	
6	1	Male	/fa/	1	Fear	Tone 3	In isolation	Correct	
6	1	Male	/fa/	2	Fear	Tone 3	In isolation	Correct	
6	2	Female	/pu/	1	Fear	Tone 3	In isolation	Correct	
6	2	Female	/pu/	2	Fear	Tone 3	In isolation	Incorrect	
6	1	Male	/pu/	1	Fear	Tone 3	In isolation	Incorrect	
6	1	Male	/pu/	2	Fear	Tone 3	In isolation	Incorrect	
6	2	Female	/ci/	1	Fear	Tone 4	In isolation	Correct	
6	2	Female	/ci/	2	Fear	Tone 4	In isolation	Correct	
6	1	Male	/ci/	1	Fear	Tone 4	In isolation	Correct	
6	1	Male	/ci/	2	Fear	Tone 4	In isolation	Correct	
6	2	Female	/fa/	1	Fear	Tone 4	In isolation	Correct	
6	2	Female	/fa/	2	Fear	Tone 4	In isolation	Correct	
6	1	Male	/fa/	1	Fear	Tone 4	In isolation	Correct	
6	1	Male	/fa/	2	Fear	Tone 4	In isolation	Correct	
6	2	Female	/pu/	1	Fear	Tone 4	In isolation	Correct	
6	2	Female	/pu/	2	Fear	Tone 4	In isolation	Correct	
6	1	Male	/pu/	1	Fear	Tone 4	In isolation	Correct	
6	1	Male	/pu/	2	Fear	Tone 4	In isolation	Incorrect	
6	2	Female	/ci/	1	Happines	Tone 1	In isolation	Correct	

6	2	Female
6	1	Male
6	1	Male
6	2	Female
6	2	Female
6	1	Male
6	1	Male
6	2	Female
6	2	Female
6	1	Male
6	1	Male
6	2	Female
6	2	Female
6	1	Male
6	1	Male
6	2	Female
6	2	Female
6	1	Male
6	1	Male
6	2	Female
6	2	Female
6	1	Male
6	1	Male
6	2	Female
6	2	Female
6	1	Male
6	1	Male
6	2	Female
6	2	Female
6	1	Male
6	1	Male
6	2	Female
6	2	Female
6	1	Male
6	1	Male
6	2	Female
6	2	Female
6	1	Male
6	1	Male
6	2	Female
6	2	Female
6	1	Male
6	1	Male
6	2	Female
6	2	Female
6	1	Male
6	1	Male
/ci/
/ci/
/ci/
/fa/
/fa/
/fa/
/fa/
/pu/
/pu/
/pu/
/pu/
/ci/
/ci/
/ci/
/ci/
/fa/
/fa/
/fa/
/fa/
/pu/
/pu/
/pu/
/pu/
/ci/
/ci/
/ci/
/ci/
/fa/
/fa/
/fa/
/fa/
/pu/
/pu/
/pu/
/pu/
/ci/
/ci/
/ci/
/ci/
/fa/
/fa/
/fa/
/fa/
/pu/
/pu/
/pu/
/pu/
2	HappinesTone 1In isolation Incorrect
HappinesTone 1In isolation Correct
HappinesTone 1In isolation Correct
HappinesTone 1In isolation Incorrect
HappinesTone 1In isolation Incorrect
HappinesTone 1In isolation Correct
HappinesTone 1In isolation Correct
HappinesTone 1In isolation Incorrect
HappinesTone 1In isolation Incorrect
HappinesTone 1In isolation Correct
HappinesTone 1In isolation Correct
HappinesTone 2In isolation Correct
HappinesTone 2In isolation Correct
HappinesTone 2In isolation Correct
HappinesTone 2In isolation Correct
HappinesTone 2In isolation Correct
HappinesTone 2In isolation Correct
HappinesTone 2In isolation Incorrect
HappinesTone 2In isolation Incorrect
HappinesTone 2In isolation Correct
HappinesTone 2In isolation Correct
HappinesTone 2In isolation Correct
HappinesTone 2In isolation Correct
HappinesTone 3In isolation Incorrect
HappinesTone 3In isolation Incorrect
HappinesTone 3In isolation Incorrect
HappinesTone 3In isolation Incorrect
HappinesTone 3In isolation Correct
HappinesTone 3In isolation Incorrect
HappinesTone 3In isolation Correct
HappinesTone 3In isolation Correct
HappinesTone 3In isolation Incorrect
HappinesTone 3In isolation Incorrect
HappinesTone 3In isolation Correct
HappinesTone 3In isolation Correct
HappinesTone 4In isolation Incorrect
HappinesTone 4In isolation Incorrect
HappinesTone 4In isolation Correct
HappinesTone 4In isolation Correct
HappinesTone 4In isolation Incorrect
HappinesTone 4In isolation Correct
HappinesTone 4In isolation Correct
HappinesTone 4In isolation Correct
HappinesTone 4In isolation Incorrect
HappinesTone 4In isolation Incorrect
HappinesTone 4In isolation Correct
HappinesTone 4In isolation Correct

6	2	Female
6	2	Female
6	1	Male
6	1	Male
/ci/
/ci/
/ci/
/ci/
Neutral Tone 1In isolation Correct
Neutral Tone 1In isolation Correct
Neutral Tone 1In isolation Correct
Neutral Tone 1In isolation Correct

6	2	Female	/fa/	1	Neutral	Tone 1	In isolation	Correct	
6	2	Female	/fa/	2	Neutral	Tone 1	In isolation	Correct	
6	1	Male	/fa/	1	Neutral	Tone 1	In isolation	Correct	
6	1	Male	/fa/	2	Neutral	Tone 1	In isolation	Correct	
6	2	Female	/pu/	1	Neutral	Tone 1	In isolation	Correct	
6	2	Female	/pu/	2	Neutral	Tone 1	In isolation	Correct	
6	1	Male	/pu/	1	Neutral	Tone 1	In isolation	Correct	
6	1	Male	/pu/	2	Neutral	Tone 1	In isolation	Correct	
6	2	Female	/ci/	1	Neutral	Tone 2	In isolation	Correct	
6	2	Female	/ci/	2	Neutral	Tone 2	In isolation	Correct	
6	1	Male	/ci/	1	Neutral	Tone 2	In isolation	Correct	
6	1	Male	/ci/	2	Neutral	Tone 2	In isolation	Correct	
6	2	Female	/fa/	1	Neutral	Tone 2	In isolation	Correct	
6	2	Female	/fa/	2	Neutral	Tone 2	In isolation	Correct	
6	1	Male	/fa/	1	Neutral	Tone 2	In isolation	Correct	
6	1	Male	/fa/	2	Neutral	Tone 2	In isolation	Correct	
6	2	Female	/pu/	1	Neutral	Tone 2	In isolation	Correct	
6	2	Female	/pu/	2	Neutral	Tone 2	In isolation	Correct	
6	1	Male	/pu/	1	Neutral	Tone 2	In isolation	Correct	
6	1	Male	/pu/	2	Neutral	Tone 2	In isolation	Correct	
6	2	Female	/ci/	1	Neutral	Tone 3	In isolation	Correct	
6	2	Female	/ci/	2	Neutral	Tone 3	In isolation	Correct	
6	1	Male	/ci/	1	Neutral	Tone 3	In isolation	Correct	
6	1	Male	/ci/	2	Neutral	Tone 3	In isolation	Correct	
6	2	Female	/fa/	1	Neutral	Tone 3	In isolation	Correct	
6	2	Female	/fa/	2	Neutral	Tone 3	In isolation	Correct	
6	1	Male	/fa/	1	Neutral	Tone 3	In isolation	Correct	
6	1	Male	/fa/	2	Neutral	Tone 3	In isolation	Correct	
6	2	Female	/pu/	1	Neutral	Tone 3	In isolation	Correct	
6	2	Female	/pu/	2	Neutral	Tone 3	In isolation	Correct	
6	1	Male	/pu/	1	Neutral	Tone 3	In isolation	Correct	
6	1	Male	/pu/	2	Neutral	Tone 3	In isolation	Correct	
6	2	Female	/ci/	1	Neutral	Tone 4	In isolation	Correct	
6	2	Female	/ci/	2	Neutral	Tone 4	In isolation	Correct	
6	1	Male	/ci/	1	Neutral	Tone 4	In isolation	Correct	
6	1	Male	/ci/	2	Neutral	Tone 4	In isolation	Correct	
6	2	Female	/fa/	1	Neutral	Tone 4	In isolation	Correct	
6	2	Female	/fa/	2	Neutral	Tone 4	In isolation	Correct	
6	1	Male	/fa/	1	Neutral	Tone 4	In isolation	Correct	
6	1	Male	/fa/	2	Neutral	Tone 4	In isolation	Correct	
6	2	Female	/pu/	1	Neutral	Tone 4	In isolation	Correct	
6	2	Female	/pu/	2	Neutral	Tone 4	In isolation	Correct	
6	1	Male	/pu/	1	Neutral	Tone 4	In isolation	Correct	
6	1	Male	/pu/	2	Neutral	Tone 4	In isolation	Correct	
6	2	Female	/ci/	1	Sadness	Tone 1	In isolation	Correct	
6	2	Female	/ci/	2	Sadness	Tone 1	In isolation	Correct	
6	1	Male	/ci/	1	Sadness	Tone 1	In isolation	Correct	
6	1	Male	/ci/	2	Sadness	Tone 1	In isolation	Correct	
6	2	Female	/fa/	1	Sadness	Tone 1	In isolation	Correct	
6	2	Female	/fa/	2	Sadness	Tone 1	In isolation	Correct	
6	1	Male	/fa/	1	Sadness	Tone 1	In isolation	Correct	

6	1	Male	/fa/	2	Sadness	Tone 1	In isolation	Correct	
6	2	Female	/pu/	1	Sadness	Tone 1	In isolation	Correct	
6	2	Female	/pu/	2	Sadness	Tone 1	In isolation	Correct	
6	1	Male	/pu/	1	Sadness	Tone 1	In isolation	Correct	
6	1	Male	/pu/	2	Sadness	Tone 1	In isolation	Correct	
6	2	Female	/ci/	1	Sadness	Tone 2	In isolation	Incorrect	
6	2	Female	/ci/	2	Sadness	Tone 2	In isolation	Incorrect	
6	1	Male	/ci/	1	Sadness	Tone 2	In isolation	Correct	
6	1	Male	/ci/	2	Sadness	Tone 2	In isolation	Correct	
6	2	Female	/fa/	1	Sadness	Tone 2	In isolation	Correct	
6	2	Female	/fa/	2	Sadness	Tone 2	In isolation	Correct	
6	1	Male	/fa/	1	Sadness	Tone 2	In isolation	Incorrect	
6	1	Male	/fa/	2	Sadness	Tone 2	In isolation	Incorrect	
6	2	Female	/pu/	1	Sadness	Tone 2	In isolation	Correct	
6	2	Female	/pu/	2	Sadness	Tone 2	In isolation	Incorrect	
6	1	Male	/pu/	1	Sadness	Tone 2	In isolation	Incorrect	
6	1	Male	/pu/	2	Sadness	Tone 2	In isolation	Correct	
6	2	Female	/ci/	1	Sadness	Tone 3	In isolation	Incorrect	
6	2	Female	/ci/	2	Sadness	Tone 3	In isolation	Incorrect	
6	1	Male	/ci/	1	Sadness	Tone 3	In isolation	Incorrect	
6	1	Male	/ci/	2	Sadness	Tone 3	In isolation	Incorrect	
6	2	Female	/fa/	1	Sadness	Tone 3	In isolation	Incorrect	
6	2	Female	/fa/	2	Sadness	Tone 3	In isolation	Incorrect	
6	1	Male	/fa/	1	Sadness	Tone 3	In isolation	Correct	
6	1	Male	/fa/	2	Sadness	Tone 3	In isolation	Incorrect	
6	2	Female	/pu/	1	Sadness	Tone 3	In isolation	Incorrect	
6	2	Female	/pu/	2	Sadness	Tone 3	In isolation	Incorrect	
6	1	Male	/pu/	1	Sadness	Tone 3	In isolation	Incorrect	
6	1	Male	/pu/	2	Sadness	Tone 3	In isolation	Incorrect	
6	2	Female	/ci/	1	Sadness	Tone 4	In isolation	Correct	
6	2	Female	/ci/	2	Sadness	Tone 4	In isolation	Correct	
6	1	Male	/ci/	1	Sadness	Tone 4	In isolation	Correct	
6	1	Male	/ci/	2	Sadness	Tone 4	In isolation	Correct	
6	2	Female	/fa/	1	Sadness	Tone 4	In isolation	Incorrect	
6	2	Female	/fa/	2	Sadness	Tone 4	In isolation	Incorrect	
6	1	Male	/fa/	1	Sadness	Tone 4	In isolation	Incorrect	
6	1	Male	/fa/	2	Sadness	Tone 4	In isolation	Correct	
6	2	Female	/pu/	1	Sadness	Tone 4	In isolation	Correct	
6	2	Female	/pu/	2	Sadness	Tone 4	In isolation	Correct	
6	1	Male	/pu/	1	Sadness	Tone 4	In isolation	Incorrect	
6	1	Male	/pu/	2	Sadness	Tone 4	In isolation	Incorrect	
6	2	Female	/ci/	1	Anger	Tone 1	In context	Incorrect	
6	2	Female	/ci/	2	Anger	Tone 1	In context	Incorrect	
6	1	Male	/ci/	1	Anger	Tone 1	In context	Correct	
6	1	Male	/ci/	2	Anger	Tone 1	In context	Correct	
6	2	Female	/fa/	1	Anger	Tone 1	In context	Correct	
6	2	Female	/fa/	2	Anger	Tone 1	In context	Correct	
6	1	Male	/fa/	1	Anger	Tone 1	In context	Correct	
6	1	Male	/fa/	2	Anger	Tone 1	In context	Correct	
6	2	Female	/pu/	1	Anger	Tone 1	In context	Incorrect	
6	2	Female	/pu/	2	Anger	Tone 1	In context	Incorrect	

6	1	Male	/pu/	1	Anger	Tone 1	In context	Correct	
6	1	Male	/pu/	2	Anger	Tone 1	In context	Incorrect	
6	2	Female	/ci/	1	Anger	Tone 2	In context	Correct	
6	2	Female	/ci/	2	Anger	Tone 2	In context	Correct	
6	1	Male	/ci/	1	Anger	Tone 2	In context	Correct	
6	1	Male	/ci/	2	Anger	Tone 2	In context	Correct	
6	2	Female	/fa/	1	Anger	Tone 2	In context	Correct	
6	2	Female	/fa/	2	Anger	Tone 2	In context	Correct	
6	1	Male	/fa/	1	Anger	Tone 2	In context	Correct	
6	1	Male	/fa/	2	Anger	Tone 2	In context	Correct	
6	2	Female	/pu/	1	Anger	Tone 2	In context	Correct	
6	2	Female	/pu/	2	Anger	Tone 2	In context	Correct	
6	1	Male	/pu/	1	Anger	Tone 2	In context	Correct	
6	1	Male	/pu/	2	Anger	Tone 2	In context	Correct	
6	2	Female	/ci/	1	Anger	Tone 3	In context	Correct	
6	2	Female	/ci/	2	Anger	Tone 3	In context	Correct	
6	1	Male	/ci/	1	Anger	Tone 3	In context	Correct	
6	1	Male	/ci/	2	Anger	Tone 3	In context	Correct	
6	2	Female	/fa/	1	Anger	Tone 3	In context	Correct	
6	2	Female	/fa/	2	Anger	Tone 3	In context	Correct	
6	1	Male	/fa/	1	Anger	Tone 3	In context	Correct	
6	1	Male	/fa/	2	Anger	Tone 3	In context	Correct	
6	2	Female	/pu/	1	Anger	Tone 3	In context	Correct	
6	2	Female	/pu/	2	Anger	Tone 3	In context	Correct	
6	1	Male	/pu/	1	Anger	Tone 3	In context	Correct	
6	1	Male	/pu/	2	Anger	Tone 3	In context	Correct	
6	2	Female	/ci/	1	Anger	Tone 4	In context	Correct	
6	2	Female	/ci/	2	Anger	Tone 4	In context	Correct	
6	1	Male	/ci/	1	Anger	Tone 4	In context	Correct	
6	1	Male	/ci/	2	Anger	Tone 4	In context	Correct	
6	2	Female	/fa/	1	Anger	Tone 4	In context	Correct	
6	2	Female	/fa/	2	Anger	Tone 4	In context	Correct	
6	1	Male	/fa/	1	Anger	Tone 4	In context	Correct	
6	1	Male	/fa/	2	Anger	Tone 4	In context	Correct	
6	2	Female	/pu/	1	Anger	Tone 4	In context	Correct	
6	2	Female	/pu/	2	Anger	Tone 4	In context	Correct	
6	1	Male	/pu/	1	Anger	Tone 4	In context	Correct	
6	1	Male	/pu/	2	Anger	Tone 4	In context	Correct	
6	2	Female	/ci/	1	Fear	Tone 1	In context	Correct	
6	2	Female	/ci/	2	Fear	Tone 1	In context	Correct	
6	1	Male	/ci/	1	Fear	Tone 1	In context	Correct	
6	1	Male	/ci/	2	Fear	Tone 1	In context	Correct	
6	2	Female	/fa/	1	Fear	Tone 1	In context	Correct	
6	2	Female	/fa/	2	Fear	Tone 1	In context	Correct	
6	1	Male	/fa/	1	Fear	Tone 1	In context	Incorrect	
6	1	Male	/fa/	2	Fear	Tone 1	In context	Correct	
6	2	Female	/pu/	1	Fear	Tone 1	In context	Correct	
6	2	Female	/pu/	2	Fear	Tone 1	In context	Correct	
6	1	Male	/pu/	1	Fear	Tone 1	In context	Correct	
6	1	Male	/pu/	2	Fear	Tone 1	In context	Correct	
6	2	Female	/ci/	1	Fear	Tone 2	In context	Correct	

6	2	Female
6	1	Male
6	1	Male
6	2	Female
6	2	Female
6	1	Male
6	1	Male
6	2	Female
6	2	Female
6	1	Male
6	1	Male
6	2	Female
6	2	Female
6	1	Male
6	1	Male
6	2	Female
6	2	Female
6	1	Male
6	1	Male
/ci/
/ci/
/ci/
/fa/
/fa/
/fa/
/fa/
/pu/
/pu/
/pu/
/pu/
/ci/
/ci/
/ci/
/ci/
/fa/
/fa/
/fa/
/fa/
2	Fear
Fear
Fear
Fear
Fear
Fear
Fear
Fear
Fear
Fear
Fear
Fear
Fear
Fear
Fear
Fear
Fear
Fear
Fear
Tone 2 In context Tone 2 In context Tone 2 In context Tone 2 In context Tone 2 In context Tone 2 In context Tone 2 In context Tone 2 In context Tone 2 In context Tone 2 In context Tone 2 In context Tone 3 In context Tone 3 In context Tone 3 In context Tone 3 In context Tone 3 In context Tone 3 In context Tone 3 In context Tone 3 In context
Correct Correct Correct Correct Correct Correct Correct Correct Correct Correct Correct Correct Correct Correct Correct Correct Correct Correct Correct

6	2	Female
/pu/
Fear
Tone 3 In context Incorrect

6	2	Female
6	1	Male
6	1	Male
6	2	Female
6	2	Female
6	1	Male
6	1	Male
6	2	Female
6	2	Female
6	1	Male
6	1	Male
/pu/
/pu/
/pu/
/ci/
/ci/
/ci/
/ci/
/fa/
/fa/
/fa/
/fa/
Fear
Fear
Fear
Fear
Fear
Fear
Fear
Fear
Fear
Fear
Fear
Tone 3 In context Tone 3 In context Tone 3 In context Tone 4 In context Tone 4 In context Tone 4 In context Tone 4 In context Tone 4 In context Tone 4 In context Tone 4 In context Tone 4 In context
Correct Correct Correct Correct Correct Correct Correct Correct Correct Correct Correct

6	2	Female
/pu/
Fear
Tone 4 In context Incorrect

6	2	Female
/pu/
Fear
Tone 4 In context
Correct

6	1	Male
/pu/
Fear
Tone 4 In context Incorrect

6	1	Male
/pu/
Fear
Tone 4 In context
Correct

6	2	Female
6	2	Female
6	1	Male
6	1	Male
6	2	Female
6	2	Female
6	1	Male
6	1	Male
6	2	Female
/ci/
/ci/
/ci/
/ci/
/fa/
/fa/
/fa/
/fa/
/pu/
HappinesTone 1 In context
HappinesTone 1 In context
HappinesTone 1 In context
HappinesTone 1 In context
HappinesTone 1 In context
HappinesTone 1 In context
HappinesTone 1 In context
HappinesTone 1 In context
HappinesTone 1 In context
Correct Correct Correct Correct Correct Correct Correct Correct Correct

6	2	Female
/pu/
HappinesTone 1 In context Incorrect

6	1	Male
/pu/
HappinesTone 1 In context
Correct

6	1	Male
/pu/
HappinesTone 1 In context Incorrect

6	2	Female
6	2	Female
6	1	Male
6	1	Male
/ci/
/ci/
/ci/
/ci/
HappinesTone 2 In context
HappinesTone 2 In context
HappinesTone 2 In context
HappinesTone 2 In context
Correct Correct Correct Correct

6	2	Female
6	2	Female
6	1	Male
6	1	Male
6	2	Female
6	2	Female
6	1	Male
6	1	Male
6	2	Female
6	2	Female
6	1	Male
6	1	Male
6	2	Female
6	2	Female
6	1	Male
6	1	Male
6	2	Female
6	2	Female
6	1	Male
6	1	Male
/fa/
/fa/
/fa/
/fa/
/pu/
/pu/
/pu/
/pu/
/ci/
/ci/
/ci/
/ci/
/fa/
/fa/
/fa/
/fa/
/pu/
/pu/
/pu/
/pu/
HappinesTone 2 In context
HappinesTone 2 In context
HappinesTone 2 In context
HappinesTone 2 In context
HappinesTone 2 In context
HappinesTone 2 In context
HappinesTone 2 In context
HappinesTone 2 In context
HappinesTone 3 In context
HappinesTone 3 In context
HappinesTone 3 In context
HappinesTone 3 In context
HappinesTone 3 In context
HappinesTone 3 In context
HappinesTone 3 In context
HappinesTone 3 In context
HappinesTone 3 In context
HappinesTone 3 In context
HappinesTone 3 In context
HappinesTone 3 In context
Correct Correct Correct Correct Correct Correct Correct Correct Correct Correct Correct Correct Correct Correct Correct Correct Correct Correct Correct Correct

6	2	Female
6	2	Female
/ci/
/ci/
HappinesTone 4 In context Incorrect
HappinesTone 4 In context Incorrect

6	1	Male
6	1	Male
6	2	Female
6	2	Female
6	1	Male
6	1	Male
/ci/
/ci/
/fa/
/fa/
/fa/
/fa/
HappinesTone 4 In context
HappinesTone 4 In context
HappinesTone 4 In context
HappinesTone 4 In context
HappinesTone 4 In context
HappinesTone 4 In context
Correct Correct Correct Correct Correct Correct

6	2	Female
/pu/
HappinesTone 4 In context Incorrect

6	2	Female
6	1	Male
6	1	Male
/pu/
/pu/
/pu/
HappinesTone 4 In context
HappinesTone 4 In context
HappinesTone 4 In context
Correct Correct Correct

6	2	Female
6	2	Female
6	1	Male
6	1	Male
6	2	Female
6	2	Female
6	1	Male
6	1	Male
6	2	Female
6	2	Female
6	1	Male
6	1	Male
6	2	Female
6	2	Female
6	1	Male
6	1	Male
6	2	Female
6	2	Female
6	1	Male
/ci/
/ci/
/ci/
/ci/
/fa/
/fa/
/fa/
/fa/
/pu/
/pu/
/pu/
/pu/
/ci/
/ci/
/ci/
/ci/
/fa/
/fa/
/fa/
Neutral Tone 1 In context
Neutral Tone 1 In context
Neutral Tone 1 In context
Neutral Tone 1 In context
Neutral Tone 1 In context
Neutral Tone 1 In context
Neutral Tone 1 In context
Neutral Tone 1 In context
Neutral Tone 1 In context
Neutral Tone 1 In context
Neutral Tone 1 In context
Neutral Tone 1 In context
Neutral Tone 2 In context
Neutral Tone 2 In context
Neutral Tone 2 In context
Neutral Tone 2 In context
Neutral Tone 2 In context
Neutral Tone 2 In context
1	Neutral Tone 2 In context
Correct Correct Correct Correct Correct Correct Correct Correct Correct Correct Correct Correct Correct Correct Correct Correct Correct Correct Correct

6	1	Male	/fa/	2	Neutral	Tone 2	In context	Correct	
6	2	Female	/pu/	1	Neutral	Tone 2	In context	Correct	
6	2	Female	/pu/	2	Neutral	Tone 2	In context	Correct	
6	1	Male	/pu/	1	Neutral	Tone 2	In context	Correct	
6	1	Male	/pu/	2	Neutral	Tone 2	In context	Correct	
6	2	Female	/ci/	1	Neutral	Tone 3	In context	Correct	
6	2	Female	/ci/	2	Neutral	Tone 3	In context	Correct	
6	1	Male	/ci/	1	Neutral	Tone 3	In context	Correct	
6	1	Male	/ci/	2	Neutral	Tone 3	In context	Correct	
6	2	Female	/fa/	1	Neutral	Tone 3	In context	Correct	
6	2	Female	/fa/	2	Neutral	Tone 3	In context	Correct	
6	1	Male	/fa/	1	Neutral	Tone 3	In context	Correct	
6	1	Male	/fa/	2	Neutral	Tone 3	In context	Correct	
6	2	Female	/pu/	1	Neutral	Tone 3	In context	Correct	
6	2	Female	/pu/	2	Neutral	Tone 3	In context	Correct	
6	1	Male	/pu/	1	Neutral	Tone 3	In context	Correct	
6	1	Male	/pu/	2	Neutral	Tone 3	In context	Correct	
6	2	Female	/ci/	1	Neutral	Tone 4	In context	Correct	
6	2	Female	/ci/	2	Neutral	Tone 4	In context	Correct	
6	1	Male	/ci/	1	Neutral	Tone 4	In context	Correct	
6	1	Male	/ci/	2	Neutral	Tone 4	In context	Correct	
6	2	Female	/fa/	1	Neutral	Tone 4	In context	Correct	
6	2	Female	/fa/	2	Neutral	Tone 4	In context	Correct	
6	1	Male	/fa/	1	Neutral	Tone 4	In context	Correct	
6	1	Male	/fa/	2	Neutral	Tone 4	In context	Correct	
6	2	Female	/pu/	1	Neutral	Tone 4	In context	Correct	
6	2	Female	/pu/	2	Neutral	Tone 4	In context	Correct	
6	1	Male	/pu/	1	Neutral	Tone 4	In context	Correct	
6	1	Male	/pu/	2	Neutral	Tone 4	In context	Correct	
6	2	Female	/ci/	1	Sadness	Tone 1	In context	Correct	
6	2	Female	/ci/	2	Sadness	Tone 1	In context	Correct	
6	1	Male	/ci/	1	Sadness	Tone 1	In context	Correct	
6	1	Male	/ci/	2	Sadness	Tone 1	In context	Correct	
6	2	Female	/fa/	1	Sadness	Tone 1	In context	Correct	
6	2	Female	/fa/	2	Sadness	Tone 1	In context	Correct	
6	1	Male	/fa/	1	Sadness	Tone 1	In context	Correct	
6	1	Male	/fa/	2	Sadness	Tone 1	In context	Incorrect	
6	2	Female	/pu/	1	Sadness	Tone 1	In context	Correct	
6	2	Female	/pu/	2	Sadness	Tone 1	In context	Correct	
6	1	Male	/pu/	1	Sadness	Tone 1	In context	Correct	
6	1	Male	/pu/	2	Sadness	Tone 1	In context	Correct	
6	2	Female	/ci/	1	Sadness	Tone 2	In context	Correct	
6	2	Female	/ci/	2	Sadness	Tone 2	In context	Correct	
6	1	Male	/ci/	1	Sadness	Tone 2	In context	Correct	
6	1	Male	/ci/	2	Sadness	Tone 2	In context	Correct	
6	2	Female	/fa/	1	Sadness	Tone 2	In context	Correct	
6	2	Female	/fa/	2	Sadness	Tone 2	In context	Correct	
6	1	Male	/fa/	1	Sadness	Tone 2	In context	Correct	
6	1	Male	/fa/	2	Sadness	Tone 2	In context	Correct	
6	2	Female	/pu/	1	Sadness	Tone 2	In context	Correct	
6	2	Female	/pu/	2	Sadness	Tone 2	In context	Correct	

6	1	Male	/pu/	1	Sadness	Tone 2	In context	Correct	
6	1	Male	/pu/	2	Sadness	Tone 2	In context	Correct	
6	2	Female	/ci/	1	Sadness	Tone 3	In context	Correct	
6	2	Female	/ci/	2	Sadness	Tone 3	In context	Correct	
6	1	Male	/ci/	1	Sadness	Tone 3	In context	Correct	
6	1	Male	/ci/	2	Sadness	Tone 3	In context	Correct	
6	2	Female	/fa/	1	Sadness	Tone 3	In context	Correct	
6	2	Female	/fa/	2	Sadness	Tone 3	In context	Correct	
6	1	Male	/fa/	1	Sadness	Tone 3	In context	Correct	
6	1	Male	/fa/	2	Sadness	Tone 3	In context	Correct	
6	2	Female	/pu/	1	Sadness	Tone 3	In context	Correct	
6	2	Female	/pu/	2	Sadness	Tone 3	In context	Correct	
6	1	Male	/pu/	1	Sadness	Tone 3	In context	Correct	
6	1	Male	/pu/	2	Sadness	Tone 3	In context	Correct	
6	2	Female	/ci/	1	Sadness	Tone 4	In context	Correct	
6	2	Female	/ci/	2	Sadness	Tone 4	In context	Correct	
6	1	Male	/ci/	1	Sadness	Tone 4	In context	Correct	
6	1	Male	/ci/	2	Sadness	Tone 4	In context	Correct	
6	2	Female	/fa/	1	Sadness	Tone 4	In context	Correct	
6	2	Female	/fa/	2	Sadness	Tone 4	In context	Correct	
6	1	Male	/fa/	1	Sadness	Tone 4	In context	Correct	
6	1	Male	/fa/	2	Sadness	Tone 4	In context	Correct	
6	2	Female	/pu/	1	Sadness	Tone 4	In context	Correct	
6	2	Female	/pu/	2	Sadness	Tone 4	In context	Correct	
6	1	Male	/pu/	1	Sadness	Tone 4	In context	Correct	
6	1	Male	/pu/	2	Sadness	Tone 4	In context	Correct	
7	2	Female	/ci/	1	Anger	Tone 1	In isolation	Correct	
7	2	Female	/ci/	2	Anger	Tone 1	In isolation	Correct	
7	1	Male	/ci/	1	Anger	Tone 1	In isolation	Correct	
7	1	Male	/ci/	2	Anger	Tone 1	In isolation	Correct	
7	2	Female	/fa/	1	Anger	Tone 1	In isolation	Correct	
7	2	Female	/fa/	2	Anger	Tone 1	In isolation	Correct	
7	1	Male	/fa/	1	Anger	Tone 1	In isolation	Correct	
7	1	Male	/fa/	2	Anger	Tone 1	In isolation	Correct	
7	2	Female	/pu/	1	Anger	Tone 1	In isolation	Incorrect	
7	2	Female	/pu/	2	Anger	Tone 1	In isolation	Correct	
7	1	Male	/pu/	1	Anger	Tone 1	In isolation	Incorrect	
7	1	Male	/pu/	2	Anger	Tone 1	In isolation	Correct	
7	2	Female	/ci/	1	Anger	Tone 2	In isolation	Incorrect	
7	2	Female	/ci/	2	Anger	Tone 2	In isolation	Incorrect	
7	1	Male	/ci/	1	Anger	Tone 2	In isolation	Incorrect	
7	1	Male	/ci/	2	Anger	Tone 2	In isolation	Correct	
7	2	Female	/fa/	1	Anger	Tone 2	In isolation	Correct	
7	2	Female	/fa/	2	Anger	Tone 2	In isolation	Incorrect	
7	1	Male	/fa/	1	Anger	Tone 2	In isolation	Correct	
7	1	Male	/fa/	2	Anger	Tone 2	In isolation	Correct	
7	2	Female	/pu/	1	Anger	Tone 2	In isolation	Correct	
7	2	Female	/pu/	2	Anger	Tone 2	In isolation	Correct	
7	1	Male	/pu/	1	Anger	Tone 2	In isolation	Correct	
7	1	Male	/pu/	2	Anger	Tone 2	In isolation	Correct	
7	2	Female	/ci/	1	Anger	Tone 3	In isolation	Incorrect	

7	2	Female	/ci/	2	Anger	Tone 3	In isolation	Incorrect	
7	1	Male	/ci/	1	Anger	Tone 3	In isolation	Correct	
7	1	Male	/ci/	2	Anger	Tone 3	In isolation	Incorrect	
7	2	Female	/fa/	1	Anger	Tone 3	In isolation	Incorrect	
7	2	Female	/fa/	2	Anger	Tone 3	In isolation	Correct	
7	1	Male	/fa/	1	Anger	Tone 3	In isolation	Correct	
7	1	Male	/fa/	2	Anger	Tone 3	In isolation	Correct	
7	2	Female	/pu/	1	Anger	Tone 3	In isolation	Incorrect	
7	2	Female	/pu/	2	Anger	Tone 3	In isolation	Incorrect	
7	1	Male	/pu/	1	Anger	Tone 3	In isolation	Incorrect	
7	1	Male	/pu/	2	Anger	Tone 3	In isolation	Correct	
7	2	Female	/ci/	1	Anger	Tone 4	In isolation	Correct	
7	2	Female	/ci/	2	Anger	Tone 4	In isolation	Correct	
7	1	Male	/ci/	1	Anger	Tone 4	In isolation	Correct	
7	1	Male	/ci/	2	Anger	Tone 4	In isolation	Correct	
7	2	Female	/fa/	1	Anger	Tone 4	In isolation	Correct	
7	2	Female	/fa/	2	Anger	Tone 4	In isolation	Correct	
7	1	Male	/fa/	1	Anger	Tone 4	In isolation	Correct	
7	1	Male	/fa/	2	Anger	Tone 4	In isolation	Incorrect	
7	2	Female	/pu/	1	Anger	Tone 4	In isolation	Correct	
7	2	Female	/pu/	2	Anger	Tone 4	In isolation	Correct	
7	1	Male	/pu/	1	Anger	Tone 4	In isolation	Correct	
7	1	Male	/pu/	2	Anger	Tone 4	In isolation	Correct	
7	2	Female	/ci/	1	Fear	Tone 1	In isolation	Correct	
7	2	Female	/ci/	2	Fear	Tone 1	In isolation	Correct	
7	1	Male	/ci/	1	Fear	Tone 1	In isolation	Correct	
7	1	Male	/ci/	2	Fear	Tone 1	In isolation	Correct	
7	2	Female	/fa/	1	Fear	Tone 1	In isolation	Correct	
7	2	Female	/fa/	2	Fear	Tone 1	In isolation	Correct	
7	1	Male	/fa/	1	Fear	Tone 1	In isolation	Correct	
7	1	Male	/fa/	2	Fear	Tone 1	In isolation	Correct	
7	2	Female	/pu/	1	Fear	Tone 1	In isolation	Incorrect	
7	2	Female	/pu/	2	Fear	Tone 1	In isolation	Correct	
7	1	Male	/pu/	1	Fear	Tone 1	In isolation	Incorrect	
7	1	Male	/pu/	2	Fear	Tone 1	In isolation	Correct	
7	2	Female	/ci/	1	Fear	Tone 2	In isolation	Incorrect	
7	2	Female	/ci/	2	Fear	Tone 2	In isolation	Incorrect	
7	1	Male	/ci/	1	Fear	Tone 2	In isolation	Correct	
7	1	Male	/ci/	2	Fear	Tone 2	In isolation	Incorrect	
7	2	Female	/fa/	1	Fear	Tone 2	In isolation	Incorrect	
7	2	Female	/fa/	2	Fear	Tone 2	In isolation	Correct	
7	1	Male	/fa/	1	Fear	Tone 2	In isolation	Correct	
7	1	Male	/fa/	2	Fear	Tone 2	In isolation	Correct	
7	2	Female	/pu/	1	Fear	Tone 2	In isolation	Correct	
7	2	Female	/pu/	2	Fear	Tone 2	In isolation	Incorrect	
7	1	Male	/pu/	1	Fear	Tone 2	In isolation	Correct	
7	1	Male	/pu/	2	Fear	Tone 2	In isolation	Correct	
7	2	Female	/ci/	1	Fear	Tone 3	In isolation	Correct	
7	2	Female	/ci/	2	Fear	Tone 3	In isolation	Incorrect	
7	1	Male	/ci/	1	Fear	Tone 3	In isolation	Correct	
7	1	Male	/ci/	2	Fear	Tone 3	In isolation	Correct	

7	2	Female
7	2	Female
7	1	Male
7	1	Male
7	2	Female
7	2	Female
7	1	Male
7	1	Male
7	2	Female
7	2	Female
7	1	Male
7	1	Male
7	2	Female
7	2	Female
7	1	Male
7	1	Male
7	2	Female
7	2	Female
7	1	Male
7	1	Male
/fa/
/fa/
/fa/
/fa/
/pu/
/pu/
/pu/
/pu/
/ci/
/ci/
/ci/
/ci/
/fa/
/fa/
/fa/
/fa/
/pu/
/pu/
/pu/
/pu/
Fear
Fear
Fear
Fear
Fear
Fear
Fear
Fear
Fear
Fear
Fear
Fear
Fear
Fear
Fear
Fear
Fear
Fear
Fear
Fear
Tone 3In isolation Incorrect Tone 3In isolation Incorrect Tone 3In isolation Correct Tone 3In isolation Incorrect Tone 3In isolation Correct Tone 3In isolation Correct Tone 3In isolation Incorrect Tone 3In isolation Correct Tone 4In isolation Correct Tone 4In isolation Correct Tone 4In isolation Correct Tone 4In isolation Incorrect Tone 4In isolation Correct Tone 4In isolation Correct Tone 4In isolation Incorrect Tone 4In isolation Incorrect Tone 4In isolation Correct Tone 4In isolation Correct Tone 4In isolation Incorrect Tone 4In isolation Correct

7	2	Female
7	2	Female
7	1	Male
7	1	Male
7	2	Female
7	2	Female
7	1	Male
7	1	Male
7	2	Female
7	2	Female
7	1	Male
7	1	Male
7	2	Female
7	2	Female
7	1	Male
7	1	Male
7	2	Female
7	2	Female
7	1	Male
7	1	Male
7	2	Female
7	2	Female
7	1	Male
7	1	Male
7	2	Female
7	2	Female
7	1	Male
7	1	Male
7	2	Female
7	2	Female
7	1	Male
/ci/
/ci/
/ci/
/ci/
/fa/
/fa/
/fa/
/fa/
/pu/
/pu/
/pu/
/pu/
/ci/
/ci/
/ci/
/ci/
/fa/
/fa/
/fa/
/fa/
/pu/
/pu/
/pu/
/pu/
/ci/
/ci/
/ci/
/ci/
/fa/
/fa/
/fa/
HappinesTone 1In isolation Incorrect
HappinesTone 1In isolation Correct
HappinesTone 1In isolation Incorrect
HappinesTone 1In isolation Incorrect
HappinesTone 1In isolation Incorrect
HappinesTone 1In isolation Incorrect
HappinesTone 1In isolation Correct
HappinesTone 1In isolation Correct
HappinesTone 1In isolation Incorrect
HappinesTone 1In isolation Correct
HappinesTone 1In isolation Correct
HappinesTone 1In isolation Correct
HappinesTone 2In isolation Incorrect
HappinesTone 2In isolation Correct
HappinesTone 2In isolation Correct
HappinesTone 2In isolation Correct
HappinesTone 2In isolation Correct
HappinesTone 2In isolation Correct
HappinesTone 2In isolation Correct
HappinesTone 2In isolation Correct
HappinesTone 2In isolation Correct
HappinesTone 2In isolation Correct
HappinesTone 2In isolation Correct
HappinesTone 2In isolation Correct
HappinesTone 3In isolation Correct
HappinesTone 3In isolation Correct
HappinesTone 3In isolation Correct
HappinesTone 3In isolation Correct
HappinesTone 3In isolation Correct
HappinesTone 3In isolation Correct
HappinesTone 3In isolation Correct

7	1	Male
7	2	Female
7	2	Female
7	1	Male
7	1	Male
7	2	Female
7	2	Female
7	1	Male
7	1	Male
7	2	Female
7	2	Female
7	1	Male
7	1	Male
7	2	Female
7	2	Female
7	1	Male
7	1	Male
/fa/
/pu/
/pu/
/pu/
/pu/
/ci/
/ci/
/ci/
/ci/
/fa/
/fa/
/fa/
/fa/
/pu/
/pu/
/pu/
/pu/
HappinesTone 3In isolation Correct
HappinesTone 3In isolation Incorrect
HappinesTone 3In isolation Incorrect
HappinesTone 3In isolation Correct
HappinesTone 3In isolation Correct
HappinesTone 4In isolation Incorrect
HappinesTone 4In isolation Incorrect
HappinesTone 4In isolation Incorrect
HappinesTone 4In isolation Correct
HappinesTone 4In isolation Incorrect
HappinesTone 4In isolation Correct
HappinesTone 4In isolation Correct
HappinesTone 4In isolation Correct
HappinesTone 4In isolation Incorrect
HappinesTone 4In isolation Incorrect
HappinesTone 4In isolation Correct
HappinesTone 4In isolation Correct

7	2	Female
7	2	Female
7	1	Male
7	1	Male
7	2	Female
7	2	Female
7	1	Male
7	1	Male
7	2	Female
7	2	Female
7	1	Male
7	1	Male
7	2	Female
7	2	Female
7	1	Male
7	1	Male
7	2	Female
7	2	Female
7	1	Male
7	1	Male
7	2	Female
7	2	Female
7	1	Male
7	1	Male
7	2	Female
7	2	Female
7	1	Male
7	1	Male
7	2	Female
7	2	Female
7	1	Male
7	1	Male
7	2	Female
7	2	Female
/ci/
/ci/
/ci/
/ci/
/fa/
/fa/
/fa/
/fa/
/pu/
/pu/
/pu/
/pu/
/ci/
/ci/
/ci/
/ci/
/fa/
/fa/
/fa/
/fa/
/pu/
/pu/
/pu/
/pu/
/ci/
/ci/
/ci/
/ci/
/fa/
/fa/
/fa/
/fa/
/pu/
/pu/
Neutral Tone 1In isolation Correct
Neutral Tone 1In isolation Correct
Neutral Tone 1In isolation Correct
Neutral Tone 1In isolation Correct
Neutral Tone 1In isolation Correct
Neutral Tone 1In isolation Correct
Neutral Tone 1In isolation Correct
Neutral Tone 1In isolation Correct
Neutral Tone 1In isolation Correct
Neutral Tone 1In isolation Correct
Neutral Tone 1In isolation Correct
Neutral Tone 1In isolation Correct
Neutral Tone 2In isolation Correct
Neutral Tone 2In isolation Correct
Neutral Tone 2In isolation Correct
Neutral Tone 2In isolation Correct
Neutral Tone 2In isolation Correct
Neutral Tone 2In isolation Correct
Neutral Tone 2In isolation Correct
Neutral Tone 2In isolation Correct
Neutral Tone 2In isolation Correct
Neutral Tone 2In isolation Correct
Neutral Tone 2In isolation Correct
Neutral Tone 2In isolation Correct
Neutral Tone 3In isolation Correct
Neutral Tone 3In isolation Correct
Neutral Tone 3In isolation Correct
Neutral Tone 3In isolation Correct
Neutral Tone 3In isolation Correct
Neutral Tone 3In isolation Correct
Neutral Tone 3In isolation Correct
Neutral Tone 3In isolation Correct
Neutral Tone 3In isolation Correct
Neutral Tone 3In isolation Correct

7	1	Male	/pu/	1	Neutral	Tone 3	In isolation	Correct	
7	1	Male	/pu/	2	Neutral	Tone 3	In isolation	Correct	
7	2	Female	/ci/	1	Neutral	Tone 4	In isolation	Correct	
7	2	Female	/ci/	2	Neutral	Tone 4	In isolation	Correct	
7	1	Male	/ci/	1	Neutral	Tone 4	In isolation	Correct	
7	1	Male	/ci/	2	Neutral	Tone 4	In isolation	Correct	
7	2	Female	/fa/	1	Neutral	Tone 4	In isolation	Correct	
7	2	Female	/fa/	2	Neutral	Tone 4	In isolation	Correct	
7	1	Male	/fa/	1	Neutral	Tone 4	In isolation	Correct	
7	1	Male	/fa/	2	Neutral	Tone 4	In isolation	Correct	
7	2	Female	/pu/	1	Neutral	Tone 4	In isolation	Correct	
7	2	Female	/pu/	2	Neutral	Tone 4	In isolation	Correct	
7	1	Male	/pu/	1	Neutral	Tone 4	In isolation	Correct	
7	1	Male	/pu/	2	Neutral	Tone 4	In isolation	Correct	
7	2	Female	/ci/	1	Sadness	Tone 1	In isolation	Correct	
7	2	Female	/ci/	2	Sadness	Tone 1	In isolation	Correct	
7	1	Male	/ci/	1	Sadness	Tone 1	In isolation	Correct	
7	1	Male	/ci/	2	Sadness	Tone 1	In isolation	Correct	
7	2	Female	/fa/	1	Sadness	Tone 1	In isolation	Correct	
7	2	Female	/fa/	2	Sadness	Tone 1	In isolation	Correct	
7	1	Male	/fa/	1	Sadness	Tone 1	In isolation	Correct	
7	1	Male	/fa/	2	Sadness	Tone 1	In isolation	Correct	
7	2	Female	/pu/	1	Sadness	Tone 1	In isolation	Correct	
7	2	Female	/pu/	2	Sadness	Tone 1	In isolation	Correct	
7	1	Male	/pu/	1	Sadness	Tone 1	In isolation	Correct	
7	1	Male	/pu/	2	Sadness	Tone 1	In isolation	Incorrect	
7	2	Female	/ci/	1	Sadness	Tone 2	In isolation	Correct	
7	2	Female	/ci/	2	Sadness	Tone 2	In isolation	Incorrect	
7	1	Male	/ci/	1	Sadness	Tone 2	In isolation	Incorrect	
7	1	Male	/ci/	2	Sadness	Tone 2	In isolation	Incorrect	
7	2	Female	/fa/	1	Sadness	Tone 2	In isolation	Correct	
7	2	Female	/fa/	2	Sadness	Tone 2	In isolation	Correct	
7	1	Male	/fa/	1	Sadness	Tone 2	In isolation	Correct	
7	1	Male	/fa/	2	Sadness	Tone 2	In isolation	Correct	
7	2	Female	/pu/	1	Sadness	Tone 2	In isolation	Correct	
7	2	Female	/pu/	2	Sadness	Tone 2	In isolation	Correct	
7	1	Male	/pu/	1	Sadness	Tone 2	In isolation	Correct	
7	1	Male	/pu/	2	Sadness	Tone 2	In isolation	Correct	
7	2	Female	/ci/	1	Sadness	Tone 3	In isolation	Correct	
7	2	Female	/ci/	2	Sadness	Tone 3	In isolation	Incorrect	
7	1	Male	/ci/	1	Sadness	Tone 3	In isolation	Correct	
7	1	Male	/ci/	2	Sadness	Tone 3	In isolation	Correct	
7	2	Female	/fa/	1	Sadness	Tone 3	In isolation	Correct	
7	2	Female	/fa/	2	Sadness	Tone 3	In isolation	Incorrect	
7	1	Male	/fa/	1	Sadness	Tone 3	In isolation	Correct	
7	1	Male	/fa/	2	Sadness	Tone 3	In isolation	Correct	
7	2	Female	/pu/	1	Sadness	Tone 3	In isolation	Incorrect	
7	2	Female	/pu/	2	Sadness	Tone 3	In isolation	Correct	
7	1	Male	/pu/	1	Sadness	Tone 3	In isolation	Correct	
7	1	Male	/pu/	2	Sadness	Tone 3	In isolation	Incorrect	
7	2	Female	/ci/	1	Sadness	Tone 4	In isolation	Incorrect	

7	2	Female	/ci/	2	Sadness	Tone 4	In isolation	Incorrect	
7	1	Male	/ci/	1	Sadness	Tone 4	In isolation	Incorrect	
7	1	Male	/ci/	2	Sadness	Tone 4	In isolation	Correct	
7	2	Female	/fa/	1	Sadness	Tone 4	In isolation	Correct	
7	2	Female	/fa/	2	Sadness	Tone 4	In isolation	Incorrect	
7	1	Male	/fa/	1	Sadness	Tone 4	In isolation	Incorrect	
7	1	Male	/fa/	2	Sadness	Tone 4	In isolation	Correct	
7	2	Female	/pu/	1	Sadness	Tone 4	In isolation	Correct	
7	2	Female	/pu/	2	Sadness	Tone 4	In isolation	Correct	
7	1	Male	/pu/	1	Sadness	Tone 4	In isolation	Incorrect	
7	1	Male	/pu/	2	Sadness	Tone 4	In isolation	Incorrect	
7	2	Female	/ci/	1	Anger	Tone 1	In context	Correct	
7	2	Female	/ci/	2	Anger	Tone 1	In context	Correct	
7	1	Male	/ci/	1	Anger	Tone 1	In context	Correct	
7	1	Male	/ci/	2	Anger	Tone 1	In context	Correct	
7	2	Female	/fa/	1	Anger	Tone 1	In context	Correct	
7	2	Female	/fa/	2	Anger	Tone 1	In context	Correct	
7	1	Male	/fa/	1	Anger	Tone 1	In context	Correct	
7	1	Male	/fa/	2	Anger	Tone 1	In context	Correct	
7	2	Female	/pu/	1	Anger	Tone 1	In context	Incorrect	
7	2	Female	/pu/	2	Anger	Tone 1	In context	Incorrect	
7	1	Male	/pu/	1	Anger	Tone 1	In context	Correct	
7	1	Male	/pu/	2	Anger	Tone 1	In context	Correct	
7	2	Female	/ci/	1	Anger	Tone 2	In context	Correct	
7	2	Female	/ci/	2	Anger	Tone 2	In context	Correct	
7	1	Male	/ci/	1	Anger	Tone 2	In context	Correct	
7	1	Male	/ci/	2	Anger	Tone 2	In context	Correct	
7	2	Female	/fa/	1	Anger	Tone 2	In context	Correct	
7	2	Female	/fa/	2	Anger	Tone 2	In context	Correct	
7	1	Male	/fa/	1	Anger	Tone 2	In context	Correct	
7	1	Male	/fa/	2	Anger	Tone 2	In context	Correct	
7	2	Female	/pu/	1	Anger	Tone 2	In context	Correct	
7	2	Female	/pu/	2	Anger	Tone 2	In context	Correct	
7	1	Male	/pu/	1	Anger	Tone 2	In context	Correct	
7	1	Male	/pu/	2	Anger	Tone 2	In context	Correct	
7	2	Female	/ci/	1	Anger	Tone 3	In context	Correct	
7	2	Female	/ci/	2	Anger	Tone 3	In context	Correct	
7	1	Male	/ci/	1	Anger	Tone 3	In context	Correct	
7	1	Male	/ci/	2	Anger	Tone 3	In context	Correct	
7	2	Female	/fa/	1	Anger	Tone 3	In context	Correct	
7	2	Female	/fa/	2	Anger	Tone 3	In context	Correct	
7	1	Male	/fa/	1	Anger	Tone 3	In context	Correct	
7	1	Male	/fa/	2	Anger	Tone 3	In context	Correct	
7	2	Female	/pu/	1	Anger	Tone 3	In context	Correct	
7	2	Female	/pu/	2	Anger	Tone 3	In context	Correct	
7	1	Male	/pu/	1	Anger	Tone 3	In context	Correct	
7	1	Male	/pu/	2	Anger	Tone 3	In context	Correct	
7	2	Female	/ci/	1	Anger	Tone 4	In context	Correct	
7	2	Female	/ci/	2	Anger	Tone 4	In context	Correct	
7	1	Male	/ci/	1	Anger	Tone 4	In context	Correct	
7	1	Male	/ci/	2	Anger	Tone 4	In context	Correct	

7	2	Female	/fa/	1	Anger	Tone 4	In context	Correct	
7	2	Female	/fa/	2	Anger	Tone 4	In context	Correct	
7	1	Male	/fa/	1	Anger	Tone 4	In context	Correct	
7	1	Male	/fa/	2	Anger	Tone 4	In context	Correct	
7	2	Female	/pu/	1	Anger	Tone 4	In context	Correct	
7	2	Female	/pu/	2	Anger	Tone 4	In context	Correct	
7	1	Male	/pu/	1	Anger	Tone 4	In context	Correct	
7	1	Male	/pu/	2	Anger	Tone 4	In context	Correct	
7	2	Female	/ci/	1	Fear	Tone 1	In context	Correct	
7	2	Female	/ci/	2	Fear	Tone 1	In context	Correct	
7	1	Male	/ci/	1	Fear	Tone 1	In context	Correct	
7	1	Male	/ci/	2	Fear	Tone 1	In context	Correct	
7	2	Female	/fa/	1	Fear	Tone 1	In context	Correct	
7	2	Female	/fa/	2	Fear	Tone 1	In context	Correct	
7	1	Male	/fa/	1	Fear	Tone 1	In context	Correct	
7	1	Male	/fa/	2	Fear	Tone 1	In context	Correct	
7	2	Female	/pu/	1	Fear	Tone 1	In context	Correct	
7	2	Female	/pu/	2	Fear	Tone 1	In context	Correct	
7	1	Male	/pu/	1	Fear	Tone 1	In context	Correct	
7	1	Male	/pu/	2	Fear	Tone 1	In context	Correct	
7	2	Female	/ci/	1	Fear	Tone 2	In context	Correct	
7	2	Female	/ci/	2	Fear	Tone 2	In context	Correct	
7	1	Male	/ci/	1	Fear	Tone 2	In context	Correct	
7	1	Male	/ci/	2	Fear	Tone 2	In context	Correct	
7	2	Female	/fa/	1	Fear	Tone 2	In context	Correct	
7	2	Female	/fa/	2	Fear	Tone 2	In context	Correct	
7	1	Male	/fa/	1	Fear	Tone 2	In context	Correct	
7	1	Male	/fa/	2	Fear	Tone 2	In context	Correct	
7	2	Female	/pu/	1	Fear	Tone 2	In context	Correct	
7	2	Female	/pu/	2	Fear	Tone 2	In context	Correct	
7	1	Male	/pu/	1	Fear	Tone 2	In context	Correct	
7	1	Male	/pu/	2	Fear	Tone 2	In context	Correct	
7	2	Female	/ci/	1	Fear	Tone 3	In context	Correct	
7	2	Female	/ci/	2	Fear	Tone 3	In context	Correct	
7	1	Male	/ci/	1	Fear	Tone 3	In context	Correct	
7	1	Male	/ci/	2	Fear	Tone 3	In context	Correct	
7	2	Female	/fa/	1	Fear	Tone 3	In context	Correct	
7	2	Female	/fa/	2	Fear	Tone 3	In context	Correct	
7	1	Male	/fa/	1	Fear	Tone 3	In context	Correct	
7	1	Male	/fa/	2	Fear	Tone 3	In context	Correct	
7	2	Female	/pu/	1	Fear	Tone 3	In context	Correct	
7	2	Female	/pu/	2	Fear	Tone 3	In context	Correct	
7	1	Male	/pu/	1	Fear	Tone 3	In context	Correct	
7	1	Male	/pu/	2	Fear	Tone 3	In context	Correct	
7	2	Female	/ci/	1	Fear	Tone 4	In context	Correct	
7	2	Female	/ci/	2	Fear	Tone 4	In context	Correct	
7	1	Male	/ci/	1	Fear	Tone 4	In context	Correct	
7	1	Male	/ci/	2	Fear	Tone 4	In context	Correct	
7	2	Female	/fa/	1	Fear	Tone 4	In context	Correct	
7	2	Female	/fa/	2	Fear	Tone 4	In context	Correct	
7	1	Male	/fa/	1	Fear	Tone 4	In context	Correct	

7	1	Male
7	2	Female
7	2	Female
7	1	Male
7	1	Male
/fa/
/pu/
/pu/
/pu/
/pu/
2	Fear
Fear
Fear
Fear
Fear
Tone 4 In context Tone 4 In context Tone 4 In context Tone 4 In context Tone 4 In context
Correct Correct Correct Correct Correct

7	2	Female
7	2	Female
7	1	Male
7	1	Male
7	2	Female
7	2	Female
7	1	Male
7	1	Male
7	2	Female
7	2	Female
7	1	Male
/ci/
/ci/
/ci/
/ci/
/fa/
/fa/
/fa/
/fa/
/pu/
/pu/
/pu/
HappinesTone 1 In context
HappinesTone 1 In context
HappinesTone 1 In context
HappinesTone 1 In context
HappinesTone 1 In context
HappinesTone 1 In context
HappinesTone 1 In context
HappinesTone 1 In context
HappinesTone 1 In context
HappinesTone 1 In context
HappinesTone 1 In context
Correct Correct Correct Correct Correct Correct Correct Correct Correct Correct Correct

7	1	Male
/pu/
HappinesTone 1 In context Incorrect

7	2	Female
7	2	Female
7	1	Male
7	1	Male
7	2	Female
7	2	Female
7	1	Male
7	1	Male
7	2	Female
7	2	Female
7	1	Male
7	1	Male
7	2	Female
7	2	Female
7	1	Male
7	1	Male
7	2	Female
7	2	Female
7	1	Male
7	1	Male
7	2	Female
7	2	Female
7	1	Male
7	1	Male
7	2	Female
7	2	Female
7	1	Male
7	1	Male
7	2	Female
7	2	Female
7	1	Male
7	1	Male
7	2	Female
7	2	Female
/ci/
/ci/
/ci/
/ci/
/fa/
/fa/
/fa/
/fa/
/pu/
/pu/
/pu/
/pu/
/ci/
/ci/
/ci/
/ci/
/fa/
/fa/
/fa/
/fa/
/pu/
/pu/
/pu/
/pu/
/ci/
/ci/
/ci/
/ci/
/fa/
/fa/
/fa/
/fa/
/pu/
/pu/
HappinesTone 2 In context
HappinesTone 2 In context
HappinesTone 2 In context
HappinesTone 2 In context
HappinesTone 2 In context
HappinesTone 2 In context
HappinesTone 2 In context
HappinesTone 2 In context
HappinesTone 2 In context
HappinesTone 2 In context
HappinesTone 2 In context
HappinesTone 2 In context
HappinesTone 3 In context
HappinesTone 3 In context
HappinesTone 3 In context
HappinesTone 3 In context
HappinesTone 3 In context
HappinesTone 3 In context
HappinesTone 3 In context
HappinesTone 3 In context
HappinesTone 3 In context
HappinesTone 3 In context
HappinesTone 3 In context
HappinesTone 3 In context
HappinesTone 4 In context
HappinesTone 4 In context
HappinesTone 4 In context
HappinesTone 4 In context
HappinesTone 4 In context
HappinesTone 4 In context
HappinesTone 4 In context
HappinesTone 4 In context
HappinesTone 4 In context
HappinesTone 4 In context
Correct Correct Correct Correct Correct Correct Correct Correct Correct Correct Correct Correct Correct Correct Correct Correct Correct Correct Correct Correct Correct Correct Correct Correct Correct Correct Correct Correct Correct Correct Correct Correct Correct Correct

7	1	Male	/pu/	1	Happines	Tone 4	In context	Correct	
7	1	Male	/pu/	2	Happines	Tone 4	In context	Correct	
7	2	Female	/ci/	1	Neutral	Tone 1	In context	Correct	
7	2	Female	/ci/	2	Neutral	Tone 1	In context	Correct	
7	1	Male	/ci/	1	Neutral	Tone 1	In context	Correct	
7	1	Male	/ci/	2	Neutral	Tone 1	In context	Correct	
7	2	Female	/fa/	1	Neutral	Tone 1	In context	Correct	
7	2	Female	/fa/	2	Neutral	Tone 1	In context	Correct	
7	1	Male	/fa/	1	Neutral	Tone 1	In context	Correct	
7	1	Male	/fa/	2	Neutral	Tone 1	In context	Correct	
7	2	Female	/pu/	1	Neutral	Tone 1	In context	Correct	
7	2	Female	/pu/	2	Neutral	Tone 1	In context	Correct	
7	1	Male	/pu/	1	Neutral	Tone 1	In context	Correct	
7	2	Female	/ci/	1	Neutral	Tone 2	In context	Correct	
7	2	Female	/ci/	2	Neutral	Tone 2	In context	Correct	
7	1	Male	/ci/	1	Neutral	Tone 2	In context	Correct	
7	1	Male	/ci/	2	Neutral	Tone 2	In context	Correct	
7	2	Female	/fa/	1	Neutral	Tone 2	In context	Correct	
7	2	Female	/fa/	2	Neutral	Tone 2	In context	Correct	
7	1	Male	/fa/	1	Neutral	Tone 2	In context	Correct	
7	1	Male	/fa/	2	Neutral	Tone 2	In context	Correct	
7	2	Female	/pu/	1	Neutral	Tone 2	In context	Correct	
7	2	Female	/pu/	2	Neutral	Tone 2	In context	Correct	
7	1	Male	/pu/	1	Neutral	Tone 2	In context	Correct	
7	1	Male	/pu/	2	Neutral	Tone 2	In context	Correct	
7	2	Female	/ci/	1	Neutral	Tone 3	In context	Correct	
7	2	Female	/ci/	2	Neutral	Tone 3	In context	Correct	
7	1	Male	/ci/	1	Neutral	Tone 3	In context	Correct	
7	1	Male	/ci/	2	Neutral	Tone 3	In context	Correct	
7	2	Female	/fa/	1	Neutral	Tone 3	In context	Correct	
7	2	Female	/fa/	2	Neutral	Tone 3	In context	Correct	
7	1	Male	/fa/	1	Neutral	Tone 3	In context	Correct	
7	1	Male	/fa/	2	Neutral	Tone 3	In context	Correct	
7	2	Female	/pu/	1	Neutral	Tone 3	In context	Correct	
7	2	Female	/pu/	2	Neutral	Tone 3	In context	Correct	
7	1	Male	/pu/	1	Neutral	Tone 3	In context	Correct	
7	1	Male	/pu/	2	Neutral	Tone 3	In context	Correct	
7	2	Female	/ci/	1	Neutral	Tone 4	In context	Correct	
7	2	Female	/ci/	2	Neutral	Tone 4	In context	Correct	
7	1	Male	/ci/	1	Neutral	Tone 4	In context	Correct	
7	1	Male	/ci/	2	Neutral	Tone 4	In context	Correct	
7	2	Female	/fa/	1	Neutral	Tone 4	In context	Correct	
7	2	Female	/fa/	2	Neutral	Tone 4	In context	Correct	
7	1	Male	/fa/	1	Neutral	Tone 4	In context	Correct	
7	1	Male	/fa/	2	Neutral	Tone 4	In context	Correct	
7	2	Female	/pu/	1	Neutral	Tone 4	In context	Correct	
7	2	Female	/pu/	2	Neutral	Tone 4	In context	Correct	
7	1	Male	/pu/	1	Neutral	Tone 4	In context	Correct	
7	1	Male	/pu/	2	Neutral	Tone 4	In context	Correct	
7	2	Female	/ci/	1	Sadness	Tone 1	In context	Correct	
7	2	Female	/ci/	2	Sadness	Tone 1	In context	Correct	

7	1	Male	/ci/	1	Sadness	Tone 1	In context	Correct	
7	1	Male	/ci/	2	Sadness	Tone 1	In context	Correct	
7	2	Female	/fa/	1	Sadness	Tone 1	In context	Correct	
7	2	Female	/fa/	2	Sadness	Tone 1	In context	Correct	
7	1	Male	/fa/	1	Sadness	Tone 1	In context	Correct	
7	1	Male	/fa/	2	Sadness	Tone 1	In context	Correct	
7	2	Female	/pu/	1	Sadness	Tone 1	In context	Correct	
7	2	Female	/pu/	2	Sadness	Tone 1	In context	Correct	
7	1	Male	/pu/	1	Sadness	Tone 1	In context	Correct	
7	1	Male	/pu/	2	Sadness	Tone 1	In context	Correct	
7	2	Female	/ci/	1	Sadness	Tone 2	In context	Correct	
7	2	Female	/ci/	2	Sadness	Tone 2	In context	Correct	
7	1	Male	/ci/	1	Sadness	Tone 2	In context	Correct	
7	1	Male	/ci/	2	Sadness	Tone 2	In context	Correct	
7	2	Female	/fa/	1	Sadness	Tone 2	In context	Correct	
7	2	Female	/fa/	2	Sadness	Tone 2	In context	Correct	
7	1	Male	/fa/	1	Sadness	Tone 2	In context	Correct	
7	1	Male	/fa/	2	Sadness	Tone 2	In context	Correct	
7	2	Female	/pu/	1	Sadness	Tone 2	In context	Correct	
7	2	Female	/pu/	2	Sadness	Tone 2	In context	Correct	
7	1	Male	/pu/	1	Sadness	Tone 2	In context	Correct	
7	1	Male	/pu/	2	Sadness	Tone 2	In context	Correct	
7	2	Female	/ci/	1	Sadness	Tone 3	In context	Correct	
7	2	Female	/ci/	2	Sadness	Tone 3	In context	Correct	
7	1	Male	/ci/	1	Sadness	Tone 3	In context	Correct	
7	1	Male	/ci/	2	Sadness	Tone 3	In context	Correct	
7	2	Female	/fa/	1	Sadness	Tone 3	In context	Correct	
7	2	Female	/fa/	2	Sadness	Tone 3	In context	Correct	
7	1	Male	/fa/	1	Sadness	Tone 3	In context	Correct	
7	1	Male	/fa/	2	Sadness	Tone 3	In context	Incorrect	
7	2	Female	/pu/	1	Sadness	Tone 3	In context	Correct	
7	2	Female	/pu/	2	Sadness	Tone 3	In context	Correct	
7	1	Male	/pu/	1	Sadness	Tone 3	In context	Correct	
7	1	Male	/pu/	2	Sadness	Tone 3	In context	Correct	
7	2	Female	/ci/	1	Sadness	Tone 4	In context	Correct	
7	2	Female	/ci/	2	Sadness	Tone 4	In context	Correct	
7	1	Male	/ci/	1	Sadness	Tone 4	In context	Correct	
7	1	Male	/ci/	2	Sadness	Tone 4	In context	Correct	
7	2	Female	/fa/	1	Sadness	Tone 4	In context	Correct	
7	2	Female	/fa/	2	Sadness	Tone 4	In context	Correct	
7	1	Male	/fa/	1	Sadness	Tone 4	In context	Correct	
7	1	Male	/fa/	2	Sadness	Tone 4	In context	Correct	
7	2	Female	/pu/	1	Sadness	Tone 4	In context	Correct	
7	2	Female	/pu/	2	Sadness	Tone 4	In context	Correct	
7	1	Male	/pu/	1	Sadness	Tone 4	In context	Correct	
7	1	Male	/pu/	2	Sadness	Tone 4	In context	Correct	
8	2	Female	/ci/	1	Anger	Tone 1	In isolation	Correct	
8	2	Female	/ci/	2	Anger	Tone 1	In isolation	Correct	
8	1	Male	/ci/	1	Anger	Tone 1	In isolation	Correct	
8	1	Male	/ci/	2	Anger	Tone 1	In isolation	Correct	
8	2	Female	/fa/	1	Anger	Tone 1	In isolation	Correct	

8	2	Female	/fa/	2	Anger	Tone 1	In isolation	Incorrect	
8	1	Male	/fa/	1	Anger	Tone 1	In isolation	Incorrect	
8	1	Male	/fa/	2	Anger	Tone 1	In isolation	Incorrect	
8	2	Female	/pu/	1	Anger	Tone 1	In isolation	Correct	
8	2	Female	/pu/	2	Anger	Tone 1	In isolation	Correct	
8	1	Male	/pu/	1	Anger	Tone 1	In isolation	Correct	
8	1	Male	/pu/	2	Anger	Tone 1	In isolation	Correct	
8	2	Female	/ci/	1	Anger	Tone 2	In isolation	Incorrect	
8	2	Female	/ci/	2	Anger	Tone 2	In isolation	Incorrect	
8	1	Male	/ci/	1	Anger	Tone 2	In isolation	Correct	
8	1	Male	/ci/	2	Anger	Tone 2	In isolation	Correct	
8	2	Female	/fa/	1	Anger	Tone 2	In isolation	Correct	
8	2	Female	/fa/	2	Anger	Tone 2	In isolation	Correct	
8	1	Male	/fa/	1	Anger	Tone 2	In isolation	Correct	
8	1	Male	/fa/	2	Anger	Tone 2	In isolation	Correct	
8	2	Female	/pu/	1	Anger	Tone 2	In isolation	Correct	
8	2	Female	/pu/	2	Anger	Tone 2	In isolation	Correct	
8	1	Male	/pu/	1	Anger	Tone 2	In isolation	Correct	
8	1	Male	/pu/	2	Anger	Tone 2	In isolation	Incorrect	
8	2	Female	/ci/	1	Anger	Tone 3	In isolation	Incorrect	
8	2	Female	/ci/	2	Anger	Tone 3	In isolation	Incorrect	
8	1	Male	/ci/	1	Anger	Tone 3	In isolation	Incorrect	
8	1	Male	/ci/	2	Anger	Tone 3	In isolation	Incorrect	
8	2	Female	/fa/	1	Anger	Tone 3	In isolation	Incorrect	
8	2	Female	/fa/	2	Anger	Tone 3	In isolation	Correct	
8	1	Male	/fa/	1	Anger	Tone 3	In isolation	Correct	
8	1	Male	/fa/	2	Anger	Tone 3	In isolation	Correct	
8	2	Female	/pu/	1	Anger	Tone 3	In isolation	Incorrect	
8	2	Female	/pu/	2	Anger	Tone 3	In isolation	Incorrect	
8	1	Male	/pu/	1	Anger	Tone 3	In isolation	Incorrect	
8	1	Male	/pu/	2	Anger	Tone 3	In isolation	Incorrect	
8	2	Female	/ci/	1	Anger	Tone 4	In isolation	Correct	
8	2	Female	/ci/	2	Anger	Tone 4	In isolation	Correct	
8	1	Male	/ci/	1	Anger	Tone 4	In isolation	Incorrect	
8	1	Male	/ci/	2	Anger	Tone 4	In isolation	Correct	
8	2	Female	/fa/	1	Anger	Tone 4	In isolation	Correct	
8	2	Female	/fa/	2	Anger	Tone 4	In isolation	Correct	
8	1	Male	/fa/	1	Anger	Tone 4	In isolation	Correct	
8	1	Male	/fa/	2	Anger	Tone 4	In isolation	Correct	
8	2	Female	/pu/	1	Anger	Tone 4	In isolation	Correct	
8	2	Female	/pu/	2	Anger	Tone 4	In isolation	Correct	
8	1	Male	/pu/	1	Anger	Tone 4	In isolation	Correct	
8	1	Male	/pu/	2	Anger	Tone 4	In isolation	Correct	
8	2	Female	/ci/	1	Fear	Tone 1	In isolation	Correct	
8	2	Female	/ci/	2	Fear	Tone 1	In isolation	Correct	
8	1	Male	/ci/	1	Fear	Tone 1	In isolation	Correct	
8	1	Male	/ci/	2	Fear	Tone 1	In isolation	Correct	
8	2	Female	/fa/	1	Fear	Tone 1	In isolation	Incorrect	
8	2	Female	/fa/	2	Fear	Tone 1	In isolation	Correct	
8	1	Male	/fa/	1	Fear	Tone 1	In isolation	Incorrect	
8	1	Male	/fa/	2	Fear	Tone 1	In isolation	Correct	

8	2	Female
8	2	Female
8	1	Male
8	1	Male
8	2	Female
8	2	Female
8	1	Male
8	1	Male
8	2	Female
8	2	Female
8	1	Male
8	1	Male
8	2	Female
8	2	Female
8	1	Male
8	1	Male
8	2	Female
8	2	Female
8	1	Male
8	1	Male
8	2	Female
8	2	Female
8	1	Male
8	1	Male
8	2	Female
8	2	Female
8	1	Male
8	1	Male
8	2	Female
8	2	Female
8	1	Male
8	1	Male
8	2	Female
8	2	Female
8	1	Male
8	1	Male
8	2	Female
8	2	Female
8	1	Male
8	1	Male
/pu/
/pu/
/pu/
/pu/
/ci/
/ci/
/ci/
/ci/
/fa/
/fa/
/fa/
/fa/
/pu/
/pu/
/pu/
/pu/
/ci/
/ci/
/ci/
/ci/
/fa/
/fa/
/fa/
/fa/
/pu/
/pu/
/pu/
/pu/
/ci/
/ci/
/ci/
/ci/
/fa/
/fa/
/fa/
/fa/
/pu/
/pu/
/pu/
/pu/
Fear
Fear
Fear
Fear
Fear
Fear
Fear
Fear
Fear
Fear
Fear
Fear
Fear
Fear
Fear
Fear
Fear
Fear
Fear
Fear
Fear
Fear
Fear
Fear
Fear
Fear
Fear
Fear
Fear
Fear
Fear
Fear
Fear
Fear
Fear
Fear
Fear
Fear
Fear
Fear
Tone 1In isolation Correct Tone 1In isolation Correct Tone 1In isolation Correct Tone 1In isolation Correct Tone 2In isolation Incorrect Tone 2In isolation Incorrect Tone 2In isolation Correct Tone 2In isolation Correct Tone 2In isolation Correct Tone 2In isolation Correct Tone 2In isolation Correct Tone 2In isolation Correct Tone 2In isolation Incorrect Tone 2In isolation Incorrect Tone 2In isolation Incorrect Tone 2In isolation Correct Tone 3In isolation Incorrect Tone 3In isolation Incorrect Tone 3In isolation Correct Tone 3In isolation Incorrect Tone 3In isolation Incorrect Tone 3In isolation Correct Tone 3In isolation Correct Tone 3In isolation Correct Tone 3In isolation Incorrect Tone 3In isolation Incorrect Tone 3In isolation Incorrect Tone 3In isolation Incorrect Tone 4In isolation Incorrect Tone 4In isolation Incorrect Tone 4In isolation Incorrect Tone 4In isolation Incorrect Tone 4In isolation Correct Tone 4In isolation Correct Tone 4In isolation Correct Tone 4In isolation Incorrect Tone 4In isolation Correct Tone 4In isolation Correct Tone 4In isolation Incorrect Tone 4In isolation Incorrect

8	2	Female
8	2	Female
8	1	Male
8	1	Male
8	2	Female
8	2	Female
8	1	Male
8	1	Male
8	2	Female
8	2	Female
8	1	Male
/ci/
/ci/
/ci/
/ci/
/fa/
/fa/
/fa/
/fa/
/pu/
/pu/
/pu/
HappinesTone 1In isolation Correct
HappinesTone 1In isolation Correct
HappinesTone 1In isolation Correct
HappinesTone 1In isolation Correct
HappinesTone 1In isolation Incorrect
HappinesTone 1In isolation Incorrect
HappinesTone 1In isolation Incorrect
HappinesTone 1In isolation Incorrect
HappinesTone 1In isolation Incorrect
HappinesTone 1In isolation Incorrect
HappinesTone 1In isolation Correct

8	1	Male
8	2	Female
8	2	Female
8	1	Male
8	1	Male
8	2	Female
8	2	Female
8	1	Male
8	1	Male
8	2	Female
8	2	Female
8	1	Male
8	1	Male
8	2	Female
8	2	Female
8	1	Male
8	1	Male
8	2	Female
8	2	Female
8	1	Male
8	1	Male
8	2	Female
8	2	Female
8	1	Male
8	1	Male
8	2	Female
8	2	Female
8	1	Male
8	1	Male
8	2	Female
8	2	Female
8	1	Male
8	1	Male
8	2	Female
8	2	Female
8	1	Male
8	1	Male
/pu/
/ci/
/ci/
/ci/
/ci/
/fa/
/fa/
/fa/
/fa/
/pu/
/pu/
/pu/
/pu/
/ci/
/ci/
/ci/
/ci/
/fa/
/fa/
/fa/
/fa/
/pu/
/pu/
/pu/
/pu/
/ci/
/ci/
/ci/
/ci/
/fa/
/fa/
/fa/
/fa/
/pu/
/pu/
/pu/
/pu/
HappinesTone 1In isolation Correct
HappinesTone 2In isolation Incorrect
HappinesTone 2In isolation Correct
HappinesTone 2In isolation Correct
HappinesTone 2In isolation Correct
HappinesTone 2In isolation Correct
HappinesTone 2In isolation Correct
HappinesTone 2In isolation Correct
HappinesTone 2In isolation Correct
HappinesTone 2In isolation Incorrect
HappinesTone 2In isolation Incorrect
HappinesTone 2In isolation Correct
HappinesTone 2In isolation Correct
HappinesTone 3In isolation Incorrect
HappinesTone 3In isolation Correct
HappinesTone 3In isolation Correct
HappinesTone 3In isolation Correct
HappinesTone 3In isolation Correct
HappinesTone 3In isolation Correct
HappinesTone 3In isolation Correct
HappinesTone 3In isolation Correct
HappinesTone 3In isolation Correct
HappinesTone 3In isolation Correct
HappinesTone 3In isolation Correct
HappinesTone 3In isolation Correct
HappinesTone 4In isolation Incorrect
HappinesTone 4In isolation Incorrect
HappinesTone 4In isolation Incorrect
HappinesTone 4In isolation Correct
HappinesTone 4In isolation Incorrect
HappinesTone 4In isolation Correct
HappinesTone 4In isolation Correct
HappinesTone 4In isolation Correct
HappinesTone 4In isolation Incorrect
HappinesTone 4In isolation Incorrect
HappinesTone 4In isolation Correct
HappinesTone 4In isolation Incorrect

8	2	Female
8	2	Female
8	1	Male
8	1	Male
8	2	Female
8	2	Female
8	1	Male
8	1	Male
8	2	Female
8	2	Female
8	1	Male
8	1	Male
8	2	Female
8	2	Female
/ci/
/ci/
/ci/
/ci/
/fa/
/fa/
/fa/
/fa/
/pu/
/pu/
/pu/
/pu/
/ci/
/ci/
Neutral Tone 1In isolation Correct
Neutral Tone 1In isolation Correct
Neutral Tone 1In isolation Correct
Neutral Tone 1In isolation Correct
Neutral Tone 1In isolation Correct
Neutral Tone 1In isolation Correct
Neutral Tone 1In isolation Correct
Neutral Tone 1In isolation Correct
Neutral Tone 1In isolation Correct
Neutral Tone 1In isolation Correct
Neutral Tone 1In isolation Correct
Neutral Tone 1In isolation Correct
Neutral Tone 2In isolation Correct
Neutral Tone 2In isolation Correct

8	1	Male	/ci/	1	Neutral	Tone 2	In isolation	Correct	
8	1	Male	/ci/	2	Neutral	Tone 2	In isolation	Incorrect	
8	2	Female	/fa/	1	Neutral	Tone 2	In isolation	Correct	
8	2	Female	/fa/	2	Neutral	Tone 2	In isolation	Correct	
8	1	Male	/fa/	1	Neutral	Tone 2	In isolation	Correct	
8	1	Male	/fa/	2	Neutral	Tone 2	In isolation	Correct	
8	2	Female	/pu/	1	Neutral	Tone 2	In isolation	Correct	
8	2	Female	/pu/	2	Neutral	Tone 2	In isolation	Correct	
8	1	Male	/pu/	1	Neutral	Tone 2	In isolation	Correct	
8	1	Male	/pu/	2	Neutral	Tone 2	In isolation	Correct	
8	2	Female	/ci/	1	Neutral	Tone 3	In isolation	Correct	
8	2	Female	/ci/	2	Neutral	Tone 3	In isolation	Correct	
8	1	Male	/ci/	1	Neutral	Tone 3	In isolation	Correct	
8	1	Male	/ci/	2	Neutral	Tone 3	In isolation	Correct	
8	2	Female	/fa/	1	Neutral	Tone 3	In isolation	Correct	
8	2	Female	/fa/	2	Neutral	Tone 3	In isolation	Correct	
8	1	Male	/fa/	1	Neutral	Tone 3	In isolation	Correct	
8	1	Male	/fa/	2	Neutral	Tone 3	In isolation	Correct	
8	2	Female	/pu/	1	Neutral	Tone 3	In isolation	Correct	
8	2	Female	/pu/	2	Neutral	Tone 3	In isolation	Correct	
8	1	Male	/pu/	1	Neutral	Tone 3	In isolation	Correct	
8	1	Male	/pu/	2	Neutral	Tone 3	In isolation	Correct	
8	2	Female	/ci/	1	Neutral	Tone 4	In isolation	Correct	
8	2	Female	/ci/	2	Neutral	Tone 4	In isolation	Correct	
8	1	Male	/ci/	1	Neutral	Tone 4	In isolation	Correct	
8	1	Male	/ci/	2	Neutral	Tone 4	In isolation	Correct	
8	2	Female	/fa/	1	Neutral	Tone 4	In isolation	Correct	
8	2	Female	/fa/	2	Neutral	Tone 4	In isolation	Correct	
8	1	Male	/fa/	1	Neutral	Tone 4	In isolation	Correct	
8	1	Male	/fa/	2	Neutral	Tone 4	In isolation	Correct	
8	2	Female	/pu/	1	Neutral	Tone 4	In isolation	Correct	
8	2	Female	/pu/	2	Neutral	Tone 4	In isolation	Correct	
8	1	Male	/pu/	1	Neutral	Tone 4	In isolation	Correct	
8	1	Male	/pu/	2	Neutral	Tone 4	In isolation	Correct	
8	2	Female	/ci/	1	Sadness	Tone 1	In isolation	Correct	
8	2	Female	/ci/	2	Sadness	Tone 1	In isolation	Correct	
8	1	Male	/ci/	1	Sadness	Tone 1	In isolation	Correct	
8	1	Male	/ci/	2	Sadness	Tone 1	In isolation	Correct	
8	2	Female	/fa/	1	Sadness	Tone 1	In isolation	Correct	
8	2	Female	/fa/	2	Sadness	Tone 1	In isolation	Correct	
8	1	Male	/fa/	1	Sadness	Tone 1	In isolation	Incorrect	
8	1	Male	/fa/	2	Sadness	Tone 1	In isolation	Correct	
8	2	Female	/pu/	1	Sadness	Tone 1	In isolation	Correct	
8	2	Female	/pu/	2	Sadness	Tone 1	In isolation	Incorrect	
8	1	Male	/pu/	1	Sadness	Tone 1	In isolation	Correct	
8	1	Male	/pu/	2	Sadness	Tone 1	In isolation	Correct	
8	2	Female	/ci/	1	Sadness	Tone 2	In isolation	Correct	
8	2	Female	/ci/	2	Sadness	Tone 2	In isolation	Incorrect	
8	1	Male	/ci/	1	Sadness	Tone 2	In isolation	Incorrect	
8	1	Male	/ci/	2	Sadness	Tone 2	In isolation	Incorrect	
8	2	Female	/fa/	1	Sadness	Tone 2	In isolation	Correct	

8	2	Female	/fa/	2	Sadness	Tone 2	In isolation	Correct	
8	1	Male	/fa/	1	Sadness	Tone 2	In isolation	Correct	
8	1	Male	/fa/	2	Sadness	Tone 2	In isolation	Correct	
8	2	Female	/pu/	1	Sadness	Tone 2	In isolation	Correct	
8	2	Female	/pu/	2	Sadness	Tone 2	In isolation	Correct	
8	1	Male	/pu/	1	Sadness	Tone 2	In isolation	Incorrect	
8	1	Male	/pu/	2	Sadness	Tone 2	In isolation	Incorrect	
8	2	Female	/ci/	1	Sadness	Tone 3	In isolation	Correct	
8	2	Female	/ci/	2	Sadness	Tone 3	In isolation	Incorrect	
8	1	Male	/ci/	1	Sadness	Tone 3	In isolation	Correct	
8	1	Male	/ci/	2	Sadness	Tone 3	In isolation	Incorrect	
8	2	Female	/fa/	1	Sadness	Tone 3	In isolation	Incorrect	
8	2	Female	/fa/	2	Sadness	Tone 3	In isolation	Incorrect	
8	1	Male	/fa/	1	Sadness	Tone 3	In isolation	Correct	
8	1	Male	/fa/	2	Sadness	Tone 3	In isolation	Incorrect	
8	2	Female	/pu/	1	Sadness	Tone 3	In isolation	Correct	
8	2	Female	/pu/	2	Sadness	Tone 3	In isolation	Correct	
8	1	Male	/pu/	1	Sadness	Tone 3	In isolation	Correct	
8	1	Male	/pu/	2	Sadness	Tone 3	In isolation	Incorrect	
8	2	Female	/ci/	1	Sadness	Tone 4	In isolation	Incorrect	
8	2	Female	/ci/	2	Sadness	Tone 4	In isolation	Correct	
8	1	Male	/ci/	1	Sadness	Tone 4	In isolation	Incorrect	
8	1	Male	/ci/	2	Sadness	Tone 4	In isolation	Correct	
8	2	Female	/fa/	1	Sadness	Tone 4	In isolation	Incorrect	
8	2	Female	/fa/	2	Sadness	Tone 4	In isolation	Incorrect	
8	1	Male	/fa/	1	Sadness	Tone 4	In isolation	Incorrect	
8	1	Male	/fa/	2	Sadness	Tone 4	In isolation	Correct	
8	2	Female	/pu/	1	Sadness	Tone 4	In isolation	Incorrect	
8	2	Female	/pu/	2	Sadness	Tone 4	In isolation	Incorrect	
8	1	Male	/pu/	1	Sadness	Tone 4	In isolation	Incorrect	
8	1	Male	/pu/	2	Sadness	Tone 4	In isolation	Incorrect	
8	2	Female	/ci/	1	Anger	Tone 1	In context	Correct	
8	2	Female	/ci/	2	Anger	Tone 1	In context	Correct	
8	1	Male	/ci/	1	Anger	Tone 1	In context	Correct	
8	1	Male	/ci/	2	Anger	Tone 1	In context	Correct	
8	2	Female	/fa/	1	Anger	Tone 1	In context	Correct	
8	2	Female	/fa/	2	Anger	Tone 1	In context	Correct	
8	1	Male	/fa/	1	Anger	Tone 1	In context	Correct	
8	1	Male	/fa/	2	Anger	Tone 1	In context	Correct	
8	2	Female	/pu/	1	Anger	Tone 1	In context	Incorrect	
8	2	Female	/pu/	2	Anger	Tone 1	In context	Incorrect	
8	1	Male	/pu/	1	Anger	Tone 1	In context	Correct	
8	1	Male	/pu/	2	Anger	Tone 1	In context	Correct	
8	2	Female	/ci/	1	Anger	Tone 2	In context	Correct	
8	2	Female	/ci/	2	Anger	Tone 2	In context	Correct	
8	1	Male	/ci/	1	Anger	Tone 2	In context	Correct	
8	1	Male	/ci/	2	Anger	Tone 2	In context	Correct	
8	2	Female	/fa/	1	Anger	Tone 2	In context	Correct	
8	2	Female	/fa/	2	Anger	Tone 2	In context	Correct	
8	1	Male	/fa/	1	Anger	Tone 2	In context	Correct	
8	1	Male	/fa/	2	Anger	Tone 2	In context	Correct	

8	2	Female	/pu/	1	Anger	Tone 2	In context	Correct	
8	2	Female	/pu/	2	Anger	Tone 2	In context	Correct	
8	1	Male	/pu/	1	Anger	Tone 2	In context	Correct	
8	1	Male	/pu/	2	Anger	Tone 2	In context	Correct	
8	2	Female	/ci/	1	Anger	Tone 3	In context	Correct	
8	2	Female	/ci/	2	Anger	Tone 3	In context	Correct	
8	1	Male	/ci/	1	Anger	Tone 3	In context	Correct	
8	1	Male	/ci/	2	Anger	Tone 3	In context	Correct	
8	2	Female	/fa/	1	Anger	Tone 3	In context	Correct	
8	2	Female	/fa/	2	Anger	Tone 3	In context	Correct	
8	1	Male	/fa/	1	Anger	Tone 3	In context	Correct	
8	1	Male	/fa/	2	Anger	Tone 3	In context	Correct	
8	2	Female	/pu/	1	Anger	Tone 3	In context	Correct	
8	2	Female	/pu/	2	Anger	Tone 3	In context	Correct	
8	1	Male	/pu/	1	Anger	Tone 3	In context	Correct	
8	1	Male	/pu/	2	Anger	Tone 3	In context	Correct	
8	2	Female	/ci/	1	Anger	Tone 4	In context	Correct	
8	2	Female	/ci/	2	Anger	Tone 4	In context	Correct	
8	1	Male	/ci/	1	Anger	Tone 4	In context	Correct	
8	1	Male	/ci/	2	Anger	Tone 4	In context	Correct	
8	2	Female	/fa/	1	Anger	Tone 4	In context	Correct	
8	2	Female	/fa/	2	Anger	Tone 4	In context	Correct	
8	1	Male	/fa/	1	Anger	Tone 4	In context	Correct	
8	1	Male	/fa/	2	Anger	Tone 4	In context	Correct	
8	2	Female	/pu/	1	Anger	Tone 4	In context	Correct	
8	2	Female	/pu/	2	Anger	Tone 4	In context	Correct	
8	1	Male	/pu/	1	Anger	Tone 4	In context	Correct	
8	1	Male	/pu/	2	Anger	Tone 4	In context	Correct	
8	2	Female	/ci/	1	Fear	Tone 1	In context	Correct	
8	2	Female	/ci/	2	Fear	Tone 1	In context	Correct	
8	1	Male	/ci/	1	Fear	Tone 1	In context	Correct	
8	1	Male	/ci/	2	Fear	Tone 1	In context	Correct	
8	2	Female	/fa/	1	Fear	Tone 1	In context	Correct	
8	2	Female	/fa/	2	Fear	Tone 1	In context	Correct	
8	1	Male	/fa/	1	Fear	Tone 1	In context	Correct	
8	1	Male	/fa/	2	Fear	Tone 1	In context	Correct	
8	2	Female	/pu/	1	Fear	Tone 1	In context	Correct	
8	2	Female	/pu/	2	Fear	Tone 1	In context	Correct	
8	1	Male	/pu/	1	Fear	Tone 1	In context	Correct	
8	1	Male	/pu/	2	Fear	Tone 1	In context	Correct	
8	2	Female	/ci/	1	Fear	Tone 2	In context	Correct	
8	2	Female	/ci/	2	Fear	Tone 2	In context	Correct	
8	1	Male	/ci/	1	Fear	Tone 2	In context	Correct	
8	1	Male	/ci/	2	Fear	Tone 2	In context	Correct	
8	2	Female	/fa/	1	Fear	Tone 2	In context	Correct	
8	2	Female	/fa/	2	Fear	Tone 2	In context	Correct	
8	1	Male	/fa/	1	Fear	Tone 2	In context	Correct	
8	1	Male	/fa/	2	Fear	Tone 2	In context	Correct	
8	2	Female	/pu/	1	Fear	Tone 2	In context	Correct	
8	2	Female	/pu/	2	Fear	Tone 2	In context	Incorrect	
8	1	Male	/pu/	1	Fear	Tone 2	In context	Correct	

8	1	Male
/pu/
2	Fear
Tone 2 In context
Correct

8	2	Female
8	2	Female
/ci/
/ci/
Fear
Fear
Tone 3 In context Incorrect Tone 3 In context Incorrect

8	1	Male
8	1	Male
8	2	Female
8	2	Female
8	1	Male
8	1	Male
8	2	Female
/ci/
/ci/
/fa/
/fa/
/fa/
/fa/
/pu/
Fear
Fear
Fear
Fear
Fear
Fear
Fear
Tone 3 In context Tone 3 In context Tone 3 In context Tone 3 In context Tone 3 In context Tone 3 In context Tone 3 In context
Correct Correct Correct Correct Correct Correct Correct

8	2	Female
/pu/
Fear
Tone 3 In context Incorrect

8	1	Male
8	1	Male
8	2	Female
8	2	Female
8	1	Male
8	1	Male
8	2	Female
8	2	Female
8	1	Male
8	1	Male
8	2	Female
8	2	Female
8	1	Male
8	1	Male
/pu/
/pu/
/ci/
/ci/
/ci/
/ci/
/fa/
/fa/
/fa/
/fa/
/pu/
/pu/
/pu/
/pu/
Fear
Fear
Fear
Fear
Fear
Fear
Fear
Fear
Fear
Fear
Fear
Fear
Fear
Fear
Tone 3 In context Tone 3 In context Tone 4 In context Tone 4 In context Tone 4 In context Tone 4 In context Tone 4 In context Tone 4 In context Tone 4 In context Tone 4 In context Tone 4 In context Tone 4 In context Tone 4 In context Tone 4 In context
Correct Correct Correct Correct Correct Correct Correct Correct Correct Correct Correct Correct Correct Correct

8	2	Female
8	2	Female
8	1	Male
8	1	Male
8	2	Female
8	2	Female
8	1	Male
8	1	Male
8	2	Female
8	2	Female
8	1	Male
/ci/
/ci/
/ci/
/ci/
/fa/
/fa/
/fa/
/fa/
/pu/
/pu/
/pu/
HappinesTone 1 In context
HappinesTone 1 In context
HappinesTone 1 In context
HappinesTone 1 In context
HappinesTone 1 In context
HappinesTone 1 In context
HappinesTone 1 In context
HappinesTone 1 In context
HappinesTone 1 In context
HappinesTone 1 In context
HappinesTone 1 In context
Correct Correct Correct Correct Correct Correct Correct Correct Correct Correct Correct

8	1	Male
/pu/
HappinesTone 1 In context Incorrect

8	2	Female
8	2	Female
8	1	Male
8	1	Male
8	2	Female
8	2	Female
8	1	Male
8	1	Male
8	2	Female
8	2	Female
8	1	Male
8	1	Male
8	2	Female
8	2	Female
/ci/
/ci/
/ci/
/ci/
/fa/
/fa/
/fa/
/fa/
/pu/
/pu/
/pu/
/pu/
/ci/
/ci/
HappinesTone 2 In context
HappinesTone 2 In context
HappinesTone 2 In context
HappinesTone 2 In context
HappinesTone 2 In context
HappinesTone 2 In context
HappinesTone 2 In context
HappinesTone 2 In context
HappinesTone 2 In context
HappinesTone 2 In context
HappinesTone 2 In context
HappinesTone 2 In context
HappinesTone 3 In context
HappinesTone 3 In context
Correct Correct Correct Correct Correct Correct Correct Correct Correct Correct Correct Correct Correct Correct

8	1	Male
8	1	Male
8	2	Female
8	2	Female
8	1	Male
8	1	Male
8	2	Female
8	2	Female
8	1	Male
8	1	Male
/ci/
/ci/
/fa/
/fa/
/fa/
/fa/
/pu/
/pu/
/pu/
/pu/
HappinesTone 3 In context
HappinesTone 3 In context
HappinesTone 3 In context
HappinesTone 3 In context
HappinesTone 3 In context
HappinesTone 3 In context
HappinesTone 3 In context
HappinesTone 3 In context
HappinesTone 3 In context
HappinesTone 3 In context
Correct Correct Correct Correct Correct Correct Correct Correct Correct Correct

8	2	Female
8	2	Female
/ci/
/ci/
HappinesTone 4 In context Incorrect
HappinesTone 4 In context Incorrect

8	1	Male
8	1	Male
8	2	Female
8	2	Female
8	1	Male
8	1	Male
8	2	Female
8	2	Female
8	1	Male
8	1	Male
/ci/
/ci/
/fa/
/fa/
/fa/
/fa/
/pu/
/pu/
/pu/
/pu/
HappinesTone 4 In context
HappinesTone 4 In context
HappinesTone 4 In context
HappinesTone 4 In context
HappinesTone 4 In context
HappinesTone 4 In context
HappinesTone 4 In context
HappinesTone 4 In context
HappinesTone 4 In context
HappinesTone 4 In context
Correct Correct Correct Correct Correct Correct Correct Correct Correct Correct

8	2	Female
8	2	Female
8	1	Male
8	1	Male
8	2	Female
8	2	Female
8	1	Male
8	1	Male
8	2	Female
8	2	Female
8	1	Male
8	1	Male
8	2	Female
8	2	Female
8	1	Male
8	1	Male
8	2	Female
8	2	Female
8	1	Male
8	1	Male
8	2	Female
8	2	Female
8	1	Male
8	1	Male
8	2	Female
8	2	Female
8	1	Male
8	1	Male
8	2	Female
/ci/
/ci/
/ci/
/ci/
/fa/
/fa/
/fa/
/fa/
/pu/
/pu/
/pu/
/pu/
/ci/
/ci/
/ci/
/ci/
/fa/
/fa/
/fa/
/fa/
/pu/
/pu/
/pu/
/pu/
/ci/
/ci/
/ci/
/ci/
/fa/
Neutral Tone 1 In context
Neutral Tone 1 In context
Neutral Tone 1 In context
Neutral Tone 1 In context
Neutral Tone 1 In context
Neutral Tone 1 In context
Neutral Tone 1 In context
Neutral Tone 1 In context
Neutral Tone 1 In context
Neutral Tone 1 In context
Neutral Tone 1 In context
Neutral Tone 1 In context
Neutral Tone 2 In context
Neutral Tone 2 In context
Neutral Tone 2 In context
Neutral Tone 2 In context
Neutral Tone 2 In context
Neutral Tone 2 In context
Neutral Tone 2 In context
Neutral Tone 2 In context
Neutral Tone 2 In context
Neutral Tone 2 In context
Neutral Tone 2 In context
Neutral Tone 2 In context
Neutral Tone 3 In context
Neutral Tone 3 In context
Neutral Tone 3 In context
Neutral Tone 3 In context
1	Neutral Tone 3 In context
Correct Correct Correct Correct Correct Correct Correct Correct Correct Correct Correct Correct Correct Correct Correct Correct Correct Correct Correct Correct Correct Correct Correct Correct Correct Correct Correct Correct Correct

8	2	Female	/fa/	2	Neutral	Tone 3	In context	Incorrect	
8	1	Male	/fa/	1	Neutral	Tone 3	In context	Correct	
8	1	Male	/fa/	2	Neutral	Tone 3	In context	Correct	
8	2	Female	/pu/	1	Neutral	Tone 3	In context	Correct	
8	2	Female	/pu/	2	Neutral	Tone 3	In context	Correct	
8	1	Male	/pu/	1	Neutral	Tone 3	In context	Correct	
8	1	Male	/pu/	2	Neutral	Tone 3	In context	Correct	
8	2	Female	/ci/	1	Neutral	Tone 4	In context	Correct	
8	2	Female	/ci/	2	Neutral	Tone 4	In context	Correct	
8	1	Male	/ci/	1	Neutral	Tone 4	In context	Correct	
8	1	Male	/ci/	2	Neutral	Tone 4	In context	Correct	
8	2	Female	/fa/	1	Neutral	Tone 4	In context	Correct	
8	2	Female	/fa/	2	Neutral	Tone 4	In context	Correct	
8	1	Male	/fa/	1	Neutral	Tone 4	In context	Correct	
8	1	Male	/fa/	2	Neutral	Tone 4	In context	Correct	
8	2	Female	/pu/	1	Neutral	Tone 4	In context	Correct	
8	2	Female	/pu/	2	Neutral	Tone 4	In context	Correct	
8	1	Male	/pu/	1	Neutral	Tone 4	In context	Correct	
8	1	Male	/pu/	2	Neutral	Tone 4	In context	Correct	
8	2	Female	/ci/	1	Sadness	Tone 1	In context	Correct	
8	2	Female	/ci/	2	Sadness	Tone 1	In context	Correct	
8	1	Male	/ci/	1	Sadness	Tone 1	In context	Correct	
8	1	Male	/ci/	2	Sadness	Tone 1	In context	Correct	
8	2	Female	/fa/	1	Sadness	Tone 1	In context	Correct	
8	2	Female	/fa/	2	Sadness	Tone 1	In context	Correct	
8	1	Male	/fa/	1	Sadness	Tone 1	In context	Incorrect	
8	1	Male	/fa/	2	Sadness	Tone 1	In context	Correct	
8	2	Female	/pu/	1	Sadness	Tone 1	In context	Correct	
8	2	Female	/pu/	2	Sadness	Tone 1	In context	Correct	
8	1	Male	/pu/	1	Sadness	Tone 1	In context	Correct	
8	1	Male	/pu/	2	Sadness	Tone 1	In context	Correct	
8	2	Female	/ci/	1	Sadness	Tone 2	In context	Correct	
8	2	Female	/ci/	2	Sadness	Tone 2	In context	Correct	
8	1	Male	/ci/	1	Sadness	Tone 2	In context	Correct	
8	1	Male	/ci/	2	Sadness	Tone 2	In context	Correct	
8	2	Female	/fa/	1	Sadness	Tone 2	In context	Correct	
8	2	Female	/fa/	2	Sadness	Tone 2	In context	Correct	
8	1	Male	/fa/	1	Sadness	Tone 2	In context	Correct	
8	1	Male	/fa/	2	Sadness	Tone 2	In context	Correct	
8	2	Female	/pu/	1	Sadness	Tone 2	In context	Correct	
8	2	Female	/pu/	2	Sadness	Tone 2	In context	Correct	
8	1	Male	/pu/	1	Sadness	Tone 2	In context	Correct	
8	1	Male	/pu/	2	Sadness	Tone 2	In context	Correct	
8	2	Female	/ci/	1	Sadness	Tone 3	In context	Correct	
8	2	Female	/ci/	2	Sadness	Tone 3	In context	Incorrect	
8	1	Male	/ci/	1	Sadness	Tone 3	In context	Correct	
8	1	Male	/ci/	2	Sadness	Tone 3	In context	Correct	
8	2	Female	/fa/	1	Sadness	Tone 3	In context	Correct	
8	2	Female	/fa/	2	Sadness	Tone 3	In context	Correct	
8	1	Male	/fa/	1	Sadness	Tone 3	In context	Correct	
8	1	Male	/fa/	2	Sadness	Tone 3	In context	Correct	

8	2	Female	/pu/	1	Sadness	Tone 3	In context	Incorrect	
8	2	Female	/pu/	2	Sadness	Tone 3	In context	Correct	
8	1	Male	/pu/	1	Sadness	Tone 3	In context	Correct	
8	1	Male	/pu/	2	Sadness	Tone 3	In context	Correct	
8	2	Female	/ci/	1	Sadness	Tone 4	In context	Correct	
8	2	Female	/ci/	2	Sadness	Tone 4	In context	Correct	
8	1	Male	/ci/	1	Sadness	Tone 4	In context	Correct	
8	1	Male	/ci/	2	Sadness	Tone 4	In context	Correct	
8	2	Female	/fa/	1	Sadness	Tone 4	In context	Correct	
8	2	Female	/fa/	2	Sadness	Tone 4	In context	Correct	
8	1	Male	/fa/	1	Sadness	Tone 4	In context	Correct	
8	1	Male	/fa/	2	Sadness	Tone 4	In context	Correct	
8	2	Female	/pu/	1	Sadness	Tone 4	In context	Correct	
8	2	Female	/pu/	2	Sadness	Tone 4	In context	Correct	
8	1	Male	/pu/	1	Sadness	Tone 4	In context	Correct	
8	1	Male	/pu/	2	Sadness	Tone 4	In context	Correct	
9	2	Female	/ci/	1	Anger	Tone 1	In isolation	Correct	
9	2	Female	/ci/	2	Anger	Tone 1	In isolation	Correct	
9	1	Male	/ci/	1	Anger	Tone 1	In isolation	Correct	
9	1	Male	/ci/	2	Anger	Tone 1	In isolation	Correct	
9	2	Female	/fa/	1	Anger	Tone 1	In isolation	Correct	
9	2	Female	/fa/	2	Anger	Tone 1	In isolation	Correct	
9	1	Male	/fa/	1	Anger	Tone 1	In isolation	Correct	
9	1	Male	/fa/	2	Anger	Tone 1	In isolation	Correct	
9	2	Female	/pu/	1	Anger	Tone 1	In isolation	Correct	
9	2	Female	/pu/	2	Anger	Tone 1	In isolation	Correct	
9	1	Male	/pu/	1	Anger	Tone 1	In isolation	Correct	
9	1	Male	/pu/	2	Anger	Tone 1	In isolation	Correct	
9	2	Female	/ci/	1	Anger	Tone 2	In isolation	Incorrect	
9	2	Female	/ci/	2	Anger	Tone 2	In isolation	Incorrect	
9	1	Male	/ci/	1	Anger	Tone 2	In isolation	Incorrect	
9	1	Male	/ci/	2	Anger	Tone 2	In isolation	Incorrect	
9	2	Female	/fa/	1	Anger	Tone 2	In isolation	Correct	
9	2	Female	/fa/	2	Anger	Tone 2	In isolation	Correct	
9	1	Male	/fa/	1	Anger	Tone 2	In isolation	Correct	
9	1	Male	/fa/	2	Anger	Tone 2	In isolation	Incorrect	
9	2	Female	/pu/	1	Anger	Tone 2	In isolation	Correct	
9	2	Female	/pu/	2	Anger	Tone 2	In isolation	Incorrect	
9	1	Male	/pu/	1	Anger	Tone 2	In isolation	Correct	
9	1	Male	/pu/	2	Anger	Tone 2	In isolation	Correct	
9	2	Female	/ci/	1	Anger	Tone 3	In isolation	Incorrect	
9	2	Female	/ci/	2	Anger	Tone 3	In isolation	Incorrect	
9	1	Male	/ci/	1	Anger	Tone 3	In isolation	Incorrect	
9	1	Male	/ci/	2	Anger	Tone 3	In isolation	Incorrect	
9	2	Female	/fa/	1	Anger	Tone 3	In isolation	Correct	
9	2	Female	/fa/	2	Anger	Tone 3	In isolation	Correct	
9	1	Male	/fa/	1	Anger	Tone 3	In isolation	Correct	
9	1	Male	/fa/	2	Anger	Tone 3	In isolation	Correct	
9	2	Female	/pu/	1	Anger	Tone 3	In isolation	Incorrect	
9	2	Female	/pu/	2	Anger	Tone 3	In isolation	Incorrect	
9	1	Male	/pu/	1	Anger	Tone 3	In isolation	Incorrect	

9	1	Male	/pu/	2	Anger	Tone 3	In isolation	Correct	
9	2	Female	/ci/	1	Anger	Tone 4	In isolation	Incorrect	
9	2	Female	/ci/	2	Anger	Tone 4	In isolation	Incorrect	
9	1	Male	/ci/	1	Anger	Tone 4	In isolation	Correct	
9	1	Male	/ci/	2	Anger	Tone 4	In isolation	Correct	
9	2	Female	/fa/	1	Anger	Tone 4	In isolation	Correct	
9	2	Female	/fa/	2	Anger	Tone 4	In isolation	Correct	
9	1	Male	/fa/	1	Anger	Tone 4	In isolation	Correct	
9	1	Male	/fa/	2	Anger	Tone 4	In isolation	Correct	
9	2	Female	/pu/	1	Anger	Tone 4	In isolation	Correct	
9	2	Female	/pu/	2	Anger	Tone 4	In isolation	Correct	
9	1	Male	/pu/	1	Anger	Tone 4	In isolation	Correct	
9	1	Male	/pu/	2	Anger	Tone 4	In isolation	Correct	
9	2	Female	/ci/	1	Fear	Tone 1	In isolation	Correct	
9	2	Female	/ci/	2	Fear	Tone 1	In isolation	Correct	
9	1	Male	/ci/	1	Fear	Tone 1	In isolation	Correct	
9	1	Male	/ci/	2	Fear	Tone 1	In isolation	Correct	
9	2	Female	/fa/	1	Fear	Tone 1	In isolation	Correct	
9	2	Female	/fa/	2	Fear	Tone 1	In isolation	Correct	
9	1	Male	/fa/	1	Fear	Tone 1	In isolation	Correct	
9	1	Male	/fa/	2	Fear	Tone 1	In isolation	Correct	
9	2	Female	/pu/	1	Fear	Tone 1	In isolation	Correct	
9	2	Female	/pu/	2	Fear	Tone 1	In isolation	Correct	
9	1	Male	/pu/	1	Fear	Tone 1	In isolation	Correct	
9	1	Male	/pu/	2	Fear	Tone 1	In isolation	Correct	
9	2	Female	/ci/	1	Fear	Tone 2	In isolation	Incorrect	
9	2	Female	/ci/	2	Fear	Tone 2	In isolation	Incorrect	
9	1	Male	/ci/	1	Fear	Tone 2	In isolation	Incorrect	
9	1	Male	/ci/	2	Fear	Tone 2	In isolation	Correct	
9	2	Female	/fa/	1	Fear	Tone 2	In isolation	Correct	
9	2	Female	/fa/	2	Fear	Tone 2	In isolation	Correct	
9	1	Male	/fa/	1	Fear	Tone 2	In isolation	Correct	
9	1	Male	/fa/	2	Fear	Tone 2	In isolation	Correct	
9	2	Female	/pu/	1	Fear	Tone 2	In isolation	Correct	
9	2	Female	/pu/	2	Fear	Tone 2	In isolation	Incorrect	
9	1	Male	/pu/	1	Fear	Tone 2	In isolation	Incorrect	
9	1	Male	/pu/	2	Fear	Tone 2	In isolation	Incorrect	
9	2	Female	/ci/	1	Fear	Tone 3	In isolation	Incorrect	
9	2	Female	/ci/	2	Fear	Tone 3	In isolation	Incorrect	
9	1	Male	/ci/	1	Fear	Tone 3	In isolation	Correct	
9	1	Male	/ci/	2	Fear	Tone 3	In isolation	Correct	
9	2	Female	/fa/	1	Fear	Tone 3	In isolation	Incorrect	
9	2	Female	/fa/	2	Fear	Tone 3	In isolation	Incorrect	
9	1	Male	/fa/	1	Fear	Tone 3	In isolation	Correct	
9	1	Male	/fa/	2	Fear	Tone 3	In isolation	Correct	
9	2	Female	/pu/	1	Fear	Tone 3	In isolation	Correct	
9	2	Female	/pu/	2	Fear	Tone 3	In isolation	Correct	
9	1	Male	/pu/	1	Fear	Tone 3	In isolation	Incorrect	
9	1	Male	/pu/	2	Fear	Tone 3	In isolation	Correct	
9	2	Female	/ci/	1	Fear	Tone 4	In isolation	Correct	
9	2	Female	/ci/	2	Fear	Tone 4	In isolation	Correct	

9	1	Male
9	1	Male
9	2	Female
9	2	Female
9	1	Male
9	1	Male
9	2	Female
9	2	Female
9	1	Male
9	1	Male
/ci/
/ci/
/fa/
/fa/
/fa/
/fa/
/pu/
/pu/
/pu/
/pu/
Fear
Fear
Fear
Fear
Fear
Fear
Fear
Fear
Fear
Fear
Tone 4In isolation Correct Tone 4In isolation Correct Tone 4In isolation Correct Tone 4In isolation Correct Tone 4In isolation Correct Tone 4In isolation Correct Tone 4In isolation Correct Tone 4In isolation Correct Tone 4In isolation Incorrect Tone 4In isolation Correct

9	2	Female
9	2	Female
9	1	Male
9	1	Male
9	2	Female
9	2	Female
9	1	Male
9	1	Male
9	2	Female
9	2	Female
9	1	Male
9	1	Male
9	2	Female
9	2	Female
9	1	Male
9	1	Male
9	2	Female
9	2	Female
9	1	Male
9	1	Male
9	2	Female
9	2	Female
9	1	Male
9	1	Male
9	2	Female
9	2	Female
9	1	Male
9	1	Male
9	2	Female
9	2	Female
9	1	Male
9	1	Male
9	2	Female
9	2	Female
9	1	Male
9	1	Male
9	2	Female
9	2	Female
9	1	Male
9	1	Male
9	2	Female
/ci/
/ci/
/ci/
/ci/
/fa/
/fa/
/fa/
/fa/
/pu/
/pu/
/pu/
/pu/
/ci/
/ci/
/ci/
/ci/
/fa/
/fa/
/fa/
/fa/
/pu/
/pu/
/pu/
/pu/
/ci/
/ci/
/ci/
/ci/
/fa/
/fa/
/fa/
/fa/
/pu/
/pu/
/pu/
/pu/
/ci/
/ci/
/ci/
/ci/
/fa/
HappinesTone 1In isolation Correct
HappinesTone 1In isolation Incorrect
HappinesTone 1In isolation Correct
HappinesTone 1In isolation Correct
HappinesTone 1In isolation Incorrect
HappinesTone 1In isolation Incorrect
HappinesTone 1In isolation Correct
HappinesTone 1In isolation Correct
HappinesTone 1In isolation Incorrect
HappinesTone 1In isolation Incorrect
HappinesTone 1In isolation Correct
HappinesTone 1In isolation Correct
HappinesTone 2In isolation Correct
HappinesTone 2In isolation Correct
HappinesTone 2In isolation Incorrect
HappinesTone 2In isolation Incorrect
HappinesTone 2In isolation Correct
HappinesTone 2In isolation Correct
HappinesTone 2In isolation Correct
HappinesTone 2In isolation Correct
HappinesTone 2In isolation Correct
HappinesTone 2In isolation Incorrect
HappinesTone 2In isolation Correct
HappinesTone 2In isolation Correct
HappinesTone 3In isolation Correct
HappinesTone 3In isolation Correct
HappinesTone 3In isolation Correct
HappinesTone 3In isolation Correct
HappinesTone 3In isolation Incorrect
HappinesTone 3In isolation Correct
HappinesTone 3In isolation Correct
HappinesTone 3In isolation Correct
HappinesTone 3In isolation Correct
HappinesTone 3In isolation Incorrect
HappinesTone 3In isolation Correct
HappinesTone 3In isolation Incorrect
HappinesTone 4In isolation Correct
HappinesTone 4In isolation Incorrect
HappinesTone 4In isolation Incorrect
HappinesTone 4In isolation Correct
HappinesTone 4In isolation Incorrect

9	2	Female	/fa/	2	Happines	Tone 4	In isolation	Correct	
9	1	Male	/fa/	1	Happines	Tone 4	In isolation	Correct	
9	1	Male	/fa/	2	Happines	Tone 4	In isolation	Correct	
9	2	Female	/pu/	1	Happines	Tone 4	In isolation	Incorrect	
9	2	Female	/pu/	2	Happines	Tone 4	In isolation	Incorrect	
9	1	Male	/pu/	1	Happines	Tone 4	In isolation	Correct	
9	1	Male	/pu/	2	Happines	Tone 4	In isolation	Correct	
9	2	Female	/ci/	1	Neutral	Tone 1	In isolation	Correct	
9	2	Female	/ci/	2	Neutral	Tone 1	In isolation	Correct	
9	1	Male	/ci/	1	Neutral	Tone 1	In isolation	Correct	
9	1	Male	/ci/	2	Neutral	Tone 1	In isolation	Correct	
9	2	Female	/fa/	1	Neutral	Tone 1	In isolation	Correct	
9	2	Female	/fa/	2	Neutral	Tone 1	In isolation	Correct	
9	1	Male	/fa/	1	Neutral	Tone 1	In isolation	Correct	
9	1	Male	/fa/	2	Neutral	Tone 1	In isolation	Correct	
9	2	Female	/pu/	1	Neutral	Tone 1	In isolation	Correct	
9	2	Female	/pu/	2	Neutral	Tone 1	In isolation	Correct	
9	1	Male	/pu/	1	Neutral	Tone 1	In isolation	Correct	
9	1	Male	/pu/	2	Neutral	Tone 1	In isolation	Correct	
9	2	Female	/ci/	1	Neutral	Tone 2	In isolation	Correct	
9	2	Female	/ci/	2	Neutral	Tone 2	In isolation	Correct	
9	1	Male	/ci/	1	Neutral	Tone 2	In isolation	Correct	
9	1	Male	/ci/	2	Neutral	Tone 2	In isolation	Correct	
9	2	Female	/fa/	1	Neutral	Tone 2	In isolation	Correct	
9	2	Female	/fa/	2	Neutral	Tone 2	In isolation	Correct	
9	1	Male	/fa/	1	Neutral	Tone 2	In isolation	Correct	
9	1	Male	/fa/	2	Neutral	Tone 2	In isolation	Correct	
9	2	Female	/pu/	1	Neutral	Tone 2	In isolation	Correct	
9	2	Female	/pu/	2	Neutral	Tone 2	In isolation	Correct	
9	1	Male	/pu/	1	Neutral	Tone 2	In isolation	Correct	
9	1	Male	/pu/	2	Neutral	Tone 2	In isolation	Correct	
9	2	Female	/ci/	1	Neutral	Tone 3	In isolation	Correct	
9	2	Female	/ci/	2	Neutral	Tone 3	In isolation	Correct	
9	1	Male	/ci/	1	Neutral	Tone 3	In isolation	Correct	
9	1	Male	/ci/	2	Neutral	Tone 3	In isolation	Correct	
9	2	Female	/fa/	1	Neutral	Tone 3	In isolation	Correct	
9	2	Female	/fa/	2	Neutral	Tone 3	In isolation	Correct	
9	1	Male	/fa/	1	Neutral	Tone 3	In isolation	Correct	
9	1	Male	/fa/	2	Neutral	Tone 3	In isolation	Correct	
9	2	Female	/pu/	1	Neutral	Tone 3	In isolation	Correct	
9	2	Female	/pu/	2	Neutral	Tone 3	In isolation	Correct	
9	1	Male	/pu/	1	Neutral	Tone 3	In isolation	Correct	
9	1	Male	/pu/	2	Neutral	Tone 3	In isolation	Correct	
9	2	Female	/ci/	1	Neutral	Tone 4	In isolation	Correct	
9	2	Female	/ci/	2	Neutral	Tone 4	In isolation	Correct	
9	1	Male	/ci/	1	Neutral	Tone 4	In isolation	Correct	
9	1	Male	/ci/	2	Neutral	Tone 4	In isolation	Correct	
9	2	Female	/fa/	1	Neutral	Tone 4	In isolation	Correct	
9	2	Female	/fa/	2	Neutral	Tone 4	In isolation	Correct	
9	1	Male	/fa/	1	Neutral	Tone 4	In isolation	Correct	
9	1	Male	/fa/	2	Neutral	Tone 4	In isolation	Correct	

9	2	Female	/pu/	1	Neutral	Tone 4	In isolation	Correct	
9	2	Female	/pu/	2	Neutral	Tone 4	In isolation	Correct	
9	1	Male	/pu/	1	Neutral	Tone 4	In isolation	Correct	
9	1	Male	/pu/	2	Neutral	Tone 4	In isolation	Correct	
9	2	Female	/ci/	1	Sadness	Tone 1	In isolation	Correct	
9	2	Female	/ci/	2	Sadness	Tone 1	In isolation	Correct	
9	1	Male	/ci/	1	Sadness	Tone 1	In isolation	Correct	
9	1	Male	/ci/	2	Sadness	Tone 1	In isolation	Correct	
9	2	Female	/fa/	1	Sadness	Tone 1	In isolation	Correct	
9	2	Female	/fa/	2	Sadness	Tone 1	In isolation	Incorrect	
9	1	Male	/fa/	1	Sadness	Tone 1	In isolation	Incorrect	
9	1	Male	/fa/	2	Sadness	Tone 1	In isolation	Correct	
9	2	Female	/pu/	1	Sadness	Tone 1	In isolation	Correct	
9	2	Female	/pu/	2	Sadness	Tone 1	In isolation	Incorrect	
9	1	Male	/pu/	1	Sadness	Tone 1	In isolation	Correct	
9	1	Male	/pu/	2	Sadness	Tone 1	In isolation	Correct	
9	2	Female	/ci/	1	Sadness	Tone 2	In isolation	Correct	
9	2	Female	/ci/	2	Sadness	Tone 2	In isolation	Incorrect	
9	1	Male	/ci/	1	Sadness	Tone 2	In isolation	Incorrect	
9	1	Male	/ci/	2	Sadness	Tone 2	In isolation	Correct	
9	2	Female	/fa/	1	Sadness	Tone 2	In isolation	Incorrect	
9	2	Female	/fa/	2	Sadness	Tone 2	In isolation	Correct	
9	1	Male	/fa/	1	Sadness	Tone 2	In isolation	Incorrect	
9	1	Male	/fa/	2	Sadness	Tone 2	In isolation	Correct	
9	2	Female	/pu/	1	Sadness	Tone 2	In isolation	Correct	
9	2	Female	/pu/	2	Sadness	Tone 2	In isolation	Incorrect	
9	1	Male	/pu/	1	Sadness	Tone 2	In isolation	Incorrect	
9	1	Male	/pu/	2	Sadness	Tone 2	In isolation	Incorrect	
9	2	Female	/ci/	1	Sadness	Tone 3	In isolation	Correct	
9	2	Female	/ci/	2	Sadness	Tone 3	In isolation	Incorrect	
9	1	Male	/ci/	1	Sadness	Tone 3	In isolation	Correct	
9	1	Male	/ci/	2	Sadness	Tone 3	In isolation	Correct	
9	2	Female	/fa/	1	Sadness	Tone 3	In isolation	Correct	
9	2	Female	/fa/	2	Sadness	Tone 3	In isolation	Correct	
9	1	Male	/fa/	1	Sadness	Tone 3	In isolation	Correct	
9	1	Male	/fa/	2	Sadness	Tone 3	In isolation	Correct	
9	2	Female	/pu/	1	Sadness	Tone 3	In isolation	Correct	
9	2	Female	/pu/	2	Sadness	Tone 3	In isolation	Correct	
9	1	Male	/pu/	1	Sadness	Tone 3	In isolation	Correct	
9	1	Male	/pu/	2	Sadness	Tone 3	In isolation	Incorrect	
9	2	Female	/ci/	1	Sadness	Tone 4	In isolation	Correct	
9	2	Female	/ci/	2	Sadness	Tone 4	In isolation	Correct	
9	1	Male	/ci/	1	Sadness	Tone 4	In isolation	Incorrect	
9	1	Male	/ci/	2	Sadness	Tone 4	In isolation	Correct	
9	2	Female	/fa/	1	Sadness	Tone 4	In isolation	Correct	
9	2	Female	/fa/	2	Sadness	Tone 4	In isolation	Incorrect	
9	1	Male	/fa/	1	Sadness	Tone 4	In isolation	Incorrect	
9	1	Male	/fa/	2	Sadness	Tone 4	In isolation	Correct	
9	2	Female	/pu/	1	Sadness	Tone 4	In isolation	Correct	
9	2	Female	/pu/	2	Sadness	Tone 4	In isolation	Correct	
9	1	Male	/pu/	1	Sadness	Tone 4	In isolation	Correct	

9	1	Male	/pu/	2	Sadness	Tone 4	In isolation	Correct	
9	2	Female	/ci/	1	Anger	Tone 1	In context	Incorrect	
9	2	Female	/ci/	2	Anger	Tone 1	In context	Incorrect	
9	1	Male	/ci/	1	Anger	Tone 1	In context	Correct	
9	1	Male	/ci/	2	Anger	Tone 1	In context	Correct	
9	2	Female	/fa/	1	Anger	Tone 1	In context	Correct	
9	2	Female	/fa/	2	Anger	Tone 1	In context	Correct	
9	1	Male	/fa/	1	Anger	Tone 1	In context	Correct	
9	1	Male	/fa/	2	Anger	Tone 1	In context	Correct	
9	2	Female	/pu/	1	Anger	Tone 1	In context	Correct	
9	2	Female	/pu/	2	Anger	Tone 1	In context	Incorrect	
9	1	Male	/pu/	1	Anger	Tone 1	In context	Correct	
9	1	Male	/pu/	2	Anger	Tone 1	In context	Correct	
9	2	Female	/ci/	1	Anger	Tone 2	In context	Correct	
9	2	Female	/ci/	2	Anger	Tone 2	In context	Correct	
9	1	Male	/ci/	1	Anger	Tone 2	In context	Correct	
9	1	Male	/ci/	2	Anger	Tone 2	In context	Correct	
9	2	Female	/fa/	1	Anger	Tone 2	In context	Correct	
9	2	Female	/fa/	2	Anger	Tone 2	In context	Correct	
9	1	Male	/fa/	1	Anger	Tone 2	In context	Correct	
9	1	Male	/fa/	2	Anger	Tone 2	In context	Correct	
9	2	Female	/pu/	1	Anger	Tone 2	In context	Correct	
9	2	Female	/pu/	2	Anger	Tone 2	In context	Correct	
9	1	Male	/pu/	1	Anger	Tone 2	In context	Correct	
9	1	Male	/pu/	2	Anger	Tone 2	In context	Correct	
9	2	Female	/ci/	1	Anger	Tone 3	In context	Correct	
9	2	Female	/ci/	2	Anger	Tone 3	In context	Correct	
9	1	Male	/ci/	1	Anger	Tone 3	In context	Correct	
9	1	Male	/ci/	2	Anger	Tone 3	In context	Correct	
9	2	Female	/fa/	1	Anger	Tone 3	In context	Correct	
9	2	Female	/fa/	2	Anger	Tone 3	In context	Correct	
9	1	Male	/fa/	1	Anger	Tone 3	In context	Correct	
9	1	Male	/fa/	2	Anger	Tone 3	In context	Correct	
9	2	Female	/pu/	1	Anger	Tone 3	In context	Correct	
9	2	Female	/pu/	2	Anger	Tone 3	In context	Correct	
9	1	Male	/pu/	1	Anger	Tone 3	In context	Correct	
9	1	Male	/pu/	2	Anger	Tone 3	In context	Correct	
9	2	Female	/ci/	1	Anger	Tone 4	In context	Correct	
9	2	Female	/ci/	2	Anger	Tone 4	In context	Correct	
9	1	Male	/ci/	1	Anger	Tone 4	In context	Correct	
9	1	Male	/ci/	2	Anger	Tone 4	In context	Correct	
9	2	Female	/fa/	1	Anger	Tone 4	In context	Correct	
9	2	Female	/fa/	2	Anger	Tone 4	In context	Correct	
9	1	Male	/fa/	1	Anger	Tone 4	In context	Correct	
9	1	Male	/fa/	2	Anger	Tone 4	In context	Correct	
9	2	Female	/pu/	1	Anger	Tone 4	In context	Correct	
9	2	Female	/pu/	2	Anger	Tone 4	In context	Correct	
9	1	Male	/pu/	1	Anger	Tone 4	In context	Correct	
9	1	Male	/pu/	2	Anger	Tone 4	In context	Correct	
9	2	Female	/ci/	1	Fear	Tone 1	In context	Correct	
9	2	Female	/ci/	2	Fear	Tone 1	In context	Correct	

9	1	Male	/ci/	1	Fear	Tone 1	In context	Correct	
9	1	Male	/ci/	2	Fear	Tone 1	In context	Correct	
9	2	Female	/fa/	1	Fear	Tone 1	In context	Correct	
9	2	Female	/fa/	2	Fear	Tone 1	In context	Correct	
9	1	Male	/fa/	1	Fear	Tone 1	In context	Correct	
9	1	Male	/fa/	2	Fear	Tone 1	In context	Correct	
9	2	Female	/pu/	1	Fear	Tone 1	In context	Incorrect	
9	2	Female	/pu/	2	Fear	Tone 1	In context	Correct	
9	1	Male	/pu/	1	Fear	Tone 1	In context	Correct	
9	1	Male	/pu/	2	Fear	Tone 1	In context	Correct	
9	2	Female	/ci/	1	Fear	Tone 2	In context	Correct	
9	2	Female	/ci/	2	Fear	Tone 2	In context	Correct	
9	1	Male	/ci/	1	Fear	Tone 2	In context	Correct	
9	1	Male	/ci/	2	Fear	Tone 2	In context	Correct	
9	2	Female	/fa/	1	Fear	Tone 2	In context	Correct	
9	2	Female	/fa/	2	Fear	Tone 2	In context	Correct	
9	1	Male	/fa/	1	Fear	Tone 2	In context	Correct	
9	1	Male	/fa/	2	Fear	Tone 2	In context	Correct	
9	2	Female	/pu/	1	Fear	Tone 2	In context	Correct	
9	2	Female	/pu/	2	Fear	Tone 2	In context	Correct	
9	1	Male	/pu/	1	Fear	Tone 2	In context	Correct	
9	1	Male	/pu/	2	Fear	Tone 2	In context	Correct	
9	2	Female	/ci/	1	Fear	Tone 3	In context	Correct	
9	2	Female	/ci/	2	Fear	Tone 3	In context	Correct	
9	1	Male	/ci/	1	Fear	Tone 3	In context	Correct	
9	1	Male	/ci/	2	Fear	Tone 3	In context	Correct	
9	2	Female	/fa/	1	Fear	Tone 3	In context	Correct	
9	2	Female	/fa/	2	Fear	Tone 3	In context	Correct	
9	1	Male	/fa/	1	Fear	Tone 3	In context	Correct	
9	1	Male	/fa/	2	Fear	Tone 3	In context	Correct	
9	2	Female	/pu/	1	Fear	Tone 3	In context	Correct	
9	2	Female	/pu/	2	Fear	Tone 3	In context	Correct	
9	1	Male	/pu/	1	Fear	Tone 3	In context	Correct	
9	1	Male	/pu/	2	Fear	Tone 3	In context	Correct	
9	2	Female	/ci/	1	Fear	Tone 4	In context	Correct	
9	2	Female	/ci/	2	Fear	Tone 4	In context	Correct	
9	1	Male	/ci/	1	Fear	Tone 4	In context	Correct	
9	1	Male	/ci/	2	Fear	Tone 4	In context	Correct	
9	2	Female	/fa/	1	Fear	Tone 4	In context	Correct	
9	2	Female	/fa/	2	Fear	Tone 4	In context	Correct	
9	1	Male	/fa/	1	Fear	Tone 4	In context	Correct	
9	1	Male	/fa/	2	Fear	Tone 4	In context	Correct	
9	2	Female	/pu/	1	Fear	Tone 4	In context	Correct	
9	2	Female	/pu/	2	Fear	Tone 4	In context	Correct	
9	1	Male	/pu/	1	Fear	Tone 4	In context	Correct	
9	1	Male	/pu/	2	Fear	Tone 4	In context	Correct	
9	2	Female	/ci/	1	Happines	Tone 1	In context	Correct	
9	2	Female	/ci/	2	Happines	Tone 1	In context	Correct	
9	1	Male	/ci/	1	Happines	Tone 1	In context	Correct	
9	1	Male	/ci/	2	Happines	Tone 1	In context	Correct	
9	2	Female	/fa/	1	Happines	Tone 1	In context	Correct	

9	2	Female
9	1	Male
9	1	Male
9	2	Female
9	2	Female
9	1	Male
/fa/
/fa/
/fa/
/pu/
/pu/
/pu/
HappinesTone 1 In context
HappinesTone 1 In context
HappinesTone 1 In context
HappinesTone 1 In context
HappinesTone 1 In context
HappinesTone 1 In context
Correct Correct Correct Correct Correct Correct

9	1	Male
/pu/
HappinesTone 1 In context Incorrect

9	2	Female
9	2	Female
9	1	Male
9	1	Male
9	2	Female
9	2	Female
9	1	Male
9	1	Male
9	2	Female
9	2	Female
9	1	Male
9	1	Male
9	2	Female
9	2	Female
9	1	Male
9	1	Male
9	2	Female
9	2	Female
9	1	Male
9	1	Male
9	2	Female
9	2	Female
9	1	Male
9	1	Male
9	2	Female
9	2	Female
9	1	Male
9	1	Male
9	2	Female
9	2	Female
9	1	Male
9	1	Male
9	2	Female
9	2	Female
9	1	Male
9	1	Male
/ci/
/ci/
/ci/
/ci/
/fa/
/fa/
/fa/
/fa/
/pu/
/pu/
/pu/
/pu/
/ci/
/ci/
/ci/
/ci/
/fa/
/fa/
/fa/
/fa/
/pu/
/pu/
/pu/
/pu/
/ci/
/ci/
/ci/
/ci/
/fa/
/fa/
/fa/
/fa/
/pu/
/pu/
/pu/
/pu/
HappinesTone 2 In context
HappinesTone 2 In context
HappinesTone 2 In context
HappinesTone 2 In context
HappinesTone 2 In context
HappinesTone 2 In context
HappinesTone 2 In context
HappinesTone 2 In context
HappinesTone 2 In context
HappinesTone 2 In context
HappinesTone 2 In context
HappinesTone 2 In context
HappinesTone 3 In context
HappinesTone 3 In context
HappinesTone 3 In context
HappinesTone 3 In context
HappinesTone 3 In context
HappinesTone 3 In context
HappinesTone 3 In context
HappinesTone 3 In context
HappinesTone 3 In context
HappinesTone 3 In context
HappinesTone 3 In context
HappinesTone 3 In context
HappinesTone 4 In context
HappinesTone 4 In context
HappinesTone 4 In context
HappinesTone 4 In context
HappinesTone 4 In context
HappinesTone 4 In context
HappinesTone 4 In context
HappinesTone 4 In context
HappinesTone 4 In context
HappinesTone 4 In context
HappinesTone 4 In context
HappinesTone 4 In context
Correct Correct Correct Correct Correct Correct Correct Correct Correct Correct Correct Correct Correct Correct Correct Correct Correct Correct Correct Correct Correct Correct Correct Correct Correct Correct Correct Correct Correct Correct Correct Correct Correct Correct Correct Correct

9	2	Female
9	2	Female
9	1	Male
9	1	Male
9	2	Female
9	2	Female
9	1	Male
9	1	Male
/ci/
/ci/
/ci/
/ci/
/fa/
/fa/
/fa/
/fa/
Neutral Tone 1 In context
Neutral Tone 1 In context
Neutral Tone 1 In context
Neutral Tone 1 In context
Neutral Tone 1 In context
Neutral Tone 1 In context
Neutral Tone 1 In context
Neutral Tone 1 In context
Correct
Correct Correct Correct Correct Correct Correct Correct

9	2	Female	/pu/	1	Neutral	Tone 1	In context	Correct	
9	2	Female	/pu/	2	Neutral	Tone 1	In context	Correct	
9	1	Male	/pu/	1	Neutral	Tone 1	In context	Correct	
9	1	Male	/pu/	2	Neutral	Tone 1	In context	Correct	
9	2	Female	/ci/	1	Neutral	Tone 2	In context	Correct	
9	2	Female	/ci/	2	Neutral	Tone 2	In context	Correct	
9	1	Male	/ci/	1	Neutral	Tone 2	In context	Correct	
9	1	Male	/ci/	2	Neutral	Tone 2	In context	Correct	
9	2	Female	/fa/	1	Neutral	Tone 2	In context	Correct	
9	2	Female	/fa/	2	Neutral	Tone 2	In context	Correct	
9	1	Male	/fa/	1	Neutral	Tone 2	In context	Correct	
9	1	Male	/fa/	2	Neutral	Tone 2	In context	Correct	
9	2	Female	/pu/	1	Neutral	Tone 2	In context	Correct	
9	2	Female	/pu/	2	Neutral	Tone 2	In context	Correct	
9	1	Male	/pu/	1	Neutral	Tone 2	In context	Correct	
9	1	Male	/pu/	2	Neutral	Tone 2	In context	Correct	
9	2	Female	/ci/	1	Neutral	Tone 3	In context	Correct	
9	2	Female	/ci/	2	Neutral	Tone 3	In context	Correct	
9	1	Male	/ci/	1	Neutral	Tone 3	In context	Correct	
9	1	Male	/ci/	2	Neutral	Tone 3	In context	Correct	
9	2	Female	/fa/	1	Neutral	Tone 3	In context	Correct	
9	2	Female	/fa/	2	Neutral	Tone 3	In context	Correct	
9	1	Male	/fa/	1	Neutral	Tone 3	In context	Correct	
9	1	Male	/fa/	2	Neutral	Tone 3	In context	Correct	
9	2	Female	/pu/	1	Neutral	Tone 3	In context	Correct	
9	2	Female	/pu/	2	Neutral	Tone 3	In context	Correct	
9	1	Male	/pu/	1	Neutral	Tone 3	In context	Correct	
9	1	Male	/pu/	2	Neutral	Tone 3	In context	Correct	
9	2	Female	/ci/	1	Neutral	Tone 4	In context	Correct	
9	2	Female	/ci/	2	Neutral	Tone 4	In context	Correct	
9	1	Male	/ci/	1	Neutral	Tone 4	In context	Correct	
9	1	Male	/ci/	2	Neutral	Tone 4	In context	Correct	
9	2	Female	/fa/	1	Neutral	Tone 4	In context	Correct	
9	2	Female	/fa/	2	Neutral	Tone 4	In context	Correct	
9	1	Male	/fa/	1	Neutral	Tone 4	In context	Correct	
9	1	Male	/fa/	2	Neutral	Tone 4	In context	Correct	
9	2	Female	/pu/	1	Neutral	Tone 4	In context	Correct	
9	2	Female	/pu/	2	Neutral	Tone 4	In context	Correct	
9	1	Male	/pu/	1	Neutral	Tone 4	In context	Correct	
9	1	Male	/pu/	2	Neutral	Tone 4	In context	Correct	
9	2	Female	/ci/	1	Sadness	Tone 1	In context	Correct	
9	2	Female	/ci/	2	Sadness	Tone 1	In context	Correct	
9	1	Male	/ci/	1	Sadness	Tone 1	In context	Correct	
9	1	Male	/ci/	2	Sadness	Tone 1	In context	Correct	
9	2	Female	/fa/	1	Sadness	Tone 1	In context	Correct	
9	2	Female	/fa/	2	Sadness	Tone 1	In context	Correct	
9	1	Male	/fa/	1	Sadness	Tone 1	In context	Correct	
9	1	Male	/fa/	2	Sadness	Tone 1	In context	Correct	
9	2	Female	/pu/	1	Sadness	Tone 1	In context	Incorrect	
9	2	Female	/pu/	2	Sadness	Tone 1	In context	Correct	
9	1	Male	/pu/	1	Sadness	Tone 1	In context	Correct	

9	1	Male	/pu/	2	Sadness	Tone 1	In context	Correct	
9	2	Female	/ci/	1	Sadness	Tone 2	In context	Correct	
9	2	Female	/ci/	2	Sadness	Tone 2	In context	Correct	
9	1	Male	/ci/	1	Sadness	Tone 2	In context	Correct	
9	1	Male	/ci/	2	Sadness	Tone 2	In context	Correct	
9	2	Female	/fa/	1	Sadness	Tone 2	In context	Correct	
9	2	Female	/fa/	2	Sadness	Tone 2	In context	Correct	
9	1	Male	/fa/	1	Sadness	Tone 2	In context	Correct	
9	1	Male	/fa/	2	Sadness	Tone 2	In context	Correct	
9	2	Female	/pu/	1	Sadness	Tone 2	In context	Correct	
9	2	Female	/pu/	2	Sadness	Tone 2	In context	Correct	
9	1	Male	/pu/	1	Sadness	Tone 2	In context	Correct	
9	1	Male	/pu/	2	Sadness	Tone 2	In context	Correct	
9	2	Female	/ci/	1	Sadness	Tone 3	In context	Correct	
9	2	Female	/ci/	2	Sadness	Tone 3	In context	Incorrect	
9	1	Male	/ci/	1	Sadness	Tone 3	In context	Incorrect	
9	1	Male	/ci/	2	Sadness	Tone 3	In context	Correct	
9	2	Female	/fa/	1	Sadness	Tone 3	In context	Correct	
9	2	Female	/fa/	2	Sadness	Tone 3	In context	Correct	
9	1	Male	/fa/	1	Sadness	Tone 3	In context	Correct	
9	1	Male	/fa/	2	Sadness	Tone 3	In context	Correct	
9	2	Female	/pu/	1	Sadness	Tone 3	In context	Correct	
9	2	Female	/pu/	2	Sadness	Tone 3	In context	Correct	
9	1	Male	/pu/	1	Sadness	Tone 3	In context	Correct	
9	1	Male	/pu/	2	Sadness	Tone 3	In context	Correct	
9	2	Female	/ci/	1	Sadness	Tone 4	In context	Correct	
9	2	Female	/ci/	2	Sadness	Tone 4	In context	Correct	
9	1	Male	/ci/	1	Sadness	Tone 4	In context	Correct	
9	1	Male	/ci/	2	Sadness	Tone 4	In context	Correct	
9	2	Female	/fa/	1	Sadness	Tone 4	In context	Correct	
9	2	Female	/fa/	2	Sadness	Tone 4	In context	Correct	
9	1	Male	/fa/	1	Sadness	Tone 4	In context	Correct	
9	1	Male	/fa/	2	Sadness	Tone 4	In context	Correct	
9	2	Female	/pu/	1	Sadness	Tone 4	In context	Correct	
9	2	Female	/pu/	2	Sadness	Tone 4	In context	Correct	
9	1	Male	/pu/	1	Sadness	Tone 4	In context	Correct	
9	1	Male	/pu/	2	Sadness	Tone 4	In context	Correct	
10	2	Female	/ci/	1	Anger	Tone 1	In isolation	Correct	
10	2	Female	/ci/	2	Anger	Tone 1	In isolation	Correct	
10	1	Male	/ci/	1	Anger	Tone 1	In isolation	Correct	
10	1	Male	/ci/	2	Anger	Tone 1	In isolation	Correct	
10	2	Female	/fa/	1	Anger	Tone 1	In isolation	Incorrect	
10	2	Female	/fa/	2	Anger	Tone 1	In isolation	Correct	
10	1	Male	/fa/	1	Anger	Tone 1	In isolation	Incorrect	
10	1	Male	/fa/	2	Anger	Tone 1	In isolation	Incorrect	
10	2	Female	/pu/	1	Anger	Tone 1	In isolation	Correct	
10	2	Female	/pu/	2	Anger	Tone 1	In isolation	Correct	
10	1	Male	/pu/	1	Anger	Tone 1	In isolation	Correct	
10	1	Male	/pu/	2	Anger	Tone 1	In isolation	Correct	
10	2	Female	/ci/	1	Anger	Tone 2	In isolation	Correct	
10	2	Female	/ci/	2	Anger	Tone 2	In isolation	Correct	

10	1	Male	/ci/	1	Anger	Tone 2	In isolation	Correct	
10	1	Male	/ci/	2	Anger	Tone 2	In isolation	Incorrect	
10	2	Female	/fa/	1	Anger	Tone 2	In isolation	Correct	
10	2	Female	/fa/	2	Anger	Tone 2	In isolation	Correct	
10	1	Male	/fa/	1	Anger	Tone 2	In isolation	Incorrect	
10	1	Male	/fa/	2	Anger	Tone 2	In isolation	Correct	
10	2	Female	/pu/	1	Anger	Tone 2	In isolation	Correct	
10	2	Female	/pu/	2	Anger	Tone 2	In isolation	Correct	
10	1	Male	/pu/	1	Anger	Tone 2	In isolation	Correct	
10	1	Male	/pu/	2	Anger	Tone 2	In isolation	Correct	
10	2	Female	/ci/	1	Anger	Tone 3	In isolation	Incorrect	
10	2	Female	/ci/	2	Anger	Tone 3	In isolation	Incorrect	
10	1	Male	/ci/	1	Anger	Tone 3	In isolation	Correct	
10	1	Male	/ci/	2	Anger	Tone 3	In isolation	Incorrect	
10	2	Female	/fa/	1	Anger	Tone 3	In isolation	Correct	
10	2	Female	/fa/	2	Anger	Tone 3	In isolation	Correct	
10	1	Male	/fa/	1	Anger	Tone 3	In isolation	Correct	
10	1	Male	/fa/	2	Anger	Tone 3	In isolation	Correct	
10	2	Female	/pu/	1	Anger	Tone 3	In isolation	Correct	
10	2	Female	/pu/	2	Anger	Tone 3	In isolation	Incorrect	
10	1	Male	/pu/	1	Anger	Tone 3	In isolation	Incorrect	
10	1	Male	/pu/	2	Anger	Tone 3	In isolation	Incorrect	
10	2	Female	/ci/	1	Anger	Tone 4	In isolation	Correct	
10	2	Female	/ci/	2	Anger	Tone 4	In isolation	Correct	
10	1	Male	/ci/	1	Anger	Tone 4	In isolation	Correct	
10	1	Male	/ci/	2	Anger	Tone 4	In isolation	Incorrect	
10	2	Female	/fa/	1	Anger	Tone 4	In isolation	Correct	
10	2	Female	/fa/	2	Anger	Tone 4	In isolation	Correct	
10	1	Male	/fa/	1	Anger	Tone 4	In isolation	Correct	
10	1	Male	/fa/	2	Anger	Tone 4	In isolation	Correct	
10	2	Female	/pu/	1	Anger	Tone 4	In isolation	Correct	
10	2	Female	/pu/	2	Anger	Tone 4	In isolation	Correct	
10	1	Male	/pu/	1	Anger	Tone 4	In isolation	Incorrect	
10	1	Male	/pu/	2	Anger	Tone 4	In isolation	Correct	
10	2	Female	/ci/	1	Fear	Tone 1	In isolation	Correct	
10	2	Female	/ci/	2	Fear	Tone 1	In isolation	Correct	
10	1	Male	/ci/	1	Fear	Tone 1	In isolation	Correct	
10	1	Male	/ci/	2	Fear	Tone 1	In isolation	Correct	
10	2	Female	/fa/	1	Fear	Tone 1	In isolation	Incorrect	
10	2	Female	/fa/	2	Fear	Tone 1	In isolation	Correct	
10	1	Male	/fa/	1	Fear	Tone 1	In isolation	Incorrect	
10	1	Male	/fa/	2	Fear	Tone 1	In isolation	Correct	
10	2	Female	/pu/	1	Fear	Tone 1	In isolation	Correct	
10	2	Female	/pu/	2	Fear	Tone 1	In isolation	Incorrect	
10	1	Male	/pu/	1	Fear	Tone 1	In isolation	Correct	
10	1	Male	/pu/	2	Fear	Tone 1	In isolation	Correct	
10	2	Female	/ci/	1	Fear	Tone 2	In isolation	Incorrect	
10	2	Female	/ci/	2	Fear	Tone 2	In isolation	Incorrect	
10	1	Male	/ci/	1	Fear	Tone 2	In isolation	Correct	
10	1	Male	/ci/	2	Fear	Tone 2	In isolation	Correct	
10	2	Female	/fa/	1	Fear	Tone 2	In isolation	Correct	

10	2
10	1
10	1
10	2
10	2
10	1
10	1
10	2
10	2
10	1
10	1
10	2
10	2
10	1
10	1
10	2
10	2
10	1
10	1
10	2
10	2
10	1
10	1
10	2
10	2
10	1
10	1
10	2
10	2
10	1
10	1
Female Male Male Female Female Male Male Female Female Male Male Female Female Male Male Female Female Male Male Female Female Male Male Female Female Male Male Female Female Male Male
/fa/
/fa/
/fa/
/pu/
/pu/
/pu/
/pu/
/ci/
/ci/
/ci/
/ci/
/fa/
/fa/
/fa/
/fa/
/pu/
/pu/
/pu/
/pu/
/ci/
/ci/
/ci/
/ci/
/fa/
/fa/
/fa/
/fa/
/pu/
/pu/
/pu/
/pu/
2	Fear
Fear
Fear
Fear
Fear
Fear
Fear
Fear
Fear
Fear
Fear
Fear
Fear
Fear
Fear
Fear
Fear
Fear
Fear
Fear
Fear
Fear
Fear
Fear
Fear
Fear
Fear
Fear
Fear
Fear
Fear
Tone 2In isolation Correct Tone 2In isolation Incorrect Tone 2In isolation Incorrect Tone 2In isolation Incorrect Tone 2In isolation Correct Tone 2In isolation Correct Tone 2In isolation Correct Tone 3In isolation Incorrect Tone 3In isolation Incorrect Tone 3In isolation Correct Tone 3In isolation Correct Tone 3In isolation Correct Tone 3In isolation Correct Tone 3In isolation Correct Tone 3In isolation Correct Tone 3In isolation Correct Tone 3In isolation Correct Tone 3In isolation Correct Tone 3In isolation Incorrect Tone 4In isolation Correct Tone 4In isolation Incorrect Tone 4In isolation Incorrect Tone 4In isolation Incorrect Tone 4In isolation Correct Tone 4In isolation Correct Tone 4In isolation Correct Tone 4In isolation Incorrect Tone 4In isolation Incorrect Tone 4In isolation Correct Tone 4In isolation Incorrect Tone 4In isolation Incorrect

10	2
10	2
10	1
10	1
10	2
10	2
10	1
10	1
10	2
10	2
10	1
10	1
10	2
10	2
10	1
10	1
10	2
10	2
10	1
10	1
Female
Female Male Male Female Female Male Male Female Female Male Male Female Female Male Male Female Female Male Male
/ci/
/ci/
/ci/
/ci/
/fa/
/fa/
/fa/
/fa/
/pu/
/pu/
/pu/
/pu/
/ci/
/ci/
/ci/
/ci/
/fa/
/fa/
/fa/
/fa/
HappinesTone 1In isolation Correct
HappinesTone 1In isolation Incorrect
HappinesTone 1In isolation Correct
HappinesTone 1In isolation Incorrect
HappinesTone 1In isolation Incorrect
HappinesTone 1In isolation Incorrect
HappinesTone 1In isolation Incorrect
HappinesTone 1In isolation Incorrect
HappinesTone 1In isolation Incorrect
HappinesTone 1In isolation Incorrect
HappinesTone 1In isolation Correct
HappinesTone 1In isolation Correct
HappinesTone 2In isolation Correct
HappinesTone 2In isolation Correct
HappinesTone 2In isolation Incorrect
HappinesTone 2In isolation Correct
HappinesTone 2In isolation Correct
HappinesTone 2In isolation Correct
HappinesTone 2In isolation Correct
HappinesTone 2In isolation Correct

10	2
10	2
10	1
10	1
10	2
10	2
10	1
10	1
10	2
10	2
10	1
10	1
10	2
10	2
10	1
10	1
10	2
10	2
10	1
10	1
10	2
10	2
10	1
10	1
10	2
10	2
10	1
10	1
Female Female Male Male Female Female Male Male Female Female Male Male Female Female Male Male Female Female Male Male Female Female Male Male Female Female Male Male
/pu/
/pu/
/pu/
/pu/
/ci/
/ci/
/ci/
/ci/
/fa/
/fa/
/fa/
/fa/
/pu/
/pu/
/pu/
/pu/
/ci/
/ci/
/ci/
/ci/
/fa/
/fa/
/fa/
/fa/
/pu/
/pu/
/pu/
/pu/
HappinesTone 2In isolation Correct
HappinesTone 2In isolation Incorrect
HappinesTone 2In isolation Correct
HappinesTone 2In isolation Correct
HappinesTone 3In isolation Correct
HappinesTone 3In isolation Correct
HappinesTone 3In isolation Correct
HappinesTone 3In isolation Correct
HappinesTone 3In isolation Correct
HappinesTone 3In isolation Correct
HappinesTone 3In isolation Correct
HappinesTone 3In isolation Correct
HappinesTone 3In isolation Correct
HappinesTone 3In isolation Incorrect
HappinesTone 3In isolation Incorrect
HappinesTone 3In isolation Correct
HappinesTone 4In isolation Incorrect
HappinesTone 4In isolation Incorrect
HappinesTone 4In isolation Incorrect
HappinesTone 4In isolation Correct
HappinesTone 4In isolation Correct
HappinesTone 4In isolation Correct
HappinesTone 4In isolation Correct
HappinesTone 4In isolation Correct
HappinesTone 4In isolation Incorrect
HappinesTone 4In isolation Correct
HappinesTone 4In isolation Incorrect
HappinesTone 4In isolation Incorrect

10	2
10	2
10	1
10	1
10	2
10	2
10	1
10	1
10	2
10	2
10	1
10	1
10	2
10	2
10	1
10	1
10	2
10	2
10	1
10	1
10	2
10	2
10	1
Female
Female Male Male Female Female Male Male Female Female Male Male Female Female Male Male Female Female Male Male Female Female Male
/ci/
/ci/
/ci/
/ci/
/fa/
/fa/
/fa/
/fa/
/pu/
/pu/
/pu/
/pu/
/ci/
/ci/
/ci/
/ci/
/fa/
/fa/
/fa/
/fa/
/pu/
/pu/
/pu/
Neutral Tone 1In isolation Correct
Neutral Tone 1In isolation Correct
Neutral Tone 1In isolation Correct
Neutral Tone 1In isolation Correct
Neutral Tone 1In isolation Correct
Neutral Tone 1In isolation Correct
Neutral Tone 1In isolation Correct
Neutral Tone 1In isolation Correct
Neutral Tone 1In isolation Correct
Neutral Tone 1In isolation Correct
Neutral Tone 1In isolation Correct
Neutral Tone 1In isolation Correct
Neutral Tone 2In isolation Correct
Neutral Tone 2In isolation Correct
Neutral Tone 2In isolation Correct
Neutral Tone 2In isolation Correct
Neutral Tone 2In isolation Correct
Neutral Tone 2In isolation Correct
Neutral Tone 2In isolation Correct
Neutral Tone 2In isolation Correct
Neutral Tone 2In isolation Correct
Neutral Tone 2In isolation Correct
1	Neutral Tone 2In isolation Correct

10	1	Male	/pu/	2	Neutral	Tone 2	In isolation	Correct	
10	2	Female	/ci/	1	Neutral	Tone 3	In isolation	Correct	
10	2	Female	/ci/	2	Neutral	Tone 3	In isolation	Correct	
10	1	Male	/ci/	1	Neutral	Tone 3	In isolation	Correct	
10	1	Male	/ci/	2	Neutral	Tone 3	In isolation	Correct	
10	2	Female	/fa/	1	Neutral	Tone 3	In isolation	Correct	
10	2	Female	/fa/	2	Neutral	Tone 3	In isolation	Correct	
10	1	Male	/fa/	1	Neutral	Tone 3	In isolation	Correct	
10	1	Male	/fa/	2	Neutral	Tone 3	In isolation	Correct	
10	2	Female	/pu/	1	Neutral	Tone 3	In isolation	Correct	
10	2	Female	/pu/	2	Neutral	Tone 3	In isolation	Correct	
10	1	Male	/pu/	1	Neutral	Tone 3	In isolation	Correct	
10	1	Male	/pu/	2	Neutral	Tone 3	In isolation	Correct	
10	2	Female	/ci/	1	Neutral	Tone 4	In isolation	Correct	
10	2	Female	/ci/	2	Neutral	Tone 4	In isolation	Correct	
10	1	Male	/ci/	1	Neutral	Tone 4	In isolation	Correct	
10	1	Male	/ci/	2	Neutral	Tone 4	In isolation	Correct	
10	2	Female	/fa/	1	Neutral	Tone 4	In isolation	Correct	
10	2	Female	/fa/	2	Neutral	Tone 4	In isolation	Correct	
10	1	Male	/fa/	1	Neutral	Tone 4	In isolation	Correct	
10	1	Male	/fa/	2	Neutral	Tone 4	In isolation	Correct	
10	2	Female	/pu/	1	Neutral	Tone 4	In isolation	Correct	
10	2	Female	/pu/	2	Neutral	Tone 4	In isolation	Correct	
10	1	Male	/pu/	1	Neutral	Tone 4	In isolation	Correct	
10	1	Male	/pu/	2	Neutral	Tone 4	In isolation	Correct	
10	2	Female	/ci/	1	Sadness	Tone 1	In isolation	Correct	
10	2	Female	/ci/	2	Sadness	Tone 1	In isolation	Correct	
10	1	Male	/ci/	1	Sadness	Tone 1	In isolation	Correct	
10	1	Male	/ci/	2	Sadness	Tone 1	In isolation	Correct	
10	2	Female	/fa/	1	Sadness	Tone 1	In isolation	Incorrect	
10	2	Female	/fa/	2	Sadness	Tone 1	In isolation	Correct	
10	1	Male	/fa/	1	Sadness	Tone 1	In isolation	Incorrect	
10	1	Male	/fa/	2	Sadness	Tone 1	In isolation	Correct	
10	2	Female	/pu/	1	Sadness	Tone 1	In isolation	Correct	
10	2	Female	/pu/	2	Sadness	Tone 1	In isolation	Correct	
10	1	Male	/pu/	1	Sadness	Tone 1	In isolation	Correct	
10	1	Male	/pu/	2	Sadness	Tone 1	In isolation	Correct	
10	2	Female	/ci/	1	Sadness	Tone 2	In isolation	Correct	
10	2	Female	/ci/	2	Sadness	Tone 2	In isolation	Incorrect	
10	1	Male	/ci/	1	Sadness	Tone 2	In isolation	Incorrect	
10	1	Male	/ci/	2	Sadness	Tone 2	In isolation	Correct	
10	2	Female	/fa/	1	Sadness	Tone 2	In isolation	Incorrect	
10	2	Female	/fa/	2	Sadness	Tone 2	In isolation	Correct	
10	1	Male	/fa/	1	Sadness	Tone 2	In isolation	Incorrect	
10	1	Male	/fa/	2	Sadness	Tone 2	In isolation	Correct	
10	2	Female	/pu/	1	Sadness	Tone 2	In isolation	Correct	
10	2	Female	/pu/	2	Sadness	Tone 2	In isolation	Correct	
10	1	Male	/pu/	1	Sadness	Tone 2	In isolation	Correct	
10	1	Male	/pu/	2	Sadness	Tone 2	In isolation	Incorrect	
10	2	Female	/ci/	1	Sadness	Tone 3	In isolation	Correct	
10	2	Female	/ci/	2	Sadness	Tone 3	In isolation	Incorrect	

10	1	Male	/ci/	1	Sadness	Tone 3	In isolation	Correct	
10	1	Male	/ci/	2	Sadness	Tone 3	In isolation	Correct	
10	2	Female	/fa/	1	Sadness	Tone 3	In isolation	Correct	
10	2	Female	/fa/	2	Sadness	Tone 3	In isolation	Incorrect	
10	1	Male	/fa/	1	Sadness	Tone 3	In isolation	Correct	
10	1	Male	/fa/	2	Sadness	Tone 3	In isolation	Correct	
10	2	Female	/pu/	1	Sadness	Tone 3	In isolation	Incorrect	
10	2	Female	/pu/	2	Sadness	Tone 3	In isolation	Correct	
10	1	Male	/pu/	1	Sadness	Tone 3	In isolation	Incorrect	
10	1	Male	/pu/	2	Sadness	Tone 3	In isolation	Incorrect	
10	2	Female	/ci/	1	Sadness	Tone 4	In isolation	Incorrect	
10	2	Female	/ci/	2	Sadness	Tone 4	In isolation	Incorrect	
10	1	Male	/ci/	1	Sadness	Tone 4	In isolation	Incorrect	
10	1	Male	/ci/	2	Sadness	Tone 4	In isolation	Correct	
10	2	Female	/fa/	1	Sadness	Tone 4	In isolation	Incorrect	
10	2	Female	/fa/	2	Sadness	Tone 4	In isolation	Incorrect	
10	1	Male	/fa/	1	Sadness	Tone 4	In isolation	Incorrect	
10	1	Male	/fa/	2	Sadness	Tone 4	In isolation	Correct	
10	2	Female	/pu/	1	Sadness	Tone 4	In isolation	Incorrect	
10	2	Female	/pu/	2	Sadness	Tone 4	In isolation	Correct	
10	1	Male	/pu/	1	Sadness	Tone 4	In isolation	Incorrect	
10	1	Male	/pu/	2	Sadness	Tone 4	In isolation	Incorrect	
10	2	Female	/ci/	1	Anger	Tone 1	In context	Incorrect	
10	2	Female	/ci/	2	Anger	Tone 1	In context	Incorrect	
10	1	Male	/ci/	1	Anger	Tone 1	In context	Correct	
10	1	Male	/ci/	2	Anger	Tone 1	In context	Correct	
10	2	Female	/fa/	1	Anger	Tone 1	In context	Correct	
10	2	Female	/fa/	2	Anger	Tone 1	In context	Correct	
10	1	Male	/fa/	1	Anger	Tone 1	In context	Correct	
10	1	Male	/fa/	2	Anger	Tone 1	In context	Correct	
10	2	Female	/pu/	1	Anger	Tone 1	In context	Incorrect	
10	2	Female	/pu/	2	Anger	Tone 1	In context	Incorrect	
10	1	Male	/pu/	1	Anger	Tone 1	In context	Correct	
10	1	Male	/pu/	2	Anger	Tone 1	In context	Correct	
10	2	Female	/ci/	1	Anger	Tone 2	In context	Correct	
10	2	Female	/ci/	2	Anger	Tone 2	In context	Correct	
10	1	Male	/ci/	1	Anger	Tone 2	In context	Correct	
10	1	Male	/ci/	2	Anger	Tone 2	In context	Correct	
10	2	Female	/fa/	1	Anger	Tone 2	In context	Correct	
10	2	Female	/fa/	2	Anger	Tone 2	In context	Correct	
10	1	Male	/fa/	1	Anger	Tone 2	In context	Correct	
10	1	Male	/fa/	2	Anger	Tone 2	In context	Correct	
10	2	Female	/pu/	1	Anger	Tone 2	In context	Correct	
10	2	Female	/pu/	2	Anger	Tone 2	In context	Correct	
10	1	Male	/pu/	1	Anger	Tone 2	In context	Correct	
10	1	Male	/pu/	2	Anger	Tone 2	In context	Correct	
10	2	Female	/ci/	1	Anger	Tone 3	In context	Correct	
10	2	Female	/ci/	2	Anger	Tone 3	In context	Correct	
10	1	Male	/ci/	1	Anger	Tone 3	In context	Correct	
10	1	Male	/ci/	2	Anger	Tone 3	In context	Correct	
10	2	Female	/fa/	1	Anger	Tone 3	In context	Correct	

10	2	Female	/fa/	2	Anger	Tone 3	In context	Correct	
10	1	Male	/fa/	1	Anger	Tone 3	In context	Correct	
10	1	Male	/fa/	2	Anger	Tone 3	In context	Correct	
10	2	Female	/pu/	1	Anger	Tone 3	In context	Correct	
10	2	Female	/pu/	2	Anger	Tone 3	In context	Correct	
10	1	Male	/pu/	1	Anger	Tone 3	In context	Correct	
10	1	Male	/pu/	2	Anger	Tone 3	In context	Correct	
10	2	Female	/ci/	1	Anger	Tone 4	In context	Correct	
10	2	Female	/ci/	2	Anger	Tone 4	In context	Correct	
10	1	Male	/ci/	1	Anger	Tone 4	In context	Correct	
10	1	Male	/ci/	2	Anger	Tone 4	In context	Correct	
10	2	Female	/fa/	1	Anger	Tone 4	In context	Correct	
10	2	Female	/fa/	2	Anger	Tone 4	In context	Correct	
10	1	Male	/fa/	1	Anger	Tone 4	In context	Correct	
10	1	Male	/fa/	2	Anger	Tone 4	In context	Correct	
10	2	Female	/pu/	1	Anger	Tone 4	In context	Correct	
10	2	Female	/pu/	2	Anger	Tone 4	In context	Correct	
10	1	Male	/pu/	1	Anger	Tone 4	In context	Correct	
10	1	Male	/pu/	2	Anger	Tone 4	In context	Correct	
10	2	Female	/ci/	1	Fear	Tone 1	In context	Correct	
10	2	Female	/ci/	2	Fear	Tone 1	In context	Correct	
10	1	Male	/ci/	1	Fear	Tone 1	In context	Correct	
10	1	Male	/ci/	2	Fear	Tone 1	In context	Correct	
10	2	Female	/fa/	1	Fear	Tone 1	In context	Correct	
10	2	Female	/fa/	2	Fear	Tone 1	In context	Correct	
10	1	Male	/fa/	1	Fear	Tone 1	In context	Correct	
10	1	Male	/fa/	2	Fear	Tone 1	In context	Correct	
10	2	Female	/pu/	1	Fear	Tone 1	In context	Correct	
10	2	Female	/pu/	2	Fear	Tone 1	In context	Correct	
10	1	Male	/pu/	1	Fear	Tone 1	In context	Correct	
10	1	Male	/pu/	2	Fear	Tone 1	In context	Correct	
10	2	Female	/ci/	1	Fear	Tone 2	In context	Correct	
10	2	Female	/ci/	2	Fear	Tone 2	In context	Correct	
10	1	Male	/ci/	1	Fear	Tone 2	In context	Correct	
10	1	Male	/ci/	2	Fear	Tone 2	In context	Correct	
10	2	Female	/fa/	1	Fear	Tone 2	In context	Correct	
10	2	Female	/fa/	2	Fear	Tone 2	In context	Correct	
10	1	Male	/fa/	1	Fear	Tone 2	In context	Correct	
10	1	Male	/fa/	2	Fear	Tone 2	In context	Correct	
10	2	Female	/pu/	1	Fear	Tone 2	In context	Correct	
10	2	Female	/pu/	2	Fear	Tone 2	In context	Correct	
10	1	Male	/pu/	1	Fear	Tone 2	In context	Correct	
10	1	Male	/pu/	2	Fear	Tone 2	In context	Correct	
10	2	Female	/ci/	1	Fear	Tone 3	In context	Correct	
10	2	Female	/ci/	2	Fear	Tone 3	In context	Correct	
10	1	Male	/ci/	1	Fear	Tone 3	In context	Correct	
10	1	Male	/ci/	2	Fear	Tone 3	In context	Correct	
10	2	Female	/fa/	1	Fear	Tone 3	In context	Correct	
10	2	Female	/fa/	2	Fear	Tone 3	In context	Correct	
10	1	Male	/fa/	1	Fear	Tone 3	In context	Correct	
10	1	Male	/fa/	2	Fear	Tone 3	In context	Correct	

10	2
10	2
10	1
10	1
10	2
10	2
10	1
10	1
10	2
10	2
10	1
10	1
10	2
10	2
10	1
10	1
Female Female Male Male Female Female Male Male Female Female Male Male Female Female Male Male
/pu/
/pu/
/pu/
/pu/
/ci/
/ci/
/ci/
/ci/
/fa/
/fa/
/fa/
/fa/
/pu/
/pu/
/pu/
/pu/
Fear
Fear
Fear
Fear
Fear
Fear
Fear
Fear
Fear
Fear
Fear
Fear
Fear
Fear
Fear
Fear
Tone 3 In context Tone 3 In context Tone 3 In context Tone 3 In context Tone 4 In context Tone 4 In context Tone 4 In context Tone 4 In context Tone 4 In context Tone 4 In context Tone 4 In context Tone 4 In context Tone 4 In context Tone 4 In context Tone 4 In context Tone 4 In context
Correct Correct Correct Correct Correct Correct Correct Correct Correct Correct Correct Correct Correct Correct Correct Correct

10	2
10	2
10	1
10	1
10	2
10	2
10	1
10	1
10	2
10	2
10	1
Female Female Male Male Female Female Male Male Female Female Male
/ci/
/ci/
/ci/
/ci/
/fa/
/fa/
/fa/
/fa/
/pu/
/pu/
/pu/
HappinesTone 1 In context
HappinesTone 1 In context
HappinesTone 1 In context
HappinesTone 1 In context
HappinesTone 1 In context
HappinesTone 1 In context
HappinesTone 1 In context
HappinesTone 1 In context
HappinesTone 1 In context
HappinesTone 1 In context
HappinesTone 1 In context
Correct Correct Correct Correct Correct Correct Correct Correct Correct Correct Correct

10	1
Male
/pu/
HappinesTone 1 In context Incorrect

10	2
10	2
10	1
10	1
10	2
10	2
10	1
10	1
10	2
10	2
10	1
10	1
10	2
10	2
10	1
10	1
10	2
10	2
10	1
10	1
10	2
10	2
10	1
Female Female Male Male Female Female Male Male Female Female Male Male Female Female Male Male Female Female Male Male Female Female Male
/ci/
/ci/
/ci/
/ci/
/fa/
/fa/
/fa/
/fa/
/pu/
/pu/
/pu/
/pu/
/ci/
/ci/
/ci/
/ci/
/fa/
/fa/
/fa/
/fa/
/pu/
/pu/
/pu/
HappinesTone 2 In context
HappinesTone 2 In context
HappinesTone 2 In context
HappinesTone 2 In context
HappinesTone 2 In context
HappinesTone 2 In context
HappinesTone 2 In context
HappinesTone 2 In context
HappinesTone 2 In context
HappinesTone 2 In context
HappinesTone 2 In context
HappinesTone 2 In context
HappinesTone 3 In context
HappinesTone 3 In context
HappinesTone 3 In context
HappinesTone 3 In context
HappinesTone 3 In context
HappinesTone 3 In context
HappinesTone 3 In context
HappinesTone 3 In context
HappinesTone 3 In context
HappinesTone 3 In context
HappinesTone 3 In context
Correct Correct Correct Correct Correct Correct Correct Correct Correct Correct Correct Correct Correct Correct Correct Correct Correct Correct Correct Correct Correct Correct Correct

10	1
10	2
10	2
10	1
10	1
10	2
10	2
10	1
10	1
10	2
10	2
10	1
10	1
Male Female Female Male Male Female Female Male Male Female Female Male Male
/pu/
/ci/
/ci/
/ci/
/ci/
/fa/
/fa/
/fa/
/fa/
/pu/
/pu/
/pu/
/pu/
HappinesTone 3 In context
HappinesTone 4 In context
HappinesTone 4 In context
HappinesTone 4 In context
HappinesTone 4 In context
HappinesTone 4 In context
HappinesTone 4 In context
HappinesTone 4 In context
HappinesTone 4 In context
HappinesTone 4 In context
HappinesTone 4 In context
HappinesTone 4 In context
HappinesTone 4 In context
Correct Correct Correct Correct Correct Correct Correct Correct Correct Correct Correct Correct Correct

10	2
10	2
10	1
10	1
10	2
10	2
10	1
10	1
10	2
10	2
10	1
10	1
10	2
10	2
10	1
10	1
10	2
10	2
10	1
10	1
10	2
10	2
10	1
10	1
10	2
10	2
10	1
10	1
10	2
10	2
10	1
10	1
10	2
10	2
10	1
10	1
10	2
10	2
Female Female Male Male Female Female Male Male Female Female Male Male Female Female Male Male Female Female Male Male Female Female Male Male Female Female Male Male Female Female Male Male Female Female Male Male Female Female
/ci/
/ci/
/ci/
/ci/
/fa/
/fa/
/fa/
/fa/
/pu/
/pu/
/pu/
/pu/
/ci/
/ci/
/ci/
/ci/
/fa/
/fa/
/fa/
/fa/
/pu/
/pu/
/pu/
/pu/
/ci/
/ci/
/ci/
/ci/
/fa/
/fa/
/fa/
/fa/
/pu/
/pu/
/pu/
/pu/
/ci/
/ci/
Neutral Tone 1 In context
Neutral Tone 1 In context
Neutral Tone 1 In context
Neutral Tone 1 In context
Neutral Tone 1 In context
Neutral Tone 1 In context
Neutral Tone 1 In context
Neutral Tone 1 In context
Neutral Tone 1 In context
Neutral Tone 1 In context
Neutral Tone 1 In context
Neutral Tone 1 In context
Neutral Tone 2 In context
Neutral Tone 2 In context
Neutral Tone 2 In context
Neutral Tone 2 In context
Neutral Tone 2 In context
Neutral Tone 2 In context
Neutral Tone 2 In context
Neutral Tone 2 In context
Neutral Tone 2 In context
Neutral Tone 2 In context
Neutral Tone 2 In context
Neutral Tone 2 In context
Neutral Tone 3 In context
Neutral Tone 3 In context
Neutral Tone 3 In context
Neutral Tone 3 In context
Neutral Tone 3 In context
Neutral Tone 3 In context
Neutral Tone 3 In context
Neutral Tone 3 In context
Neutral Tone 3 In context
Neutral Tone 3 In context
Neutral Tone 3 In context
Neutral Tone 3 In context
Neutral Tone 4 In context
Neutral Tone 4 In context
Correct Correct Correct Correct Correct Correct Correct Correct Correct Correct Correct Correct Correct Correct Correct Correct Correct Correct Correct Correct Correct Correct Correct Correct Correct Correct Correct Correct Correct Correct Correct Correct Correct Correct Correct Correct Correct Correct

10	1	Male	/ci/	1	Neutral	Tone 4	In context	Correct	
10	1	Male	/ci/	2	Neutral	Tone 4	In context	Correct	
10	2	Female	/fa/	1	Neutral	Tone 4	In context	Correct	
10	2	Female	/fa/	2	Neutral	Tone 4	In context	Correct	
10	1	Male	/fa/	1	Neutral	Tone 4	In context	Correct	
10	1	Male	/fa/	2	Neutral	Tone 4	In context	Correct	
10	2	Female	/pu/	1	Neutral	Tone 4	In context	Correct	
10	2	Female	/pu/	2	Neutral	Tone 4	In context	Correct	
10	1	Male	/pu/	1	Neutral	Tone 4	In context	Correct	
10	1	Male	/pu/	2	Neutral	Tone 4	In context	Correct	
10	2	Female	/ci/	1	Sadness	Tone 1	In context	Correct	
10	2	Female	/ci/	2	Sadness	Tone 1	In context	Correct	
10	1	Male	/ci/	1	Sadness	Tone 1	In context	Correct	
10	1	Male	/ci/	2	Sadness	Tone 1	In context	Correct	
10	2	Female	/fa/	1	Sadness	Tone 1	In context	Correct	
10	2	Female	/fa/	2	Sadness	Tone 1	In context	Correct	
10	1	Male	/fa/	1	Sadness	Tone 1	In context	Correct	
10	1	Male	/fa/	2	Sadness	Tone 1	In context	Correct	
10	2	Female	/pu/	1	Sadness	Tone 1	In context	Incorrect	
10	2	Female	/pu/	2	Sadness	Tone 1	In context	Correct	
10	1	Male	/pu/	1	Sadness	Tone 1	In context	Correct	
10	1	Male	/pu/	2	Sadness	Tone 1	In context	Correct	
10	2	Female	/ci/	1	Sadness	Tone 2	In context	Correct	
10	2	Female	/ci/	2	Sadness	Tone 2	In context	Incorrect	
10	1	Male	/ci/	1	Sadness	Tone 2	In context	Correct	
10	1	Male	/ci/	2	Sadness	Tone 2	In context	Correct	
10	2	Female	/fa/	1	Sadness	Tone 2	In context	Correct	
10	2	Female	/fa/	2	Sadness	Tone 2	In context	Correct	
10	1	Male	/fa/	1	Sadness	Tone 2	In context	Correct	
10	1	Male	/fa/	2	Sadness	Tone 2	In context	Correct	
10	2	Female	/pu/	1	Sadness	Tone 2	In context	Correct	
10	2	Female	/pu/	2	Sadness	Tone 2	In context	Correct	
10	1	Male	/pu/	1	Sadness	Tone 2	In context	Correct	
10	1	Male	/pu/	2	Sadness	Tone 2	In context	Correct	
10	2	Female	/ci/	1	Sadness	Tone 3	In context	Correct	
10	2	Female	/ci/	2	Sadness	Tone 3	In context	Correct	
10	1	Male	/ci/	1	Sadness	Tone 3	In context	Correct	
10	1	Male	/ci/	2	Sadness	Tone 3	In context	Correct	
10	2	Female	/fa/	1	Sadness	Tone 3	In context	Correct	
10	2	Female	/fa/	2	Sadness	Tone 3	In context	Correct	
10	1	Male	/fa/	1	Sadness	Tone 3	In context	Correct	
10	1	Male	/fa/	2	Sadness	Tone 3	In context	Correct	
10	2	Female	/pu/	1	Sadness	Tone 3	In context	Correct	
10	2	Female	/pu/	2	Sadness	Tone 3	In context	Correct	
10	1	Male	/pu/	1	Sadness	Tone 3	In context	Correct	
10	1	Male	/pu/	2	Sadness	Tone 3	In context	Correct	
10	2	Female	/ci/	1	Sadness	Tone 4	In context	Correct	
10	2	Female	/ci/	2	Sadness	Tone 4	In context	Correct	
10	1	Male	/ci/	1	Sadness	Tone 4	In context	Correct	
10	1	Male	/ci/	2	Sadness	Tone 4	In context	Correct	
10	2	Female	/fa/	1	Sadness	Tone 4	In context	Correct	

10	2	Female	/fa/	2	Sadness	Tone 4	In context	Correct	
10	1	Male	/fa/	1	Sadness	Tone 4	In context	Correct	
10	1	Male	/fa/	2	Sadness	Tone 4	In context	Correct	
10	2	Female	/pu/	1	Sadness	Tone 4	In context	Correct	
10	2	Female	/pu/	2	Sadness	Tone 4	In context	Correct	
10	1	Male	/pu/	1	Sadness	Tone 4	In context	Correct	
10	1	Male	/pu/	2	Sadness	Tone 4	In context	Correct	
11	2	Female	/ci/	1	Anger	Tone 1	In isolation	Correct	
11	2	Female	/ci/	2	Anger	Tone 1	In isolation	Correct	
11	1	Male	/ci/	1	Anger	Tone 1	In isolation	Correct	
11	1	Male	/ci/	2	Anger	Tone 1	In isolation	Correct	
11	2	Female	/fa/	1	Anger	Tone 1	In isolation	Correct	
11	2	Female	/fa/	2	Anger	Tone 1	In isolation	Correct	
11	1	Male	/fa/	1	Anger	Tone 1	In isolation	Correct	
11	1	Male	/fa/	2	Anger	Tone 1	In isolation	Correct	
11	2	Female	/pu/	1	Anger	Tone 1	In isolation	Incorrect	
11	2	Female	/pu/	2	Anger	Tone 1	In isolation	Correct	
11	1	Male	/pu/	1	Anger	Tone 1	In isolation	Correct	
11	1	Male	/pu/	2	Anger	Tone 1	In isolation	Correct	
11	2	Female	/ci/	1	Anger	Tone 2	In isolation	Incorrect	
11	2	Female	/ci/	2	Anger	Tone 2	In isolation	Incorrect	
11	1	Male	/ci/	1	Anger	Tone 2	In isolation	Correct	
11	1	Male	/ci/	2	Anger	Tone 2	In isolation	Correct	
11	2	Female	/fa/	1	Anger	Tone 2	In isolation	Correct	
11	2	Female	/fa/	2	Anger	Tone 2	In isolation	Correct	
11	1	Male	/fa/	1	Anger	Tone 2	In isolation	Correct	
11	1	Male	/fa/	2	Anger	Tone 2	In isolation	Correct	
11	2	Female	/pu/	1	Anger	Tone 2	In isolation	Correct	
11	2	Female	/pu/	2	Anger	Tone 2	In isolation	Correct	
11	1	Male	/pu/	1	Anger	Tone 2	In isolation	Correct	
11	1	Male	/pu/	2	Anger	Tone 2	In isolation	Correct	
11	2	Female	/ci/	1	Anger	Tone 3	In isolation	Incorrect	
11	2	Female	/ci/	2	Anger	Tone 3	In isolation	Incorrect	
11	1	Male	/ci/	1	Anger	Tone 3	In isolation	Incorrect	
11	1	Male	/ci/	2	Anger	Tone 3	In isolation	Incorrect	
11	2	Female	/fa/	1	Anger	Tone 3	In isolation	Incorrect	
11	2	Female	/fa/	2	Anger	Tone 3	In isolation	Incorrect	
11	1	Male	/fa/	1	Anger	Tone 3	In isolation	Correct	
11	1	Male	/fa/	2	Anger	Tone 3	In isolation	Incorrect	
11	2	Female	/pu/	1	Anger	Tone 3	In isolation	Incorrect	
11	2	Female	/pu/	2	Anger	Tone 3	In isolation	Incorrect	
11	1	Male	/pu/	1	Anger	Tone 3	In isolation	Incorrect	
11	1	Male	/pu/	2	Anger	Tone 3	In isolation	Incorrect	
11	2	Female	/ci/	1	Anger	Tone 4	In isolation	Correct	
11	2	Female	/ci/	2	Anger	Tone 4	In isolation	Correct	
11	1	Male	/ci/	1	Anger	Tone 4	In isolation	Correct	
11	1	Male	/ci/	2	Anger	Tone 4	In isolation	Correct	
11	2	Female	/fa/	1	Anger	Tone 4	In isolation	Correct	
11	2	Female	/fa/	2	Anger	Tone 4	In isolation	Correct	
11	1	Male	/fa/	1	Anger	Tone 4	In isolation	Correct	
11	1	Male	/fa/	2	Anger	Tone 4	In isolation	Correct	

11	2	Female	/pu/	1	Anger	Tone 4	In isolation	Incorrect	
11	2	Female	/pu/	2	Anger	Tone 4	In isolation	Correct	
11	1	Male	/pu/	1	Anger	Tone 4	In isolation	Correct	
11	1	Male	/pu/	2	Anger	Tone 4	In isolation	Correct	
11	2	Female	/ci/	1	Fear	Tone 1	In isolation	Correct	
11	2	Female	/ci/	2	Fear	Tone 1	In isolation	Correct	
11	1	Male	/ci/	1	Fear	Tone 1	In isolation	Correct	
11	1	Male	/ci/	2	Fear	Tone 1	In isolation	Correct	
11	2	Female	/fa/	1	Fear	Tone 1	In isolation	Correct	
11	2	Female	/fa/	2	Fear	Tone 1	In isolation	Correct	
11	1	Male	/fa/	1	Fear	Tone 1	In isolation	Correct	
11	1	Male	/fa/	2	Fear	Tone 1	In isolation	Correct	
11	2	Female	/pu/	1	Fear	Tone 1	In isolation	Incorrect	
11	2	Female	/pu/	2	Fear	Tone 1	In isolation	Correct	
11	1	Male	/pu/	1	Fear	Tone 1	In isolation	Correct	
11	1	Male	/pu/	2	Fear	Tone 1	In isolation	Correct	
11	2	Female	/ci/	1	Fear	Tone 2	In isolation	Incorrect	
11	2	Female	/ci/	2	Fear	Tone 2	In isolation	Incorrect	
11	1	Male	/ci/	1	Fear	Tone 2	In isolation	Correct	
11	1	Male	/ci/	2	Fear	Tone 2	In isolation	Correct	
11	2	Female	/fa/	1	Fear	Tone 2	In isolation	Correct	
11	2	Female	/fa/	2	Fear	Tone 2	In isolation	Correct	
11	1	Male	/fa/	1	Fear	Tone 2	In isolation	Correct	
11	1	Male	/fa/	2	Fear	Tone 2	In isolation	Correct	
11	2	Female	/pu/	1	Fear	Tone 2	In isolation	Incorrect	
11	2	Female	/pu/	2	Fear	Tone 2	In isolation	Incorrect	
11	1	Male	/pu/	1	Fear	Tone 2	In isolation	Correct	
11	1	Male	/pu/	2	Fear	Tone 2	In isolation	Correct	
11	2	Female	/ci/	1	Fear	Tone 3	In isolation	Incorrect	
11	2	Female	/ci/	2	Fear	Tone 3	In isolation	Incorrect	
11	1	Male	/ci/	1	Fear	Tone 3	In isolation	Incorrect	
11	1	Male	/ci/	2	Fear	Tone 3	In isolation	Incorrect	
11	2	Female	/fa/	1	Fear	Tone 3	In isolation	Incorrect	
11	2	Female	/fa/	2	Fear	Tone 3	In isolation	Incorrect	
11	1	Male	/fa/	1	Fear	Tone 3	In isolation	Incorrect	
11	1	Male	/fa/	2	Fear	Tone 3	In isolation	Incorrect	
11	2	Female	/pu/	1	Fear	Tone 3	In isolation	Incorrect	
11	2	Female	/pu/	2	Fear	Tone 3	In isolation	Incorrect	
11	1	Male	/pu/	1	Fear	Tone 3	In isolation	Incorrect	
11	1	Male	/pu/	2	Fear	Tone 3	In isolation	Incorrect	
11	2	Female	/ci/	1	Fear	Tone 4	In isolation	Incorrect	
11	2	Female	/ci/	2	Fear	Tone 4	In isolation	Incorrect	
11	1	Male	/ci/	1	Fear	Tone 4	In isolation	Correct	
11	1	Male	/ci/	2	Fear	Tone 4	In isolation	Correct	
11	2	Female	/fa/	1	Fear	Tone 4	In isolation	Correct	
11	2	Female	/fa/	2	Fear	Tone 4	In isolation	Correct	
11	1	Male	/fa/	1	Fear	Tone 4	In isolation	Correct	
11	1	Male	/fa/	2	Fear	Tone 4	In isolation	Correct	
11	2	Female	/pu/	1	Fear	Tone 4	In isolation	Correct	
11	2	Female	/pu/	2	Fear	Tone 4	In isolation	Correct	
11	1	Male	/pu/	1	Fear	Tone 4	In isolation	Correct	

11	1
Male
/pu/
2	Fear
Tone 4In isolation Correct

11	2
11	2
11	1
11	1
11	2
11	2
11	1
11	1
11	2
11	2
11	1
11	1
11	2
11	2
11	1
11	1
11	2
11	2
11	1
11	1
11	2
11	2
11	1
11	1
11	2
11	2
11	1
11	1
11	2
11	2
11	1
11	1
11	2
11	2
11	1
11	1
11	2
11	2
11	1
11	1
11	2
11	2
11	1
11	1
11	2
11	2
11	1
11	1
Female Female Male Male Female Female Male Male Female Female Male Male Female Female Male Male Female Female Male Male Female Female Male Male Female Female Male Male Female Female Male Male Female Female Male Male Female Female Male Male Female Female Male Male Female Female Male Male
/ci/
/ci/
/ci/
/ci/
/fa/
/fa/
/fa/
/fa/
/pu/
/pu/
/pu/
/pu/
/ci/
/ci/
/ci/
/ci/
/fa/
/fa/
/fa/
/fa/
/pu/
/pu/
/pu/
/pu/
/ci/
/ci/
/ci/
/ci/
/fa/
/fa/
/fa/
/fa/
/pu/
/pu/
/pu/
/pu/
/ci/
/ci/
/ci/
/ci/
/fa/
/fa/
/fa/
/fa/
/pu/
/pu/
/pu/
/pu/
HappinesTone 1In isolation Correct
HappinesTone 1In isolation Incorrect
HappinesTone 1In isolation Correct
HappinesTone 1In isolation Correct
HappinesTone 1In isolation Incorrect
HappinesTone 1In isolation Incorrect
HappinesTone 1In isolation Correct
HappinesTone 1In isolation Correct
HappinesTone 1In isolation Incorrect
HappinesTone 1In isolation Incorrect
HappinesTone 1In isolation Correct
HappinesTone 1In isolation Correct
HappinesTone 2In isolation Correct
HappinesTone 2In isolation Correct
HappinesTone 2In isolation Correct
HappinesTone 2In isolation Correct
HappinesTone 2In isolation Correct
HappinesTone 2In isolation Correct
HappinesTone 2In isolation Correct
HappinesTone 2In isolation Correct
HappinesTone 2In isolation Correct
HappinesTone 2In isolation Correct
HappinesTone 2In isolation Correct
HappinesTone 2In isolation Correct
HappinesTone 3In isolation Incorrect
HappinesTone 3In isolation Incorrect
HappinesTone 3In isolation Incorrect
HappinesTone 3In isolation Correct
HappinesTone 3In isolation Incorrect
HappinesTone 3In isolation Incorrect
HappinesTone 3In isolation Correct
HappinesTone 3In isolation Correct
HappinesTone 3In isolation Incorrect
HappinesTone 3In isolation Correct
HappinesTone 3In isolation Incorrect
HappinesTone 3In isolation Incorrect
HappinesTone 4In isolation Correct
HappinesTone 4In isolation Incorrect
HappinesTone 4In isolation Correct
HappinesTone 4In isolation Correct
HappinesTone 4In isolation Incorrect
HappinesTone 4In isolation Correct
HappinesTone 4In isolation Correct
HappinesTone 4In isolation Correct
HappinesTone 4In isolation Incorrect
HappinesTone 4In isolation Incorrect
HappinesTone 4In isolation Correct
HappinesTone 4In isolation Correct

11	2
11	2
Female
Female
/ci/
/ci/
Neutral Tone 1In isolation Correct
Neutral Tone 1In isolation Correct

11	1	Male	/ci/	1	Neutral	Tone 1	In isolation	Correct	
11	1	Male	/ci/	2	Neutral	Tone 1	In isolation	Correct	
11	2	Female	/fa/	1	Neutral	Tone 1	In isolation	Correct	
11	2	Female	/fa/	2	Neutral	Tone 1	In isolation	Correct	
11	1	Male	/fa/	1	Neutral	Tone 1	In isolation	Correct	
11	1	Male	/fa/	2	Neutral	Tone 1	In isolation	Correct	
11	2	Female	/pu/	1	Neutral	Tone 1	In isolation	Correct	
11	2	Female	/pu/	2	Neutral	Tone 1	In isolation	Correct	
11	1	Male	/pu/	1	Neutral	Tone 1	In isolation	Correct	
11	1	Male	/pu/	2	Neutral	Tone 1	In isolation	Correct	
11	2	Female	/ci/	1	Neutral	Tone 2	In isolation	Correct	
11	2	Female	/ci/	2	Neutral	Tone 2	In isolation	Correct	
11	1	Male	/ci/	1	Neutral	Tone 2	In isolation	Correct	
11	1	Male	/ci/	2	Neutral	Tone 2	In isolation	Incorrect	
11	2	Female	/fa/	1	Neutral	Tone 2	In isolation	Correct	
11	2	Female	/fa/	2	Neutral	Tone 2	In isolation	Correct	
11	1	Male	/fa/	1	Neutral	Tone 2	In isolation	Correct	
11	1	Male	/fa/	2	Neutral	Tone 2	In isolation	Correct	
11	2	Female	/pu/	1	Neutral	Tone 2	In isolation	Correct	
11	2	Female	/pu/	2	Neutral	Tone 2	In isolation	Correct	
11	1	Male	/pu/	1	Neutral	Tone 2	In isolation	Correct	
11	1	Male	/pu/	2	Neutral	Tone 2	In isolation	Correct	
11	2	Female	/ci/	1	Neutral	Tone 3	In isolation	Correct	
11	2	Female	/ci/	2	Neutral	Tone 3	In isolation	Correct	
11	1	Male	/ci/	1	Neutral	Tone 3	In isolation	Correct	
11	1	Male	/ci/	2	Neutral	Tone 3	In isolation	Correct	
11	2	Female	/fa/	1	Neutral	Tone 3	In isolation	Correct	
11	2	Female	/fa/	2	Neutral	Tone 3	In isolation	Correct	
11	1	Male	/fa/	1	Neutral	Tone 3	In isolation	Correct	
11	1	Male	/fa/	2	Neutral	Tone 3	In isolation	Correct	
11	2	Female	/pu/	1	Neutral	Tone 3	In isolation	Correct	
11	2	Female	/pu/	2	Neutral	Tone 3	In isolation	Correct	
11	1	Male	/pu/	1	Neutral	Tone 3	In isolation	Correct	
11	1	Male	/pu/	2	Neutral	Tone 3	In isolation	Correct	
11	2	Female	/ci/	1	Neutral	Tone 4	In isolation	Correct	
11	2	Female	/ci/	2	Neutral	Tone 4	In isolation	Correct	
11	1	Male	/ci/	1	Neutral	Tone 4	In isolation	Correct	
11	1	Male	/ci/	2	Neutral	Tone 4	In isolation	Correct	
11	2	Female	/fa/	1	Neutral	Tone 4	In isolation	Correct	
11	2	Female	/fa/	2	Neutral	Tone 4	In isolation	Correct	
11	1	Male	/fa/	1	Neutral	Tone 4	In isolation	Correct	
11	1	Male	/fa/	2	Neutral	Tone 4	In isolation	Correct	
11	2	Female	/pu/	1	Neutral	Tone 4	In isolation	Correct	
11	2	Female	/pu/	2	Neutral	Tone 4	In isolation	Correct	
11	1	Male	/pu/	1	Neutral	Tone 4	In isolation	Correct	
11	1	Male	/pu/	2	Neutral	Tone 4	In isolation	Correct	
11	2	Female	/ci/	1	Sadness	Tone 1	In isolation	Correct	
11	2	Female	/ci/	2	Sadness	Tone 1	In isolation	Correct	
11	1	Male	/ci/	1	Sadness	Tone 1	In isolation	Correct	
11	1	Male	/ci/	2	Sadness	Tone 1	In isolation	Correct	
11	2	Female	/fa/	1	Sadness	Tone 1	In isolation	Correct	

11	2	Female	/fa/	2	Sadness	Tone 1	In isolation	Correct	
11	1	Male	/fa/	1	Sadness	Tone 1	In isolation	Correct	
11	1	Male	/fa/	2	Sadness	Tone 1	In isolation	Correct	
11	2	Female	/pu/	1	Sadness	Tone 1	In isolation	Correct	
11	2	Female	/pu/	2	Sadness	Tone 1	In isolation	Correct	
11	1	Male	/pu/	1	Sadness	Tone 1	In isolation	Correct	
11	1	Male	/pu/	2	Sadness	Tone 1	In isolation	Correct	
11	2	Female	/ci/	1	Sadness	Tone 2	In isolation	Incorrect	
11	2	Female	/ci/	2	Sadness	Tone 2	In isolation	Incorrect	
11	1	Male	/ci/	1	Sadness	Tone 2	In isolation	Correct	
11	1	Male	/ci/	2	Sadness	Tone 2	In isolation	Correct	
11	2	Female	/fa/	1	Sadness	Tone 2	In isolation	Correct	
11	2	Female	/fa/	2	Sadness	Tone 2	In isolation	Correct	
11	1	Male	/fa/	1	Sadness	Tone 2	In isolation	Correct	
11	1	Male	/fa/	2	Sadness	Tone 2	In isolation	Correct	
11	2	Female	/pu/	1	Sadness	Tone 2	In isolation	Incorrect	
11	2	Female	/pu/	2	Sadness	Tone 2	In isolation	Correct	
11	1	Male	/pu/	1	Sadness	Tone 2	In isolation	Correct	
11	1	Male	/pu/	2	Sadness	Tone 2	In isolation	Correct	
11	2	Female	/ci/	1	Sadness	Tone 3	In isolation	Incorrect	
11	2	Female	/ci/	2	Sadness	Tone 3	In isolation	Incorrect	
11	1	Male	/ci/	1	Sadness	Tone 3	In isolation	Incorrect	
11	1	Male	/ci/	2	Sadness	Tone 3	In isolation	Incorrect	
11	2	Female	/fa/	1	Sadness	Tone 3	In isolation	Incorrect	
11	2	Female	/fa/	2	Sadness	Tone 3	In isolation	Incorrect	
11	1	Male	/fa/	1	Sadness	Tone 3	In isolation	Incorrect	
11	1	Male	/fa/	2	Sadness	Tone 3	In isolation	Incorrect	
11	2	Female	/pu/	1	Sadness	Tone 3	In isolation	Incorrect	
11	2	Female	/pu/	2	Sadness	Tone 3	In isolation	Incorrect	
11	1	Male	/pu/	1	Sadness	Tone 3	In isolation	Incorrect	
11	1	Male	/pu/	2	Sadness	Tone 3	In isolation	Incorrect	
11	2	Female	/ci/	1	Sadness	Tone 4	In isolation	Correct	
11	2	Female	/ci/	2	Sadness	Tone 4	In isolation	Correct	
11	1	Male	/ci/	1	Sadness	Tone 4	In isolation	Correct	
11	1	Male	/ci/	2	Sadness	Tone 4	In isolation	Correct	
11	2	Female	/fa/	1	Sadness	Tone 4	In isolation	Incorrect	
11	2	Female	/fa/	2	Sadness	Tone 4	In isolation	Incorrect	
11	1	Male	/fa/	1	Sadness	Tone 4	In isolation	Incorrect	
11	1	Male	/fa/	2	Sadness	Tone 4	In isolation	Correct	
11	2	Female	/pu/	1	Sadness	Tone 4	In isolation	Correct	
11	2	Female	/pu/	2	Sadness	Tone 4	In isolation	Correct	
11	1	Male	/pu/	1	Sadness	Tone 4	In isolation	Correct	
11	1	Male	/pu/	2	Sadness	Tone 4	In isolation	Incorrect	
11	2	Female	/ci/	1	Anger	Tone 1	In context	Correct	
11	2	Female	/ci/	2	Anger	Tone 1	In context	Incorrect	
11	1	Male	/ci/	1	Anger	Tone 1	In context	Correct	
11	1	Male	/ci/	2	Anger	Tone 1	In context	Correct	
11	2	Female	/fa/	1	Anger	Tone 1	In context	Correct	
11	2	Female	/fa/	2	Anger	Tone 1	In context	Correct	
11	1	Male	/fa/	1	Anger	Tone 1	In context	Correct	
11	1	Male	/fa/	2	Anger	Tone 1	In context	Correct	

11	2	Female	/pu/	1	Anger	Tone 1	In context	Incorrect	
11	2	Female	/pu/	2	Anger	Tone 1	In context	Incorrect	
11	1	Male	/pu/	1	Anger	Tone 1	In context	Incorrect	
11	1	Male	/pu/	2	Anger	Tone 1	In context	Correct	
11	2	Female	/ci/	1	Anger	Tone 2	In context	Correct	
11	2	Female	/ci/	2	Anger	Tone 2	In context	Correct	
11	1	Male	/ci/	1	Anger	Tone 2	In context	Correct	
11	1	Male	/ci/	2	Anger	Tone 2	In context	Correct	
11	2	Female	/fa/	1	Anger	Tone 2	In context	Correct	
11	2	Female	/fa/	2	Anger	Tone 2	In context	Correct	
11	1	Male	/fa/	1	Anger	Tone 2	In context	Correct	
11	1	Male	/fa/	2	Anger	Tone 2	In context	Correct	
11	2	Female	/pu/	1	Anger	Tone 2	In context	Correct	
11	2	Female	/pu/	2	Anger	Tone 2	In context	Correct	
11	1	Male	/pu/	1	Anger	Tone 2	In context	Correct	
11	1	Male	/pu/	2	Anger	Tone 2	In context	Correct	
11	2	Female	/ci/	1	Anger	Tone 3	In context	Correct	
11	2	Female	/ci/	2	Anger	Tone 3	In context	Correct	
11	1	Male	/ci/	1	Anger	Tone 3	In context	Correct	
11	1	Male	/ci/	2	Anger	Tone 3	In context	Correct	
11	2	Female	/fa/	1	Anger	Tone 3	In context	Correct	
11	2	Female	/fa/	2	Anger	Tone 3	In context	Correct	
11	1	Male	/fa/	1	Anger	Tone 3	In context	Correct	
11	1	Male	/fa/	2	Anger	Tone 3	In context	Correct	
11	2	Female	/pu/	1	Anger	Tone 3	In context	Correct	
11	2	Female	/pu/	2	Anger	Tone 3	In context	Correct	
11	1	Male	/pu/	1	Anger	Tone 3	In context	Correct	
11	1	Male	/pu/	2	Anger	Tone 3	In context	Correct	
11	2	Female	/ci/	1	Anger	Tone 4	In context	Correct	
11	2	Female	/ci/	2	Anger	Tone 4	In context	Correct	
11	1	Male	/ci/	1	Anger	Tone 4	In context	Correct	
11	1	Male	/ci/	2	Anger	Tone 4	In context	Correct	
11	2	Female	/fa/	1	Anger	Tone 4	In context	Correct	
11	2	Female	/fa/	2	Anger	Tone 4	In context	Correct	
11	1	Male	/fa/	1	Anger	Tone 4	In context	Correct	
11	1	Male	/fa/	2	Anger	Tone 4	In context	Correct	
11	2	Female	/pu/	1	Anger	Tone 4	In context	Correct	
11	2	Female	/pu/	2	Anger	Tone 4	In context	Correct	
11	1	Male	/pu/	1	Anger	Tone 4	In context	Correct	
11	1	Male	/pu/	2	Anger	Tone 4	In context	Correct	
11	2	Female	/ci/	1	Fear	Tone 1	In context	Correct	
11	2	Female	/ci/	2	Fear	Tone 1	In context	Correct	
11	1	Male	/ci/	1	Fear	Tone 1	In context	Correct	
11	1	Male	/ci/	2	Fear	Tone 1	In context	Correct	
11	2	Female	/fa/	1	Fear	Tone 1	In context	Correct	
11	2	Female	/fa/	2	Fear	Tone 1	In context	Correct	
11	1	Male	/fa/	1	Fear	Tone 1	In context	Correct	
11	1	Male	/fa/	2	Fear	Tone 1	In context	Correct	
11	2	Female	/pu/	1	Fear	Tone 1	In context	Correct	
11	2	Female	/pu/	2	Fear	Tone 1	In context	Correct	
11	1	Male	/pu/	1	Fear	Tone 1	In context	Correct	

11	1
11	2
Male Female
/pu/
/ci/
2	Fear
Fear
Tone 1 In context Tone 2 In context
Correct Correct

11	2
Female
/ci/
Fear
Tone 2 In context Incorrect

11	1
11	1
11	2
11	2
11	1
11	1
11	2
11	2
11	1
11	1
11	2
11	2
11	1
11	1
11	2
11	2
11	1
11	1
Male Male Female Female Male Male Female Female Male Male Female Female Male Male Female Female Male Male
/ci/
/ci/
/fa/
/fa/
/fa/
/fa/
/pu/
/pu/
/pu/
/pu/
/ci/
/ci/
/ci/
/ci/
/fa/
/fa/
/fa/
/fa/
Fear
Fear
Fear
Fear
Fear
Fear
Fear
Fear
Fear
Fear
Fear
Fear
Fear
Fear
Fear
Fear
Fear
Fear
Tone 2 In context Tone 2 In context Tone 2 In context Tone 2 In context Tone 2 In context Tone 2 In context Tone 2 In context Tone 2 In context Tone 2 In context Tone 2 In context Tone 3 In context Tone 3 In context Tone 3 In context Tone 3 In context Tone 3 In context Tone 3 In context Tone 3 In context Tone 3 In context
Correct Correct Correct Correct Correct Correct Correct Correct Correct Correct Correct Correct Correct Correct Correct Correct Correct Correct

11	2
11	2
Female Female
/pu/
/pu/
Fear
Fear
Tone 3 In context Incorrect Tone 3 In context Incorrect

11	1
11	1
11	2
11	2
11	1
11	1
11	2
11	2
11	1
11	1
11	2
Male Male Female Female Male Male Female Female Male Male Female
/pu/
/pu/
/ci/
/ci/
/ci/
/ci/
/fa/
/fa/
/fa/
/fa/
/pu/
Fear
Fear
Fear
Fear
Fear
Fear
Fear
Fear
Fear
Fear
Fear
Tone 3 In context Tone 3 In context Tone 4 In context Tone 4 In context Tone 4 In context Tone 4 In context Tone 4 In context Tone 4 In context Tone 4 In context Tone 4 In context Tone 4 In context
Correct Correct Correct Correct Correct Correct Correct Correct Correct Correct Correct

11	2
Female
/pu/
Fear
Tone 4 In context Incorrect

11	1
11	1
Male Male
/pu/
/pu/
Fear
Fear
Tone 4 In context Tone 4 In context
Correct Correct

11	2
11	2
11	1
11	1
11	2
11	2
11	1
11	1
11	2
11	2
11	1
Female Female Male Male Female Female Male Male Female Female Male
/ci/
/ci/
/ci/
/ci/
/fa/
/fa/
/fa/
/fa/
/pu/
/pu/
/pu/
HappinesTone 1 In context
HappinesTone 1 In context
HappinesTone 1 In context
HappinesTone 1 In context
HappinesTone 1 In context
HappinesTone 1 In context
HappinesTone 1 In context
HappinesTone 1 In context
HappinesTone 1 In context
HappinesTone 1 In context
HappinesTone 1 In context
Correct Correct Correct Correct Correct Correct Correct Correct Correct Correct Correct

11	1
Male
/pu/
HappinesTone 1 In context Incorrect

11	2
11	2
Female Female
/ci/
/ci/
HappinesTone 2 In context
HappinesTone 2 In context
Correct Correct

11	1
11	1
11	2
11	2
11	1
11	1
11	2
11	2
11	1
11	1
11	2
11	2
11	1
11	1
11	2
11	2
11	1
11	1
11	2
11	2
11	1
11	1
11	2
11	2
11	1
11	1
11	2
11	2
11	1
11	1
11	2
11	2
11	1
11	1
Male Male Female Female Male Male Female Female Male Male Female Female Male Male Female Female Male Male Female Female Male Male Female Female Male Male Female Female Male Male Female Female Male Male
/ci/
/ci/
/fa/
/fa/
/fa/
/fa/
/pu/
/pu/
/pu/
/pu/
/ci/
/ci/
/ci/
/ci/
/fa/
/fa/
/fa/
/fa/
/pu/
/pu/
/pu/
/pu/
/ci/
/ci/
/ci/
/ci/
/fa/
/fa/
/fa/
/fa/
/pu/
/pu/
/pu/
/pu/
HappinesTone 2 In context
HappinesTone 2 In context
HappinesTone 2 In context
HappinesTone 2 In context
HappinesTone 2 In context
HappinesTone 2 In context
HappinesTone 2 In context
HappinesTone 2 In context
HappinesTone 2 In context
HappinesTone 2 In context
HappinesTone 3 In context
HappinesTone 3 In context
HappinesTone 3 In context
HappinesTone 3 In context
HappinesTone 3 In context
HappinesTone 3 In context
HappinesTone 3 In context
HappinesTone 3 In context
HappinesTone 3 In context
HappinesTone 3 In context
HappinesTone 3 In context
HappinesTone 3 In context
HappinesTone 4 In context
HappinesTone 4 In context
HappinesTone 4 In context
HappinesTone 4 In context
HappinesTone 4 In context
HappinesTone 4 In context
HappinesTone 4 In context
HappinesTone 4 In context
HappinesTone 4 In context
HappinesTone 4 In context
HappinesTone 4 In context
HappinesTone 4 In context
Correct Correct Correct Correct Correct Correct Correct Correct Correct Correct Correct Correct Correct Correct Correct Correct Correct Correct Correct Correct Correct Correct Correct Correct Correct Correct Correct Correct Correct Correct Correct Correct Correct Correct

11	2
11	2
11	1
11	1
11	2
11	2
11	1
11	1
11	2
11	2
11	1
11	1
11	2
11	2
11	1
11	1
11	2
Female
Female Male Male Female Female Male Male Female Female Male Male Female Female Male Male Female
/ci/
/ci/
/ci/
/ci/
/fa/
/fa/
/fa/
/fa/
/pu/
/pu/
/pu/
/pu/
/ci/
/ci/
/ci/
/ci/
/fa/
Neutral Tone 1 In context
Neutral Tone 1 In context
Neutral Tone 1 In context
Neutral Tone 1 In context
Neutral Tone 1 In context
Neutral Tone 1 In context
Neutral Tone 1 In context
Neutral Tone 1 In context
Neutral Tone 1 In context
Neutral Tone 1 In context
Neutral Tone 1 In context
Neutral Tone 1 In context
Neutral Tone 2 In context
Neutral Tone 2 In context
Neutral Tone 2 In context
Neutral Tone 2 In context
1	Neutral Tone 2 In context
Correct
Correct Correct Correct Correct Correct Correct Correct Correct Correct Correct Correct Correct Correct Correct Correct Correct

11	2	Female	/fa/	2	Neutral	Tone 2	In context	Correct	
11	1	Male	/fa/	1	Neutral	Tone 2	In context	Correct	
11	1	Male	/fa/	2	Neutral	Tone 2	In context	Correct	
11	2	Female	/pu/	1	Neutral	Tone 2	In context	Correct	
11	2	Female	/pu/	2	Neutral	Tone 2	In context	Correct	
11	1	Male	/pu/	1	Neutral	Tone 2	In context	Correct	
11	1	Male	/pu/	2	Neutral	Tone 2	In context	Correct	
11	2	Female	/ci/	1	Neutral	Tone 3	In context	Correct	
11	2	Female	/ci/	2	Neutral	Tone 3	In context	Correct	
11	1	Male	/ci/	1	Neutral	Tone 3	In context	Correct	
11	1	Male	/ci/	2	Neutral	Tone 3	In context	Correct	
11	2	Female	/fa/	1	Neutral	Tone 3	In context	Correct	
11	2	Female	/fa/	2	Neutral	Tone 3	In context	Correct	
11	1	Male	/fa/	1	Neutral	Tone 3	In context	Correct	
11	1	Male	/fa/	2	Neutral	Tone 3	In context	Correct	
11	2	Female	/pu/	1	Neutral	Tone 3	In context	Correct	
11	2	Female	/pu/	2	Neutral	Tone 3	In context	Correct	
11	1	Male	/pu/	1	Neutral	Tone 3	In context	Correct	
11	1	Male	/pu/	2	Neutral	Tone 3	In context	Correct	
11	2	Female	/ci/	1	Neutral	Tone 4	In context	Correct	
11	2	Female	/ci/	2	Neutral	Tone 4	In context	Correct	
11	1	Male	/ci/	1	Neutral	Tone 4	In context	Correct	
11	1	Male	/ci/	2	Neutral	Tone 4	In context	Correct	
11	2	Female	/fa/	1	Neutral	Tone 4	In context	Correct	
11	2	Female	/fa/	2	Neutral	Tone 4	In context	Correct	
11	1	Male	/fa/	1	Neutral	Tone 4	In context	Correct	
11	1	Male	/fa/	2	Neutral	Tone 4	In context	Correct	
11	2	Female	/pu/	1	Neutral	Tone 4	In context	Correct	
11	2	Female	/pu/	2	Neutral	Tone 4	In context	Correct	
11	1	Male	/pu/	1	Neutral	Tone 4	In context	Correct	
11	1	Male	/pu/	2	Neutral	Tone 4	In context	Correct	
11	2	Female	/ci/	1	Sadness	Tone 1	In context	Correct	
11	2	Female	/ci/	2	Sadness	Tone 1	In context	Correct	
11	1	Male	/ci/	1	Sadness	Tone 1	In context	Correct	
11	1	Male	/ci/	2	Sadness	Tone 1	In context	Correct	
11	2	Female	/fa/	1	Sadness	Tone 1	In context	Correct	
11	2	Female	/fa/	2	Sadness	Tone 1	In context	Correct	
11	1	Male	/fa/	1	Sadness	Tone 1	In context	Correct	
11	1	Male	/fa/	2	Sadness	Tone 1	In context	Correct	
11	2	Female	/pu/	1	Sadness	Tone 1	In context	Correct	
11	2	Female	/pu/	2	Sadness	Tone 1	In context	Correct	
11	1	Male	/pu/	1	Sadness	Tone 1	In context	Correct	
11	1	Male	/pu/	2	Sadness	Tone 1	In context	Correct	
11	2	Female	/ci/	1	Sadness	Tone 2	In context	Correct	
11	2	Female	/ci/	2	Sadness	Tone 2	In context	Correct	
11	1	Male	/ci/	1	Sadness	Tone 2	In context	Correct	
11	1	Male	/ci/	2	Sadness	Tone 2	In context	Correct	
11	2	Female	/fa/	1	Sadness	Tone 2	In context	Correct	
11	2	Female	/fa/	2	Sadness	Tone 2	In context	Correct	
11	1	Male	/fa/	1	Sadness	Tone 2	In context	Correct	
11	1	Male	/fa/	2	Sadness	Tone 2	In context	Correct	

11	2	Female	/pu/	1	Sadness	Tone 2	In context	Correct	
11	2	Female	/pu/	2	Sadness	Tone 2	In context	Correct	
11	1	Male	/pu/	1	Sadness	Tone 2	In context	Correct	
11	1	Male	/pu/	2	Sadness	Tone 2	In context	Correct	
11	2	Female	/ci/	1	Sadness	Tone 3	In context	Correct	
11	2	Female	/ci/	2	Sadness	Tone 3	In context	Correct	
11	1	Male	/ci/	1	Sadness	Tone 3	In context	Correct	
11	1	Male	/ci/	2	Sadness	Tone 3	In context	Correct	
11	2	Female	/fa/	1	Sadness	Tone 3	In context	Correct	
11	2	Female	/fa/	2	Sadness	Tone 3	In context	Correct	
11	1	Male	/fa/	1	Sadness	Tone 3	In context	Correct	
11	1	Male	/fa/	2	Sadness	Tone 3	In context	Correct	
11	2	Female	/pu/	1	Sadness	Tone 3	In context	Correct	
11	2	Female	/pu/	2	Sadness	Tone 3	In context	Correct	
11	1	Male	/pu/	1	Sadness	Tone 3	In context	Correct	
11	1	Male	/pu/	2	Sadness	Tone 3	In context	Correct	
11	2	Female	/ci/	1	Sadness	Tone 4	In context	Correct	
11	2	Female	/ci/	2	Sadness	Tone 4	In context	Correct	
11	1	Male	/ci/	1	Sadness	Tone 4	In context	Correct	
11	1	Male	/ci/	2	Sadness	Tone 4	In context	Correct	
11	2	Female	/fa/	1	Sadness	Tone 4	In context	Correct	
11	2	Female	/fa/	2	Sadness	Tone 4	In context	Correct	
11	1	Male	/fa/	1	Sadness	Tone 4	In context	Correct	
11	1	Male	/fa/	2	Sadness	Tone 4	In context	Correct	
11	2	Female	/pu/	1	Sadness	Tone 4	In context	Correct	
11	2	Female	/pu/	2	Sadness	Tone 4	In context	Correct	
11	1	Male	/pu/	1	Sadness	Tone 4	In context	Correct	
11	1	Male	/pu/	2	Sadness	Tone 4	In context	Correct	
12	2	Female	/ci/	1	Anger	Tone 1	In isolation	Correct	
12	2	Female	/ci/	2	Anger	Tone 1	In isolation	Correct	
12	1	Male	/ci/	1	Anger	Tone 1	In isolation	Correct	
12	1	Male	/ci/	2	Anger	Tone 1	In isolation	Correct	
12	2	Female	/fa/	1	Anger	Tone 1	In isolation	Correct	
12	2	Female	/fa/	2	Anger	Tone 1	In isolation	Correct	
12	1	Male	/fa/	1	Anger	Tone 1	In isolation	Correct	
12	1	Male	/fa/	2	Anger	Tone 1	In isolation	Correct	
12	2	Female	/pu/	1	Anger	Tone 1	In isolation	Incorrect	
12	2	Female	/pu/	2	Anger	Tone 1	In isolation	Incorrect	
12	1	Male	/pu/	1	Anger	Tone 1	In isolation	Correct	
12	1	Male	/pu/	2	Anger	Tone 1	In isolation	Correct	
12	2	Female	/ci/	1	Anger	Tone 2	In isolation	Incorrect	
12	2	Female	/ci/	2	Anger	Tone 2	In isolation	Incorrect	
12	1	Male	/ci/	1	Anger	Tone 2	In isolation	Correct	
12	1	Male	/ci/	2	Anger	Tone 2	In isolation	Incorrect	
12	2	Female	/fa/	1	Anger	Tone 2	In isolation	Correct	
12	2	Female	/fa/	2	Anger	Tone 2	In isolation	Correct	
12	1	Male	/fa/	1	Anger	Tone 2	In isolation	Correct	
12	1	Male	/fa/	2	Anger	Tone 2	In isolation	Correct	
12	2	Female	/pu/	1	Anger	Tone 2	In isolation	Correct	
12	2	Female	/pu/	2	Anger	Tone 2	In isolation	Correct	
12	1	Male	/pu/	1	Anger	Tone 2	In isolation	Correct	

12	1	Male	/pu/	2	Anger	Tone 2	In isolation	Correct	
12	2	Female	/ci/	1	Anger	Tone 3	In isolation	Incorrect	
12	2	Female	/ci/	2	Anger	Tone 3	In isolation	Incorrect	
12	1	Male	/ci/	1	Anger	Tone 3	In isolation	Incorrect	
12	1	Male	/ci/	2	Anger	Tone 3	In isolation	Incorrect	
12	2	Female	/fa/	1	Anger	Tone 3	In isolation	Incorrect	
12	2	Female	/fa/	2	Anger	Tone 3	In isolation	Incorrect	
12	1	Male	/fa/	1	Anger	Tone 3	In isolation	Correct	
12	1	Male	/fa/	2	Anger	Tone 3	In isolation	Correct	
12	2	Female	/pu/	1	Anger	Tone 3	In isolation	Incorrect	
12	2	Female	/pu/	2	Anger	Tone 3	In isolation	Incorrect	
12	1	Male	/pu/	1	Anger	Tone 3	In isolation	Incorrect	
12	1	Male	/pu/	2	Anger	Tone 3	In isolation	Incorrect	
12	2	Female	/ci/	1	Anger	Tone 4	In isolation	Correct	
12	2	Female	/ci/	2	Anger	Tone 4	In isolation	Correct	
12	1	Male	/ci/	1	Anger	Tone 4	In isolation	Correct	
12	1	Male	/ci/	2	Anger	Tone 4	In isolation	Correct	
12	2	Female	/fa/	1	Anger	Tone 4	In isolation	Correct	
12	2	Female	/fa/	2	Anger	Tone 4	In isolation	Correct	
12	1	Male	/fa/	1	Anger	Tone 4	In isolation	Correct	
12	1	Male	/fa/	2	Anger	Tone 4	In isolation	Correct	
12	2	Female	/pu/	1	Anger	Tone 4	In isolation	Correct	
12	2	Female	/pu/	2	Anger	Tone 4	In isolation	Correct	
12	1	Male	/pu/	1	Anger	Tone 4	In isolation	Correct	
12	1	Male	/pu/	2	Anger	Tone 4	In isolation	Correct	
12	2	Female	/ci/	1	Fear	Tone 1	In isolation	Correct	
12	2	Female	/ci/	2	Fear	Tone 1	In isolation	Correct	
12	1	Male	/ci/	1	Fear	Tone 1	In isolation	Correct	
12	1	Male	/ci/	2	Fear	Tone 1	In isolation	Correct	
12	2	Female	/fa/	1	Fear	Tone 1	In isolation	Correct	
12	2	Female	/fa/	2	Fear	Tone 1	In isolation	Correct	
12	1	Male	/fa/	1	Fear	Tone 1	In isolation	Correct	
12	1	Male	/fa/	2	Fear	Tone 1	In isolation	Correct	
12	2	Female	/pu/	1	Fear	Tone 1	In isolation	Correct	
12	2	Female	/pu/	2	Fear	Tone 1	In isolation	Correct	
12	1	Male	/pu/	1	Fear	Tone 1	In isolation	Correct	
12	1	Male	/pu/	2	Fear	Tone 1	In isolation	Correct	
12	2	Female	/ci/	1	Fear	Tone 2	In isolation	Incorrect	
12	2	Female	/ci/	2	Fear	Tone 2	In isolation	Incorrect	
12	1	Male	/ci/	1	Fear	Tone 2	In isolation	Correct	
12	1	Male	/ci/	2	Fear	Tone 2	In isolation	Correct	
12	2	Female	/fa/	1	Fear	Tone 2	In isolation	Correct	
12	2	Female	/fa/	2	Fear	Tone 2	In isolation	Correct	
12	1	Male	/fa/	1	Fear	Tone 2	In isolation	Correct	
12	1	Male	/fa/	2	Fear	Tone 2	In isolation	Correct	
12	2	Female	/pu/	1	Fear	Tone 2	In isolation	Incorrect	
12	2	Female	/pu/	2	Fear	Tone 2	In isolation	Incorrect	
12	1	Male	/pu/	1	Fear	Tone 2	In isolation	Incorrect	
12	1	Male	/pu/	2	Fear	Tone 2	In isolation	Correct	
12	2	Female	/ci/	1	Fear	Tone 3	In isolation	Incorrect	
12	2	Female	/ci/	2	Fear	Tone 3	In isolation	Incorrect	

12	1
12	1
12	2
12	2
12	1
12	1
12	2
12	2
12	1
12	1
12	2
12	2
12	1
12	1
12	2
12	2
12	1
12	1
12	2
12	2
12	1
12	1
Male Male Female Female Male Male Female Female Male Male Female Female Male Male Female Female Male Male Female Female Male Male
/ci/
/ci/
/fa/
/fa/
/fa/
/fa/
/pu/
/pu/
/pu/
/pu/
/ci/
/ci/
/ci/
/ci/
/fa/
/fa/
/fa/
/fa/
/pu/
/pu/
/pu/
/pu/
Fear
Fear
Fear
Fear
Fear
Fear
Fear
Fear
Fear
Fear
Fear
Fear
Fear
Fear
Fear
Fear
Fear
Fear
Fear
Fear
Fear
Fear
Tone 3In isolation Incorrect Tone 3In isolation Correct Tone 3In isolation Incorrect Tone 3In isolation Correct Tone 3In isolation Correct Tone 3In isolation Correct Tone 3In isolation Correct Tone 3In isolation Correct Tone 3In isolation Correct Tone 3In isolation Incorrect Tone 4In isolation Correct Tone 4In isolation Correct Tone 4In isolation Correct Tone 4In isolation Correct Tone 4In isolation Incorrect Tone 4In isolation Correct Tone 4In isolation Correct Tone 4In isolation Incorrect Tone 4In isolation Correct Tone 4In isolation Correct Tone 4In isolation Incorrect Tone 4In isolation Incorrect

12	2
12	2
12	1
12	1
12	2
12	2
12	1
12	1
12	2
12	2
12	1
12	1
12	2
12	2
12	1
12	1
12	2
12	2
12	1
12	1
12	2
12	2
12	1
12	1
12	2
12	2
12	1
12	1
12	2
Female Female Male Male Female Female Male Male Female Female Male Male Female Female Male Male Female Female Male Male Female Female Male Male Female Female Male Male Female
/ci/
/ci/
/ci/
/ci/
/fa/
/fa/
/fa/
/fa/
/pu/
/pu/
/pu/
/pu/
/ci/
/ci/
/ci/
/ci/
/fa/
/fa/
/fa/
/fa/
/pu/
/pu/
/pu/
/pu/
/ci/
/ci/
/ci/
/ci/
/fa/
HappinesTone 1In isolation Correct
HappinesTone 1In isolation Incorrect
HappinesTone 1In isolation Correct
HappinesTone 1In isolation Correct
HappinesTone 1In isolation Incorrect
HappinesTone 1In isolation Incorrect
HappinesTone 1In isolation Correct
HappinesTone 1In isolation Correct
HappinesTone 1In isolation Incorrect
HappinesTone 1In isolation Incorrect
HappinesTone 1In isolation Correct
HappinesTone 1In isolation Correct
HappinesTone 2In isolation Incorrect
HappinesTone 2In isolation Correct
HappinesTone 2In isolation Correct
HappinesTone 2In isolation Correct
HappinesTone 2In isolation Correct
HappinesTone 2In isolation Correct
HappinesTone 2In isolation Correct
HappinesTone 2In isolation Correct
HappinesTone 2In isolation Correct
HappinesTone 2In isolation Correct
HappinesTone 2In isolation Correct
HappinesTone 2In isolation Correct
HappinesTone 3In isolation Correct
HappinesTone 3In isolation Incorrect
HappinesTone 3In isolation Correct
HappinesTone 3In isolation Correct
HappinesTone 3In isolation Correct

12	2
12	1
12	1
12	2
12	2
12	1
12	1
12	2
12	2
12	1
12	1
12	2
12	2
12	1
12	1
12	2
12	2
12	1
12	1
Female Male Male Female Female Male Male Female Female Male Male Female Female Male Male Female Female Male Male
/fa/
/fa/
/fa/
/pu/
/pu/
/pu/
/pu/
/ci/
/ci/
/ci/
/ci/
/fa/
/fa/
/fa/
/fa/
/pu/
/pu/
/pu/
/pu/
HappinesTone 3In isolation Correct
HappinesTone 3In isolation Correct
HappinesTone 3In isolation Correct
HappinesTone 3In isolation Incorrect
HappinesTone 3In isolation Correct
HappinesTone 3In isolation Correct
HappinesTone 3In isolation Incorrect
HappinesTone 4In isolation Incorrect
HappinesTone 4In isolation Incorrect
HappinesTone 4In isolation Correct
HappinesTone 4In isolation Correct
HappinesTone 4In isolation Incorrect
HappinesTone 4In isolation Correct
HappinesTone 4In isolation Correct
HappinesTone 4In isolation Correct
HappinesTone 4In isolation Incorrect
HappinesTone 4In isolation Incorrect
HappinesTone 4In isolation Correct
HappinesTone 4In isolation Correct

12	2
12	2
12	1
12	1
12	2
12	2
12	1
12	1
12	2
12	2
12	1
12	1
12	2
12	2
12	1
12	1
12	2
12	2
12	1
12	1
12	2
12	2
12	1
12	1
12	2
12	2
12	1
12	1
12	2
12	2
12	1
12	1
Female Female Male Male Female Female Male Male Female Female Male Male Female Female Male Male Female Female Male Male Female Female Male Male Female Female Male Male Female Female Male Male
/ci/
/ci/
/ci/
/ci/
/fa/
/fa/
/fa/
/fa/
/pu/
/pu/
/pu/
/pu/
/ci/
/ci/
/ci/
/ci/
/fa/
/fa/
/fa/
/fa/
/pu/
/pu/
/pu/
/pu/
/ci/
/ci/
/ci/
/ci/
/fa/
/fa/
/fa/
/fa/
Neutral Tone 1In isolation Correct
Neutral Tone 1In isolation Correct
Neutral Tone 1In isolation Correct
Neutral Tone 1In isolation Correct
Neutral Tone 1In isolation Correct
Neutral Tone 1In isolation Correct
Neutral Tone 1In isolation Correct
Neutral Tone 1In isolation Correct
Neutral Tone 1In isolation Correct
Neutral Tone 1In isolation Correct
Neutral Tone 1In isolation Correct
Neutral Tone 1In isolation Correct
Neutral Tone 2In isolation Correct
Neutral Tone 2In isolation Correct
Neutral Tone 2In isolation Correct
Neutral Tone 2In isolation Correct
Neutral Tone 2In isolation Correct
Neutral Tone 2In isolation Correct
Neutral Tone 2In isolation Correct
Neutral Tone 2In isolation Correct
Neutral Tone 2In isolation Correct
Neutral Tone 2In isolation Correct
Neutral Tone 2In isolation Correct
Neutral Tone 2In isolation Correct
Neutral Tone 3In isolation Correct
Neutral Tone 3In isolation Correct
Neutral Tone 3In isolation Correct
Neutral Tone 3In isolation Correct
Neutral Tone 3In isolation Correct
Neutral Tone 3In isolation Correct
Neutral Tone 3In isolation Correct
Neutral Tone 3In isolation Correct

12	2	Female	/pu/	1	Neutral	Tone 3	In isolation	Correct	
12	2	Female	/pu/	2	Neutral	Tone 3	In isolation	Correct	
12	1	Male	/pu/	1	Neutral	Tone 3	In isolation	Correct	
12	1	Male	/pu/	2	Neutral	Tone 3	In isolation	Correct	
12	2	Female	/ci/	1	Neutral	Tone 4	In isolation	Correct	
12	2	Female	/ci/	2	Neutral	Tone 4	In isolation	Correct	
12	1	Male	/ci/	1	Neutral	Tone 4	In isolation	Correct	
12	1	Male	/ci/	2	Neutral	Tone 4	In isolation	Correct	
12	2	Female	/fa/	1	Neutral	Tone 4	In isolation	Correct	
12	2	Female	/fa/	2	Neutral	Tone 4	In isolation	Correct	
12	1	Male	/fa/	1	Neutral	Tone 4	In isolation	Correct	
12	1	Male	/fa/	2	Neutral	Tone 4	In isolation	Correct	
12	2	Female	/pu/	1	Neutral	Tone 4	In isolation	Correct	
12	2	Female	/pu/	2	Neutral	Tone 4	In isolation	Correct	
12	1	Male	/pu/	1	Neutral	Tone 4	In isolation	Correct	
12	1	Male	/pu/	2	Neutral	Tone 4	In isolation	Correct	
12	2	Female	/ci/	1	Sadness	Tone 1	In isolation	Correct	
12	2	Female	/ci/	2	Sadness	Tone 1	In isolation	Correct	
12	1	Male	/ci/	1	Sadness	Tone 1	In isolation	Correct	
12	1	Male	/ci/	2	Sadness	Tone 1	In isolation	Correct	
12	2	Female	/fa/	1	Sadness	Tone 1	In isolation	Correct	
12	2	Female	/fa/	2	Sadness	Tone 1	In isolation	Correct	
12	1	Male	/fa/	1	Sadness	Tone 1	In isolation	Correct	
12	1	Male	/fa/	2	Sadness	Tone 1	In isolation	Correct	
12	2	Female	/pu/	1	Sadness	Tone 1	In isolation	Correct	
12	2	Female	/pu/	2	Sadness	Tone 1	In isolation	Correct	
12	1	Male	/pu/	1	Sadness	Tone 1	In isolation	Correct	
12	1	Male	/pu/	2	Sadness	Tone 1	In isolation	Correct	
12	2	Female	/ci/	1	Sadness	Tone 2	In isolation	Incorrect	
12	2	Female	/ci/	2	Sadness	Tone 2	In isolation	Incorrect	
12	1	Male	/ci/	1	Sadness	Tone 2	In isolation	Incorrect	
12	1	Male	/ci/	2	Sadness	Tone 2	In isolation	Incorrect	
12	2	Female	/fa/	1	Sadness	Tone 2	In isolation	Incorrect	
12	2	Female	/fa/	2	Sadness	Tone 2	In isolation	Correct	
12	1	Male	/fa/	1	Sadness	Tone 2	In isolation	Incorrect	
12	1	Male	/fa/	2	Sadness	Tone 2	In isolation	Incorrect	
12	2	Female	/pu/	1	Sadness	Tone 2	In isolation	Incorrect	
12	2	Female	/pu/	2	Sadness	Tone 2	In isolation	Incorrect	
12	1	Male	/pu/	1	Sadness	Tone 2	In isolation	Correct	
12	1	Male	/pu/	2	Sadness	Tone 2	In isolation	Incorrect	
12	2	Female	/ci/	1	Sadness	Tone 3	In isolation	Correct	
12	2	Female	/ci/	2	Sadness	Tone 3	In isolation	Incorrect	
12	1	Male	/ci/	1	Sadness	Tone 3	In isolation	Incorrect	
12	1	Male	/ci/	2	Sadness	Tone 3	In isolation	Correct	
12	2	Female	/fa/	1	Sadness	Tone 3	In isolation	Correct	
12	2	Female	/fa/	2	Sadness	Tone 3	In isolation	Correct	
12	1	Male	/fa/	1	Sadness	Tone 3	In isolation	Correct	
12	1	Male	/fa/	2	Sadness	Tone 3	In isolation	Correct	
12	2	Female	/pu/	1	Sadness	Tone 3	In isolation	Correct	
12	2	Female	/pu/	2	Sadness	Tone 3	In isolation	Incorrect	
12	1	Male	/pu/	1	Sadness	Tone 3	In isolation	Correct	

12	1	Male	/pu/	2	Sadness	Tone 3	In isolation	Correct	
12	2	Female	/ci/	1	Sadness	Tone 4	In isolation	Incorrect	
12	2	Female	/ci/	2	Sadness	Tone 4	In isolation	Correct	
12	1	Male	/ci/	1	Sadness	Tone 4	In isolation	Correct	
12	1	Male	/ci/	2	Sadness	Tone 4	In isolation	Correct	
12	2	Female	/fa/	1	Sadness	Tone 4	In isolation	Incorrect	
12	2	Female	/fa/	2	Sadness	Tone 4	In isolation	Incorrect	
12	1	Male	/fa/	1	Sadness	Tone 4	In isolation	Incorrect	
12	1	Male	/fa/	2	Sadness	Tone 4	In isolation	Correct	
12	2	Female	/pu/	1	Sadness	Tone 4	In isolation	Correct	
12	2	Female	/pu/	2	Sadness	Tone 4	In isolation	Correct	
12	1	Male	/pu/	1	Sadness	Tone 4	In isolation	Incorrect	
12	1	Male	/pu/	2	Sadness	Tone 4	In isolation	Correct	
12	2	Female	/ci/	1	Anger	Tone 1	In context	Correct	
12	2	Female	/ci/	2	Anger	Tone 1	In context	Incorrect	
12	1	Male	/ci/	1	Anger	Tone 1	In context	Correct	
12	1	Male	/ci/	2	Anger	Tone 1	In context	Correct	
12	2	Female	/fa/	1	Anger	Tone 1	In context	Correct	
12	2	Female	/fa/	2	Anger	Tone 1	In context	Correct	
12	1	Male	/fa/	1	Anger	Tone 1	In context	Correct	
12	1	Male	/fa/	2	Anger	Tone 1	In context	Correct	
12	2	Female	/pu/	1	Anger	Tone 1	In context	Incorrect	
12	2	Female	/pu/	2	Anger	Tone 1	In context	Incorrect	
12	1	Male	/pu/	1	Anger	Tone 1	In context	Correct	
12	1	Male	/pu/	2	Anger	Tone 1	In context	Correct	
12	2	Female	/ci/	1	Anger	Tone 2	In context	Correct	
12	2	Female	/ci/	2	Anger	Tone 2	In context	Correct	
12	1	Male	/ci/	1	Anger	Tone 2	In context	Correct	
12	1	Male	/ci/	2	Anger	Tone 2	In context	Correct	
12	2	Female	/fa/	1	Anger	Tone 2	In context	Correct	
12	2	Female	/fa/	2	Anger	Tone 2	In context	Correct	
12	1	Male	/fa/	1	Anger	Tone 2	In context	Correct	
12	1	Male	/fa/	2	Anger	Tone 2	In context	Correct	
12	2	Female	/pu/	1	Anger	Tone 2	In context	Correct	
12	2	Female	/pu/	2	Anger	Tone 2	In context	Correct	
12	1	Male	/pu/	1	Anger	Tone 2	In context	Correct	
12	1	Male	/pu/	2	Anger	Tone 2	In context	Correct	
12	2	Female	/ci/	1	Anger	Tone 3	In context	Correct	
12	2	Female	/ci/	2	Anger	Tone 3	In context	Correct	
12	1	Male	/ci/	1	Anger	Tone 3	In context	Correct	
12	1	Male	/ci/	2	Anger	Tone 3	In context	Correct	
12	2	Female	/fa/	1	Anger	Tone 3	In context	Correct	
12	2	Female	/fa/	2	Anger	Tone 3	In context	Correct	
12	1	Male	/fa/	1	Anger	Tone 3	In context	Correct	
12	1	Male	/fa/	2	Anger	Tone 3	In context	Correct	
12	2	Female	/pu/	1	Anger	Tone 3	In context	Correct	
12	2	Female	/pu/	2	Anger	Tone 3	In context	Correct	
12	1	Male	/pu/	1	Anger	Tone 3	In context	Correct	
12	1	Male	/pu/	2	Anger	Tone 3	In context	Correct	
12	2	Female	/ci/	1	Anger	Tone 4	In context	Correct	
12	2	Female	/ci/	2	Anger	Tone 4	In context	Correct	

12	1	Male	/ci/	1	Anger	Tone 4	In context	Correct	
12	1	Male	/ci/	2	Anger	Tone 4	In context	Correct	
12	2	Female	/fa/	1	Anger	Tone 4	In context	Correct	
12	2	Female	/fa/	2	Anger	Tone 4	In context	Correct	
12	1	Male	/fa/	1	Anger	Tone 4	In context	Correct	
12	1	Male	/fa/	2	Anger	Tone 4	In context	Correct	
12	2	Female	/pu/	1	Anger	Tone 4	In context	Correct	
12	2	Female	/pu/	2	Anger	Tone 4	In context	Correct	
12	1	Male	/pu/	1	Anger	Tone 4	In context	Correct	
12	1	Male	/pu/	2	Anger	Tone 4	In context	Correct	
12	2	Female	/ci/	1	Fear	Tone 1	In context	Correct	
12	2	Female	/ci/	2	Fear	Tone 1	In context	Correct	
12	1	Male	/ci/	1	Fear	Tone 1	In context	Correct	
12	1	Male	/ci/	2	Fear	Tone 1	In context	Correct	
12	2	Female	/fa/	1	Fear	Tone 1	In context	Correct	
12	2	Female	/fa/	2	Fear	Tone 1	In context	Correct	
12	1	Male	/fa/	1	Fear	Tone 1	In context	Correct	
12	1	Male	/fa/	2	Fear	Tone 1	In context	Correct	
12	2	Female	/pu/	1	Fear	Tone 1	In context	Correct	
12	2	Female	/pu/	2	Fear	Tone 1	In context	Correct	
12	1	Male	/pu/	1	Fear	Tone 1	In context	Correct	
12	1	Male	/pu/	2	Fear	Tone 1	In context	Correct	
12	2	Female	/ci/	1	Fear	Tone 2	In context	Correct	
12	2	Female	/ci/	2	Fear	Tone 2	In context	Correct	
12	1	Male	/ci/	1	Fear	Tone 2	In context	Correct	
12	1	Male	/ci/	2	Fear	Tone 2	In context	Correct	
12	2	Female	/fa/	1	Fear	Tone 2	In context	Correct	
12	2	Female	/fa/	2	Fear	Tone 2	In context	Correct	
12	1	Male	/fa/	1	Fear	Tone 2	In context	Correct	
12	1	Male	/fa/	2	Fear	Tone 2	In context	Correct	
12	2	Female	/pu/	1	Fear	Tone 2	In context	Correct	
12	2	Female	/pu/	2	Fear	Tone 2	In context	Correct	
12	1	Male	/pu/	1	Fear	Tone 2	In context	Correct	
12	1	Male	/pu/	2	Fear	Tone 2	In context	Correct	
12	2	Female	/ci/	1	Fear	Tone 3	In context	Incorrect	
12	2	Female	/ci/	2	Fear	Tone 3	In context	Incorrect	
12	1	Male	/ci/	1	Fear	Tone 3	In context	Correct	
12	1	Male	/ci/	2	Fear	Tone 3	In context	Correct	
12	2	Female	/fa/	1	Fear	Tone 3	In context	Correct	
12	2	Female	/fa/	2	Fear	Tone 3	In context	Correct	
12	1	Male	/fa/	1	Fear	Tone 3	In context	Correct	
12	1	Male	/fa/	2	Fear	Tone 3	In context	Correct	
12	2	Female	/pu/	1	Fear	Tone 3	In context	Incorrect	
12	2	Female	/pu/	2	Fear	Tone 3	In context	Correct	
12	1	Male	/pu/	1	Fear	Tone 3	In context	Correct	
12	1	Male	/pu/	2	Fear	Tone 3	In context	Correct	
12	2	Female	/ci/	1	Fear	Tone 4	In context	Correct	
12	2	Female	/ci/	2	Fear	Tone 4	In context	Correct	
12	1	Male	/ci/	1	Fear	Tone 4	In context	Correct	
12	1	Male	/ci/	2	Fear	Tone 4	In context	Correct	
12	2	Female	/fa/	1	Fear	Tone 4	In context	Correct	

12	2
12	1
12	1
12	2
12	2
12	1
12	1
Female Male Male Female Female Male Male
/fa/
/fa/
/fa/
/pu/
/pu/
/pu/
/pu/
2	Fear
Fear
Fear
Fear
Fear
Fear
Fear
Tone 4 In context Tone 4 In context Tone 4 In context Tone 4 In context Tone 4 In context Tone 4 In context Tone 4 In context
Correct Correct Correct Correct Correct Correct Correct

12	2
12	2
12	1
12	1
12	2
12	2
12	1
12	1
12	2
12	2
12	1
12	1
12	2
12	2
12	1
12	1
12	2
12	2
12	1
12	1
12	2
12	2
12	1
12	1
12	2
12	2
12	1
12	1
12	2
12	2
12	1
12	1
12	2
12	2
12	1
12	1
Female Female Male Male Female Female Male Male Female Female Male Male Female Female Male Male Female Female Male Male Female Female Male Male Female Female Male Male Female Female Male Male Female Female Male Male
/ci/
/ci/
/ci/
/ci/
/fa/
/fa/
/fa/
/fa/
/pu/
/pu/
/pu/
/pu/
/ci/
/ci/
/ci/
/ci/
/fa/
/fa/
/fa/
/fa/
/pu/
/pu/
/pu/
/pu/
/ci/
/ci/
/ci/
/ci/
/fa/
/fa/
/fa/
/fa/
/pu/
/pu/
/pu/
/pu/
HappinesTone 1 In context
HappinesTone 1 In context
HappinesTone 1 In context
HappinesTone 1 In context
HappinesTone 1 In context
HappinesTone 1 In context
HappinesTone 1 In context
HappinesTone 1 In context
HappinesTone 1 In context
HappinesTone 1 In context
HappinesTone 1 In context
HappinesTone 1 In context
HappinesTone 2 In context
HappinesTone 2 In context
HappinesTone 2 In context
HappinesTone 2 In context
HappinesTone 2 In context
HappinesTone 2 In context
HappinesTone 2 In context
HappinesTone 2 In context
HappinesTone 2 In context
HappinesTone 2 In context
HappinesTone 2 In context
HappinesTone 2 In context
HappinesTone 3 In context
HappinesTone 3 In context
HappinesTone 3 In context
HappinesTone 3 In context
HappinesTone 3 In context
HappinesTone 3 In context
HappinesTone 3 In context
HappinesTone 3 In context
HappinesTone 3 In context
HappinesTone 3 In context
HappinesTone 3 In context
HappinesTone 3 In context
Correct Correct Correct Correct Correct Correct Correct Correct Correct Correct Correct Correct Correct Correct Correct Correct Correct Correct Correct Correct Correct Correct Correct Correct Correct Correct Correct Correct Correct Correct Correct Correct Correct Correct Correct Correct

12	2
12	2
Female
Female
/ci/
/ci/
HappinesTone 4 In context Incorrect
HappinesTone 4 In context Incorrect

12	1
12	1
12	2
12	2
12	1
12	1
Male Male Female Female Male Male
/ci/
/ci/
/fa/
/fa/
/fa/
/fa/
HappinesTone 4 In context
HappinesTone 4 In context
HappinesTone 4 In context
HappinesTone 4 In context
HappinesTone 4 In context
HappinesTone 4 In context
Correct Correct Correct Correct Correct Correct

12	2	Female	/pu/	1	Happines	Tone 4	In context	Incorrect	
12	2	Female	/pu/	2	Happines	Tone 4	In context	Incorrect	
12	1	Male	/pu/	1	Happines	Tone 4	In context	Correct	
12	1	Male	/pu/	2	Happines	Tone 4	In context	Correct	
12	2	Female	/ci/	1	Neutral	Tone 1	In context	Correct	
12	2	Female	/ci/	2	Neutral	Tone 1	In context	Correct	
12	1	Male	/ci/	1	Neutral	Tone 1	In context	Correct	
12	1	Male	/ci/	2	Neutral	Tone 1	In context	Correct	
12	2	Female	/fa/	1	Neutral	Tone 1	In context	Correct	
12	2	Female	/fa/	2	Neutral	Tone 1	In context	Correct	
12	1	Male	/fa/	1	Neutral	Tone 1	In context	Correct	
12	1	Male	/fa/	2	Neutral	Tone 1	In context	Correct	
12	2	Female	/pu/	1	Neutral	Tone 1	In context	Correct	
12	2	Female	/pu/	2	Neutral	Tone 1	In context	Correct	
12	1	Male	/pu/	1	Neutral	Tone 1	In context	Correct	
12	1	Male	/pu/	2	Neutral	Tone 1	In context	Correct	
12	2	Female	/ci/	1	Neutral	Tone 2	In context	Correct	
12	2	Female	/ci/	2	Neutral	Tone 2	In context	Correct	
12	1	Male	/ci/	1	Neutral	Tone 2	In context	Correct	
12	1	Male	/ci/	2	Neutral	Tone 2	In context	Correct	
12	2	Female	/fa/	1	Neutral	Tone 2	In context	Correct	
12	2	Female	/fa/	2	Neutral	Tone 2	In context	Correct	
12	1	Male	/fa/	1	Neutral	Tone 2	In context	Correct	
12	1	Male	/fa/	2	Neutral	Tone 2	In context	Correct	
12	2	Female	/pu/	1	Neutral	Tone 2	In context	Correct	
12	2	Female	/pu/	2	Neutral	Tone 2	In context	Correct	
12	1	Male	/pu/	1	Neutral	Tone 2	In context	Incorrect	
12	1	Male	/pu/	2	Neutral	Tone 2	In context	Correct	
12	2	Female	/ci/	1	Neutral	Tone 3	In context	Correct	
12	2	Female	/ci/	2	Neutral	Tone 3	In context	Correct	
12	1	Male	/ci/	1	Neutral	Tone 3	In context	Correct	
12	1	Male	/ci/	2	Neutral	Tone 3	In context	Correct	
12	2	Female	/fa/	1	Neutral	Tone 3	In context	Correct	
12	2	Female	/fa/	2	Neutral	Tone 3	In context	Correct	
12	1	Male	/fa/	1	Neutral	Tone 3	In context	Correct	
12	1	Male	/fa/	2	Neutral	Tone 3	In context	Correct	
12	2	Female	/pu/	1	Neutral	Tone 3	In context	Correct	
12	2	Female	/pu/	2	Neutral	Tone 3	In context	Correct	
12	1	Male	/pu/	1	Neutral	Tone 3	In context	Correct	
12	1	Male	/pu/	2	Neutral	Tone 3	In context	Correct	
12	2	Female	/ci/	1	Neutral	Tone 4	In context	Correct	
12	2	Female	/ci/	2	Neutral	Tone 4	In context	Correct	
12	1	Male	/ci/	1	Neutral	Tone 4	In context	Correct	
12	1	Male	/ci/	2	Neutral	Tone 4	In context	Correct	
12	2	Female	/fa/	1	Neutral	Tone 4	In context	Correct	
12	2	Female	/fa/	2	Neutral	Tone 4	In context	Correct	
12	1	Male	/fa/	1	Neutral	Tone 4	In context	Correct	
12	1	Male	/fa/	2	Neutral	Tone 4	In context	Correct	
12	2	Female	/pu/	1	Neutral	Tone 4	In context	Correct	
12	2	Female	/pu/	2	Neutral	Tone 4	In context	Correct	
12	1	Male	/pu/	1	Neutral	Tone 4	In context	Correct	

12	1	Male	/pu/	2	Neutral	Tone 4	In context	Correct	
12	2	Female	/ci/	1	Sadness	Tone 1	In context	Correct	
12	2	Female	/ci/	2	Sadness	Tone 1	In context	Correct	
12	1	Male	/ci/	1	Sadness	Tone 1	In context	Correct	
12	1	Male	/ci/	2	Sadness	Tone 1	In context	Correct	
12	2	Female	/fa/	1	Sadness	Tone 1	In context	Correct	
12	2	Female	/fa/	2	Sadness	Tone 1	In context	Correct	
12	1	Male	/fa/	1	Sadness	Tone 1	In context	Incorrect	
12	1	Male	/fa/	2	Sadness	Tone 1	In context	Correct	
12	2	Female	/pu/	1	Sadness	Tone 1	In context	Correct	
12	2	Female	/pu/	2	Sadness	Tone 1	In context	Correct	
12	1	Male	/pu/	1	Sadness	Tone 1	In context	Correct	
12	1	Male	/pu/	2	Sadness	Tone 1	In context	Correct	
12	2	Female	/ci/	1	Sadness	Tone 2	In context	Correct	
12	2	Female	/ci/	2	Sadness	Tone 2	In context	Correct	
12	1	Male	/ci/	1	Sadness	Tone 2	In context	Correct	
12	1	Male	/ci/	2	Sadness	Tone 2	In context	Correct	
12	2	Female	/fa/	1	Sadness	Tone 2	In context	Correct	
12	2	Female	/fa/	2	Sadness	Tone 2	In context	Correct	
12	1	Male	/fa/	1	Sadness	Tone 2	In context	Correct	
12	1	Male	/fa/	2	Sadness	Tone 2	In context	Correct	
12	2	Female	/pu/	1	Sadness	Tone 2	In context	Correct	
12	2	Female	/pu/	2	Sadness	Tone 2	In context	Correct	
12	1	Male	/pu/	1	Sadness	Tone 2	In context	Correct	
12	1	Male	/pu/	2	Sadness	Tone 2	In context	Correct	
12	2	Female	/ci/	1	Sadness	Tone 3	In context	Correct	
12	2	Female	/ci/	2	Sadness	Tone 3	In context	Correct	
12	1	Male	/ci/	1	Sadness	Tone 3	In context	Correct	
12	1	Male	/ci/	2	Sadness	Tone 3	In context	Correct	
12	2	Female	/fa/	1	Sadness	Tone 3	In context	Correct	
12	2	Female	/fa/	2	Sadness	Tone 3	In context	Correct	
12	1	Male	/fa/	1	Sadness	Tone 3	In context	Correct	
12	1	Male	/fa/	2	Sadness	Tone 3	In context	Correct	
12	2	Female	/pu/	1	Sadness	Tone 3	In context	Correct	
12	2	Female	/pu/	2	Sadness	Tone 3	In context	Correct	
12	1	Male	/pu/	1	Sadness	Tone 3	In context	Correct	
12	1	Male	/pu/	2	Sadness	Tone 3	In context	Correct	
12	2	Female	/ci/	1	Sadness	Tone 4	In context	Correct	
12	2	Female	/ci/	2	Sadness	Tone 4	In context	Correct	
12	1	Male	/ci/	1	Sadness	Tone 4	In context	Correct	
12	1	Male	/ci/	2	Sadness	Tone 4	In context	Correct	
12	2	Female	/fa/	1	Sadness	Tone 4	In context	Correct	
12	2	Female	/fa/	2	Sadness	Tone 4	In context	Correct	
12	1	Male	/fa/	1	Sadness	Tone 4	In context	Correct	
12	1	Male	/fa/	2	Sadness	Tone 4	In context	Correct	
12	2	Female	/pu/	1	Sadness	Tone 4	In context	Correct	
12	2	Female	/pu/	2	Sadness	Tone 4	In context	Correct	
12	1	Male	/pu/	1	Sadness	Tone 4	In context	Correct	
12	1	Male	/pu/	2	Sadness	Tone 4	In context	Correct	
13	2	Female	/ci/	1	Anger	Tone 1	In isolation	Correct	
13	2	Female	/ci/	2	Anger	Tone 1	In isolation	Incorrect	

13	1	Male	/ci/	1	Anger	Tone 1	In isolation	Correct	
13	1	Male	/ci/	2	Anger	Tone 1	In isolation	Correct	
13	2	Female	/fa/	1	Anger	Tone 1	In isolation	Incorrect	
13	2	Female	/fa/	2	Anger	Tone 1	In isolation	Correct	
13	1	Male	/fa/	1	Anger	Tone 1	In isolation	Incorrect	
13	1	Male	/fa/	2	Anger	Tone 1	In isolation	Incorrect	
13	2	Female	/pu/	1	Anger	Tone 1	In isolation	Incorrect	
13	2	Female	/pu/	2	Anger	Tone 1	In isolation	Correct	
13	1	Male	/pu/	1	Anger	Tone 1	In isolation	Incorrect	
13	1	Male	/pu/	2	Anger	Tone 1	In isolation	Incorrect	
13	2	Female	/ci/	1	Anger	Tone 2	In isolation	Correct	
13	2	Female	/ci/	2	Anger	Tone 2	In isolation	Incorrect	
13	1	Male	/ci/	1	Anger	Tone 2	In isolation	Incorrect	
13	1	Male	/ci/	2	Anger	Tone 2	In isolation	Incorrect	
13	2	Female	/fa/	1	Anger	Tone 2	In isolation	Correct	
13	2	Female	/fa/	2	Anger	Tone 2	In isolation	Correct	
13	1	Male	/fa/	1	Anger	Tone 2	In isolation	Correct	
13	1	Male	/fa/	2	Anger	Tone 2	In isolation	Incorrect	
13	2	Female	/pu/	1	Anger	Tone 2	In isolation	Correct	
13	2	Female	/pu/	2	Anger	Tone 2	In isolation	Correct	
13	1	Male	/pu/	1	Anger	Tone 2	In isolation	Correct	
13	1	Male	/pu/	2	Anger	Tone 2	In isolation	Incorrect	
13	2	Female	/ci/	1	Anger	Tone 3	In isolation	Incorrect	
13	2	Female	/ci/	2	Anger	Tone 3	In isolation	Incorrect	
13	1	Male	/ci/	1	Anger	Tone 3	In isolation	Incorrect	
13	1	Male	/ci/	2	Anger	Tone 3	In isolation	Incorrect	
13	2	Female	/fa/	1	Anger	Tone 3	In isolation	Incorrect	
13	2	Female	/fa/	2	Anger	Tone 3	In isolation	Correct	
13	1	Male	/fa/	1	Anger	Tone 3	In isolation	Correct	
13	1	Male	/fa/	2	Anger	Tone 3	In isolation	Correct	
13	2	Female	/pu/	1	Anger	Tone 3	In isolation	Incorrect	
13	2	Female	/pu/	2	Anger	Tone 3	In isolation	Incorrect	
13	1	Male	/pu/	1	Anger	Tone 3	In isolation	Incorrect	
13	1	Male	/pu/	2	Anger	Tone 3	In isolation	Correct	
13	2	Female	/ci/	1	Anger	Tone 4	In isolation	Correct	
13	2	Female	/ci/	2	Anger	Tone 4	In isolation	Correct	
13	1	Male	/ci/	1	Anger	Tone 4	In isolation	Correct	
13	1	Male	/ci/	2	Anger	Tone 4	In isolation	Correct	
13	2	Female	/fa/	1	Anger	Tone 4	In isolation	Correct	
13	2	Female	/fa/	2	Anger	Tone 4	In isolation	Correct	
13	1	Male	/fa/	1	Anger	Tone 4	In isolation	Correct	
13	1	Male	/fa/	2	Anger	Tone 4	In isolation	Correct	
13	2	Female	/pu/	1	Anger	Tone 4	In isolation	Correct	
13	2	Female	/pu/	2	Anger	Tone 4	In isolation	Correct	
13	1	Male	/pu/	1	Anger	Tone 4	In isolation	Correct	
13	1	Male	/pu/	2	Anger	Tone 4	In isolation	Correct	
13	2	Female	/ci/	1	Fear	Tone 1	In isolation	Correct	
13	2	Female	/ci/	2	Fear	Tone 1	In isolation	Correct	
13	1	Male	/ci/	1	Fear	Tone 1	In isolation	Correct	
13	1	Male	/ci/	2	Fear	Tone 1	In isolation	Correct	
13	2	Female	/fa/	1	Fear	Tone 1	In isolation	Correct	

13	2	Female	/fa/	2	Fear	Tone 1	In isolation	Correct	
13	1	Male	/fa/	1	Fear	Tone 1	In isolation	Correct	
13	1	Male	/fa/	2	Fear	Tone 1	In isolation	Correct	
13	2	Female	/pu/	1	Fear	Tone 1	In isolation	Correct	
13	2	Female	/pu/	2	Fear	Tone 1	In isolation	Correct	
13	1	Male	/pu/	1	Fear	Tone 1	In isolation	Incorrect	
13	1	Male	/pu/	2	Fear	Tone 1	In isolation	Correct	
13	2	Female	/ci/	1	Fear	Tone 2	In isolation	Incorrect	
13	2	Female	/ci/	2	Fear	Tone 2	In isolation	Incorrect	
13	1	Male	/ci/	1	Fear	Tone 2	In isolation	Incorrect	
13	1	Male	/ci/	2	Fear	Tone 2	In isolation	Incorrect	
13	2	Female	/fa/	1	Fear	Tone 2	In isolation	Correct	
13	2	Female	/fa/	2	Fear	Tone 2	In isolation	Correct	
13	1	Male	/fa/	1	Fear	Tone 2	In isolation	Correct	
13	1	Male	/fa/	2	Fear	Tone 2	In isolation	Correct	
13	2	Female	/pu/	1	Fear	Tone 2	In isolation	Incorrect	
13	2	Female	/pu/	2	Fear	Tone 2	In isolation	Incorrect	
13	1	Male	/pu/	1	Fear	Tone 2	In isolation	Correct	
13	1	Male	/pu/	2	Fear	Tone 2	In isolation	Incorrect	
13	2	Female	/ci/	1	Fear	Tone 3	In isolation	Incorrect	
13	2	Female	/ci/	2	Fear	Tone 3	In isolation	Incorrect	
13	1	Male	/ci/	1	Fear	Tone 3	In isolation	Correct	
13	1	Male	/ci/	2	Fear	Tone 3	In isolation	Correct	
13	2	Female	/fa/	1	Fear	Tone 3	In isolation	Incorrect	
13	2	Female	/fa/	2	Fear	Tone 3	In isolation	Incorrect	
13	1	Male	/fa/	1	Fear	Tone 3	In isolation	Incorrect	
13	1	Male	/fa/	2	Fear	Tone 3	In isolation	Correct	
13	2	Female	/pu/	1	Fear	Tone 3	In isolation	Incorrect	
13	2	Female	/pu/	2	Fear	Tone 3	In isolation	Incorrect	
13	1	Male	/pu/	1	Fear	Tone 3	In isolation	Correct	
13	1	Male	/pu/	2	Fear	Tone 3	In isolation	Incorrect	
13	2	Female	/ci/	1	Fear	Tone 4	In isolation	Incorrect	
13	2	Female	/ci/	2	Fear	Tone 4	In isolation	Incorrect	
13	1	Male	/ci/	1	Fear	Tone 4	In isolation	Correct	
13	1	Male	/ci/	2	Fear	Tone 4	In isolation	Correct	
13	2	Female	/fa/	1	Fear	Tone 4	In isolation	Correct	
13	2	Female	/fa/	2	Fear	Tone 4	In isolation	Correct	
13	1	Male	/fa/	1	Fear	Tone 4	In isolation	Correct	
13	1	Male	/fa/	2	Fear	Tone 4	In isolation	Incorrect	
13	2	Female	/pu/	1	Fear	Tone 4	In isolation	Incorrect	
13	2	Female	/pu/	2	Fear	Tone 4	In isolation	Correct	
13	1	Male	/pu/	1	Fear	Tone 4	In isolation	Incorrect	
13	1	Male	/pu/	2	Fear	Tone 4	In isolation	Correct	
13	2	Female	/ci/	1	Happines	Tone 1	In isolation	Correct	
13	2	Female	/ci/	2	Happines	Tone 1	In isolation	Incorrect	
13	1	Male	/ci/	1	Happines	Tone 1	In isolation	Correct	
13	1	Male	/ci/	2	Happines	Tone 1	In isolation	Correct	
13	2	Female	/fa/	1	Happines	Tone 1	In isolation	Incorrect	
13	2	Female	/fa/	2	Happines	Tone 1	In isolation	Incorrect	
13	1	Male	/fa/	1	Happines	Tone 1	In isolation	Incorrect	
13	1	Male	/fa/	2	Happines	Tone 1	In isolation	Incorrect	

13	2
13	2
13	1
13	1
13	2
13	2
13	1
13	1
13	2
13	2
13	1
13	1
13	2
13	2
13	1
13	1
13	2
13	2
13	1
13	1
13	2
13	2
13	1
13	1
13	2
13	2
13	1
13	1
13	2
13	2
13	1
13	1
13	2
13	2
13	1
13	1
13	2
13	2
13	1
13	1
Female Female Male Male Female Female Male Male Female Female Male Male Female Female Male Male Female Female Male Male Female Female Male Male Female Female Male Male Female Female Male Male Female Female Male Male Female Female Male Male
/pu/
/pu/
/pu/
/pu/
/ci/
/ci/
/ci/
/ci/
/fa/
/fa/
/fa/
/fa/
/pu/
/pu/
/pu/
/pu/
/ci/
/ci/
/ci/
/ci/
/fa/
/fa/
/fa/
/fa/
/pu/
/pu/
/pu/
/pu/
/ci/
/ci/
/ci/
/ci/
/fa/
/fa/
/fa/
/fa/
/pu/
/pu/
/pu/
/pu/
HappinesTone 1In isolation Correct
HappinesTone 1In isolation Correct
HappinesTone 1In isolation Correct
HappinesTone 1In isolation Correct
HappinesTone 2In isolation Incorrect
HappinesTone 2In isolation Incorrect
HappinesTone 2In isolation Incorrect
HappinesTone 2In isolation Incorrect
HappinesTone 2In isolation Correct
HappinesTone 2In isolation Correct
HappinesTone 2In isolation Incorrect
HappinesTone 2In isolation Incorrect
HappinesTone 2In isolation Correct
HappinesTone 2In isolation Correct
HappinesTone 2In isolation Correct
HappinesTone 2In isolation Incorrect
HappinesTone 3In isolation Incorrect
HappinesTone 3In isolation Incorrect
HappinesTone 3In isolation Correct
HappinesTone 3In isolation Correct
HappinesTone 3In isolation Correct
HappinesTone 3In isolation Correct
HappinesTone 3In isolation Correct
HappinesTone 3In isolation Correct
HappinesTone 3In isolation Incorrect
HappinesTone 3In isolation Incorrect
HappinesTone 3In isolation Correct
HappinesTone 3In isolation Correct
HappinesTone 4In isolation Incorrect
HappinesTone 4In isolation Incorrect
HappinesTone 4In isolation Correct
HappinesTone 4In isolation Correct
HappinesTone 4In isolation Incorrect
HappinesTone 4In isolation Correct
HappinesTone 4In isolation Correct
HappinesTone 4In isolation Correct
HappinesTone 4In isolation Incorrect
HappinesTone 4In isolation Incorrect
HappinesTone 4In isolation Correct
HappinesTone 4In isolation Correct

13	2
13	2
13	1
13	1
13	2
13	2
13	1
13	1
13	2
13	2
13	1
Female
Female Male Male Female Female Male Male Female Female Male
/ci/
/ci/
/ci/
/ci/
/fa/
/fa/
/fa/
/fa/
/pu/
/pu/
/pu/
Neutral Tone 1In isolation Correct
Neutral Tone 1In isolation Correct
Neutral Tone 1In isolation Correct
Neutral Tone 1In isolation Correct
Neutral Tone 1In isolation Correct
Neutral Tone 1In isolation Correct
Neutral Tone 1In isolation Correct
Neutral Tone 1In isolation Correct
Neutral Tone 1In isolation Correct
Neutral Tone 1In isolation Correct
1	Neutral Tone 1In isolation Correct

13	1	Male	/pu/	2	Neutral	Tone 1	In isolation	Correct	
13	2	Female	/ci/	1	Neutral	Tone 2	In isolation	Correct	
13	2	Female	/ci/	2	Neutral	Tone 2	In isolation	Correct	
13	1	Male	/ci/	1	Neutral	Tone 2	In isolation	Correct	
13	1	Male	/ci/	2	Neutral	Tone 2	In isolation	Incorrect	
13	2	Female	/fa/	1	Neutral	Tone 2	In isolation	Correct	
13	2	Female	/fa/	2	Neutral	Tone 2	In isolation	Correct	
13	1	Male	/fa/	1	Neutral	Tone 2	In isolation	Correct	
13	1	Male	/fa/	2	Neutral	Tone 2	In isolation	Correct	
13	2	Female	/pu/	1	Neutral	Tone 2	In isolation	Correct	
13	2	Female	/pu/	2	Neutral	Tone 2	In isolation	Correct	
13	1	Male	/pu/	1	Neutral	Tone 2	In isolation	Correct	
13	1	Male	/pu/	2	Neutral	Tone 2	In isolation	Correct	
13	2	Female	/ci/	1	Neutral	Tone 3	In isolation	Correct	
13	2	Female	/ci/	2	Neutral	Tone 3	In isolation	Correct	
13	1	Male	/ci/	1	Neutral	Tone 3	In isolation	Correct	
13	1	Male	/ci/	2	Neutral	Tone 3	In isolation	Correct	
13	2	Female	/fa/	1	Neutral	Tone 3	In isolation	Correct	
13	2	Female	/fa/	2	Neutral	Tone 3	In isolation	Correct	
13	1	Male	/fa/	1	Neutral	Tone 3	In isolation	Correct	
13	1	Male	/fa/	2	Neutral	Tone 3	In isolation	Correct	
13	2	Female	/pu/	1	Neutral	Tone 3	In isolation	Correct	
13	2	Female	/pu/	2	Neutral	Tone 3	In isolation	Correct	
13	1	Male	/pu/	1	Neutral	Tone 3	In isolation	Correct	
13	1	Male	/pu/	2	Neutral	Tone 3	In isolation	Correct	
13	2	Female	/ci/	1	Neutral	Tone 4	In isolation	Correct	
13	2	Female	/ci/	2	Neutral	Tone 4	In isolation	Correct	
13	1	Male	/ci/	1	Neutral	Tone 4	In isolation	Correct	
13	1	Male	/ci/	2	Neutral	Tone 4	In isolation	Correct	
13	2	Female	/fa/	1	Neutral	Tone 4	In isolation	Correct	
13	2	Female	/fa/	2	Neutral	Tone 4	In isolation	Correct	
13	1	Male	/fa/	1	Neutral	Tone 4	In isolation	Correct	
13	1	Male	/fa/	2	Neutral	Tone 4	In isolation	Correct	
13	2	Female	/pu/	1	Neutral	Tone 4	In isolation	Correct	
13	2	Female	/pu/	2	Neutral	Tone 4	In isolation	Correct	
13	1	Male	/pu/	1	Neutral	Tone 4	In isolation	Correct	
13	1	Male	/pu/	2	Neutral	Tone 4	In isolation	Correct	
13	2	Female	/ci/	1	Sadness	Tone 1	In isolation	Correct	
13	2	Female	/ci/	2	Sadness	Tone 1	In isolation	Correct	
13	1	Male	/ci/	1	Sadness	Tone 1	In isolation	Correct	
13	1	Male	/ci/	2	Sadness	Tone 1	In isolation	Correct	
13	2	Female	/fa/	1	Sadness	Tone 1	In isolation	Correct	
13	2	Female	/fa/	2	Sadness	Tone 1	In isolation	Correct	
13	1	Male	/fa/	1	Sadness	Tone 1	In isolation	Incorrect	
13	1	Male	/fa/	2	Sadness	Tone 1	In isolation	Correct	
13	2	Female	/pu/	1	Sadness	Tone 1	In isolation	Correct	
13	2	Female	/pu/	2	Sadness	Tone 1	In isolation	Correct	
13	1	Male	/pu/	1	Sadness	Tone 1	In isolation	Incorrect	
13	1	Male	/pu/	2	Sadness	Tone 1	In isolation	Correct	
13	2	Female	/ci/	1	Sadness	Tone 2	In isolation	Incorrect	
13	2	Female	/ci/	2	Sadness	Tone 2	In isolation	Correct	

13	1	Male	/ci/	1	Sadness	Tone 2	In isolation	Incorrect	
13	1	Male	/ci/	2	Sadness	Tone 2	In isolation	Correct	
13	2	Female	/fa/	1	Sadness	Tone 2	In isolation	Correct	
13	2	Female	/fa/	2	Sadness	Tone 2	In isolation	Correct	
13	1	Male	/fa/	1	Sadness	Tone 2	In isolation	Correct	
13	1	Male	/fa/	2	Sadness	Tone 2	In isolation	Correct	
13	2	Female	/pu/	1	Sadness	Tone 2	In isolation	Correct	
13	2	Female	/pu/	2	Sadness	Tone 2	In isolation	Correct	
13	1	Male	/pu/	1	Sadness	Tone 2	In isolation	Correct	
13	1	Male	/pu/	2	Sadness	Tone 2	In isolation	Correct	
13	2	Female	/ci/	1	Sadness	Tone 3	In isolation	Correct	
13	2	Female	/ci/	2	Sadness	Tone 3	In isolation	Incorrect	
13	1	Male	/ci/	1	Sadness	Tone 3	In isolation	Correct	
13	1	Male	/ci/	2	Sadness	Tone 3	In isolation	Correct	
13	2	Female	/fa/	1	Sadness	Tone 3	In isolation	Correct	
13	2	Female	/fa/	2	Sadness	Tone 3	In isolation	Correct	
13	1	Male	/fa/	1	Sadness	Tone 3	In isolation	Correct	
13	1	Male	/fa/	2	Sadness	Tone 3	In isolation	Correct	
13	2	Female	/pu/	1	Sadness	Tone 3	In isolation	Correct	
13	2	Female	/pu/	2	Sadness	Tone 3	In isolation	Incorrect	
13	1	Male	/pu/	1	Sadness	Tone 3	In isolation	Correct	
13	1	Male	/pu/	2	Sadness	Tone 3	In isolation	Correct	
13	2	Female	/ci/	1	Sadness	Tone 4	In isolation	Correct	
13	2	Female	/ci/	2	Sadness	Tone 4	In isolation	Correct	
13	1	Male	/ci/	1	Sadness	Tone 4	In isolation	Incorrect	
13	1	Male	/ci/	2	Sadness	Tone 4	In isolation	Correct	
13	2	Female	/fa/	1	Sadness	Tone 4	In isolation	Correct	
13	2	Female	/fa/	2	Sadness	Tone 4	In isolation	Incorrect	
13	1	Male	/fa/	1	Sadness	Tone 4	In isolation	Correct	
13	1	Male	/fa/	2	Sadness	Tone 4	In isolation	Correct	
13	2	Female	/pu/	1	Sadness	Tone 4	In isolation	Incorrect	
13	2	Female	/pu/	2	Sadness	Tone 4	In isolation	Correct	
13	1	Male	/pu/	1	Sadness	Tone 4	In isolation	Incorrect	
13	1	Male	/pu/	2	Sadness	Tone 4	In isolation	Incorrect	
13	2	Female	/ci/	1	Anger	Tone 1	In context	Correct	
13	2	Female	/ci/	2	Anger	Tone 1	In context	Correct	
13	1	Male	/ci/	1	Anger	Tone 1	In context	Correct	
13	1	Male	/ci/	2	Anger	Tone 1	In context	Correct	
13	2	Female	/fa/	1	Anger	Tone 1	In context	Correct	
13	2	Female	/fa/	2	Anger	Tone 1	In context	Correct	
13	1	Male	/fa/	1	Anger	Tone 1	In context	Correct	
13	1	Male	/fa/	2	Anger	Tone 1	In context	Correct	
13	2	Female	/pu/	1	Anger	Tone 1	In context	Incorrect	
13	2	Female	/pu/	2	Anger	Tone 1	In context	Correct	
13	1	Male	/pu/	1	Anger	Tone 1	In context	Correct	
13	1	Male	/pu/	2	Anger	Tone 1	In context	Correct	
13	2	Female	/ci/	1	Anger	Tone 2	In context	Correct	
13	2	Female	/ci/	2	Anger	Tone 2	In context	Correct	
13	1	Male	/ci/	1	Anger	Tone 2	In context	Correct	
13	1	Male	/ci/	2	Anger	Tone 2	In context	Correct	
13	2	Female	/fa/	1	Anger	Tone 2	In context	Correct	

13	2	Female	/fa/	2	Anger	Tone 2	In context	Correct	
13	1	Male	/fa/	1	Anger	Tone 2	In context	Correct	
13	1	Male	/fa/	2	Anger	Tone 2	In context	Correct	
13	2	Female	/pu/	1	Anger	Tone 2	In context	Correct	
13	2	Female	/pu/	2	Anger	Tone 2	In context	Correct	
13	1	Male	/pu/	1	Anger	Tone 2	In context	Correct	
13	1	Male	/pu/	2	Anger	Tone 2	In context	Correct	
13	2	Female	/ci/	1	Anger	Tone 3	In context	Correct	
13	2	Female	/ci/	2	Anger	Tone 3	In context	Correct	
13	1	Male	/ci/	1	Anger	Tone 3	In context	Correct	
13	1	Male	/ci/	2	Anger	Tone 3	In context	Correct	
13	2	Female	/fa/	1	Anger	Tone 3	In context	Correct	
13	2	Female	/fa/	2	Anger	Tone 3	In context	Correct	
13	1	Male	/fa/	1	Anger	Tone 3	In context	Correct	
13	1	Male	/fa/	2	Anger	Tone 3	In context	Correct	
13	2	Female	/pu/	1	Anger	Tone 3	In context	Correct	
13	2	Female	/pu/	2	Anger	Tone 3	In context	Correct	
13	1	Male	/pu/	1	Anger	Tone 3	In context	Correct	
13	1	Male	/pu/	2	Anger	Tone 3	In context	Correct	
13	2	Female	/ci/	1	Anger	Tone 4	In context	Correct	
13	2	Female	/ci/	2	Anger	Tone 4	In context	Correct	
13	1	Male	/ci/	1	Anger	Tone 4	In context	Correct	
13	1	Male	/ci/	2	Anger	Tone 4	In context	Correct	
13	2	Female	/fa/	1	Anger	Tone 4	In context	Correct	
13	2	Female	/fa/	2	Anger	Tone 4	In context	Correct	
13	1	Male	/fa/	1	Anger	Tone 4	In context	Correct	
13	1	Male	/fa/	2	Anger	Tone 4	In context	Correct	
13	2	Female	/pu/	1	Anger	Tone 4	In context	Correct	
13	2	Female	/pu/	2	Anger	Tone 4	In context	Correct	
13	1	Male	/pu/	1	Anger	Tone 4	In context	Correct	
13	1	Male	/pu/	2	Anger	Tone 4	In context	Correct	
13	2	Female	/ci/	1	Fear	Tone 1	In context	Correct	
13	2	Female	/ci/	2	Fear	Tone 1	In context	Correct	
13	1	Male	/ci/	1	Fear	Tone 1	In context	Correct	
13	1	Male	/ci/	2	Fear	Tone 1	In context	Correct	
13	2	Female	/fa/	1	Fear	Tone 1	In context	Correct	
13	2	Female	/fa/	2	Fear	Tone 1	In context	Correct	
13	1	Male	/fa/	1	Fear	Tone 1	In context	Correct	
13	1	Male	/fa/	2	Fear	Tone 1	In context	Correct	
13	2	Female	/pu/	1	Fear	Tone 1	In context	Correct	
13	2	Female	/pu/	2	Fear	Tone 1	In context	Correct	
13	1	Male	/pu/	1	Fear	Tone 1	In context	Correct	
13	1	Male	/pu/	2	Fear	Tone 1	In context	Correct	
13	2	Female	/ci/	1	Fear	Tone 2	In context	Correct	
13	2	Female	/ci/	2	Fear	Tone 2	In context	Incorrect	
13	1	Male	/ci/	1	Fear	Tone 2	In context	Correct	
13	1	Male	/ci/	2	Fear	Tone 2	In context	Correct	
13	2	Female	/fa/	1	Fear	Tone 2	In context	Correct	
13	2	Female	/fa/	2	Fear	Tone 2	In context	Correct	
13	1	Male	/fa/	1	Fear	Tone 2	In context	Correct	
13	1	Male	/fa/	2	Fear	Tone 2	In context	Correct	

13	2
Female
/pu/
Fear
Tone 2 In context Incorrect

13	2
13	1
13	1
Female Male Male
/pu/
/pu/
/pu/
Fear
Fear
Fear
Tone 2 In context Tone 2 In context Tone 2 In context
Correct Correct Correct

13	2
13	2
Female Female
/ci/
/ci/
Fear
Fear
Tone 3 In context Incorrect Tone 3 In context Incorrect

13	1
13	1
13	2
13	2
13	1
13	1
Male Male Female Female Male Male
/ci/
/ci/
/fa/
/fa/
/fa/
/fa/
Fear
Fear
Fear
Fear
Fear
Fear
Tone 3 In context Tone 3 In context Tone 3 In context Tone 3 In context Tone 3 In context Tone 3 In context
Correct Correct Correct Correct Correct Correct

13	2
13	2
Female Female
/pu/
/pu/
Fear
Fear
Tone 3 In context Incorrect Tone 3 In context Incorrect

13	1
13	1
13	2
13	2
Male Male Female Female
/pu/
/pu/
/ci/
/ci/
Fear
Fear
Fear
Fear
Tone 3 In context Tone 3 In context Tone 4 In context Tone 4 In context
Correct Correct Correct Correct

13	1
Male
/ci/
Fear
Tone 4 In context Incorrect

13	1
13	2
13	2
13	1
13	1
13	2
13	2
Male Female Female Male Male Female Female
/ci/
/fa/
/fa/
/fa/
/fa/
/pu/
/pu/
Fear
Fear
Fear
Fear
Fear
Fear
Fear
Tone 4 In context Tone 4 In context Tone 4 In context Tone 4 In context Tone 4 In context Tone 4 In context Tone 4 In context
Correct Correct Correct Correct Correct Correct Correct

13	1
Male
/pu/
Fear
Tone 4 In context Incorrect

13	1
Male
/pu/
Fear
Tone 4 In context
Correct

13	2
13	2
13	1
13	1
13	2
13	2
13	1
13	1
13	2
13	2
13	1
Female Female Male Male Female Female Male Male Female Female Male
/ci/
/ci/
/ci/
/ci/
/fa/
/fa/
/fa/
/fa/
/pu/
/pu/
/pu/
HappinesTone 1 In context
HappinesTone 1 In context
HappinesTone 1 In context
HappinesTone 1 In context
HappinesTone 1 In context
HappinesTone 1 In context
HappinesTone 1 In context
HappinesTone 1 In context
HappinesTone 1 In context
HappinesTone 1 In context
HappinesTone 1 In context
Correct Correct Correct Correct Correct Correct Correct Correct Correct Correct Correct

13	1
Male
/pu/
HappinesTone 1 In context Incorrect

13	2
13	2
13	1
13	1
13	2
13	2
13	1
13	1
13	2
13	2
13	1
Female Female Male Male Female Female Male Male Female Female Male
/ci/
/ci/
/ci/
/ci/
/fa/
/fa/
/fa/
/fa/
/pu/
/pu/
/pu/
HappinesTone 2 In context
HappinesTone 2 In context
HappinesTone 2 In context
HappinesTone 2 In context
HappinesTone 2 In context
HappinesTone 2 In context
HappinesTone 2 In context
HappinesTone 2 In context
HappinesTone 2 In context
HappinesTone 2 In context
HappinesTone 2 In context
Correct Correct Correct Correct Correct Correct Correct Correct Correct Correct Correct

13	1
13	2
13	2
13	1
13	1
13	2
13	2
13	1
13	1
13	2
13	2
13	1
13	1
13	2
13	2
13	1
13	1
13	2
13	2
13	1
13	1
Male Female Female Male Male Female Female Male Male Female Female Male Male Female Female Male Male Female Female Male Male
/pu/
/ci/
/ci/
/ci/
/ci/
/fa/
/fa/
/fa/
/fa/
/pu/
/pu/
/pu/
/pu/
/ci/
/ci/
/ci/
/ci/
/fa/
/fa/
/fa/
/fa/
HappinesTone 2 In context
HappinesTone 3 In context
HappinesTone 3 In context
HappinesTone 3 In context
HappinesTone 3 In context
HappinesTone 3 In context
HappinesTone 3 In context
HappinesTone 3 In context
HappinesTone 3 In context
HappinesTone 3 In context
HappinesTone 3 In context
HappinesTone 3 In context
HappinesTone 3 In context
HappinesTone 4 In context
HappinesTone 4 In context
HappinesTone 4 In context
HappinesTone 4 In context
HappinesTone 4 In context
HappinesTone 4 In context
HappinesTone 4 In context
HappinesTone 4 In context
Correct Correct Correct Correct Correct Correct Correct Correct Correct Correct Correct Correct Correct Correct Correct Correct Correct Correct Correct Correct Correct

13	2
Female
/pu/
HappinesTone 4 In context Incorrect

13	2
13	1
13	1
Female Male Male
/pu/
/pu/
/pu/
HappinesTone 4 In context
HappinesTone 4 In context
HappinesTone 4 In context
Correct Correct Correct

13	2
13	2
13	1
13	1
13	2
13	2
13	1
13	1
13	2
13	2
13	1
13	1
13	2
13	2
13	1
13	1
13	2
13	2
13	1
13	1
13	2
13	2
13	1
13	1
13	2
13	2
Female Female Male Male Female Female Male Male Female Female Male Male Female Female Male Male Female Female Male Male Female Female Male Male Female Female
/ci/
/ci/
/ci/
/ci/
/fa/
/fa/
/fa/
/fa/
/pu/
/pu/
/pu/
/pu/
/ci/
/ci/
/ci/
/ci/
/fa/
/fa/
/fa/
/fa/
/pu/
/pu/
/pu/
/pu/
/ci/
/ci/
Neutral Tone 1 In context
Neutral Tone 1 In context
Neutral Tone 1 In context
Neutral Tone 1 In context
Neutral Tone 1 In context
Neutral Tone 1 In context
Neutral Tone 1 In context
Neutral Tone 1 In context
Neutral Tone 1 In context
Neutral Tone 1 In context
Neutral Tone 1 In context
Neutral Tone 1 In context
Neutral Tone 2 In context
Neutral Tone 2 In context
Neutral Tone 2 In context
Neutral Tone 2 In context
Neutral Tone 2 In context
Neutral Tone 2 In context
Neutral Tone 2 In context
Neutral Tone 2 In context
Neutral Tone 2 In context
Neutral Tone 2 In context
Neutral Tone 2 In context
Neutral Tone 2 In context
Neutral Tone 3 In context
Neutral Tone 3 In context
Correct Correct Correct Correct Correct Correct Correct Correct Correct Correct Correct Correct Correct Correct Correct Correct Correct Correct Correct Correct Correct Correct Correct Correct Correct Correct

13	1	Male	/ci/	1	Neutral	Tone 3	In context	Correct	
13	1	Male	/ci/	2	Neutral	Tone 3	In context	Correct	
13	2	Female	/fa/	1	Neutral	Tone 3	In context	Correct	
13	2	Female	/fa/	2	Neutral	Tone 3	In context	Correct	
13	1	Male	/fa/	1	Neutral	Tone 3	In context	Correct	
13	1	Male	/fa/	2	Neutral	Tone 3	In context	Correct	
13	2	Female	/pu/	1	Neutral	Tone 3	In context	Correct	
13	2	Female	/pu/	2	Neutral	Tone 3	In context	Correct	
13	1	Male	/pu/	1	Neutral	Tone 3	In context	Correct	
13	1	Male	/pu/	2	Neutral	Tone 3	In context	Correct	
13	2	Female	/ci/	1	Neutral	Tone 4	In context	Correct	
13	2	Female	/ci/	2	Neutral	Tone 4	In context	Correct	
13	1	Male	/ci/	1	Neutral	Tone 4	In context	Correct	
13	1	Male	/ci/	2	Neutral	Tone 4	In context	Correct	
13	2	Female	/fa/	1	Neutral	Tone 4	In context	Correct	
13	2	Female	/fa/	2	Neutral	Tone 4	In context	Correct	
13	1	Male	/fa/	1	Neutral	Tone 4	In context	Correct	
13	1	Male	/fa/	2	Neutral	Tone 4	In context	Correct	
13	2	Female	/pu/	1	Neutral	Tone 4	In context	Correct	
13	2	Female	/pu/	2	Neutral	Tone 4	In context	Correct	
13	1	Male	/pu/	1	Neutral	Tone 4	In context	Correct	
13	1	Male	/pu/	2	Neutral	Tone 4	In context	Correct	
13	2	Female	/ci/	1	Sadness	Tone 1	In context	Correct	
13	2	Female	/ci/	2	Sadness	Tone 1	In context	Correct	
13	1	Male	/ci/	1	Sadness	Tone 1	In context	Correct	
13	1	Male	/ci/	2	Sadness	Tone 1	In context	Correct	
13	2	Female	/fa/	1	Sadness	Tone 1	In context	Correct	
13	2	Female	/fa/	2	Sadness	Tone 1	In context	Correct	
13	1	Male	/fa/	1	Sadness	Tone 1	In context	Incorrect	
13	1	Male	/fa/	2	Sadness	Tone 1	In context	Correct	
13	2	Female	/pu/	1	Sadness	Tone 1	In context	Correct	
13	2	Female	/pu/	2	Sadness	Tone 1	In context	Correct	
13	1	Male	/pu/	1	Sadness	Tone 1	In context	Correct	
13	1	Male	/pu/	2	Sadness	Tone 1	In context	Correct	
13	2	Female	/ci/	1	Sadness	Tone 2	In context	Correct	
13	2	Female	/ci/	2	Sadness	Tone 2	In context	Incorrect	
13	1	Male	/ci/	1	Sadness	Tone 2	In context	Correct	
13	1	Male	/ci/	2	Sadness	Tone 2	In context	Correct	
13	2	Female	/fa/	1	Sadness	Tone 2	In context	Correct	
13	2	Female	/fa/	2	Sadness	Tone 2	In context	Correct	
13	1	Male	/fa/	1	Sadness	Tone 2	In context	Correct	
13	1	Male	/fa/	2	Sadness	Tone 2	In context	Correct	
13	2	Female	/pu/	1	Sadness	Tone 2	In context	Correct	
13	2	Female	/pu/	2	Sadness	Tone 2	In context	Correct	
13	1	Male	/pu/	1	Sadness	Tone 2	In context	Correct	
13	1	Male	/pu/	2	Sadness	Tone 2	In context	Correct	
13	2	Female	/ci/	1	Sadness	Tone 3	In context	Correct	
13	2	Female	/ci/	2	Sadness	Tone 3	In context	Incorrect	
13	1	Male	/ci/	1	Sadness	Tone 3	In context	Correct	
13	1	Male	/ci/	2	Sadness	Tone 3	In context	Correct	
13	2	Female	/fa/	1	Sadness	Tone 3	In context	Incorrect	

13	2	Female	/fa/	2	Sadness	Tone 3	In context	Incorrect	
13	1	Male	/fa/	1	Sadness	Tone 3	In context	Correct	
13	1	Male	/fa/	2	Sadness	Tone 3	In context	Correct	
13	2	Female	/pu/	1	Sadness	Tone 3	In context	Correct	
13	2	Female	/pu/	2	Sadness	Tone 3	In context	Correct	
13	1	Male	/pu/	1	Sadness	Tone 3	In context	Correct	
13	1	Male	/pu/	2	Sadness	Tone 3	In context	Correct	
13	2	Female	/ci/	1	Sadness	Tone 4	In context	Correct	
13	2	Female	/ci/	2	Sadness	Tone 4	In context	Correct	
13	1	Male	/ci/	1	Sadness	Tone 4	In context	Correct	
13	1	Male	/ci/	2	Sadness	Tone 4	In context	Correct	
13	2	Female	/fa/	1	Sadness	Tone 4	In context	Correct	
13	2	Female	/fa/	2	Sadness	Tone 4	In context	Correct	
13	1	Male	/fa/	1	Sadness	Tone 4	In context	Correct	
13	1	Male	/fa/	2	Sadness	Tone 4	In context	Correct	
13	2	Female	/pu/	1	Sadness	Tone 4	In context	Correct	
13	2	Female	/pu/	2	Sadness	Tone 4	In context	Correct	
13	1	Male	/pu/	1	Sadness	Tone 4	In context	Correct	
13	1	Male	/pu/	2	Sadness	Tone 4	In context	Correct	
14	2	Female	/ci/	1	Anger	Tone 1	In isolation	Correct	
14	2	Female	/ci/	2	Anger	Tone 1	In isolation	Correct	
14	1	Male	/ci/	1	Anger	Tone 1	In isolation	Correct	
14	1	Male	/ci/	2	Anger	Tone 1	In isolation	Correct	
14	2	Female	/fa/	1	Anger	Tone 1	In isolation	Correct	
14	2	Female	/fa/	2	Anger	Tone 1	In isolation	Correct	
14	1	Male	/fa/	1	Anger	Tone 1	In isolation	Correct	
14	1	Male	/fa/	2	Anger	Tone 1	In isolation	Incorrect	
14	2	Female	/pu/	1	Anger	Tone 1	In isolation	Correct	
14	2	Female	/pu/	2	Anger	Tone 1	In isolation	Correct	
14	1	Male	/pu/	1	Anger	Tone 1	In isolation	Correct	
14	1	Male	/pu/	2	Anger	Tone 1	In isolation	Correct	
14	2	Female	/ci/	1	Anger	Tone 2	In isolation	Incorrect	
14	2	Female	/ci/	2	Anger	Tone 2	In isolation	Incorrect	
14	1	Male	/ci/	1	Anger	Tone 2	In isolation	Correct	
14	1	Male	/ci/	2	Anger	Tone 2	In isolation	Correct	
14	2	Female	/fa/	1	Anger	Tone 2	In isolation	Correct	
14	2	Female	/fa/	2	Anger	Tone 2	In isolation	Correct	
14	1	Male	/fa/	1	Anger	Tone 2	In isolation	Correct	
14	1	Male	/fa/	2	Anger	Tone 2	In isolation	Correct	
14	2	Female	/pu/	1	Anger	Tone 2	In isolation	Correct	
14	2	Female	/pu/	2	Anger	Tone 2	In isolation	Correct	
14	1	Male	/pu/	1	Anger	Tone 2	In isolation	Correct	
14	1	Male	/pu/	2	Anger	Tone 2	In isolation	Correct	
14	2	Female	/ci/	1	Anger	Tone 3	In isolation	Incorrect	
14	2	Female	/ci/	2	Anger	Tone 3	In isolation	Incorrect	
14	1	Male	/ci/	1	Anger	Tone 3	In isolation	Incorrect	
14	1	Male	/ci/	2	Anger	Tone 3	In isolation	Incorrect	
14	2	Female	/fa/	1	Anger	Tone 3	In isolation	Incorrect	
14	2	Female	/fa/	2	Anger	Tone 3	In isolation	Incorrect	
14	1	Male	/fa/	1	Anger	Tone 3	In isolation	Correct	
14	1	Male	/fa/	2	Anger	Tone 3	In isolation	Correct	

14	2	Female	/pu/	1	Anger	Tone 3	In isolation	Incorrect	
14	2	Female	/pu/	2	Anger	Tone 3	In isolation	Incorrect	
14	1	Male	/pu/	1	Anger	Tone 3	In isolation	Incorrect	
14	1	Male	/pu/	2	Anger	Tone 3	In isolation	Incorrect	
14	2	Female	/ci/	1	Anger	Tone 4	In isolation	Incorrect	
14	2	Female	/ci/	2	Anger	Tone 4	In isolation	Incorrect	
14	1	Male	/ci/	1	Anger	Tone 4	In isolation	Incorrect	
14	1	Male	/ci/	2	Anger	Tone 4	In isolation	Correct	
14	2	Female	/fa/	1	Anger	Tone 4	In isolation	Correct	
14	2	Female	/fa/	2	Anger	Tone 4	In isolation	Correct	
14	1	Male	/fa/	1	Anger	Tone 4	In isolation	Correct	
14	1	Male	/fa/	2	Anger	Tone 4	In isolation	Correct	
14	2	Female	/pu/	1	Anger	Tone 4	In isolation	Incorrect	
14	2	Female	/pu/	2	Anger	Tone 4	In isolation	Correct	
14	1	Male	/pu/	1	Anger	Tone 4	In isolation	Incorrect	
14	1	Male	/pu/	2	Anger	Tone 4	In isolation	Incorrect	
14	2	Female	/ci/	1	Fear	Tone 1	In isolation	Correct	
14	2	Female	/ci/	2	Fear	Tone 1	In isolation	Correct	
14	1	Male	/ci/	1	Fear	Tone 1	In isolation	Correct	
14	1	Male	/ci/	2	Fear	Tone 1	In isolation	Correct	
14	2	Female	/fa/	1	Fear	Tone 1	In isolation	Correct	
14	2	Female	/fa/	2	Fear	Tone 1	In isolation	Correct	
14	1	Male	/fa/	1	Fear	Tone 1	In isolation	Correct	
14	1	Male	/fa/	2	Fear	Tone 1	In isolation	Correct	
14	2	Female	/pu/	1	Fear	Tone 1	In isolation	Correct	
14	2	Female	/pu/	2	Fear	Tone 1	In isolation	Correct	
14	1	Male	/pu/	1	Fear	Tone 1	In isolation	Correct	
14	1	Male	/pu/	2	Fear	Tone 1	In isolation	Correct	
14	2	Female	/ci/	1	Fear	Tone 2	In isolation	Incorrect	
14	2	Female	/ci/	2	Fear	Tone 2	In isolation	Incorrect	
14	1	Male	/ci/	1	Fear	Tone 2	In isolation	Correct	
14	1	Male	/ci/	2	Fear	Tone 2	In isolation	Correct	
14	2	Female	/fa/	1	Fear	Tone 2	In isolation	Correct	
14	2	Female	/fa/	2	Fear	Tone 2	In isolation	Correct	
14	1	Male	/fa/	1	Fear	Tone 2	In isolation	Correct	
14	1	Male	/fa/	2	Fear	Tone 2	In isolation	Correct	
14	2	Female	/pu/	1	Fear	Tone 2	In isolation	Correct	
14	2	Female	/pu/	2	Fear	Tone 2	In isolation	Correct	
14	1	Male	/pu/	1	Fear	Tone 2	In isolation	Correct	
14	1	Male	/pu/	2	Fear	Tone 2	In isolation	Correct	
14	2	Female	/ci/	1	Fear	Tone 3	In isolation	Incorrect	
14	2	Female	/ci/	2	Fear	Tone 3	In isolation	Incorrect	
14	1	Male	/ci/	1	Fear	Tone 3	In isolation	Incorrect	
14	1	Male	/ci/	2	Fear	Tone 3	In isolation	Correct	
14	2	Female	/fa/	1	Fear	Tone 3	In isolation	Correct	
14	2	Female	/fa/	2	Fear	Tone 3	In isolation	Incorrect	
14	1	Male	/fa/	1	Fear	Tone 3	In isolation	Incorrect	
14	1	Male	/fa/	2	Fear	Tone 3	In isolation	Incorrect	
14	2	Female	/pu/	1	Fear	Tone 3	In isolation	Incorrect	
14	2	Female	/pu/	2	Fear	Tone 3	In isolation	Incorrect	
14	1	Male	/pu/	1	Fear	Tone 3	In isolation	Incorrect	

14	1
14	2
14	2
14	1
14	1
14	2
14	2
14	1
14	1
14	2
14	2
14	1
14	1
Male Female Female Male Male Female Female Male Male Female Female Male Male
/pu/
/ci/
/ci/
/ci/
/ci/
/fa/
/fa/
/fa/
/fa/
/pu/
/pu/
/pu/
/pu/
2	Fear
Fear
Fear
Fear
Fear
Fear
Fear
Fear
Fear
Fear
Fear
Fear
Fear
Tone 3In isolation Incorrect Tone 4In isolation Correct Tone 4In isolation Correct Tone 4In isolation Incorrect Tone 4In isolation Correct Tone 4In isolation Correct Tone 4In isolation Correct Tone 4In isolation Correct Tone 4In isolation Correct Tone 4In isolation Correct Tone 4In isolation Correct Tone 4In isolation Correct Tone 4In isolation Correct

14	2
14	2
14	1
14	1
14	2
14	2
14	1
14	1
14	2
14	2
14	1
14	1
14	2
14	2
14	1
14	1
14	2
14	2
14	1
14	1
14	2
14	2
14	1
14	1
14	2
14	2
14	1
14	1
14	2
14	2
14	1
14	1
14	2
14	2
14	1
14	1
14	2
14	2
Female Female Male Male Female Female Male Male Female Female Male Male Female Female Male Male Female Female Male Male Female Female Male Male Female Female Male Male Female Female Male Male Female Female Male Male Female Female
/ci/
/ci/
/ci/
/ci/
/fa/
/fa/
/fa/
/fa/
/pu/
/pu/
/pu/
/pu/
/ci/
/ci/
/ci/
/ci/
/fa/
/fa/
/fa/
/fa/
/pu/
/pu/
/pu/
/pu/
/ci/
/ci/
/ci/
/ci/
/fa/
/fa/
/fa/
/fa/
/pu/
/pu/
/pu/
/pu/
/ci/
/ci/
HappinesTone 1In isolation Correct
HappinesTone 1In isolation Incorrect
HappinesTone 1In isolation Correct
HappinesTone 1In isolation Correct
HappinesTone 1In isolation Incorrect
HappinesTone 1In isolation Incorrect
HappinesTone 1In isolation Correct
HappinesTone 1In isolation Correct
HappinesTone 1In isolation Incorrect
HappinesTone 1In isolation Incorrect
HappinesTone 1In isolation Correct
HappinesTone 1In isolation Correct
HappinesTone 2In isolation Correct
HappinesTone 2In isolation Incorrect
HappinesTone 2In isolation Correct
HappinesTone 2In isolation Correct
HappinesTone 2In isolation Correct
HappinesTone 2In isolation Correct
HappinesTone 2In isolation Correct
HappinesTone 2In isolation Correct
HappinesTone 2In isolation Correct
HappinesTone 2In isolation Correct
HappinesTone 2In isolation Correct
HappinesTone 2In isolation Correct
HappinesTone 3In isolation Correct
HappinesTone 3In isolation Correct
HappinesTone 3In isolation Correct
HappinesTone 3In isolation Correct
HappinesTone 3In isolation Correct
HappinesTone 3In isolation Correct
HappinesTone 3In isolation Correct
HappinesTone 3In isolation Correct
HappinesTone 3In isolation Correct
HappinesTone 3In isolation Correct
HappinesTone 3In isolation Correct
HappinesTone 3In isolation Incorrect
HappinesTone 4In isolation Incorrect
HappinesTone 4In isolation Incorrect

14	1
14	1
14	2
14	2
14	1
14	1
14	2
14	2
14	1
14	1
Male Male Female Female Male Male Female Female Male Male
/ci/
/ci/
/fa/
/fa/
/fa/
/fa/
/pu/
/pu/
/pu/
/pu/
HappinesTone 4In isolation Incorrect
HappinesTone 4In isolation Correct
HappinesTone 4In isolation Correct
HappinesTone 4In isolation Incorrect
HappinesTone 4In isolation Correct
HappinesTone 4In isolation Correct
HappinesTone 4In isolation Incorrect
HappinesTone 4In isolation Incorrect
HappinesTone 4In isolation Correct
HappinesTone 4In isolation Correct

14	2
14	2
14	1
14	1
14	2
14	2
14	1
14	1
14	2
14	2
14	1
14	1
14	2
14	2
14	1
14	1
14	2
14	2
14	1
14	1
14	2
14	2
14	1
14	1
14	2
14	2
14	1
14	1
14	2
14	2
14	1
14	1
14	2
14	2
14	1
14	1
14	2
14	2
14	1
14	1
14	2
Female Female Male Male Female Female Male Male Female Female Male Male Female Female Male Male Female Female Male Male Female Female Male Male Female Female Male Male Female Female Male Male Female Female Male Male Female Female Male Male Female
/ci/
/ci/
/ci/
/ci/
/fa/
/fa/
/fa/
/fa/
/pu/
/pu/
/pu/
/pu/
/ci/
/ci/
/ci/
/ci/
/fa/
/fa/
/fa/
/fa/
/pu/
/pu/
/pu/
/pu/
/ci/
/ci/
/ci/
/ci/
/fa/
/fa/
/fa/
/fa/
/pu/
/pu/
/pu/
/pu/
/ci/
/ci/
/ci/
/ci/
/fa/
Neutral Tone 1In isolation Correct
Neutral Tone 1In isolation Correct
Neutral Tone 1In isolation Correct
Neutral Tone 1In isolation Correct
Neutral Tone 1In isolation Correct
Neutral Tone 1In isolation Correct
Neutral Tone 1In isolation Correct
Neutral Tone 1In isolation Correct
Neutral Tone 1In isolation Correct
Neutral Tone 1In isolation Correct
Neutral Tone 1In isolation Correct
Neutral Tone 1In isolation Correct
Neutral Tone 2In isolation Correct
Neutral Tone 2In isolation Correct
Neutral Tone 2In isolation Correct
Neutral Tone 2In isolation Incorrect
Neutral Tone 2In isolation Correct
Neutral Tone 2In isolation Correct
Neutral Tone 2In isolation Correct
Neutral Tone 2In isolation Correct
Neutral Tone 2In isolation Correct
Neutral Tone 2In isolation Correct
Neutral Tone 2In isolation Correct
Neutral Tone 2In isolation Correct
Neutral Tone 3In isolation Correct
Neutral Tone 3In isolation Correct
Neutral Tone 3In isolation Correct
Neutral Tone 3In isolation Correct
Neutral Tone 3In isolation Correct
Neutral Tone 3In isolation Correct
Neutral Tone 3In isolation Correct
Neutral Tone 3In isolation Correct
Neutral Tone 3In isolation Correct
Neutral Tone 3In isolation Correct
Neutral Tone 3In isolation Correct
Neutral Tone 3In isolation Correct
Neutral Tone 4In isolation Correct
Neutral Tone 4In isolation Correct
Neutral Tone 4In isolation Correct
Neutral Tone 4In isolation Correct
1	Neutral Tone 4In isolation Correct

14	2	Female	/fa/	2	Neutral	Tone 4	In isolation	Correct	
14	1	Male	/fa/	1	Neutral	Tone 4	In isolation	Correct	
14	1	Male	/fa/	2	Neutral	Tone 4	In isolation	Correct	
14	2	Female	/pu/	1	Neutral	Tone 4	In isolation	Correct	
14	2	Female	/pu/	2	Neutral	Tone 4	In isolation	Correct	
14	1	Male	/pu/	1	Neutral	Tone 4	In isolation	Correct	
14	1	Male	/pu/	2	Neutral	Tone 4	In isolation	Correct	
14	2	Female	/ci/	1	Sadness	Tone 1	In isolation	Correct	
14	2	Female	/ci/	2	Sadness	Tone 1	In isolation	Correct	
14	1	Male	/ci/	1	Sadness	Tone 1	In isolation	Correct	
14	1	Male	/ci/	2	Sadness	Tone 1	In isolation	Correct	
14	2	Female	/fa/	1	Sadness	Tone 1	In isolation	Correct	
14	2	Female	/fa/	2	Sadness	Tone 1	In isolation	Correct	
14	1	Male	/fa/	1	Sadness	Tone 1	In isolation	Incorrect	
14	1	Male	/fa/	2	Sadness	Tone 1	In isolation	Correct	
14	2	Female	/pu/	1	Sadness	Tone 1	In isolation	Correct	
14	2	Female	/pu/	2	Sadness	Tone 1	In isolation	Incorrect	
14	1	Male	/pu/	1	Sadness	Tone 1	In isolation	Correct	
14	1	Male	/pu/	2	Sadness	Tone 1	In isolation	Correct	
14	2	Female	/ci/	1	Sadness	Tone 2	In isolation	Incorrect	
14	2	Female	/ci/	2	Sadness	Tone 2	In isolation	Incorrect	
14	1	Male	/ci/	1	Sadness	Tone 2	In isolation	Correct	
14	1	Male	/ci/	2	Sadness	Tone 2	In isolation	Correct	
14	2	Female	/fa/	1	Sadness	Tone 2	In isolation	Correct	
14	2	Female	/fa/	2	Sadness	Tone 2	In isolation	Correct	
14	1	Male	/fa/	1	Sadness	Tone 2	In isolation	Correct	
14	1	Male	/fa/	2	Sadness	Tone 2	In isolation	Correct	
14	2	Female	/pu/	1	Sadness	Tone 2	In isolation	Correct	
14	2	Female	/pu/	2	Sadness	Tone 2	In isolation	Correct	
14	1	Male	/pu/	1	Sadness	Tone 2	In isolation	Correct	
14	1	Male	/pu/	2	Sadness	Tone 2	In isolation	Correct	
14	2	Female	/ci/	1	Sadness	Tone 3	In isolation	Incorrect	
14	2	Female	/ci/	2	Sadness	Tone 3	In isolation	Incorrect	
14	1	Male	/ci/	1	Sadness	Tone 3	In isolation	Correct	
14	1	Male	/ci/	2	Sadness	Tone 3	In isolation	Correct	
14	2	Female	/fa/	1	Sadness	Tone 3	In isolation	Incorrect	
14	2	Female	/fa/	2	Sadness	Tone 3	In isolation	Incorrect	
14	1	Male	/fa/	1	Sadness	Tone 3	In isolation	Incorrect	
14	1	Male	/fa/	2	Sadness	Tone 3	In isolation	Incorrect	
14	2	Female	/pu/	1	Sadness	Tone 3	In isolation	Correct	
14	2	Female	/pu/	2	Sadness	Tone 3	In isolation	Incorrect	
14	1	Male	/pu/	1	Sadness	Tone 3	In isolation	Correct	
14	1	Male	/pu/	2	Sadness	Tone 3	In isolation	Correct	
14	2	Female	/ci/	1	Sadness	Tone 4	In isolation	Correct	
14	2	Female	/ci/	2	Sadness	Tone 4	In isolation	Correct	
14	1	Male	/ci/	1	Sadness	Tone 4	In isolation	Correct	
14	1	Male	/ci/	2	Sadness	Tone 4	In isolation	Correct	
14	2	Female	/fa/	1	Sadness	Tone 4	In isolation	Incorrect	
14	2	Female	/fa/	2	Sadness	Tone 4	In isolation	Incorrect	
14	1	Male	/fa/	1	Sadness	Tone 4	In isolation	Incorrect	
14	1	Male	/fa/	2	Sadness	Tone 4	In isolation	Correct	

14	2	Female	/pu/	1	Sadness	Tone 4	In isolation	Correct	
14	2	Female	/pu/	2	Sadness	Tone 4	In isolation	Correct	
14	1	Male	/pu/	1	Sadness	Tone 4	In isolation	Correct	
14	1	Male	/pu/	2	Sadness	Tone 4	In isolation	Correct	
14	2	Female	/ci/	1	Anger	Tone 1	In context	Correct	
14	2	Female	/ci/	2	Anger	Tone 1	In context	Correct	
14	1	Male	/ci/	1	Anger	Tone 1	In context	Correct	
14	1	Male	/ci/	2	Anger	Tone 1	In context	Correct	
14	2	Female	/fa/	1	Anger	Tone 1	In context	Correct	
14	2	Female	/fa/	2	Anger	Tone 1	In context	Correct	
14	1	Male	/fa/	1	Anger	Tone 1	In context	Correct	
14	1	Male	/fa/	2	Anger	Tone 1	In context	Correct	
14	2	Female	/pu/	1	Anger	Tone 1	In context	Incorrect	
14	2	Female	/pu/	2	Anger	Tone 1	In context	Correct	
14	1	Male	/pu/	1	Anger	Tone 1	In context	Correct	
14	1	Male	/pu/	2	Anger	Tone 1	In context	Correct	
14	2	Female	/ci/	1	Anger	Tone 2	In context	Correct	
14	2	Female	/ci/	2	Anger	Tone 2	In context	Correct	
14	1	Male	/ci/	1	Anger	Tone 2	In context	Correct	
14	1	Male	/ci/	2	Anger	Tone 2	In context	Correct	
14	2	Female	/fa/	1	Anger	Tone 2	In context	Correct	
14	2	Female	/fa/	2	Anger	Tone 2	In context	Correct	
14	1	Male	/fa/	1	Anger	Tone 2	In context	Correct	
14	1	Male	/fa/	2	Anger	Tone 2	In context	Correct	
14	2	Female	/pu/	1	Anger	Tone 2	In context	Correct	
14	2	Female	/pu/	2	Anger	Tone 2	In context	Correct	
14	1	Male	/pu/	1	Anger	Tone 2	In context	Correct	
14	1	Male	/pu/	2	Anger	Tone 2	In context	Correct	
14	2	Female	/ci/	1	Anger	Tone 3	In context	Correct	
14	2	Female	/ci/	2	Anger	Tone 3	In context	Correct	
14	1	Male	/ci/	1	Anger	Tone 3	In context	Correct	
14	1	Male	/ci/	2	Anger	Tone 3	In context	Correct	
14	2	Female	/fa/	1	Anger	Tone 3	In context	Correct	
14	2	Female	/fa/	2	Anger	Tone 3	In context	Correct	
14	1	Male	/fa/	1	Anger	Tone 3	In context	Correct	
14	1	Male	/fa/	2	Anger	Tone 3	In context	Correct	
14	2	Female	/pu/	1	Anger	Tone 3	In context	Correct	
14	2	Female	/pu/	2	Anger	Tone 3	In context	Correct	
14	1	Male	/pu/	1	Anger	Tone 3	In context	Correct	
14	1	Male	/pu/	2	Anger	Tone 3	In context	Correct	
14	2	Female	/ci/	1	Anger	Tone 4	In context	Correct	
14	2	Female	/ci/	2	Anger	Tone 4	In context	Correct	
14	1	Male	/ci/	1	Anger	Tone 4	In context	Correct	
14	1	Male	/ci/	2	Anger	Tone 4	In context	Incorrect	
14	2	Female	/fa/	1	Anger	Tone 4	In context	Correct	
14	2	Female	/fa/	2	Anger	Tone 4	In context	Correct	
14	1	Male	/fa/	1	Anger	Tone 4	In context	Correct	
14	1	Male	/fa/	2	Anger	Tone 4	In context	Correct	
14	2	Female	/pu/	1	Anger	Tone 4	In context	Correct	
14	2	Female	/pu/	2	Anger	Tone 4	In context	Correct	
14	1	Male	/pu/	1	Anger	Tone 4	In context	Correct	

14	1	Male	/pu/	2	Anger	Tone 4	In context	Correct	
14	2	Female	/ci/	1	Fear	Tone 1	In context	Correct	
14	2	Female	/ci/	2	Fear	Tone 1	In context	Correct	
14	1	Male	/ci/	1	Fear	Tone 1	In context	Correct	
14	1	Male	/ci/	2	Fear	Tone 1	In context	Correct	
14	2	Female	/fa/	1	Fear	Tone 1	In context	Correct	
14	2	Female	/fa/	2	Fear	Tone 1	In context	Correct	
14	1	Male	/fa/	1	Fear	Tone 1	In context	Correct	
14	1	Male	/fa/	2	Fear	Tone 1	In context	Correct	
14	2	Female	/pu/	1	Fear	Tone 1	In context	Correct	
14	2	Female	/pu/	2	Fear	Tone 1	In context	Correct	
14	1	Male	/pu/	1	Fear	Tone 1	In context	Correct	
14	1	Male	/pu/	2	Fear	Tone 1	In context	Correct	
14	2	Female	/ci/	1	Fear	Tone 2	In context	Correct	
14	2	Female	/ci/	2	Fear	Tone 2	In context	Correct	
14	1	Male	/ci/	1	Fear	Tone 2	In context	Correct	
14	1	Male	/ci/	2	Fear	Tone 2	In context	Correct	
14	2	Female	/fa/	1	Fear	Tone 2	In context	Correct	
14	2	Female	/fa/	2	Fear	Tone 2	In context	Correct	
14	1	Male	/fa/	1	Fear	Tone 2	In context	Correct	
14	1	Male	/fa/	2	Fear	Tone 2	In context	Correct	
14	2	Female	/pu/	1	Fear	Tone 2	In context	Correct	
14	2	Female	/pu/	2	Fear	Tone 2	In context	Incorrect	
14	1	Male	/pu/	1	Fear	Tone 2	In context	Correct	
14	1	Male	/pu/	2	Fear	Tone 2	In context	Correct	
14	2	Female	/ci/	1	Fear	Tone 3	In context	Incorrect	
14	2	Female	/ci/	2	Fear	Tone 3	In context	Correct	
14	1	Male	/ci/	1	Fear	Tone 3	In context	Correct	
14	1	Male	/ci/	2	Fear	Tone 3	In context	Correct	
14	2	Female	/fa/	1	Fear	Tone 3	In context	Correct	
14	2	Female	/fa/	2	Fear	Tone 3	In context	Correct	
14	1	Male	/fa/	1	Fear	Tone 3	In context	Correct	
14	1	Male	/fa/	2	Fear	Tone 3	In context	Correct	
14	2	Female	/pu/	1	Fear	Tone 3	In context	Incorrect	
14	2	Female	/pu/	2	Fear	Tone 3	In context	Incorrect	
14	1	Male	/pu/	1	Fear	Tone 3	In context	Correct	
14	1	Male	/pu/	2	Fear	Tone 3	In context	Correct	
14	2	Female	/ci/	1	Fear	Tone 4	In context	Correct	
14	2	Female	/ci/	2	Fear	Tone 4	In context	Correct	
14	1	Male	/ci/	1	Fear	Tone 4	In context	Correct	
14	1	Male	/ci/	2	Fear	Tone 4	In context	Correct	
14	2	Female	/fa/	1	Fear	Tone 4	In context	Correct	
14	2	Female	/fa/	2	Fear	Tone 4	In context	Correct	
14	1	Male	/fa/	1	Fear	Tone 4	In context	Correct	
14	1	Male	/fa/	2	Fear	Tone 4	In context	Correct	
14	2	Female	/pu/	1	Fear	Tone 4	In context	Correct	
14	2	Female	/pu/	2	Fear	Tone 4	In context	Correct	
14	1	Male	/pu/	1	Fear	Tone 4	In context	Correct	
14	1	Male	/pu/	2	Fear	Tone 4	In context	Correct	
14	2	Female	/ci/	1	Happines	Tone 1	In context	Correct	
14	2	Female	/ci/	2	Happines	Tone 1	In context	Correct	

14	1
14	1
14	2
14	2
14	1
14	1
14	2
14	2
14	1
14	1
14	2
14	2
14	1
14	1
14	2
14	2
14	1
14	1
14	2
14	2
14	1
14	1
14	2
14	2
14	1
14	1
14	2
14	2
14	1
14	1
14	2
14	2
14	1
14	1
Male Male Female Female Male Male Female Female Male Male Female Female Male Male Female Female Male Male Female Female Male Male Female Female Male Male Female Female Male Male Female Female Male Male
/ci/
/ci/
/fa/
/fa/
/fa/
/fa/
/pu/
/pu/
/pu/
/pu/
/ci/
/ci/
/ci/
/ci/
/fa/
/fa/
/fa/
/fa/
/pu/
/pu/
/pu/
/pu/
/ci/
/ci/
/ci/
/ci/
/fa/
/fa/
/fa/
/fa/
/pu/
/pu/
/pu/
/pu/
HappinesTone 1 In context
HappinesTone 1 In context
HappinesTone 1 In context
HappinesTone 1 In context
HappinesTone 1 In context
HappinesTone 1 In context
HappinesTone 1 In context
HappinesTone 1 In context
HappinesTone 1 In context
HappinesTone 1 In context
HappinesTone 2 In context
HappinesTone 2 In context
HappinesTone 2 In context
HappinesTone 2 In context
HappinesTone 2 In context
HappinesTone 2 In context
HappinesTone 2 In context
HappinesTone 2 In context
HappinesTone 2 In context
HappinesTone 2 In context
HappinesTone 2 In context
HappinesTone 2 In context
HappinesTone 3 In context
HappinesTone 3 In context
HappinesTone 3 In context
HappinesTone 3 In context
HappinesTone 3 In context
HappinesTone 3 In context
HappinesTone 3 In context
HappinesTone 3 In context
HappinesTone 3 In context
HappinesTone 3 In context
HappinesTone 3 In context
HappinesTone 3 In context
Correct Correct Correct Correct Correct Correct Correct Correct Correct Correct Correct Correct Correct Correct Correct Correct Correct Correct Correct Correct Correct Correct Correct Correct Correct Correct Correct Correct Correct Correct Correct Correct Correct Correct

14	2
14	2
Female
Female
/ci/
/ci/
HappinesTone 4 In context Incorrect
HappinesTone 4 In context Incorrect

14	1
14	1
14	2
14	2
14	1
14	1
14	2
14	2
14	1
14	1
Male Male Female Female Male Male Female Female Male Male
/ci/
/ci/
/fa/
/fa/
/fa/
/fa/
/pu/
/pu/
/pu/
/pu/
HappinesTone 4 In context
HappinesTone 4 In context
HappinesTone 4 In context
HappinesTone 4 In context
HappinesTone 4 In context
HappinesTone 4 In context
HappinesTone 4 In context
HappinesTone 4 In context
HappinesTone 4 In context
HappinesTone 4 In context
Correct Correct Correct Correct Correct Correct Correct Correct Correct Correct

14	2
14	2
14	1
14	1
14	2
Female Female Male Male Female
/ci/
/ci/
/ci/
/ci/
/fa/
Neutral Tone 1 In context
Neutral Tone 1 In context
Neutral Tone 1 In context
Neutral Tone 1 In context
1	Neutral Tone 1 In context
Correct Correct Correct Correct Correct

14	2	Female	/fa/	2	Neutral	Tone 1	In context	Correct	
14	1	Male	/fa/	1	Neutral	Tone 1	In context	Correct	
14	1	Male	/fa/	2	Neutral	Tone 1	In context	Correct	
14	2	Female	/pu/	1	Neutral	Tone 1	In context	Correct	
14	2	Female	/pu/	2	Neutral	Tone 1	In context	Correct	
14	1	Male	/pu/	1	Neutral	Tone 1	In context	Correct	
14	1	Male	/pu/	2	Neutral	Tone 1	In context	Correct	
14	2	Female	/ci/	1	Neutral	Tone 2	In context	Correct	
14	2	Female	/ci/	2	Neutral	Tone 2	In context	Correct	
14	1	Male	/ci/	1	Neutral	Tone 2	In context	Correct	
14	1	Male	/ci/	2	Neutral	Tone 2	In context	Correct	
14	2	Female	/fa/	1	Neutral	Tone 2	In context	Correct	
14	2	Female	/fa/	2	Neutral	Tone 2	In context	Correct	
14	1	Male	/fa/	1	Neutral	Tone 2	In context	Correct	
14	1	Male	/fa/	2	Neutral	Tone 2	In context	Correct	
14	2	Female	/pu/	1	Neutral	Tone 2	In context	Correct	
14	2	Female	/pu/	2	Neutral	Tone 2	In context	Correct	
14	1	Male	/pu/	1	Neutral	Tone 2	In context	Correct	
14	1	Male	/pu/	2	Neutral	Tone 2	In context	Correct	
14	2	Female	/ci/	1	Neutral	Tone 3	In context	Correct	
14	2	Female	/ci/	2	Neutral	Tone 3	In context	Correct	
14	1	Male	/ci/	1	Neutral	Tone 3	In context	Correct	
14	1	Male	/ci/	2	Neutral	Tone 3	In context	Correct	
14	2	Female	/fa/	1	Neutral	Tone 3	In context	Correct	
14	2	Female	/fa/	2	Neutral	Tone 3	In context	Correct	
14	1	Male	/fa/	1	Neutral	Tone 3	In context	Correct	
14	1	Male	/fa/	2	Neutral	Tone 3	In context	Correct	
14	2	Female	/pu/	1	Neutral	Tone 3	In context	Correct	
14	2	Female	/pu/	2	Neutral	Tone 3	In context	Correct	
14	1	Male	/pu/	1	Neutral	Tone 3	In context	Correct	
14	1	Male	/pu/	2	Neutral	Tone 3	In context	Correct	
14	2	Female	/ci/	1	Neutral	Tone 4	In context	Correct	
14	2	Female	/ci/	2	Neutral	Tone 4	In context	Correct	
14	1	Male	/ci/	1	Neutral	Tone 4	In context	Correct	
14	1	Male	/ci/	2	Neutral	Tone 4	In context	Correct	
14	2	Female	/fa/	1	Neutral	Tone 4	In context	Correct	
14	2	Female	/fa/	2	Neutral	Tone 4	In context	Correct	
14	1	Male	/fa/	1	Neutral	Tone 4	In context	Correct	
14	1	Male	/fa/	2	Neutral	Tone 4	In context	Correct	
14	2	Female	/pu/	1	Neutral	Tone 4	In context	Correct	
14	2	Female	/pu/	2	Neutral	Tone 4	In context	Correct	
14	1	Male	/pu/	1	Neutral	Tone 4	In context	Correct	
14	1	Male	/pu/	2	Neutral	Tone 4	In context	Correct	
14	2	Female	/ci/	1	Sadness	Tone 1	In context	Correct	
14	2	Female	/ci/	2	Sadness	Tone 1	In context	Correct	
14	1	Male	/ci/	1	Sadness	Tone 1	In context	Correct	
14	1	Male	/ci/	2	Sadness	Tone 1	In context	Correct
[truncated: 1,200,050 more chars]
