# Supplementary material for: Emotional tones of voice affect the acoustics and perception of Mandarin tones
Source: PLoS One. 2023 Apr 5;18(4):e0283635. doi: 10.1371/journal.pone.0283635 (PMC10075469; doi:10.1371/journal.pone.0283635)
Supplement: Attachment — Submitted filename: Response to Reviewers.docx [file pone.0283635.s005.docx]

Vera Kempe

Academic Editor

PLOS ONE

February 28, 2022

Dear Dr. Kempe,

We would like to thank you and the reviewers for your detailed and thoughtful comments on our manuscript “Emotional tones of voice affect the acoustics and perception of Mandarin tones” (PONE-D-21-23777). We have prepared a revision based on your feedback. Our itemized response to the concerns raised by the reviewers is appended below. As instructed, we have included a marked-up copy and an unmarked version. Highlights of the revision include:

- We have revised the introduction to make sure the research questions and hypotheses are grounded in the most relevant literature.
- We have redone statistical analyses for Experiment 2 using the mixed-model multinomial regression analysis as suggested by Reviewer 2.
- We have revised our interpretations of the findings based on the updated conceptual foundation and data analyses.
- We have asked a native speaker of English to check our word choice, grammar, and writing style. We have also streamlined the presentation based on her feedback.

We would like to make one correction. In the original manuscript we reported 30 participants in Experiment 2, but it should have been 36. Prior to this study, we had collected pilot data from a group of 30 participants. The pilot data was not included in the current study. We apologize for the error.

Finally, we made the following changes per Journal instructions:

- We have changed “female/male” to “woman/man”, and “gender” to “sex” in accordance with APA general principles.
- We have included the minimal data set underlying the results with our manuscript submission. The data set can be found in Supporting Information.
- We have removed the reference to an unpublished master’s thesis.

Thank you for helping us write a better paper. We look forward to hearing from you again.

Sincerely yours,

____________________

Woeichyn Chu, PhD

Distinguished Professor

***Editor comments:***

*Specifically, the reviewers acknowledge the merits of this study but see major problems with the integration with the current literature as well as with the justification of the design and the appropriateness of the analyses. Because PLOS ONE emphasises methodological soundness over novelty and impact I am minded to give you the opportunity to address the reviewers' comments very carefully. Reviewer 2 makes particularly pertinent and detailed comments that I encourage you to address. I particularly see the need to address the comments regarding ANOVA as an unsuitable statistical approach to these data. In addition, please make sure the data are being made available to the reviewers - you state that they are but the reviewers had trouble finding them. Finally, I would like to point out that request of revision does not constitute guarantee of acceptance of the revised version.*

**Response**: We thank Dr. Kempe for the reminder regarding methodological soundness. We have followed the reviewers’ advice to revamp the conceptual foundation of the study. We have replaced the ANOVA with a mixed-model multinomial regression analysis for Experiment 2. We made sure the data is available to the reviewers.

***Reviewer #1 comments:***

*The study is interesting and relevant. I enjoy reading it very much. The separation between acoustics and perception is wonderful. Please find the points of improvement/discussion below.*

**Response**: We thank Dr. Liu for recognizing the merit of this study.

*1. Is the manuscript technically sound, and do the data support the conclusions?*

*Narratives can appear more concisely throughout the manuscript.*

**Response**: We have revised the manuscript to make it more concise.

*If possible, a bit more discussion on the other way (lexical affecting emotional tone) would be appreciated.*

**Response**: We have added more information about how lexical tones effect the acoustics and perception of emotions:

- In the section “How Emotions Affect Speech Acoustics”, we added two studies showing restricted F0 variations of emotions in other tonal languages (Chong, Kim, & Davis, 2015, for Cantonese; and Luksaneeyanawin, 1998, for Thai). We also added a Mandarin-Japanese study that showed the opposite pattern (Li, Jia, Fang, & Dang, 2013).
- In the section “How Emotions Are Perceived in Speech”, we added discussion of a study on emotion recognition in Mandarin (Wang, Ding, & Gu, 2012).

*There is a potential design issue in the “context” condition, which can be resolved by careful discussion since the experiments have been done.*

**Response**: We have clarified our use of the term “context” throughout the manuscript.

*Based on the previous comment, please double-check the research questions. Make sure the questions asked are the variables being examined.*
 **Response**: We have revised the introduction to make sure the research questions are aligned with the variables being examined.

*2. Has the statistical analysis been performed appropriately and rigorously?

The effect size is omitted in the report.*

**Response**: We have included effect size as instructed.

*3. Have the authors made all data underlying the findings in their manuscript fully available?

Maybe I missed this: it is unclear where (anonymous) data are stored.*

**Response**: We apologize for missing the data in the original submission. As instructed, we have included the minimal data set.

*4. Is the manuscript presented in an intelligible fashion and written in standard English?

The manuscript is understandable but is encouraged to be double-checked on its English writing style. There is space for further improvement.*

**Response**: We have asked a native speaker of English to check our word choice, grammar, and writing style.

*Detailed comments:

Abstract

1. Polish writing, including style, words and grammar*

*A. The starting sentence can be polished to enhance the significance of the study*

**Response**: We have revised the starting sentence to enhance the significance of the study.

*B. “the effect of emotional tones on Mandarin tone acoustics varied depending on specific Mandarin tones, specific emotional tones, and talker gender.” Please rephrase.*

**Response**: We have rephrased this sentence to improve its clarity.

*C. “select materials”: selected? (same in Ln283)*

**Response**: We have changed the word here and elsewhere.

*D. “Emotional tones also affected the four acoustic measures to different degrees.” Please integrate with the narratives prior.*

**Response**: We have integrated this sentence into a previous sentence.

*2. Reflecting on the narratives of Exp.2, clarify whether Exp.1 is presented in isolation or in context.*

**Response**: We have clarified that acoustic analyses in Experiment 1 were conducted on target syllables extracted from a carrier phrase.

*Introduction

Ln48: I understand that the authors adopted a four-basic emotion model. I wonder why the more commonly accepted six-basic emotion model, adding surprise and disgust in the study, is not considered.*

**Response**: We thank Dr. Liu for pointing this out. The four basic emotion model was proposed by Ortony & Turner (1990) and the six basic emotion model was proposed by Ekman (1999). Both were cited extensively: Ortony & Turner (1990) was cited 3107 times and Ekman (1999) 4399 times. We decided to adopt the four emotion model because the four emotions are included in the six-emotion model, and most of the studies on Mandarin have used the four-emotion model (Yuan, Shen, & Chen, 2002; Zhang, Ching, & Kong, 2006; Lin & Fon, 2012; Wang & Qian, 2018; Chang, Young, Li, Chu, & Ho, 2018; Chang, Young, & Yuan, 2010; Wang, Lee, & Ma, 2016). Adopting the four-emotion model would make comparisons with previous studies more straightforward.

Ekman, P. Basic Emotions. In Dalgleish T, Power T, editors, The Handbook of Cognition and Emotion. Sussex: John Wiley & Sons, Ltd.; 1999. p. 45‐60

*Ln51: Please double check "amplitude" or "intensity"*

**Response**: We have replaced “intensity” with “amplitude” so that “amplitude” is used consistently throughout the manuscript.

*Ln70: 1. I have the feeling that these scenarios are restricted to certain context/isolation, or when only key cues are involved. This is later discussed in Ln575. 2. I wonder if these are more like logical possibilities unless the authors argue that different scenarios may occur with different emotional-lexical tone combos.*

**Response**: We agree that the scenarios are more like logical possibilities, which did not contribute much to motivating the research questions. This point is also made by Reviewer 2. We have removed this paragraph and subsequent discussions that were based on this taxonomy.

*Ln85: I wonder whether the other way around is worth highlighting, namely, how lexical tone affects emotion (e.g., emotional tone) perception? This is analysed and discussed after all (e.g., Ln805, later asymmetry)*

**Response**: As noted in our earlier response, we have added more information regarding the effect of lexical tone on emotion perception to the section “how emotions are perceived in speech”.

*Ln192: Does falling tone affect anger perception?*

**Response**: Liang and Chen (2019) examined Mandarin tone perception but not emotional perception. Results from our Experiment 2 showed that anger perception in isolated syllables was more accurate in the falling tone (T4: 93%) compared to other tones (T1: 77%; T2: 76%; T3: 53%). Anger perception in syllables embedded in the carrier phrase was comparable among the four tones (T1: 97%; T2: 97%; T3: 96%, T4: 99%).

*Ln198-212: Some information here seems to have been mentioned earlier.*

**Response**: This paragraph was intended to offer a summary of the studies reviewed in this section. In light of the reviewer’s comment, we have deleted this summary.

*Ln232, 234: Wang et al. - repetitive presentation*

**Response**: We have rewritten this section to remove the repetition.

*Ln261: It is unclear why lexical tones lacking F0 variation would be predicted to make the emotional tone less perceptible in the first place. Can it be the other way around? That is, since lexical tone has no variation, would variations of emotion prevail?*

**Response**: We thank Dr. Liu for this insight. This statement was intended to be a summary of Wang et al. (2015, 2018) reviewed earlier. We have rewritten this section to include both the authors’ hypothesis and the reviewer’s interpretation.

*Experiment/Analyses

Ln310: Some tables are a little bit difficult to process although the meaning is clear. I do not have suggestions here because the authors have already given careful thoughts on the presentation of these tables.*

**Response**: We thank Dr. Liu for understanding our effort to summarize the wide range of findings in the literature. We have added more information to the caption of this table and others to clarify the meaning of the symbols and notations. *Ln318: Why is 4 a good number of participants as the baseline for the acoustic characteristics of emotional tone?*

**Response**: Ideally, the more participants, the better. However, our funding only allowed us to recruit a maximum of eight (4 female and 4 male) professional actors. To our knowledge, this number is comparable to those used in similar studies. *Ln537-540: Please rephrase to reduce repetitiveness.*

**Response**: We have rewritten this section to remove the repetition. *Ln566: Please rephrase "In addition, we also"*

**Response**: We removed “also” from this sentence as instructed.

*Discussion

Regarding "Context": There can be several possibilities to understand this word in the “context” of the current paper. It may be worthwhile specifying which context the authors are referring to (carrier sentences).*

*Also, what the context adds to/change the picture needs to be spelt out more overtly (e.g., ease of semantic parsing and/or tonal coarticulation? If one, why not the other?)*

*One methodological issue is to what extent the discrepancy between context emotion (no emotion if I understand correctly) and target word emotion would affect the current findings in the context condition. This needs to be discussed.*

**Response**: We thank Dr. Liu for this important observation. Reviewer 2 also made a similar comment. We have clarified what we mean by “context” throughout the manuscript.

***Reviewer #2 comments:***

*1. Is the manuscript technically sound, and do the data support the conclusions?*

*a. The current study vs. directly relevant past studies: This study is a potentially very valuable addition to the line of research on the influence of emotion on the production and perception of lexical tones in Mandarin. Different from past studies, the present study looks at the influence of emotion on lexical tones in both production and perception. However, this does not mean that past work with a focus on either the production side of the coin or the perception should be disconnected from the current study. In other words, the authors did not seem to make a sufficient use of existent findings to motivate their study and develop the hypotheses and predictions. Specially, the authors focused on how the realisation of each of the five basic emotions impacts the f0 mean, f0 range, intensity and duration of the target syllable. The literature review 'how emotional tones affect speech acoustics' only discussed the differences in the degree to which an acoustic parameter is exploited to express emotions between tone and non-tone languages. The authors referred to 'the literature reviewed' when presenting their predictions for changes in f0, duration and intensity (lines 300-302). But past studies on how emotion expression influences the f0 and duration of each lexical tone in Mandarin was not reviewed. Furthermore, although research on the effect of emotion expression on the perception of Mandarin tones by native speakers of Mandarin and second language learners of Mandarin is small in numbers, there has been some published work on this topic, e.g. Liang & Chen (2019), which was published in the ICPhS 2019 proceedings. This work was mistakenly cited as Liang and Chen's MA thesis in the references section, summarised in the section 'How emotional tones affect speech perception' and subsequently dismissed. This is a pity because the authors could have built their work on the findings from Liang and Chen, especially considering the fact that they had the same research question on the production side as Liang and Chen, and used a similar method in both Experiment 1 and Experiment 2 to Liang and Chen’s methodology. The main differences concerning production elicitation seems to be that Liang and Chen used phototactically legal non-words as target words and systematically varied the tonal contexts preceding the monosyllabic target words whereas the current study used real words and had only one tonal context, i.e. the monosyllabic target words were preceded by tone 1 in the carried sentence. Liang and Chen's findings can thus provide a very useful starting point for the current study in terms of both hypothesis formulation and methodology.*

**Response**: We thank the reviewer for this important advice on grounding the current study in the literature. We agree that the study by Liang and Chen (2019) offers an excellent foundation for this study. We have revised the introduction, results, and discussion to highlight how Liang and Chen’s (2019) study has been used to develop our research questions and hypotheses, and how our results compared to Liang and Chen’s (2019) findings.

We also agree that a more meaningful connection should be made with previous studies focusing on either production or perception. We have rewritten the introduction and discussion to enhance the connection.

Re: “But past studies on how emotion expression influences the f0 and duration of each lexical tone in Mandarin was not reviewed.” Thank you for the comment. We have added a summary of Chao’s (1933) description of how emotions are implemented in Mandarin tones. We have also included a discussion of Li, Fang, and Dang’s (2011) acoustic study.

Finally, we apologize for the confusion between Liang and Chen’s ICPhS proceedings and Liang’s MA thesis. We had referenced the thesis in the original version because the thesis seemed to provide more details of the study. In light of the reviewer’s feedback, we have used the ICPhS citation throughout the manuscript. *b. Taiwan Mandarin is prosodically not the same as Beijing Mandarin (e.g. in prosodic realisation of focus), even though they both have the same four lexical tones. This difference is not sufficiently acknowledged throughout the study. Notably, the similarities in findings between this study and those from Liang and Chen (2019) can have interesting implications for the prosodic similarities and differences regarding the interaction between emotion and lexical tones between Taiwan Mandarin and Beijing Mandarin.*

**Response**: We thank the reviewer for bringing to our attention this comparison. We have acknowledged this difference as a limitation of the current study.

*c. Experiment 1: The participants were specifically instructed not to place 'excessive stress on the target syllables'. This seems to suggest that the participants have no uniformed way to determine the information structure of each target sentence. They could have treated each target sentence as a response to a 'what-happens'-like question, rendering the whole sentence focal (broad focus) or treated the target word as the focus (narrow focus) in spite of the instructions. What the participants did regarding the information structure of the target sentences may influence how they used prosody to realise emotion and tones. For example, they might have distributed the prosodic changes over the whole sentence if they had a broad-focus analysis, or the prosodic changes might have concentrated in the target syllables if they had a narrow-focus analysis.*

**Response**: We thank the reviewer for this insight. Our intention was to elicit a broad focus, i.e., distributing the prosodic changes over the whole sentence, instead of narrowly focusing on the target word. We instructed the participants to avoid placing excessive stress on the target word because the carrier phrase was the same for different target words, and we worried there would be a tendency for the speakers to emphasize the target word. We have revised the description to clarify that the instruction was intended to elicit a broad-focus analysis.

*d. Experiment 1: The recordings from the participants were rated by 30 native speakers of Taiwan Mandarin. No information was given on who these participants were and how they were recruited. Were they the same participants who did Experiment 2?*

**Response**: Yes, the same participants also participated in Experiment 2. We apologize for not specifying this information in the original manuscript. We have added this information in the revision. Also, there were actually 36 participants for the rating task. We apologize for the error. We have made corrections throughout the manuscript.

*e. Experiment 2: Experiment 2 hinged on two opposing assumptions. Neither is well motivated. One of the assumptions was that ‘emotional tones alter the canonical acoustic characteristics of Mandarin tones, making it difficult for listeners to retrieve the Mandarin tones’ (Lines 554-556). The authors did not specify anywhere in the manuscript what the ‘canonical acoustic characteristics of Mandarin tones’ should be. I do not think that past studies have provided evidence for such a claim either. Instead, it has been shown that the changes in the acoustic realisation of lexical tones related to the expression of emotions are restricted to the gradient changes in terms of higher of lower mean pitch, smaller or wider pitch range, or longer or shorter duration. Such changes are phonetic by nature because lexical tones are not defined by absolute pitch, duration and intensity. The other assumption is that ‘listeners are able to disaggregate the acoustic changes into two sources – Mandarin tones and emotional tones’. This assumption is itself based on the assumptions that lexical tones have absolute f0, duration and intensity values, which is not the case, and the perception of emotion prosody is categorical, for which evidence has emerged (e.g. Laukka 2005).

Laukka, P. (2005). Categorical perception of vocal emotion expressions. Emotion, 5(3), 277–295.*

**Response**: We thank the reviewer for these observations. We certainly agree that lexical tones are not defined by absolute pitch, intensity, or duration. The acoustic characteristics of tones vary across talkers, phonetic contexts, and emotions. Our use of the term “canonical” was meant to highlight that emotions, like talkers (e.g., Moore and Jongman, 1997), tonal contexts (e.g., Xu, 1997), and other sources of acoustic variability, may change the citation form of lexical tones. The gradient acoustic changes noted by the reviewer illustrate exactly the idea. It was not our intention to claim that a lexical tone is associated with an ideal set of acoustic characteristics. In light of the reviewer’s comment, we have removed the term “canonical” and rephrased this statement to avoid that impression.

Our use of the term “disaggregate” was intended to highlight that the listener’s task is to interpret the acoustic signal by taking into consideration possible sources of acoustic variability. That is, listeners need to analyze the acoustic signal in terms of lexical tones, emotional tones, and other sources that contribute to the acoustic variability. This is analogous to interpreting lexical tones by considering talker F0 range (e.g., Moore and Jongman, 1997). It was not our intention to assume a lexical tone is associated with an ideal set of acoustic characteristics. We have rephrased this statement to avoid that impression.

We thank the reviewer for bringing Laukka (2005) to our attention. Since our study was not designed to test whether emotions are perceived categorically or not, we did not include the study in the references.

*f. The factors ‘context’ vs. ‘isolation’ in Experiment 2: The target words were presented to the participants in both their original carrier sentence and out of their original carrier sentence (e.g. cut out of the carrier sentences) to study ‘how the presence of context would affect the identification of Mandarin tones and emotion tones’. The latter was referred to as the isolation condition. The authors did not offer any hypotheses or predictions for the effect of context based on current understanding of the effect of tonal coarticulation on tonal perception and that emotion expression can affect the prosody of the whole sentence.

It should also be pointed out that the isolation condition cannot be representative of how a lexical tone is produced in isolation, i.e. with no neighbouring sounds, or how an emption is expressed in a monosyllabic word with no neighbouring sounds. When a monosyllabic word is produced in isolation, it is produced as a one-word utterance, prosodically speaking. It has no tonal coarticulation and carries all the prosodic adjustments that need to be made to express a certain emotion.

Besides, in the ‘context’ condition, the target words were preceded by a Tone-1 word and followed by neutral tone-word in all sentences. Can the current results be generalised to other tonal contexts? For example, what happens when the target word is preceded by a Tone-2, Tone-3 or Tone-4 word?*

**Response**: We thank the reviewer for this important observation. Reviewer 1 also made a similar comment. We apologize for not specifying the hypotheses regarding context in the original version. We have elaborated on our characterization of context throughout the manuscript. We have also added specific hypotheses to the section “the present study”.

We agree that the “isolation” condition does not represent how a lexical tone is produced in isolation. As the reviewer correctly pointed out, the “isolated” syllables were not produced in isolation; rather, they were excised from a sentence with the carrier phrase. We have revised the manuscript to clarify this and the nature of the isolation vs. context comparison.

We agree with the reviewer’s observation about generalization. Since the carrier phrase was the same for all target syllables, our results cannot generalize to other tonal contexts. On the other hand, Liang and Chen (2019) systematically manipulated the preceding tone. We have revised the manuscript to acknowledge this limitation.

*g. Experiment 2, lines 609-615: The authors stated that ‘the order of the stimuli within each block was randomized for each participant’. How was this done exactly? Take the block of isolated syllables for tonal identification for example. What kind of principles were used in the randomization?*

**Response**: We used the Random Number Generator in LabVIEW (National Instruments) for randomization. Each of the 120 syllables was assigned a number ranging from 1 to 120. The Random Number Generator was then used to create a unique presentation order for each participant. We have added this description to specify the randomization procedure.

*h. Experiment 2: I don’t understand why stimuli produced in the neutral emotion were not included in the task on perception of emotion. Excluding this option might potentially increase the chance of choosing the right emotion from 20% to 25%.*

**Response**: Thank you for this observation. We had intended to have 4 response options for both Mandarin tone identification (4 tones) and emotion recognition (4 emotions), but we agree we should have included the neutral emotion in the emotion recognition task. We have acknowledged this limitation in the manuscript.

*i. The discussion on the differences in the context and isolation conditions was somewhat confusing. The authors seemed to acknowledge that there were prosodic cues to emotion outside the target word in a sentence in some places (e.g. lines 829-830). But in the conclusion section, the authors attributed the better performance in the context condition to listeners’ knowledge of tonal co-articulation (lines 843-844). This is true for the perception of tones but not true for the perception of emotion.*

**Response**: We thank the reviewer for this observation. As acknowledged earlier, context was indeed not explained sufficiently in the original version. We have revised the manuscript to clarify the interpretation of context.  *2. Has the statistical analysis been performed appropriately and rigorously?*

*a. The data from Experiment 2 were categorical by nature: 4 categories for identification of lexical tones; 4 categories for identification of emotions. These data should thus be analysed using methods that can deal with categorical data. Repeated measures ANOVA, which was used in the paper, is not the appropriate analysis. The authors can consider using the mixed-model multinomial regression analysis in R.*

**Response**: We thank the reviewer for pointing this out. As instructed, we have redone the analysis by using a mixed-model multinomial regression analysis in R. We have rewritten the results and discussion based on the updated analysis.

*b. The effect of emotion on the shape of the f0 contours of the lexical tones was discussed based on visual observations (lines 380-390). A more rigorous and more appropriate approach would be to use the Functional Data Analysis to mathematically quantify the shapes of each contour in terms of functional principle components and then use the fPCs as dependent variables.*

*Gubian, M., Torreira, F., & Boves, L. (2015). Using Functional Data Analysis for investigating multidimensional dynamic phonetic contrasts. Journal of Phonetics, 49(0), 16–40.* [*https://doi.org/http://dx.doi.org/10.1016/j.wocn.2014.10.001*](https://nam11.safelinks.protection.outlook.com/?url=https%3A%2F%2Fdoi.org%2Fhttp%3A%2F%2Fdx.doi.org%2F10.1016%2Fj.wocn.2014.10.001&data=04%7C01%7Cleec1%40ohio.edu%7Cbf2d863f57f745c7e70f08d9af362ee2%7Cf3308007477c4a70888934611817c55a%7C0%7C0%7C637733470747318166%7CUnknown%7CTWFpbGZsb3d8eyJWIjoiMC4wLjAwMDAiLCJQIjoiV2luMzIiLCJBTiI6Ik1haWwiLCJXVCI6Mn0%3D%7C2000&sdata=%2FaSsitUJK0IfSXqHu7%2F3IklFRWQ%2BG5csRU5bDbEhMeo%3D&reserved=0)

*Chen, A., & Boves, L. (2018). What’s in a word: Sounding sarcastic in British English. Journal of the International Phonetic Association, 48(1).* [*https://doi.org/10.1017/S0025100318000038*](https://nam11.safelinks.protection.outlook.com/?url=https%3A%2F%2Fdoi.org%2F10.1017%2FS0025100318000038&data=04%7C01%7Cleec1%40ohio.edu%7Cbf2d863f57f745c7e70f08d9af362ee2%7Cf3308007477c4a70888934611817c55a%7C0%7C0%7C637733470747328162%7CUnknown%7CTWFpbGZsb3d8eyJWIjoiMC4wLjAwMDAiLCJQIjoiV2luMzIiLCJBTiI6Ik1haWwiLCJXVCI6Mn0%3D%7C2000&sdata=I%2B9Z1ZUZVl0Y2UQj4cTc0L6xAIVhiPfLxgDwPAbrULA%3D&reserved=0)

**Response**: We thank the reviewer for pointing this out and offering the references. We agree that the Functional Data Analysis would be the most rigorous approach for a quantitative analysis of dynamic F0 contours. However, the focus of our acoustic analysis was the four measures (mean F0, F0 range, mean amplitude, and duration), not the F0 contours. Our purpose of including the figure and qualitative description was to show that the four tones were produced as intended. We certainly agree that analyzing the dynamic F0 contours would be quite informative. However, we feel it is best to leave the analysis to a separate study because including such an analysis would make our paper even longer than it already is.

Finally, as the reviewer pointed out earlier, the acoustic realization of lexical tones in the expression of emotions is typically restricted to gradient changes in terms of mean pitch, pitch range, or duration. We agree with the reviewer that these acoustic measures would offer more insights for the purpose of this study. We have revised this section to acknowledge that our observations are qualitative in nature. We have also included the reference offered by the reviewer.

*c. Crucial details are missing on the f0 contours presented in Figure 1: How were the f0 contours of the lexical tones in Figure 1 determined? Were the mean f0 contours of all speakers? If so, how were they extracted? Were they time-normalised?*

**Response**: We apologize for missing these details. We have added a description of how the F0 contours were determined. Each F0 contour was indeed generated by averaging over all speakers of the same sex. The contours were also time-normalized as the reviewer pointed out.

*d. Statistics on all the pairwise comparisons should be provided for each analysis.*

**Response**: We have made sure that all pairwise comparisons are provided where applicable.

*3. Have the authors made all data underlying the findings in their manuscript fully available?*

*The authors stated that ‘All relevant data are within the manuscript and its Supporting Information files.’ I did not find out how to access Supporting Information in PLOS One. It seems that I could only download the manuscript. I ticked the ‘Yes’ box for this question but I’d like acknowledge that I do not have the information to judge on the availability of the data in Supporting Information that has not been presented in the manuscript.*

**Response**: We apologize for missing the data in the original submission. We have supplied a minimal data set as instructed.

*4. Is the manuscript presented in an intelligible fashion and written in standard English?*

*I fear that the clarity of the manuscript has been affected by the presentation and exposition, even though there are very few language errors. The major issue I have with the writing is that the authors regularly made claims without substantiating them. A few examples:
a. The authors sketched four possible scenarios for how emotion may affect the production and perception of tones on pp. 4-5 (lines 70-86). These scenarios appear poorly informed given what has already been known.*

**Response**: We agree that the four scenarios were not grounded in the literature and did not contribute to motivating the research questions. Reviewer 1 also made a similar comment. We have removed this paragraph and subsequent discussions that were based on this taxonomy. Instead, we have followed the reviewer’s suggestion to use Liang and Chen (2019) as the foundation of this study. *b. Lines 98-99: ‘Despite the universal appearance of emotion tones, their acoustic correlates vary across studies.’ I do not understand claims such as this one that seem to contain contradictory information. In this particular case, the review of the literature in the preceding lines clearly pointed to cross-linguistic differences in how f0, duration, intensity are varied to express the same emotion.*

**Response**: We apologize for the confusion. We have rephrased this statement to resolve the contradiction. *c. Lines 167-169: These lines were supposed to summarise the main findings reviewed in the same paragraph. However, the preceding review did not talk about discrimination of consonants and recognition of spoken words. The findings reviewed were mostly concerned with recognizing word meaning.*

**Response**: By “discrimination of consonants” we were referring to Mullenix et al. (2002), who manipulated the final consonant of the names (e.g., Todd-Tom). By “recognition of spoken words”, we were referring to Mullenix et al. (2002), Kitayama and Ishii (2002), Ishii et al. (2003), Nygaard and Lunders (2002), Nygaard and Queen (2008): they all used some form of a word recognition task. This summary is no longer in the revised manuscript due to extensive rewriting.

*d. Lines 175-184: These lines were supposed to give more details on Singh et al.’s work after the summarising sentence on what their work was about in lines 173-175. However, what was presented in lines 175-184 was hard to follow and did not really match the summarising sentence.*

**Response**: We apologize for the lack of clarity. We have rewritten the description to clarify what was done in this study.

*e. Lines 195-197 on the limitation of Liang and Chen’s study: “Since no acoustic analysis was reported, it is not clear to what extent these findings could be explained by the actual acoustic difference among the emotional tones.” I am not sure I can follow the logic here. Liang and Chen had the stimuli rated by a native speaker of Mandarin in terms of emotion before presenting them to their participants. The emotions were correctly recognised in all cases. This would allow the authors to conclude that emotions were accurately produced in the stimuli and differences found in the perception of tones in different emotions could be attributed to the differences in prosodic realisation of emotions.*

**Response**: We thank the reviewer for this observation. We agree that ratings by native speakers constitute valid evidence that emotions are produced as intended. We were simply pointing out that no acoustic analysis was reported in the study. We have removed this statement to avoid the impression that the stimuli were not valid.

*f. Lines 231-232: “Although emotional tones appear to be perceived in a similar way irrespective of specific languages, it is not clear whether the use of lexical tones in tonal languages affects the perception of emotional tones.” Immediately following these lines, the authors reviewed Wang et al.’s study on exactly this topic. Their findings seem quite straightforward to me. What is still not clear and should therefore be investigated in the current study then?*

**Response**: We apologize for the confusion. We have revised this statement to resolve the contradiction.

*g. Lines 271-273: I have the same concern as with lines 231-232.*

**Response**: We apologize for the confusion. We have removed this sentence from the revision.

*h. Lines 256-259: “Evidence from L1 and L2 studies of segmental and tonal perception further suggests that tonal language experience affects how lexical tones and segmental structure are processed when they are produced with emotional tones.” Such studies were not reviewed in the preceding sections.*

**Response**: This statement was a summary of Singh, Lee, and Goh (2016), and Liang and Chen (2019). Both studies showed that tonal language experience affected perception of speech produced with emotions. This summary is no longer in the revised manuscript because of extensive rewriting.

*i. Lines 673-676: The description of the results (Tables 8, 9) was not accurate.*

**Response**: We have revised these statements to make sure the description is accurate.

*j. Lines 746-747: The description of the result was not accurate.*

**Response**: We have revised these statements to make sure the description is accurate.
