# Supplementary material for: Emotional tones of voice affect the acoustics and perception of Mandarin tones
Source: PLoS One. 2023 Apr 5;18(4):e0283635. doi: 10.1371/journal.pone.0283635 (PMC10075469; doi:10.1371/journal.pone.0283635)
Supplement: Attachment — Submitted filename: Response to Reviewers.docx [file pone.0283635.s006.docx]

**Comments to the Author**

Reviewer #3: In line with the previous two reviewer’s general comments, I agree that the current work is relevant and can be a valuable addition to our current knowledge base.

We thank Dr. Chong for recognizing the contribution of this study.

1. Is the manuscript technically sound, and do the data support the conclusions?

A) Reviewers 1 and 2 both made similar remarks about the disconnect between the broader motivations of the study under review and how it is positioned within the context of similar research. Reviewer 2 recommended that the authors use the work of Liang and Chen (2019) as an anchor point for the current manuscript.

The authors have done well to incorporate the reviewers comments and the Liang and Chen (2019) study is given prominence in the introduction.

However, given the importance of the study as the cornerstone of the current work, this manuscript will benefit from a revision to provide greater clarity and details of the Liang and Chen study. In its current form, I feel that a reader will need to first review Liang and Chen’s thesis in order to follow this manuscript.

As instructed, we have provided more details of Liang and Chen’s study in the introduction (lines 201-216).

B) Reviewer 1 asked why the authors adopted a four basic emotion model (angry, happy, fear and sad) instead of a six emotion model (which on top of the four aforementioned emotion types, also include surprise and disgust).

The authors replied that the decision was made on the basis that most studies on Mandarin have adopted the four emotion model, hence using the four emotion model would make comparisons with previous studies more straightforward.

I am not sure if I quite follow the logic of this reasoning. As the four emotion model is a subset of the six emotion model, straightforward comparisons can still be made if the current study adopted the six emotion model.

Moreover, using the six emotion model has a number of other benefits. One, it would allow comparisons against a much larger literature base (studies using either four or six emotion models) and potentially against the wider literature of other tonal and non-tonal languages.

The second benefit pertains to Reviewer 2’s comment about the inclusion of Neutral as a possible response option for Experiment 2 to reduce chance level accuracy (from 25% to 20%). A six emotion model would achieve a similar effect by having two additional emotion types as response options.

A third advantage, which I feel is more critical, relates to the aim of study. Fundamental Frequency (F0) is considered to be one of the most salient carriers of emotion information as it is argued that F0 may be an acoustic correlate of arousal (Scherer, 2003). For example, emotions with high arousal such as anger may be expressed with a higher mean F0 and conversely, emotions with low arousal such as sadness may be expressed with a lower mean F0. In this regard, of the four emotions examined in this study, only one (Sad) is considered to be of low arousal and the study may therefore benefit from the inclusion of a larger range of emotion types of varying arousal levels to allow a more nuanced study of the interaction between emotions and lexical tone production.

We thank Dr. Chong for pointing out the benefits of adopting the six-emotion model. Since the study has been implemented with four-emotion speech materials, we believe the best we can do at this point, without re-running the entire the study, is to offer a broader theoretical foundation and acknowledge the limitation of the four-emotion model. To these ends, we have added a description of theories of emotion in the introduction (lines 70-93). We have also added a description in the conclusion to acknowledge the limitation of the four-emotion model (lines 783-791).

C) Reviewer 2 raised a question regarding the randomisation procedure that was undertaken within each experimental block in Experiment 2. Similarly, I have a concern regarding randomisation but of the experimental blocks. From my reading, I understand that the order of block presentation was fixed for all participants such that they always viewed stimuli in the isolation condition before the context condition.

I am curious about the authors reasoning for this. I feel that this is may be a weakness as confounds such as training or exposure may have an unwanted impact on the observed results (e.g., participants have familiarised themselves with the stimuli by the time they get to the context condition, hence resulting in higher identification accuracy). This is given greater scrutiny especially since the authors are making claims based on the comparison of the isolation to the carrier condition.

Dr. Chong is correct that the isolation condition was always presented before the context condition. We agree that familiarization from the isolation blocks could have boosted accuracy in the context blocks. In hindsight, we could have randomized the order of the blocks to mitigate this concern. We have acknowledged this limitation in the conclusion (lines 803-805). We have also added a paragraph in the introduction regarding the benefit of context (lines 266-292).

D) Reviewer 2 asked why Neutral stimuli were not included in the emotion perception task as the exclusion of this option had raised chance level accuracy rates from 20% to 25%.

The authors responded on page 33, third sentence from the top of the page (pardon the clunky referencing but there doesn’t seem to be line numbering on the revised version), that ‘Neutral was regrettably not included’. I think it would be more meaningful to justify why Neutral was not included, rather than an admission that it was not there.

Nevertheless, in my opinion, the exclusion of Neutral has the benefit of making this a forced choice task that prevented the potential abuse of Neutral as a bin for stimuli that participants were uncertain about.

However, my concern is about why Neutral as a response option was excluded when Neutral stimuli were presented to the participants in the emotion identification task. From my reading, it appears that Neutral stimuli were presented in every experimental block (each experimental block consists of 120 stimuli, 3 syllables, 4 tones, 5 emotions, and 2 repetitions – bottom of pg. 31). Does this also mean that the participants would be giving an incorrect response to the Neutral stimuli by default simply because the Neutral option was not available?

We apologize for not including line numbers in the previous revision. We have added line numbers to this revision.

We thank Dr. Chong for pointing out the benefit of excluding the NEUTRAL response option. In fact, NEUTRAL stimuli were also excluded from the emotion recognition task—we apologize for not making this explicit. That is, in the emotion recognition task, the NEUTRAL stimuli were excluded, resulting in 192 stimuli (3 syllables, 4 tones, 4 emotions, 2 repetitions, and 2 talkers) per block (isolation block and 1 context block). In the tone identification task, all five emotions were included, resulting in 240 stimuli (3 syllables, 4 tones, 5 emotions, 2 repetitions, and 2 talkers) per block. We have revised the description to clarify the design (line 569-574).

E) Both reviewers had raised issues regarding the unclear rationale behind the isolation vs. context comparisons, and the lack of clarity in the discussion of the findings.

Unfortunately, despite the revision, I am unsure if I follow the logic and significance of the comparison between the isolation and context conditions. There were no predictions or references given that would contradict the rather intuitive hypothesis and critical discussions regarding these findings still seems a little thin.

This section could potentially benefit from another rewrite to clearly state the significance of the isolation vs. context comparison, the underlying or competing mechanisms at work, and how this comparison adds to our current knowledge base. In particular, I find the second paragraph of page 29 hard to follow.

We agree that the benefit of context is rather intuitive, therefore we did not elaborate on the rationale behind the comparison in the introduction. We did present predictions regarding the comparison in Experiment 2 immediately before the method (lines 520-546). To highlight the rationale for this comparison, we have added a section entitled “Benefit of context” to the introduction. We have also moved the predictions currently in Experiment 2 to this new section.

F) Another point that is unclear to me is the motivation behind the authors’ aim to examine sex differences. From my reading, there is only one justification given, which is to extend Liang and Chen’s (2019) study where all stimuli were produced by a single female speaker (this information was not given in the current manuscript).

Despite the focus on sex differences, it is unclear whether the analyses here were meant to be exploratory or confirmatory. The manuscript does not provide adequate references or predictions on precisely how sex may have an impact on lexical and emotion tone production and why it is important that we study this.

I note that this issue was not raised by the previous reviewers. My reason for bringing this up is that, the manuscript will be easier to read if clearer objectives and deeper insights into the authors’ thought process is given. Moreover, this has implications regarding the appropriateness of the statistical tests undertaken by the authors.

From my understanding, at the broadest level, this study aims to address how emotion (not emotion and sex) affects the production and perception of Mandarin lexical tones. While it is interesting to tease apart and comment on sex differences, I feel that the logical next step, which is missing, is how these effects may be controlled for in order to draw inferences regarding the generalisability of the findings and its robustness against sex and other potential confounds arising from idiosyncratic individual differences.

In this regard, I highly recommend the use of mixed effects models where speaker, sex and perhaps even syllable type may be entered as random intercepts and slopes. This is aligned with Reviewer 2’s suggestion that mixed effects models be applied on data from Experiment 2. I am of the opinion that this should also be applied to Experiment 1 and with more rigour than what is currently done (more on this in the next section).

We agree that this study focused on the effect of emotion (not emotion and talker sex) on tone production and perception. As suggested, we have rerun the statistical analysis for Experiment 1 (acoustic analysis) using linear mixed-effects models with talker, talker sex, syllable type, and repetition as random effects.

2. Has the statistical analysis been performed appropriately and rigorously?

A. Reviewer 2 recommended the use of Functional Data Analysis for the analysis of F0 contours.

The authors agreed that the use of Functional Data Analysis was appropriate but unwarranted as the current analysis of contours was intended to be a manipulation check of sorts to show that the four tones were produced as intended.

I agree that the use of Functional Data Analysis may not be necessary if the analysis was intended to be a simple visual sanity check. However, in its current form, explorations of the contours appear to be beyond the level of a simple sanity check as contrasts between sex and emotions were made. It is therefore inaccurate for the authors to claim that the ‘qualitative observations were meant to corroborate that the tones were ‘produced as intended’ (second last sentence of first paragraph on page 19) as it is not clear what the ‘intended’ shape of the contour is for the different sexes and emotion types.

My recommendation is for the authors to acknowledge that this is an exploratory examination to visualise and better understand the stimuli produced in the current study, rather than framing this as a comparison against a ‘standard’.

We agree that the F0 plot was intended to show how the Mandarin tones were produced by talkers in this study. As suggested, we have added a note about this point and deleted statements about potential sex and emotion contrasts.

B. Reviewer 2 suggest that that mixed-model multinomial regression analysis in R may be a more appropriate analysis than the repeated measures ANOVA performed by the authors.

The authors have now applied mixed model logistic regression in their analysis of the data from Experiment 2.

More critically, I am concerned about how the variables were entered into the model in the revised manuscript. I am not sure if I understand why participants were entered as a random effect instead of variables such as sex, talker or syllable. It also unclear why the model was needlessly overcomplicated by the inclusion of the interaction effect between emotion and context when this is not the aim of the study (and also subsequently ignored by the authors – pg. 39). It would also be instructive for the authors to cite the R package that was used.

The significant interactions effects were further illustrated by the authors through the use of confusion matrices. While the findings presented are sound, I feel that an edit would provide better clarity. For example, the authors wrote ‘The most common error for Mandarin tones produced with ANGRY was Tone 4’. My suggestions would be to rephrase this to something along the lines of ‘There appears to be a response bias where Mandarin lexical tones, Tones 1 and 3 in particular, were most commonly misidentified as Tone 4 when presented in an Angry tone of voice’.

We thank the reviewer for the suggestion to clarify and simplify the regression model. As suggested, we have rerun the analysis by focusing on factors most relevant to our research questions. In particular, fixed effects now include tone, emotion, context, and tone-emotion interaction. Random effects now include talker, syllable type, and repetition.

The R packages we used included **lme4** and **car**. As instructed, we have included the following citations:

Bates D, Mächler M, Bolker B, Walker S (2015). “Fitting Linear Mixed-Effects Models Using lme4.” Journal of Statistical Software, 67(1), 1–48. doi:10.18637/jss.v067.i01.

Fox J, Weisberg S (2019). An R Companion to Applied Regression, Third edition. Sage, Thousand Oaks CA. https://socialsciences.mcmaster.ca/jfox/Books/Companion/.

As for the confusion matrices, we have also revised the descriptions as the reviewer suggested to improve the clarity of our interpretation.

C. I would also like the authors to review how the confidence interval error bars of Figures 3 and 6 were generated. Some of the CI bars are in the negative range, which although can happen through calculation but not in actual measure (e.g., F0 range). This is likely indicative of a rather unusual or problematic distribution in the data which calls into question whether sufficient data preparation and cleaning (outliers?) has been conducted prior to analysis.

Thank you for the observation. We have replaced the original figures with boxplots, which show the data distribution more clearly.

D. When conducting regression analysis, it is good practice to include within the body of the results section the statistics of the analysis such as the Beta estimates, standard errors, and Z-ratio. These are currently only available as downloadable supplementary information.

We thank Dr. Chong for this suggestion. Since the manuscript is already quite long, we feel that including detailed statistics in main text will make reading difficult. Therefore we have chosen to keep the details in the supplementary information.

3. Have the authors made all data underlying the findings in their manuscript fully available?
A. Following the provided link, I am able to view only a simplified output of the mixed effects model. No other data was found.

Thank you for this observation. We included the full output of a linear mixed-effect logistic regression model in the supporting information.

4. Is the manuscript presented in intelligible fashion and written in standard English?
A. In general, both reviewers commented to the effect that edits were required to improve the quality of the manuscript. Reviewer 2 further noted that the authors frequently made unsubstantiated claims.

The authors have made extensive rewrites with assistance from a native speaker of English who provided feedback on word choice, grammar and writing style.

It is clear that the quality of the writing has improved in the revised manuscript on account of the substantial effort put in by the authors’ and their openness to suggestions. However, it can definitely benefit from another round of polishing and editing as there are areas within the manuscript (e.g., the section on confusion matrices) that although is grammatically sound, lacks clarity.

As suggested, we have revised section on confusion matrices to improve clarity.

Reviewer #4: 

This manuscript addresses and interesting question, namely how linguist and paralinguistic cues mutually influence each other in communication. Specifically, it does this by investigating how the expression of emotion in Mandarin affects the production and perception of tones 1 to 4 and, conversely, how the production of tones 1 to 4 affects the production and perception of emotion. A tonal language such as Mandarin provides an excellent testbed for the linguistics/paralinguistics interface, because emotional expression often modulates pitch, and pitch is one of the defining features (if not the defining feature) of Mandarin tones.

Since I did not review the original manuscript, I tried to pay close attention to the original comments and the replies from the authors. However, even though I did take the original reviews as a basis, there are some new remarks and recommendations in my review.

Theoretical points
------------------

One of the points of discussion in the original reviews was how to defend the use of four emotion categories. The authors have explained that these four are the four basic emotions in Ortony and Turners 1990 paper. This is somewhat unfortunate, since this paper is a highly cited /critique/ of the whole idea of basic emotion theory (a critique deemed relevant enough for Paul Ekman to directly engage the paper in his 1992 paper). That said, and although I would appreciate a better motivation for the -small number of- emotions included in the corpus, I do not particularly mind that there are only four. The more fundamental problem is that the low number of emotions (and the ones chosen) are never discussed in terms of the consequences for recognition in the vocal domain. For example, the fact that there is only one positive emotion (happiness) and only one low aroused emotion (sadness) strongly influences the decision problem that participants face. Likewise, it also strongly influences the results of the analysis, since (for example) the acoustic profile of sadness is so much different from the others in intensity, that significant results are bound to emerge. Contrast this with a situation where other low aroused emotions such as disgust or tenderness are in the dataset and you get very different results for, say, F0 or intensity. For a balanced and transparent discussion of the meaning of the results, factors like these needs to be taken into account, both in introducing the study and in the discussion section.

Along these lines I think the exclusive focus on basic emotions is needlessly limiting. Especially considering the fact that results and conclusions are often cast in terms of positive versus negative emotions, I think the dimensional approach to emotions (e.g., Russel, 1980, Russell and Feldman Barrett, 1999 and further) should be given proper attention (for example, when describing the emotions in the study, but also when interpreting the results, which is, as mentioned often already done in terms of valence -which is notably not a property of basic emotion theory which considers all emotions orthogonal categories). A reference to Laukka 2005 (in the rebuttal letter) is somewhat misleading, since although it provides evidence for categorical (which is not the same) perception of emotions, a paper from the same author in the same year using the same dataset uses the dimensional approach (Laukka, Juslin, & Bresin, 2005).

We thank Dr. Goudbeek for this comment. Dr. Chong also raised a similar comment. As noted in our earlier response, we have added a description of theories of emotion in the introduction (lines 70-93). We have also added a description in the conclusion to acknowledge the limitation of the four-emotion model (lines 783-791).

Methodolical / Statistical points
---------------------------------

In addition to these theoretical considerations, there are some methodological issues that need to be addressed.

For the analysis of variance in experiment 1, the (statistical) design is somewhat unclear. The design is introduced with "For each of the four acoustic measures, a three-way mixed-design analysis of variance (ANOVA) was conducted [...] with emotion [] and Mandarin tones [...] as within-subject factors and talker sex [...] as a between-subject factor." What is not clear is why tone and emotion are within factors, but why other stimulus characteristics that were deemed relevant in the construction of the corpus (syllable, repetition) were not used as within factors. Statistically, this variance is unexplained variance, but in a more sophisticated analysis, these could be random effects over which the study could generalize (see Judd, Westfall, and Kenny, 2012, but also Winter and Grice, 2021). In any case, the precise number of items in the analysis should be clarified, if only because otherwise the degrees of freedom in the analysis become difficult to interpret.

We thank Dr. Goudbeek for this observation. As noted in our response to Dr. Chong, we have rerun the statistical analysis using linear mixed-effects models with talker, talker sex, syllable type, and repetition as random effects.

As a follow up to the same analysis (the mixed ANOVA of experiment 1), the authors use LSD as a post hoc test. This test does not correct in any way for multiple comparisons, thus increasing the possibility of a type I error. While there might be arguments to not correcting every multiple comparison with a bonferroni test, the choice to not correct at all (certainly with such a large number of comparisons. This is particularly relevant in light of Figure 1, where almost all CI's overlap substantially (indicating the lack of a significant difference) which is in stark contrast to the findings of the post hoc analysis.

We agree with Dr. Goudbeek about the need to control for type I error. As suggested, we have used the Tukey test in the revised statistical analysis to correct for multiple comparisons.

For the second analysis, multinomial mixed effects models are indeed the correct way to analyze this kind of data. However, mixed models also enable the inclusion of items (in addition to participants) as a random effect (again, see Judd, Westfall, and Kenny, 2012, but many others). This appears to not have been done, severely limiting the generalizability of the findings. For the revision, items should be entered as a random effect in the analysis.

Thank you for this observation. Dr. Chong also made a similar point. As suggested, we have included item characteristics (talker, syllable type, and repetition) as random effects.

It should be make more clear what participants did in the two experiments. If the same group of participants was used throughout, does that then mean that some judgments were made more than one or were some judgements reused? E.g., both experiment 1 and experiment 2 contain a rating task. Were the selected data in experiment 1 rated again or not?

The raters for Experiment 1 were different from the listeners for Experiment 2. Therefore no judgments were reused. We apologize for the confusion.

Minor issues / typo's

P17 (Abstract): Lexical tones and emotions are conveyed by a similar set of acoustic parameters; -> partly similar set (because both tones and emotions are also conveyed by parameters beyond F0)

Corrected.

P18 conveys not only -> not only conveys

Corrected.

P18 Emotional tone is defined as the vocal expression of emotion, which conveys a speaker’s affective states; -> this is a very strong statement given the discussion in the field about the status of emotion as a category and what it exactly is that vocal expression expresses. So, either more than one reference is needed, or a more nuanced statement (preferably both)

We have revised this statement and added more references (lines 49-50).

P18 When discussing Scherer's (2003) review, the high correlation between F0 and, particularly, amplitude needs to be mentioned. This is important, because using both F0 and Amplitude simultaneously as variables needs to take this interdependence into account.

We have added information about the correlation as suggested (line 54-55).

P19 A lexical tone language -> a tonal language (?)

Revised.

LN71 When compared to a neutral tone of voice … a longer duration [3-12, 24-31]: this maybe a succinct summary of the literature, but in order for it to be useful, especially given the many mutually exclusive effects (e.g. a sad voice has a narrower or wider or similar F0 range), some summary conclusion or integration is necessary (over and above “there is some consistency, but also not”). In addition: all these are comparisons of the emotion to neutral, which is severely limiting the findings, right? That should be acknowledged.

We suspect the conflicting findings arise from methodological differences between studies. We have revised the summary to elaborate on this point (lines 96-106 & lines 115-120).

We understand that comparing emotions to each other would be quite informative. Our intention was to use neutral as a baseline to keep the number of comparisons manageable. We have acknowledged this limitation in the revised manuscript (lines 803-805).

P19 Similarly, when the authors say that "The variability, however, is consistent with the idea that emotion is sociocultural in nature", they are not wrong, but this statement does not connect that well with using basic emotions as a starting point. It would -to some extent- be in line with the dialect theory of emotion (e.g., Elfenbein and Ambady, 2002), which is based on basic emotion theory. So, some more clarity about the theoretical background of the authors and this paper is needed. What is the conceptual background here?

We thank Dr. Goudbeek for this observation and the reference. We have included the reference to support the statement. Since the focus of this study is on how emotions affect the acoustics and perception of Mandarin tones, rather than the effect of culture on emotion expression, we believe this fascinating topic can be addressed in a different study.

P21 In contrast, duration varied significantly among the emotions for Mandarin but not for Italian. > Not a big issue, but this is most likely also partly due to the fact that Italian is a syllable timed language, where there is much less room for variation in syllable duration.

Thank you for this observation. We have added this point to the text.

P22 -> In which language was the study by Mullennix et al.?

It was English. We have clarified this.

P23 -> I have a hard time seeing why the study of Nygaard and Queen. is important here: the effects seemed to be semantic, but how does this connect to the tone/emotion tradeoff that is expected by the authors?

We agree that this study is not super relevant to the issue of tone-emotion relationship. However, the point of this section is to show that emotion can affect spoken word recognition, which necessarily involves processing meaning. If we removed this reference, then all studies of word recognition reviewed in this section should be removed. With these considerations, we would like to keep this reference.

P23 / P24 -> the use of the word "compromised" is somewhat ambiguous; explain how emotion compromised. Similarly for the word asymmetrical: asymmetrical in what way?

We have replaced these words with a more elaborated description.

P24 Dutch speaking learners -> of Mandarin

Corrected.

P24/25 -> The finding about intermediate learners being better seems not so relevant, unless there is a link with emotion, no?

This finding is based on proficiency-tone interaction and the effect does not involve emotion. We agree that this does not seem relevant, so we have removed this statement.

P25 "Depending on specific Mandarin tones"; this is crucial for this paper (I think) and that authors could explain more why they think different tones are affected differently by (different?) emotions (and how this plays out). If this is not possible given the available information, that should be explained, too, then.

Thank you for the observation. The tone-emotion interaction was indeed a major finding of the study. We did not have sufficient information from the literature to make predictions about which tones would be affected more by emotions. Our data also did not reveal consistent tone-specific patterns to allow meaningful speculations beyond the presence of the interaction itself.

P25 "The way emotions are perceived appears to be language-universal": this contradicts earlier statements about the sociocultural nature of emotions.

Thank you for pointing this out. This statement has been deleted.

P27 -> the juxtaposition of acoustics and perception is a bit odd, I'd use production and perception

We still think acoustics is a more precise term that describes what we did in this study, i.e., acoustic analysis of speech produced by the talkers. Speech production could be examined with physiological measures, but that is not what we did in this study.

P28 -> extracted from the carrier phrase -> in isolation

We had used “in isolation” in the original version, but one of the original reviewers asked us to clarify that the isolated syllables were extracted from the carrier phrase, not produced in isolation. We agree that “in isolation” is a more succinct term and have used it in this revision.

P29 "Considered the four most common basic emotions" -> perhaps rephrase in light of the comments made above

We have rephrased this statement to highlight different perspectives on characterizing emotions.

P29 "Based on the literature review" -> It is important to realize / flag that most (if not all) of the review concerns empirical "just so" findings, without much theoretical underpinning as to why the expected effects are predicted. There is not necessarily something wrong with that, but it is important to consider.

Thank you for the observation. We acknowledge that we did not have sufficient information to make predictions about specific tones or to interpret the tone-emotion interactions fully.

P29/30 -"We also expect emotions to be modulated by specific Mandarin tones and talker sex" -> How? And if it is impossible to say how, explain why that is.

At Dr. Chong’s suggestion, we have removed talker sex as a fixed effect.

P30 Were participants compensated in any way for their participation?

Yes, all participants were compensated. We have included payment information in the revised manuscript.

P32 Were the recordings managed by a (stage) director (see, for example, Banse and Scherer 1996) or were the actors working alone?

Before the recording, the first author discussed with the actors the emotional tones that they should aim for. The actors completed the recording in a sound-treated booth while the first author monitored the recording. We have added this information to the revised manuscript.

P32 Acoustic analysis: indicate how many recordings there were (960, I think)

Yes, there were 960 (3 syllables * 4 tones * 5 emotions * 2 repetitions * 8 speakers) speech samples in Experiment 1. We have added this information to the revised manuscript.

P32 State the aim of the rating procedure: why was it done?

The rating was done to make sure the intended emotions were actually present in the speech materials. This was explained in the section on acoustic analysis.

P32 Explain why NEUTRAL was not rated

We did not think neutral stimuli needed to be rated because they were emotionally neutral. In hindsight, we agree that it would have been a good idea to include neutral stimuli in the rating task too.

P33 name the four acoustic measures analyzed

The names of the four acoustic measures are specified in lines 402-404.

P34 the Functional Data Analysis -> Functional Data Analysis (drop the determiner)

Corrected.

P35 error bars indicate 95% interval -> the 95% interval

Corrected.

P38 and talker -> there appears to be an extra space (twice)

Corrected.

P45 The different results for stimuli with and without carrier phrase is reminiscent of work done on (speaker) normalization. Work by Holger Mitterer and Mathias Sjerps, for example, as well early work by Donald Broadbent (the filter theory)

Thank you for making the connection with talker normalization. We have included this idea in the section on the benefit of context in the introduction.

P48 The NEUTRAL emotion -> NEUTRAL category

Thank you for the suggestion. By “NEUTRAL emotion” we meant NEUTRAL as an emotional tone of voice (defined in lines 46). Since Mandarin has a neutral (lexical) tone, we feel that labeling NEUTRAL as an emotion avoids the potential confusion with the lexical tone.

P49 in the carrier phrase -> when preceded by a carrier phrase

We have changed this phrase when it first appears in line 291-292.

P49 As indicated, (random) item effects should be incorporated in the analysis

Yes, as noted earlier, we have included item characteristics as random effects.

P50 "we focus on two interactions" -> but the second one (tone-context) is not really statistically analyzed or introduced (while the other one is).

In the revised modeling, we examined only the tone-emotion interaction in light of Dr. Chong’s suggestion. Therefore we are no longer discussing the tone-context interaction.

References

Elfenbein, H. A., & Ambady, N. (2002). On the universality and cultural specificity of emotion recognition: a meta-analysis. Psychological bulletin, 128(2), 203.

Judd, C. M., Westfall, J., & Kenny, D. A. (2012). Treating stimuli as a random factor in social psychology: a new and comprehensive solution to a pervasive but largely ignored problem. Journal of personality and social psychology, 103(1), 54.

Winter, B., & Grice, M. (2021). Independence and generalizability in linguistics. Linguistics, 59(5), 1251-1277.

Laukka, P., Juslin, P., & Bresin, R. (2005). A dimensional approach to vocal expression of emotion. Cognition & Emotion, 19(5), 633-653.
