# Supplementary material for: Emotional tones of voice affect the acoustics and perception of Mandarin tones
Source: PLoS One. 2023 Apr 5;18(4):e0283635. doi: 10.1371/journal.pone.0283635 (PMC10075469; doi:10.1371/journal.pone.0283635)
Supplement: Attachment — Submitted filename: Response to Reviewers_20230312.docx [file pone.0283635.s007.docx]

**Additional Editor Comments**

Personally, I think how emotional tones and lexical tones interacted in the perception of Mandarin is a very interesting topic. While I agree with the previous reviewers that the experimental design is not perfect (e.g., not having all six emotions and not having a neutral response option), I believe the authors have discussed their results in an unbiased manner by stating the limitations explicitly. Given that no experiment is perfect, I think the most important point is to provide enough information so that readers can make informed judgement about the study and be inspired to conduct better experiments to clarify uncertainties. With this in mind, I am willing to support the manuscript for publication.

Thank you for recognizing the potential contribution of this study. We agree that there is always room for improvement. Thank you for supporting this manuscript for publication.

**Comments to the Author**

Reviewer #5: I generally agree with previous review comments regarding the rationale behind and the design of the study (e.g., the four-emotion model, lack of counterbalancing on the conditions). I think the authors have adequately addressed these concerns in the last round of revisions, by providing additional justification or acknowledging the limitations. But I still noticed quite a few inaccuracies in both the results and the writing, and I hope the authors can do another round of proofreading and editing. I think this request has been raised repeatedly in previous rounds of review, and the authors should take the suggestion more seriously. Below I list some of these inaccuracies together with other minor comments. (I say “some” because I might not have spotted all of them.)

We appreciate the reviewer’s detailed and thoughtful comments. We have made corrections based on the reviewer’s suggestions. We have also invited a colleague (a native speaker of English) to proofread and edit the manuscript.

1. Line 352, 557: How much is the compensation in USD?

We have converted the currency to USD: $54 (LN352) and $34 (LN557) USD.

2. Line 368: “a total of 960 stimuli for each participant”, delete “for each participant”?

Thank you for noting the redundancy. We have deleted it.

3. Line 663: “accuracy appears higher for target syllables presented than in isolation”, presented in context?

Yes, we have revised it to “accuracy appears higher for target syllables presented in context than in isolation.” Thank you for catching the missing phrase.

4. Line 673: “and the tone-emotion interaction fixed effects”, as fixed effects?

Yes, we have added “as before “fixed effects”. Thank you.

5. Line 679: “comparisons were indicates that”?

We have revised it to “comparisons indicate that.”

6. Line 702: Not all common errors are highlighted, e.g., Tone 1 SAD identified as HAPPY for 25%.

Our intention was to highlight the most common error that exceed 20%, not all errors exceeding 20%. Please see caption (LN 700-701). In this case, it is FEAR at 29%.

7. Line 705: “ranging from 86% to 99%”, ranging from 85% to 99%?

Thank you. We have updated the percentage.

8. Line 781: “but it affects the identification of specific Mandarin tones to a greater extent than it affects the recognition of specific emotions”, this is not fully correct considering that the context seemed to boost emotion recognition accuracy to a larger extent (from 21%-93% to 85%-99%; tone identification from 40%-98% to 78%-100%)?

Thank you for the observation. We have deleted this clause.

9. Figure 5: “Duration (ms)”, Duration (s)?

Thank you. We have corrected the label.

10. Figures 6, 7: Better specify tone identification /emotion recognition accuracy rather than “Accuracy”, in the figures.

Thank you. We made the changes as suggested.
